# Reprogramming of palmitic acid induced by dephosphorylation of ACOX1 promotes β-catenin palmitoylation to drive colorectal cancer progression

**DOI:** 10.1038/s41421-022-00515-x

**Published:** 2023-03-07

**Authors:** Qiang Zhang, Xiaoya Yang, Jinjie Wu, Shubiao Ye, Junli Gong, Wai Ming Cheng, Zhanhao Luo, Jing Yu, Yugeng Liu, Wanyi Zeng, Chen Liu, Zhizhong Xiong, Yuan Chen, Zhen He, Ping Lan

**Affiliations:** 1 The Sixth Affiliated Hospital, School of Medicine, Sun Yat-sen University, Guangzhou, Guangdong China; 2 Guangdong Provincial Key Laboratory of Colorectal and Pelvic Floor Diseases, Guangdong Institute of Gastroenterology, Guangzhou, Guangdong China; 3 Center for Synthetic Microbiome, Institute of Synthetic Biology, Shenzhen Institutes of Advanced Technology, Chinese Academy of Sciences, Shenzhen, Guangdong China

**Keywords:** Cancer metabolism, Post-translational modifications, Colorectal cancer, Post-translational modifications

## Abstract

Metabolic reprogramming is a hallmark of cancer. However, it is not well known how metabolism affects cancer progression. We identified that metabolic enzyme acyl-CoA oxidase 1 (ACOX1) suppresses colorectal cancer (CRC) progression by regulating palmitic acid (PA) reprogramming. ACOX1 is highly downregulated in CRC, which predicts poor clinical outcome in CRC patients. Functionally, *ACOX1* depletion promotes CRC cell proliferation in vitro and colorectal tumorigenesis in mouse models, whereas ACOX1 overexpression inhibits patient-derived xenograft growth. Mechanistically, DUSP14 dephosphorylates ACOX1 at serine 26, promoting its polyubiquitination and proteasomal degradation, thereby leading to an increase of the ACOX1 substrate PA. Accumulated PA promotes β-catenin cysteine 466 palmitoylation, which inhibits CK1- and GSK3-directed phosphorylation of β-catenin and subsequent β-Trcp-mediated proteasomal degradation. In return, stabilized β-catenin directly represses *ACOX1* transcription and indirectly activates *DUSP14* transcription by upregulating c-Myc, a typical target of β-catenin. Finally, we confirmed that the DUSP14-ACOX1-PA-β-catenin axis is dysregulated in clinical CRC samples. Together, these results identify ACOX1 as a tumor suppressor, the downregulation of which increases PA-mediated β-catenin palmitoylation and stabilization and hyperactivates β-catenin signaling thus promoting CRC progression. Particularly, targeting β-catenin palmitoylation by 2-bromopalmitate (2-BP) can efficiently inhibit β-catenin-dependent tumor growth in vivo, and pharmacological inhibition of DUSP14-ACOX1-β-catenin axis by Nu-7441 reduced the viability of CRC cells. Our results reveal an unexpected role of PA reprogramming induced by dephosphorylation of ACOX1 in activating β-catenin signaling and promoting cancer progression, and propose the inhibition of the dephosphorylation of ACOX1 by DUSP14 or β-catenin palmitoylation as a viable option for CRC treatment.

## Introduction

Metabolic reprogramming is critical for malignant transformation and tumor initiation and progression^1^. Alterations of intracellular and extracellular metabolites caused by metabolic reprogramming have profound effects on gene expression, protein modification, cellular differentiation, and the tumor microenvironment^2–5^. Metabolic enzyme acyl-CoA oxidase 1 (ACOX1), a rate-limiting enzyme in peroxisomal fatty acid β-oxidation, catalyzes acyl-CoA conversion to enoyl-CoA^6^. ACOX1 preferentially oxidizes long or very long straight-chain fatty acids^6–9^, while the related enzymes ACOX2 and ACOX3 catabolize branched-chain fatty acids and intermediates involved in bile acid synthesis^10^. Knockout of *ACOX1* promotes hepatocellular carcinoma in mice^11,12^, and overexpression of ACOX1 inhibits oral cancer progression^13^. In addition, *ACOX1* acts as a target gene of mir-15B-5p to inhibit tumor cell metastasis^14^. These studies indicate the inhibitory role of ACOX1 in cancer^11–15^. However, the role of metabolic reprogramming caused by dysregulation of the metabolic enzyme ACOX1’s post-translational modification in colorectal cancer (CRC) remains elusive.

Palmitic acid (PA), an ACOX1 substrate^7^ and a dominant fatty acid in a high-fat diet^16^, has been shown to produce energy and regulate intracellular signaling molecules involved in the development of cancer^17^. Previous studies have identified that PA promotes metastasis in melanoma, breast cancer, and gastric cancer in a CD36-dependent manner^18,19^, and also promotes the growth of prostate cancer by activating STAT3 signaling^20^. Recent research has revealed that dietary metabolite PA alters transcriptional and chromatin programs by modulating H3K4me3 in oral carcinomas and melanoma^21^. Furthermore, PA can modify cysteine residues in a process termed palmitoylation^22–24^. Increasing evidence suggests that palmitoylation of proteins (such as PDL1, GULT1, STAT3, and IFNGR1) affects protein functions and tumor progression^24–27^. Therefore, whether ACOX1-mediated PA reprogramming affects tumor progression by regulating protein palmitoylation remains unknown.

β-catenin signaling is essential for maintaining cell homeostasis and embryonic development and is related to tumor cell proliferation, apoptosis, invasion, stemness, and chemotherapy resistance^28,29^. Studies have shown that β-catenin signaling is abnormally activated in more than 90% of patients with CRC^30^. Post-translational modifications (such as phosphorylation, ubiquitination, acetylation, and glycosylation) of β-catenin have been demonstrated to regulate β-catenin signaling^31–34^. In addition, emerging evidence indicates that PA complements the β-catenin signaling activity^19^. However, whether β-catenin could be palmitoylated by PA remains unclear.

Here, we demonstrate that ACOX1 is significantly underexpressed in CRC through a systematic bioinformatics screen and propose that reprogramming of PA induced by dysregulation of ACOX1 post-translational modification promotes CRC progression by activating β-catenin signaling via PA-mediated β-catenin palmitoylation and stabilization.

## Results

### ACOX1 is downregulated and associated with progression in CRC

**Fig. 1:**
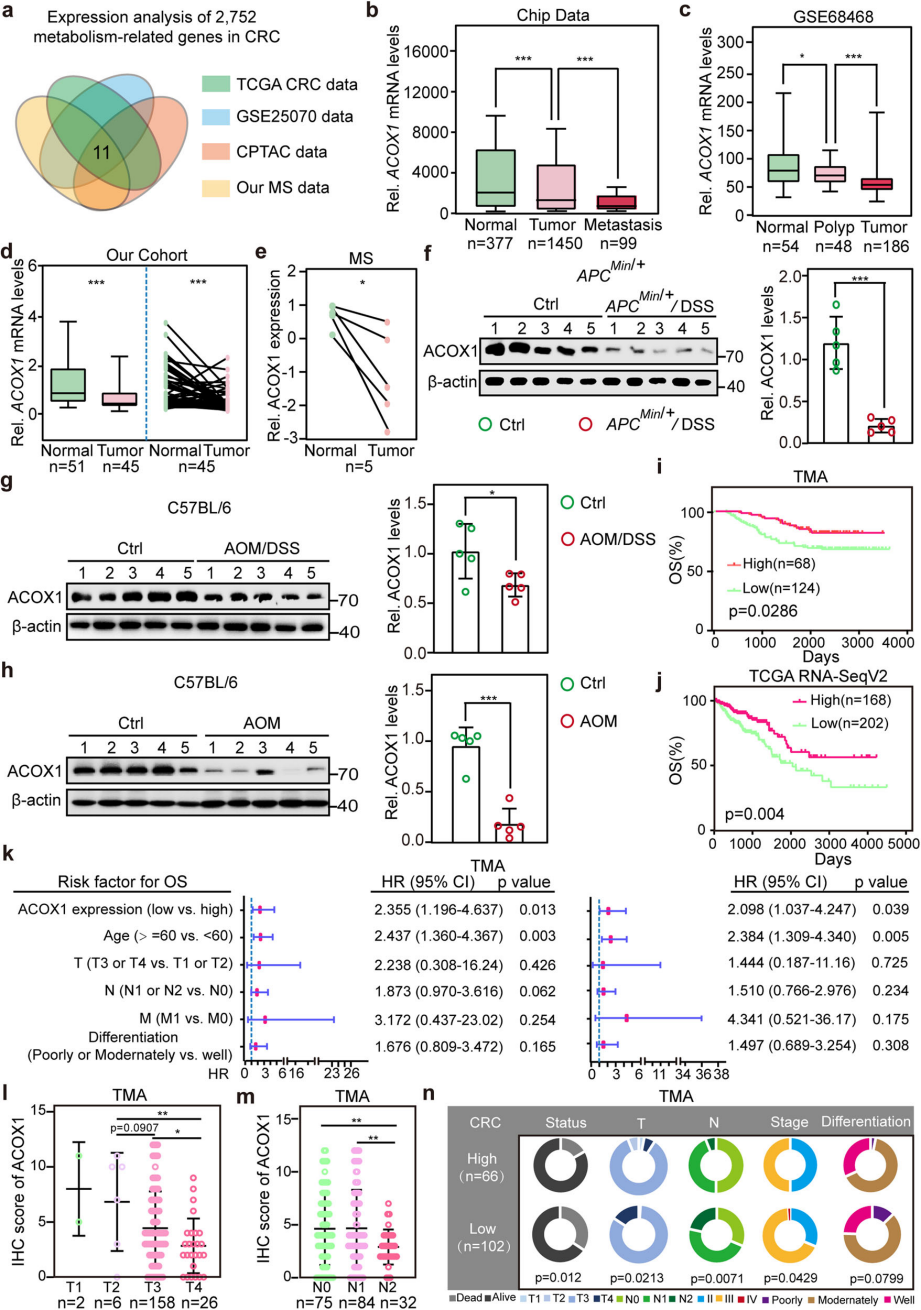
ACOX1 is downregulated and associated with progression in CRC. **a** Venn diagram exhibiting 11 differentially expressed genes (DEGs) in CRCs based on transcription levels and protein levels in the TCGA, GSE25070, CPTAC datasets, and our protein quantitative MS. **b** Analysis of *ACOX1* expression in adjacent normal tissues, primary tumor tissues and metastatic tumors from gene chip data. **c** Analysis of *ACOX1* expression in adjacent normal tissues, polyp tissues, and tumor tissues from GSE68468. **d** Unpaired and paired analysis of *ACOX1* expression in adjacent normal tissues versus primary tumor samples from the Sixth Affiliated Hospital of Sun Yat-sen University. **e** Analysis of ACOX1 protein expression in our protein quantitative MS. **f**–**h** Expression of ACOX1 protein in control colon tissues (Ctrl) and tumor samples from *APC*^*Min/+*^/DSS-inducted CRC mice (*APC*^*Min/+*^/DSS) (**f**) or AOM/DSS-inducted CRC mice (AOM/DSS) (**g**) or AOM-inducted CRC mice (AOM) (**h**) analyzed by immunoblotting (left) and quantified by densitometry (right). **i**, **j** Kaplan–Meier overall survival curves of human CRC patients with low versus high *ACOX1* mRNA or protein expression, based on CRC TMA (**i**), and TCGA RNA-SeqV2 (**j**). **k** ACOX1 expression is an independent prognostic factor for poor survival. Forest plot showing univariate (left) and multivariate (right) Cox regression analysis of different clinical parameters for CRC patients in TMA. HR, hazard ratio; CI, confidence interval. **l**, **m** Analysis of ACOX1 protein in patients with different T stages (**l**) and lymph node metastases (**m**) in CRC TMA. **n** Pie charts showing the relationship between clinicopathologic factors and ACOX1 protein expression in CRC TMA. Data were analyzed using unpaired Student’s *t*-test (**b**–**d**, **f**–**h**, **l**, **m**), paired Student’s *t*-test (**e**), log-rank test (**i**, **j**) or *χ*^2^ test (**n**). Data are presented as means ± SD; *P < 0.05, **P < 0.01, ***P < 0.001; *n*, number of patient samples.

To identify metabolism-related genes playing crucial roles in colorectal tumorigenesis, the transcriptional levels of 2752 metabolism-related genes^35^ were analyzed in at least 1000 CRCs from various datasets, including The Cancer Genome Atlas (TCGA) CRC RNA-SeqV2, TCGA CRC RNA-Seq, and Gene Expression Omnibus (GEO) (Supplementary Table S1). Additionally, protein levels of these metabolism-related genes were also analyzed in at least 100 CRCs from the Clinical Proteomic Tumor Analysis Consortium (CPTAC) dataset and our quantitative mass spectrometry (MS) of clinical samples (Supplementary Table S1). Eleven metabolism-related genes that were significantly altered in CRCs, were selected by overlapping analysis (Fig. 1a; Supplementary Table S1). Specifically, ACOX1, the only metabolic rate-limiting enzyme, was identified for subsequent analysis.

Analysis of the BioGPS Gene Expression Atlas indicated that the transcriptional level of *ACOX1* (not *ACOX2* or *ACOX3*) was notably downregulated in CRCs (Supplementary Fig. S1a). Similarly, analysis of TCGA and GEO datasets showed a significant downregulation of *ACOX1* mRNA in CRCs (Supplementary Fig. S1b). Additional datasets, such as public gene chip data^36^, TCGA and GEO databases revealed that *ACOX1* mRNA was negatively correlated with advanced disease (Fig. 1b, c; Supplementary Fig. S1c, d). Consistently, decreased *ACOX1* mRNA was also observed in early-stage CRC (TNM, Stage I, and II) (Fig. 1d; Supplementary Table S2). Importantly, the classification of CRC intrinsic-consensus molecular subtypes (iCMSs)^37^ based on TCGA transcriptomics showed that *ACOX1* expression was significantly dysregulated in iCMS2 tumor samples, where β-catenin signaling is hyperactivated, relative to iCMS3 tumor samples (Supplementary Fig. S1e–g). In addition to the transcriptomic level, a fuller analysis showed that ACOX1 protein was also markedly downregulated in CRCs (Fig. 1e; Supplementary Fig. S1h). Immunohistochemistry (IHC) analysis of our clinical samples also revealed decreased ACOX1 protein in CRCs (Supplementary Fig. S1i), further validating the result in the Human Protein Atlas (HPA) database (Supplementary Fig. S1j). Furthermore, we also found a decrease in ACOX1 protein in azoxymethane/dextran sulfate sodium (AOM/DSS)^38^, DSS (*APC*^*Min/+*^/DSS)^39^ and AOM^40^-induced mouse CRC models (Fig. 1f–h; Supplementary Fig. S1k–p). Given the low mutation frequency of *ACOX1* alleles in CRC patients (Supplementary Fig. S1q), we suggested that ACOX1 downregulation is the main cause of ACOX1 inactivation in CRC. These results confirmed that ACOX1 is poorly expressed at the transcriptional and protein levels in CRC.

Next, we evaluated our CRC tissue microarray (TMA) containing 192 CRC tissues by IHC (Supplementary Table S3), and observed that CRC patients with low levels of ACOX1 exhibited poor survival (Fig. 1i). This observation was validated in TCGA, GEO, and Vasaikar’s CPTAC^41^ datasets (Fig. 1j; Supplementary Fig. S2a–c). Univariate and multivariate Cox regression analysis was carried out to assess the importance of *ACOX1* expression for CRC prognosis together with other risk factors including age, TNM stage, or tumor differentiation. The results showed that *ACOX1* expression was an independent prognostic factor for CRC (Fig. 1k; Supplementary Fig. S2d). Moreover, ACOX1 protein expression was also significantly associated with the clinical stage, T stage, and lymph node metastases (N) of CRC (Fig. 1l–n). Collectively, these results demonstrated that ACOX1 expression is negatively correlated with the progression of CRC.

### *ACOX1* depletion promotes colorectal tumorigenesis

**Fig. 2:**
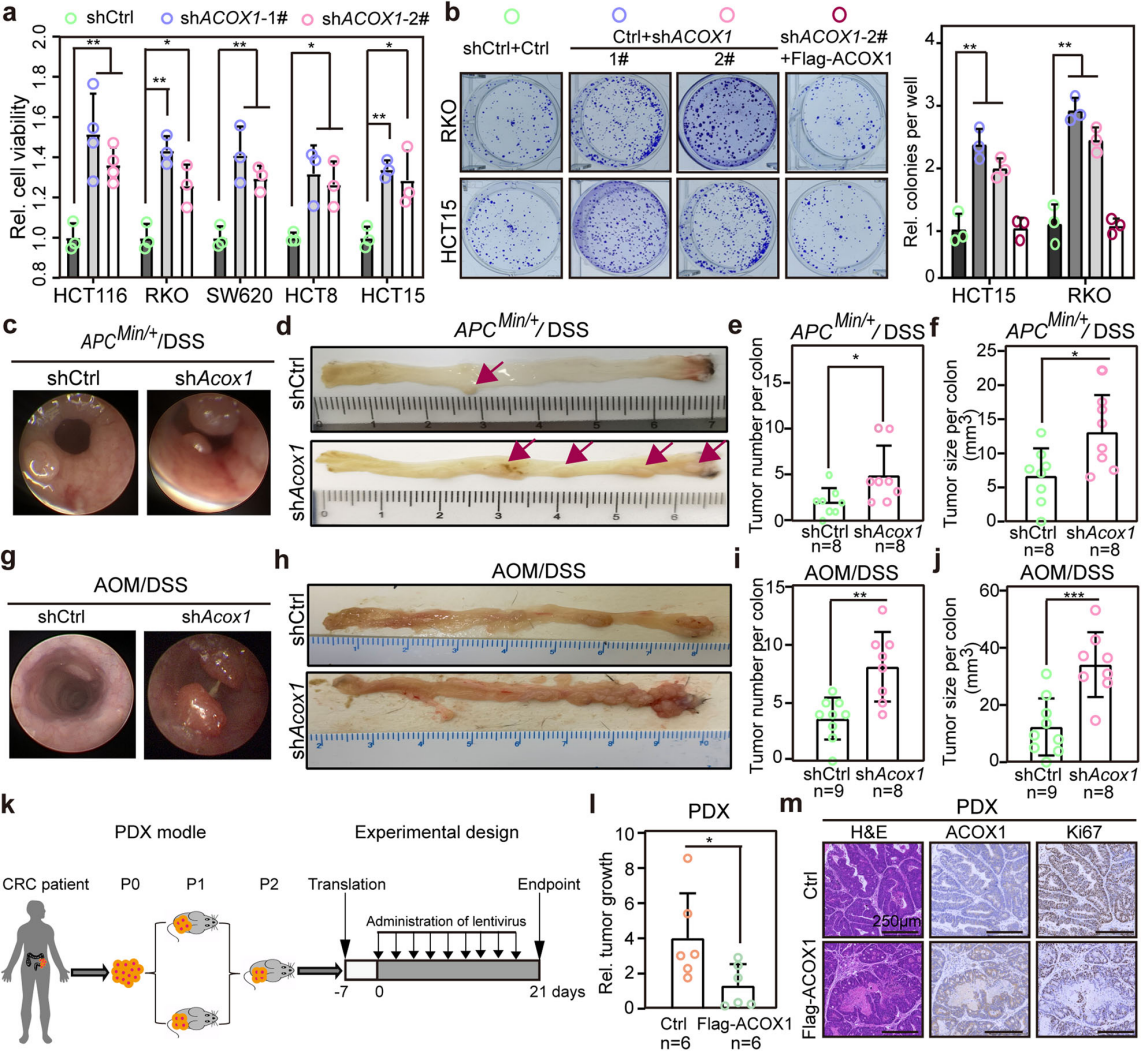
Depletion of *ACOX1* promotes CRC cell proliferation in vitro and colorectal tumorigenesis in mice. **a** Enhanced CRC cell viability by *ACOX1* depletion. Cell viability of *ACOX1*-depleted CRC cells (HCT15, RKO, SW620, HCT8, and HCT15) was analyzed for CCK-8. **b** Colony formation of RKO and HCT15 cells stably expressing the indicated vectors (left), and bar graphs showing the relative colony numbers (right). **c**–**f** Representative colonoscopy (**c**), macroscopic morphologies (**d**), tumor numbers (**e**), and tumor sizes (**f**) of *APC*^*Min/+*^ mice in control and *ACOX1*-depleted groups. **g**–**j** Representative colonoscopy (**g**), macroscopic morphologies (**h**), tumor numbers (**i**) and tumor sizes (**j**) of C57BL/6 mice in control and *ACOX1*-depleted groups. **k** Schematic diagram showing the experimental design for PDX model. **l** Suppression of PDX growth by ACOX1 overexpression. BALB/c nude mice were subcutaneously transplanted with PDXs into flanks and injected with lentivirus expressing Ctrl or Flag-ACOX1 every 2 days for 18 days. At day 21, PDXs were collected, and relative tumor growth was calculated (ratio of volume: day 21/day 0). **m** Decreased tumor cell proliferation by ACOX1 overexpression in PDXs. H&E and IHC for ACOX1 and Ki67. Data were analyzed using unpaired Student’s *t*-test (**a**, **b**, **e**, **f**, **i**, **j**, **l**). Data are presented as means ± SD; *P < 0.05, **P < 0.01, ***P < 0.001; *n*, number of mouse samples.

To define whether ACOX1 is a tumor suppressor in CRC, we ectopically expressed or silenced *ACOX1* using Flag-tagged ACOX1 or *ACOX1*-specific short hairpin RNAs (shRNAs) in CRC cell lines (HCT15, RKO, HCT8, HCT116, and SW620), respectively (Supplementary Fig. S3a, b). We observed that depletion of *ACOX1* promoted CRC cell proliferation and colony formation (Fig. 2a, b), while overexpression of ACOX1 inhibited CRC cell proliferation and migration (Supplementary Fig. S3b–d).

To further confirm that ACOX1 inhibits colorectal tumorigenesis in vivo, we built two CRC mouse models: AOM/DSS and *APC*^*Min/+*^/DSS (Supplementary Fig. S3e, f). As expected, mice with *ACOX1* depletion presented significantly more tumors, larger tumors, and markedly more histologic dysplasia (Fig. 2c–j; Supplementary Fig. S3g–j). To better elucidate the inhibitory effect of ACOX1 in CRC, we collected clinical CRC samples and constructed patient-derived xenograft (PDX) models (Fig. 2k). Consistent with the result above, ACOX1 overexpression by lentivirus inhibited tumor growth in PDX models (Fig. 2l, m; Supplementary Fig. S3k, l). These findings suggest that ACOX1 inhibits colorectal tumorigenesis in vitro and in vivo.

### DUSP14 promotes ACOX1 degradation in a ubiquitination-dependent manner

**Fig. 3:**
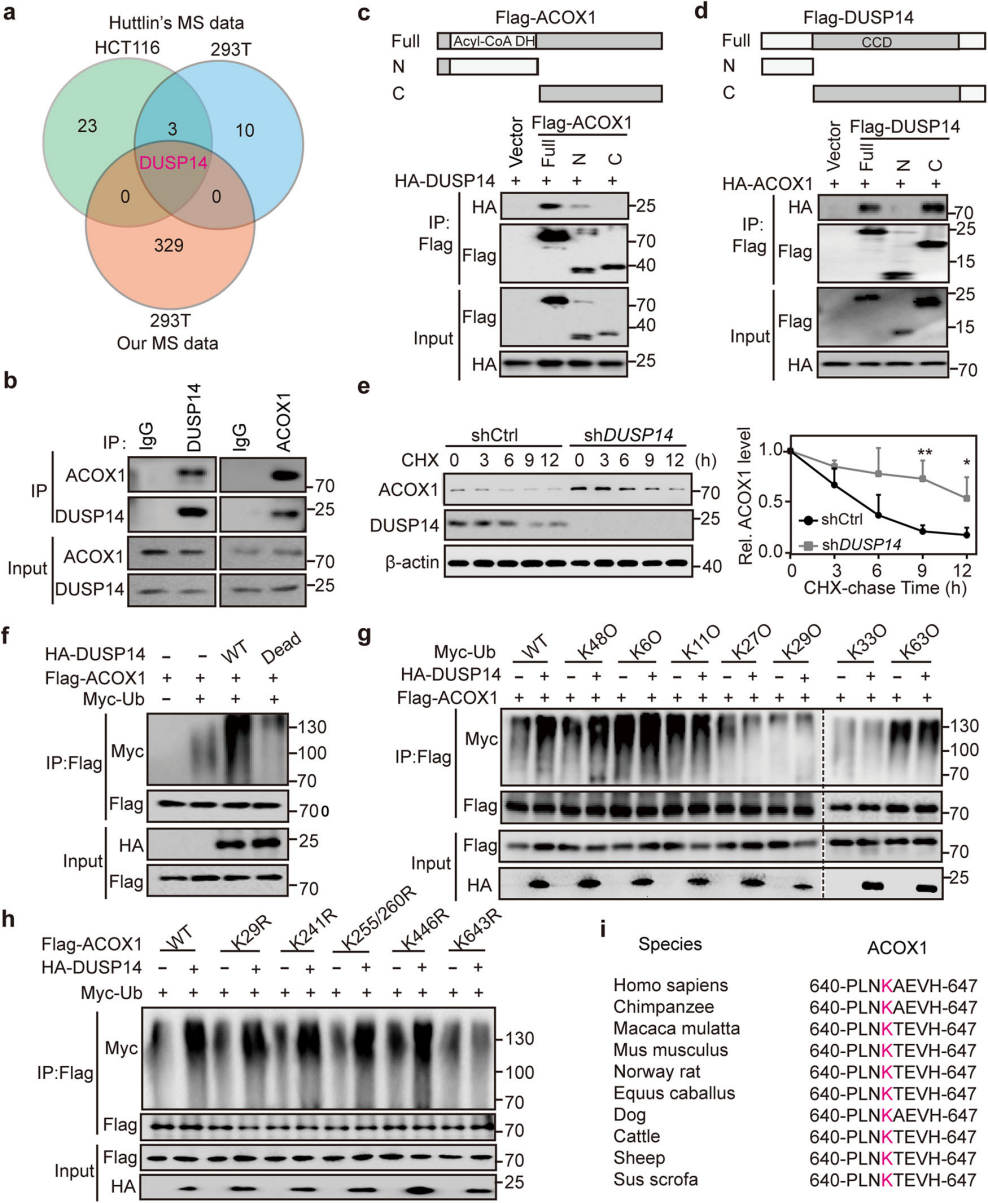
DUSP14 promotes ACOX1 degradation in a ubiquitination-dependent manner. **a** Venn diagram exhibiting DUSP14 as an ACOX1 interactor. **b** Endogenous interaction of ACOX1 and DUSP14 in HCT15 cells. HCT15 cells were treated with MG132 (20 μM) for 6 h before harvest and cell lysates were analyzed for Co-IP. **c**, **d** ACOX1–DUSP14 interaction via N-terminal domain and CCD domain. Generation of ACOX1-mutant constructs (**c**) and DUSP14-mutant constructs (**d**). HEK293T cell lysates transfected with indicated plasmids analyzed for Co-IP. **e** Time-course analysis of ACOX1 protein levels in *DUSP14*-depleted HCT15 cells (left). ACOX1 proteins quantified by densitometry, with β-actin as a normalizer (right). **f** Increased ACOX1 polyubiquitination by DUSP14 WT but not DUSP14 Dead. Myc-Ub was co-transfected with Flag-ACOX1 and HA-DUSP14 (WT or Dead) into HEK293T cells, and the cell lysates were subjected to immunoprecipitation. **g** DUSP14 mediates K48-linked ubiquitination of ACOX1. Flag-ACOX1 was co-transfected with HA-DUSP14 and Myc-Ub (WT, K6O, K11O, K27O, K29O, K33O, or K63O) into HEK293T cells, and the cell lysates were subjected to immunoprecipitation. KXO represents substitutions of arginine for all lysine resides except the lysine at X position. **h** DUSP14 mediates ubiquitination of ACOX1 at K643. Myc-Ub was co-transfected with HA-DUSP14 and Flag-ACOX1 (WT, K29R, K241R, K255/260 R, K446R, or K63R) into HEK293T cells, and the cell lysates were subjected to immunoprecipitation. **i** Alignment of lysine 643 and adjacent amino acids of ACOX1 among multiple species. Data were analyzed using unpaired Student’s *t*-test (**e**). Data are presented as means ± SD; *P < 0.05, **P < 0.01.

To uncover the functional effectors regulating ACOX1, we expressed Flag-tagged ACOX1 in HEK293T cells, immunoprecipitated the epitope-tagged protein, and analyzed the precipitate by MS. Combined with Huttlin’s MS data (thousands of cell lines (HCTT16 and 293 T) with each expressing a tagged version of a protein were lysed and immunoprecipitated, followed by MS to identify their biophysically interacting proteins)^42^, we identified DUSP14 as a candidate ACOX1 interactor (Fig. 3a; Supplementary Fig. S4a), and the endogenous interaction was further validated by Co-immunoprecipitation (Co-IP) assays in HCT15 and RKO cells (Fig. 3b; Supplementary Fig. S4b). Subsequent Co-IP assays revealed that the N-terminal domain of ACOX1 was responsible for its binding to DUSP14, while the C-terminal domain of DUSP14 was required for its interaction with ACOX1 (Fig. 3c, d). A time-course analysis following a cycloheximide block showed that depletion of *DUSP14* significantly extended the half-life of endogenous ACOX1 in HCT15 and RKO cells (Fig. 3e; Supplementary Fig. S4c).

Ubiquitin-mediated degradation is critical for protein stability^43,44^. Therefore, to explore whether DUSP14 promotes ACOX1 degradation via the ubiquitin-proteasome system, we transfected HEK293T cells with Myc-Ub, ACOX1, and DUSP14 wild type (WT) or DUSP14 mutant (DUSP14 Dead) with mutation of cysteine 111 to serine^45^, which damaged the phosphatase activity. Ubiquitination assays showed that DUSP14 WT (not DUSP14 Dead) overexpression markedly increased the polyubiquitination of ACOX1 (Fig. 3f), and DUSP14 promoted K48-linked ubiquitination of ACOX1, but not other position-linked ubiquitination of ACOX1 (Fig. 3g). To further explore the ubiquitination site(s) of ACOX1 mediated by DUSP14, we screened 6 candidate sites (K29, K241, K255, K260, K446, and K643) in the Phospho-Site Plus database and identified that DUSP14-mediated K48-linked ubiquitination of ACOX1 at K643, an evolutionally conserved residue among multiple species (Fig. 3h, i).

To determine the functional role of DUSP14 in CRC, we re-analyzed the public databases mentioned earlier and found that DUSP14 was highly expressed in CRCs (Supplementary Fig. S5a, b) and was positively correlated with advanced disease (Supplementary Fig. S5c–e). Further analysis showed that upregulation of *DUSP14* mRNA may be the result of *DUSP14* copy number amplification (Supplementary Fig. S5f, g). Meanwhile, *DUSP14* mRNA upregulation strongly correlated with poor overall survival in CRC patients (Supplementary Fig. S5h–j). Collectively, all of the findings suggest that DUSP14 is highly expressed in CRC, thus promoting ACOX1 degradation via the ubiquitin-proteasome system.

### Dephosphorylation of ACOX1 at S26 by DUSP14 is critical for CRC growth

**Fig. 4:**
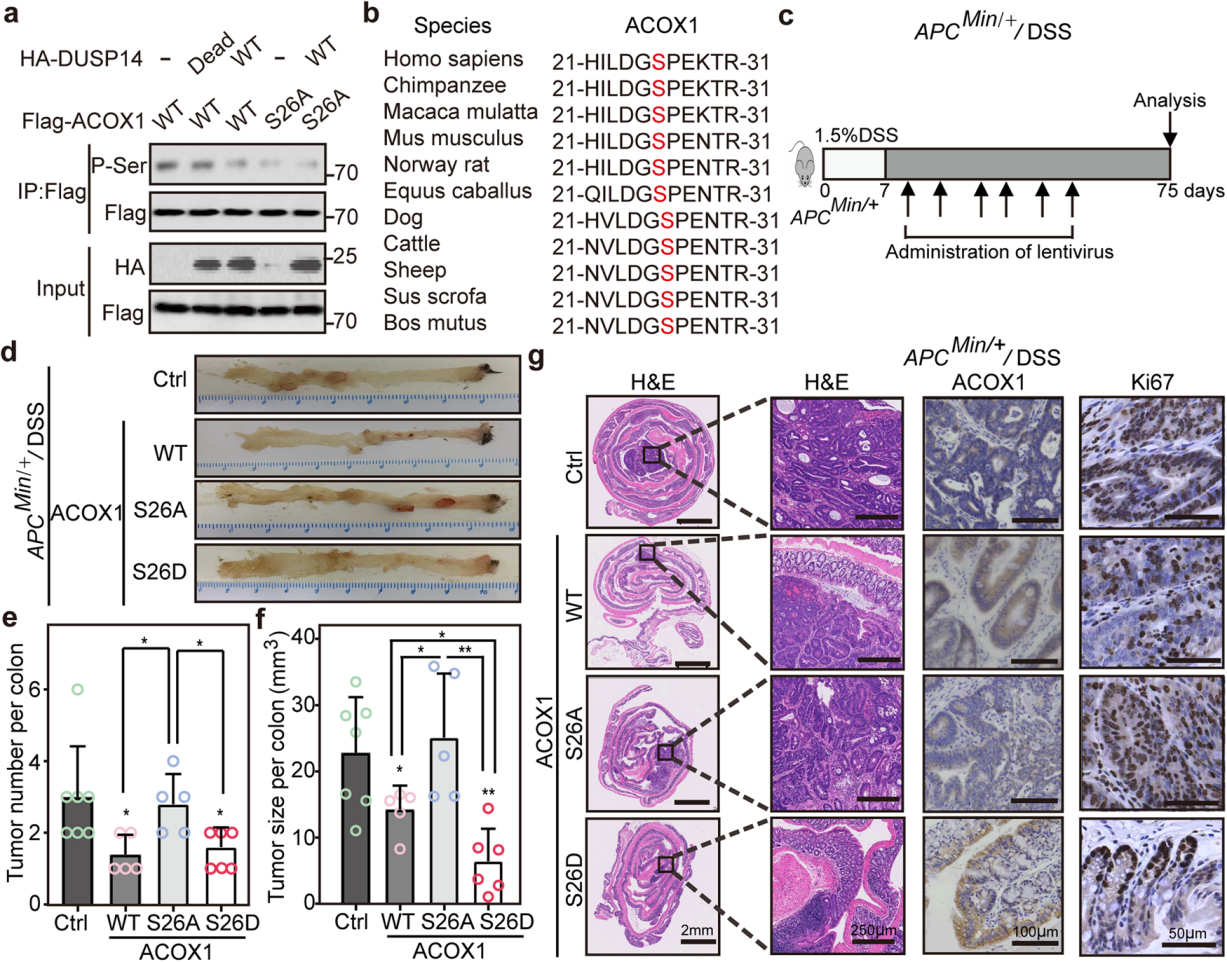
Dephosphorylation of ACOX1 by DUSP14 promotes CRC growth. **a** Dephosphorylation of ACOX1 S26 by DUSP14. Flag-ACOX1 (WT or S26A) was co-transfected with HA-DUSP14 (WT or Dead) into HEK293T cells, and the cell lysates were subjected to immunoprecipitation. **b** Alignment of serine 26 and adjacent amino acids of ACOX1 among multiple species. **c** Schematic diagram showing the experimental design for mouse CRC model. **d**–**g** Representative macroscopic morphologies (**d**), tumor numbers (**e**), tumor sizes (**f**) and H&E, Ki67 staining (**g**) of mice in control and ACOX1 (WT, S26A or S26D) groups. Data were analyzed using unpaired Student’s *t*-test (**e**, **f**). Data are presented as means ± SD; *P < 0.05, **P < 0.01; *n*, number of mouse samples.

Considering that DUSP14 is a multitarget phosphatase^45^ and DUSP14 regulates ACOX1 stability, we postulated that DUSP14 promotes ubiquitination and degradation of ACOX1 via dephosphorylation. To prove this, immunoprecipitation assays were performed, which revealed that DUSP14 specifically decreased ACOX1 serine phosphorylation rather than threonine phosphorylation and tyrosine phosphorylation (Supplementary Fig. S6a), implicating that DUSP14 dephosphorylates ACOX1 at serine residue(s). Next, we identified three serine phosphorylation sites (serine 26, serine 126, and serine 127) by MS analysis (Supplementary Fig. S6b). Further analysis showed that DUSP14 failed to promote the degradation and serine dephosphorylation of ACOX1 S26A mutant (Fig. 4a; Supplementary Fig. S6c). ACOX1 S26 is evolutionally conserved among vertebrates (Fig. 4b). Moreover, the phosphorylation-mimic mutant ACOX1 S26D exhibited an extended half-life and decreased ubiquitination levels, whereas the S26A mutant exhibited an opposite effect (Supplementary Fig. S6d, e). Additional structural analysis and glutaraldehyde cross-linking experiments revealed that DUSP14-mediated ACOX1 dephosphorylation did not affect the formation of ACOX1 homodimerization (Supplementary Fig. S6f, g). These studies indicated that dephosphorylation of ACOX1 at S26 by DUSP14 is a critical determinant of the ACOX1 protein stability.

Next, the lentiviruses encoding ACOX1 WT, ACOX1 S26A, or ACOX1 S26D were delivered intraperitoneally to DSS-treated *APC*^*Min/+*^ mice (Fig. 4c). Mice treated with ACOX1 WT or ACOX1 S26D exhibited fewer tumors, smaller tumors, and less histologic dysplasia (Fig. 4d–g; Supplementary Fig. S6h). In contrast, mice treated with ACOX1 S26A exhibited similar tumor burdens and pathological features as the controls (Fig. 4d–g; Supplementary Fig. S6h). These results show that DUSP14-mediated ACOX1 dephosphorylation is critical for CRC growth.

### *ACOX1* depletion stabilizes β-catenin and enhances its transcriptional activity via PA

**Fig. 5:**
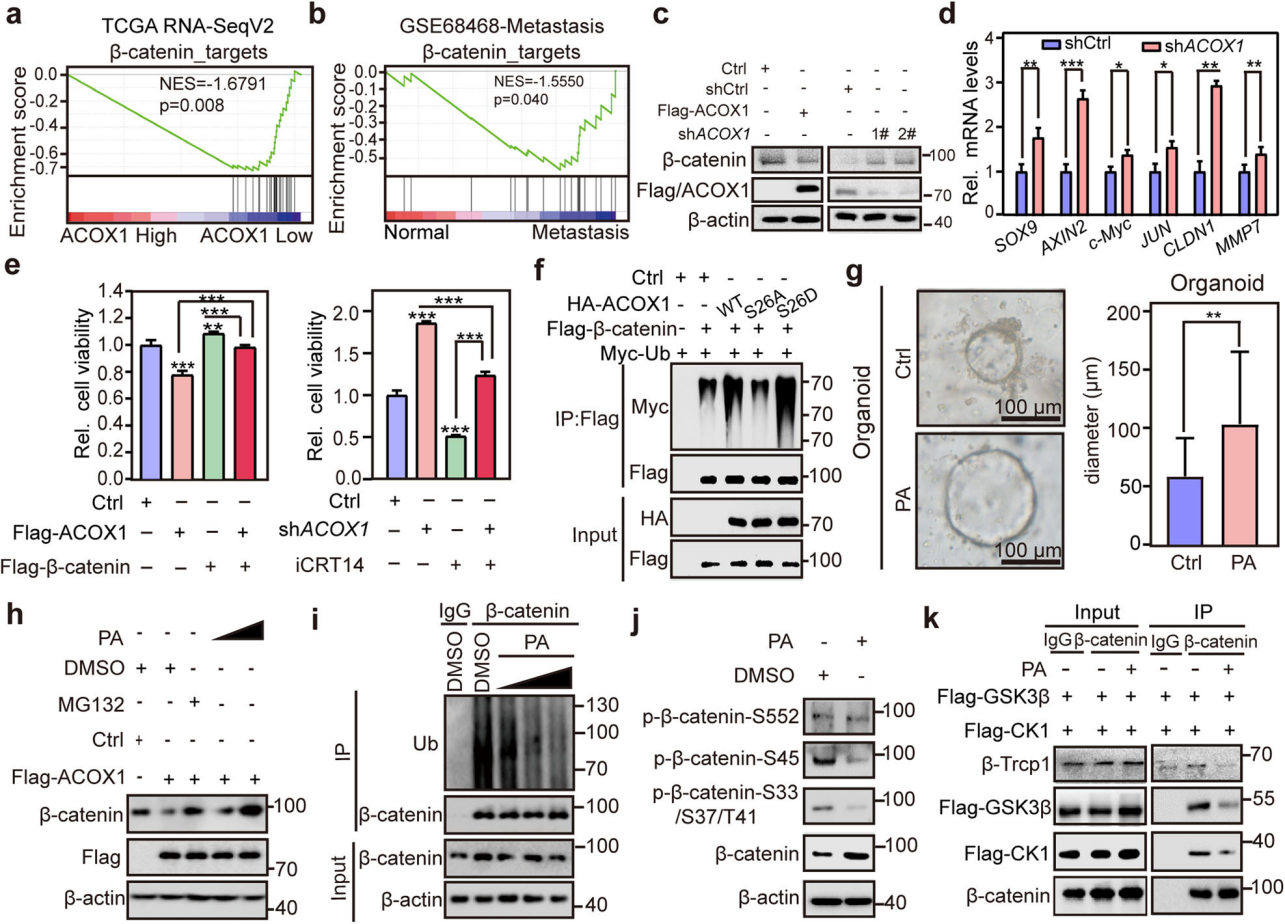
Depletion of *ACOX1* stabilizes β-catenin and enhances the transcriptional activity via PA. **a** Negative correlation between *ACOX1* and β-catenin target genes. GSEA of β-catenin target gene set in the expression profiles of TCGA RNA-SeqV2 according to the expression of *ACOX1*. **b** High expression of β-catenin target genes in metastatic tissues. GSEA of β-catenin target gene set in the expression profiles of normal tissues versus metastatic tissues from GSE68468. **c** Expression of β-catenin and ACOX1 analyzed by immunoblotting. *ACOX1*-depleted or ACOX1-overexpressed HCT15 cell lysates were subjected to immunoblotting. **d** Increased β-catenin target gene expression by *ACOX1* depletion. HCT15 cells stably expressing control shRNA or *ACOX1* shRNA (sh*ACOX1*) were analyzed by RT-qPCR. **e** ACOX1-induced inhibition of HCT15 cell viability rescued by β-catenin overexpression. HCT15 cells stably expressing Flag-ACOX1 or Flag-β-catenin were cultured for 5 days and counted by CCK-8 (left). Suppression of sh*ACOX1*-induced cell hyper-viability by β-catenin inhibition. HCT15 cells (shCtrl or sh*ACOX1* stably expressed) were treated with iCRT14 (100 μM) for 5 days and counted by CCK-8 (right). **f** Promoted β-catenin polyubiquitination by ACOX1 WT and S26D mutant but not S26A mutant. Myc-Ub was co-transfected with Flag-β-catenin and HA-ACOX1 (WT, S26A, or S26D) into HEK293T cells, and the cell lysates were subjected to immunoprecipitation. **g** Promoted human colonic organoid growth by PA treatment. Organoids were derived from human intestinal normal tissues, treated with PA (300 μM), and assessed by diameter size. Scale bars, 100 μm. **h** ACOX1-mediated β-catenin inhibition rescued by PA treatment. HCT15 cells transfected with Flag-ACOX1 plasmid were treated with MG132 (20 μM) for 6 h or PA (20 μM, or 100 μM) for 24 h as indicated and cell lysates were subjected to immunoblotting. **i** Decreased endogenous β-catenin polyubiquitination by PA treatment. HEK293T cells were treated with PA (50 μM, 100 μM, or 200 μM) for 24 h, and the cell lysates were subjected to immunoprecipitation. **j** Expression of β-catenin phosphorylation. HEK293T cells were treated with PA (100 μM) for 24 h and the cell lysates were subjected to immunoblotting. **k** Decreased interactions between β-catenin and CK1/GSK3β/β-Trcp by PA treatment. HEK293T cells transfected with Flag-CK1 and Flag-GSK3β were treated with MG132 (20 μM) for 6 h or PA (100 μM) for 24 h and cell lysates were subjected to Co-IP. Data were analyzed using unpaired Student’s *t*-test (**d**, **e**, **g**). Data are presented as means ± SD; *P < 0.05, **P < 0.01, ***P < 0.001.

To explore the CRC-related cellular signaling regulated by ACOX1, gene set enrichment analysis (GSEA) was performed in the TCGA database. GSEA revealed that ACOX1 negatively correlated with Wnt signaling, but not other cancer-related signaling pathways (Supplementary Fig. S7a). Meanwhile, β-catenin target genes were upregulated in tumor tissues and metastasis samples with lower levels of *ACOX1* in public databases (Fig. 5a, b; Supplementary Fig. S7b, c). Interestingly, *ACOX1* depletion did not affect *CTNNB1* (encoding β-catenin protein) mRNA levels but increased β-catenin abundance in HCT15 and RKO cells (Fig. 5c; Supplementary Fig. S7d, e). Depletion of *ACOX1* markedly increased β-catenin target gene expression in HCT15 and RKO cells (Fig. 5d; Supplementary Fig. S7f). To further validate that ACOX1 inhibits CRC cell growth by impairing β-catenin-mediated target gene transcription, CCK-8 assays were performed in HCT15 and RKO cells. As expected, overexpression of ACOX1 inhibited CRC cell viability, which was rescued by β-catenin overexpression (Fig. 5e; Supplementary Fig. S7g). Conversely, *ACOX1* depletion increased CRC cell viability, which was inhibited by iCRT14, a β-catenin transcriptional activity inhibitor that disrupts the binding of β-catenin to TCF (Fig. 5e; Supplementary Fig. S7g).

β-catenin is prominently degraded in a ubiquitin-proteasome manner^46^. Thus, we speculated whether ACOX1 reduces the stability of β-catenin via promoting its polyubiquitination. As expected, both ACOX1 WT and ACOX S26D increased β-catenin polyubiquitination levels (Fig. 5f), whereas ACOX1 S26A failed to do so (Fig. 5f), supporting that DUSP14-mediated ACOX1 dephosphorylation is critical for colorectal tumorigenesis.

ACOX1 is a rate-limiting enzyme in peroxisomal fatty acid β-oxidation^6^. PA, a substrate of ACOX1^7^, has been reported to promote tumor cell growth and migration^18–20^. Hence, we speculated that ACOX1 regulates β-catenin stability via PA. Overexpression of ACOX1 significantly decreased PA levels in both HCT15 and RKO cells (Supplementary Fig. S7h). Furthermore, PA markedly promoted human colonic organoid growth (Fig. 5g). Overexpression of ACOX1 decreased β-catenin abundance, which was rescued by PA treatment (Fig. 5h). In addition, proteomic analysis showed that PA promotes expression of β-catenin and its target genes in HCT15 cells (Supplementary Fig. S7i, j). These results suggest that *ACOX1* depletion stabilizes β-catenin via PA.

To determine whether PA regulated ubiquitination of β-catenin, we performed ubiquitination assays in HEK293T cells, and the results suggested that PA decreased endogenous β-catenin polyubiquitination in a dose-dependent manner (Fig. 5i). Previous studies have demonstrated that phosphorylation of β-catenin at Ser45 by CK1 could trigger sequential phosphorylation of Thr41, Ser37, and Ser33 by GSK3 (preferentially by GSK3β)^47,48^, leading to the recognization of phosphorylated β-catenin by E3 ubiquitin ligase β-TrCP and subsequent degradation by the ubiquitin-proteasome system^49^. In addition, AKT phosphorylates β-catenin at Ser552 to promote β-catenin accumulation in both the cytosol and the nucleus and thus enhances its transcriptional activity^50^. To explore the mechanisms of β-catenin stabilization regulated by PA, immunoblotting analysis was performed, which revealed that PA significantly decreased β-catenin Ser33, Ser37, Thr41, and Ser45 phosphorylation. However, it failed to affect the Ser552 phosphorylation of β-catenin (Fig. 5j; Supplementary Fig. S7k), suggesting that PA inhibits CK1- and GSK3-mediated β-catenin phosphorylation. Co-IP assays revealed that PA suppressed the interactions between β-catenin and CK1, GSK3, and β-TrCP (Fig. 5k). Together, these results suggest that ACOX1 depletion stabilizes β-catenin and enhances its transcriptional activity via PA.

### PA-mediated β-catenin palmitoylation inhibits the ubiquitination of β-catenin

**Fig. 6:**
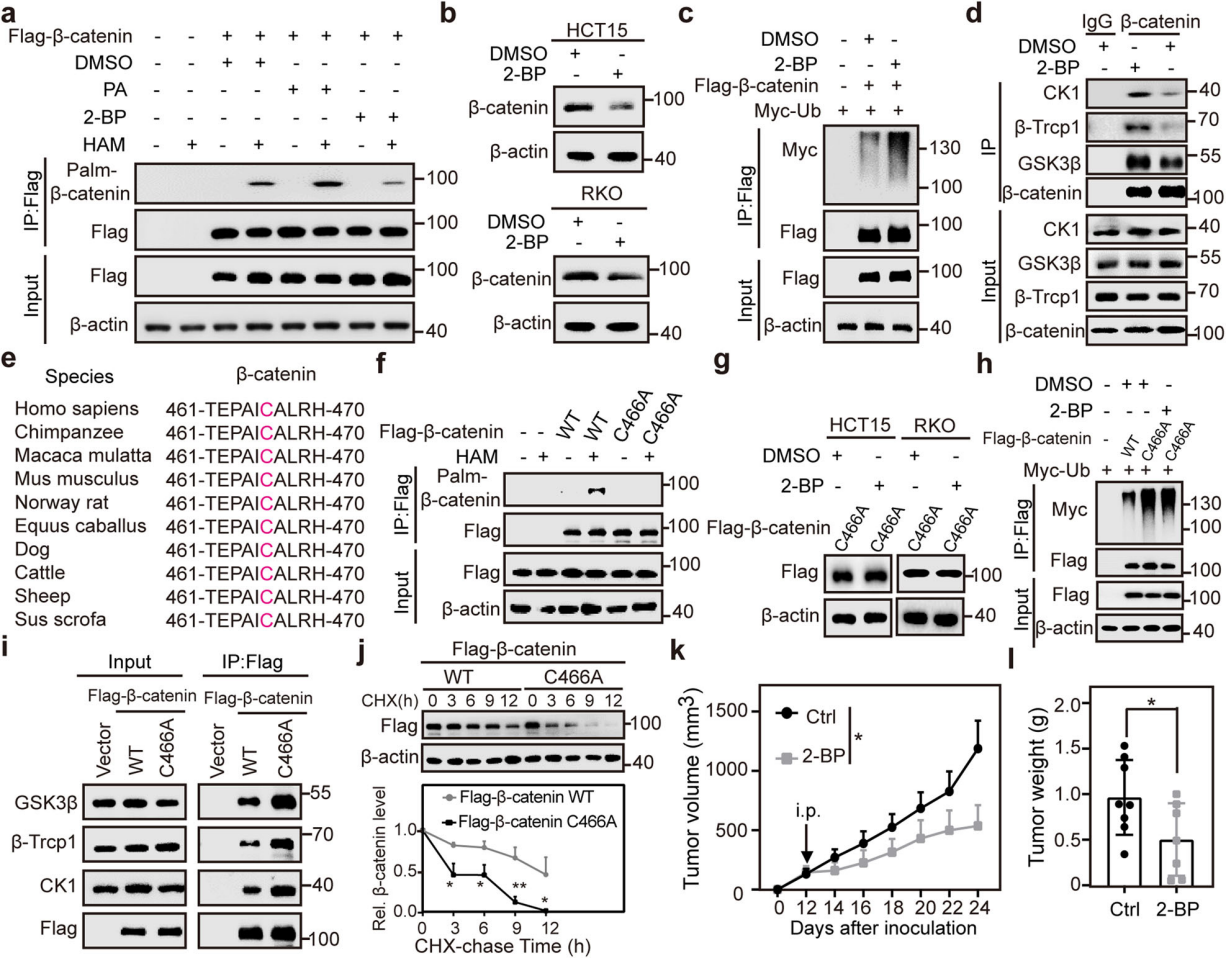
PA-mediated β-catenin palmitoylation inhibits the ubiquitination of β-catenin. **a** Increased and decreased β-catenin palmitoylation by PA and 2-BP, respectively. HEK293T cells transfected with Flag-β-catenin were treated with 2-BP (100 μM) for 6 h or PA (100 μM) for 24 h and cell lysates were subjected to immunoprecipitation. Palmitoylated β-catenin was detected with HRP-conjugated streptavidin antibody. **b** Decreased β-catenin abundance by 2-BP treatment. HCT15 and RKO cells were treated with 2-BP (100 μM) for 6 h and cell lysates were subjected to immunoblotting. **c** Increased β-catenin polyubiquitination by 2-BP treatment. HEK293T cells transfected with Flag-β-catenin and/or Myc-Ub were treated with 2-BP (100 μM) or DMSO for 6 h and cell lysates were subjected to immunoprecipitation. **d** Interactions of β-catenin and CK1/GSK3/β-TrCP were enhanced by 2-BP treatment. HEK293T cells were treated with 2-BP (100 μM) or DMSO for 6 h and cell lysates were subjected to immunoprecipitation. **e** Alignment of cysteine 466 and adjacent amino acids of β-catenin among multiple species. **f** Unalterated β-catenin C466A protein abundance by 2-BP treatment. HCT15 and RKO cells transfected with Flag-β-catenin C466A were treated with 2-BP (100 μM) for 6 h, and cell lysates were subjected to immunoblotting. **g** Abolished β-catenin palmitoylation by β-catenin C466A mutation. HEK293T cells were transfected with Flag-β-catenin or Flag-β-catenin C466A and cell lysates were subjected to immunoprecipitation. **h** Increased interactions of β-catenin and CK1/GSK3/β-TrCP by β-catenin C466A mutant. HEK293T cells were transfected with Flag-β-catenin or Flag-β-catenin C466A, and cell lysates were analyzed by Co-IP. **i** Unalterated β-catenin C466A poly-ubiquitination by 2-BP treatment. HEK293T cells transfected with Flag-β-catenin or Flag-β-catenin C466A were treated with 2-BP (100 μM) or DMSO for 6 h and cell lysates were subjected to immunoprecipitation. **j** Decreased β-catenin half-life by C466A mutation. Time-course analysis of β-catenin levels in Flag-β-catenin WT-, or C466A-overexpressed HEK293T cells (upper). β-catenin quantified by densitometry, with β-actin as a normalizer (lower). **k** Nude mice carrying HCT15 tumors were intraperitoneally injected with 2-BP, and tumor volume was evaluated. **l** The tumor weight in the subcutaneous xenograft model. Data were analyzed using unpaired Student’s *t*-test (**j**, **k**, **l**). Data are presented as means ± SD; *P < 0.05, **P < 0.01.

Previous studies demonstrated that PA is the substrate of protein palmitoylation^22–24^, and that protein palmitoylation can alter the protein–protein interaction^24^. Therefore, we hypothesized that β-catenin is palmitoylated, which subsequently inhibits its interactions with CK1/GSK3/β-TrCP. As expected, palmitoylation of endogenous β-catenin was confirmed in HEK293T cells (Supplementary Fig. S7l). Interestingly, endogenous palmitoylated β-catenin accounted for 31.7% and 28.4% of total β-catenin in HCT15 and RKO cells, respectively (Supplementary Fig. S7m). Importantly, palmitoylation of β-catenin was increased by PA but decreased by 2-bromopalmitate (2-BP), a palmitoylation inhibitor (Fig. 6a). Inhibition of palmitoylation by 2-BP also decreased β-catenin abundance (Fig. 6b), and promoted β-catenin polyubiquitination (Fig. 6c). These data reveal that β-catenin stabilization can be regulated through a novel palmitoylation modification.

To clarify whether β-catenin palmitoylation affected the interactions between β-catenin and CK1/GSK3/β-TrCP, we performed Co-IP assays and found that 2-BP increased the β-catenin–CK1/GSK3/β-TrCP interactions (Fig. 6d). Next, we used the predictor Swiss-Palm^51^ and identified cysteine 466 as a candidate conservative palmitoylation site in β-catenin across species (Fig. 6e). Mutation of cysteine 466 to alanine substantially abolished β-catenin palmitoylation (Fig. 6f; Supplementary Fig. S7n), suggesting that this cysteine residue is the major palmitoylation site of β-catenin. In addition, this mutation rendered resistance to 2-BP-mediated β-catenin downregulation, promoted β-catenin–CK1/GSK3/β-TrCP interactions, increased β-catenin polyubiquitination, and shortened the half-life of β-catenin (Fig. 6g–j).

To investigate the effect of cysteine 466 palmitoylation of β-catenin on CRC cell growth, we constructed a series of stable cell lines for CCK-8 and colony formation assays. The results revealed that depletion of β-catenin inhibited CRC cell colony formation and proliferation, which were substantially rescued by the re-expression of β-catenin WT, but not its C466A mutant (Supplementary Fig. S7o–q). To further pharmacologically inhibit β-catenin palmitoylation in vivo, 2-BP was tested in the subcutaneous xenograft model. Intraperitoneal injection of 2-BP (40 mg/kg; one injection per day) in nude mice carrying HCT15 tumors inhibited tumor growth (Fig. 6k, l; Supplementary Fig. S7r). Consistently, 2-BP injection significantly decreased β-catenin palmitoylation and protein abundance in tumor tissues (Supplementary Fig. S7s). Together, these results demonstrate that PA-mediated β-catenin palmitoylation is essential for inhibiting the β-catenin–CK1/GSK3/β-TrCP interactions, thereby enhancing β-catenin stability, and that targeting β-catenin palmitoylation by 2-BP can efficiently suppress tumor growth.

### β-catenin directly suppresses *ACOX1* transcription and indirectly activates *DUSP14* transcription via c-Myc

**Fig. 7:**
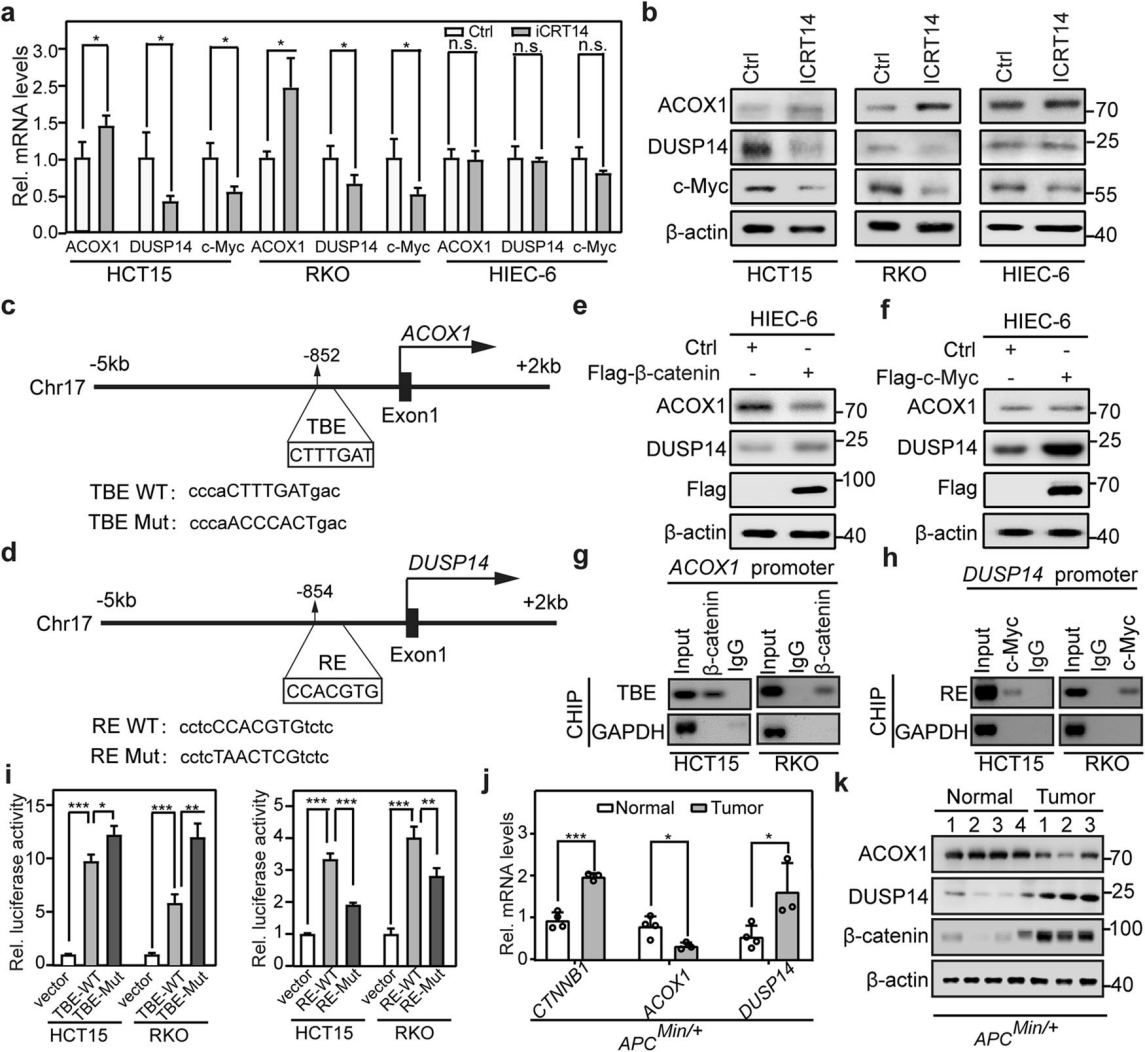
β-catenin directly represses *ACOX1* transcription and indirectly activates *DUSP14* transcription via c-Myc. **a**, **b** Upregulated ACOX1 expression and downregulated DUSP14 and c-Myc expression by β-catenin inhibition in CRC cells, but not in HIEC-6 cells. Indicated cells were treated with iCRT14 (100 μM) for 24 h and analyzed by RT-qPCR (**a**) and immunoblotting (**b**). **c**, **d** Schematic presentation of TCF/LEFs-binding element on the *ACOX1* locus (**c**) and c-Myc-binding sites on the *DUSP14* locus (**d**). TBE, TCF/LEFs-binding element; RE, c-Myc-responsive element. Consensus sequence mutations are shown as TBE Mut and RE Mut. **e** Downregulated ACOX1 expression and upregulated DUSP14 expression by β-catenin overexpression. **f** Upregulated DUSP14 expression by c-Myc overexpression. HIEC-6 cells transfected with Flag-β-catenin (**e**) or Flag-c-Myc (**f**) were subjected to western blot analysis. **g**, **h** β-catenin occupancy on the *ACOX1* promoter (**g**) and c-Myc occupancy on the *DUSP14* promoter (**h**). HCT15 and RKO cells were analyzed by ChIP assays. **i** Assessed Luciferase reporter activities in the presence of exogenous β-catenin (left) and c-Myc (right) in HCT15 and RKO cells. **j**, **k** Downregulation of ACOX1 and upregulation of DUSP14 in *APC*^*Min/+*^ intestinal tumors. ACOX1 and DUSP14 expression was analyzed in normal small intestinal tissues and intestinal adenoma samples from *APC*^*Min/+*^ mouse (21 weeks of age) by RT-qPCR (**j**) and western blot analysis (**k**). Data were analyzed using unpaired Student’s *t*-test (**a**, **i**, **j**). Data are presented as means ± SD; *P < 0.05, **P < 0.01, ***P < 0.001; *n*, number of mouse samples.

To explore the cause of *ACOX1* mRNA downregulation, we analyzed *ACOX1* copy number; however, there was no difference between normal tissue and tumor samples (Supplementary Fig. S8a), suggesting that *ACOX1* copy number alteration is not the cause of *ACOX1* mRNA downregulation. PPARA has been considered to be the main transcription factor of ACOX1^52,53^; however, normal tissues and tumor samples from the GEO datasets showed no difference in their expressions of *PPARA* mRNA (Supplementary Fig. S8b). Given the correlation between ACOX1 and β-catenin targets in CRC (Fig. 5a; Supplementary Fig. S7b, c), we assessed whether β-catenin regulates ACOX1 in CRC. Interestingly, iCRT14-treated CRC cells exhibited upregulation of *ACOX1* transcripts and protein, but downregulation of *DUSP14* transcripts and protein (Fig. 7a, b). However, iCRT14 did not appear to affect their transcripts or protein expression in normal human intestinal epithelial cells HIEC-6 (Fig. 7a, b), indicating that background-level β-catenin does not affect *ACOX1* and *DUSP14* transcription.

Motifmap website analysis identified the potential TCF/LEFs-binding elements (TBE; CTTTGA/TA/T) in the *ACOX1* promoter regions (–5 to +2 kb) (Fig. 7c) and the putative c-Myc E-box response elements (RE; CCACGTG) in the *DUSP14* promoter regions (–5 to +2 kb) (Fig. 7d). Ectopic expression of β-catenin downregulated *ACOX1* (Fig. 7e) and c-Myc ectopic expression upregulated *DUSP14* (Fig. 7f). Chromatin immunoprecipitation (ChIP) assays revealed that β-catenin occupied the promoter of *ACOX1* while c-Myc bound the promoter of *DUSP14* in HCT15 and RKO cells (Fig. 7g, h). Luciferase reporter assays confirmed that β-catenin significantly suppressed TBE, and that c-Myc activated RE, as compared to TBE or RE mutant (TBE or RE Mut) in HCT15 and RKO cells (Fig. 7i).

Next, we examined whether β-catenin regulates ACOX1 and DUSP14 in vivo. Both *ACOX1* mRNA and protein levels were significantly decreased, whereas the mRNA and protein levels of *DUSP14* were highly increased in intestinal adenomas of *APC*^*Min/+*^ mice (Fig. 7j, k). These results support the notion that β-catenin directly or indirectly regulates the *ACOX1* and *DUSP14* transcription in CRC, thus constituting a reciprocal regulation among β-catenin, ACOX1, and DUSP14.

### The DUSP14-ACOX1-PA-β-catenin axis is dysregulated in human CRC

**Fig. 8:**
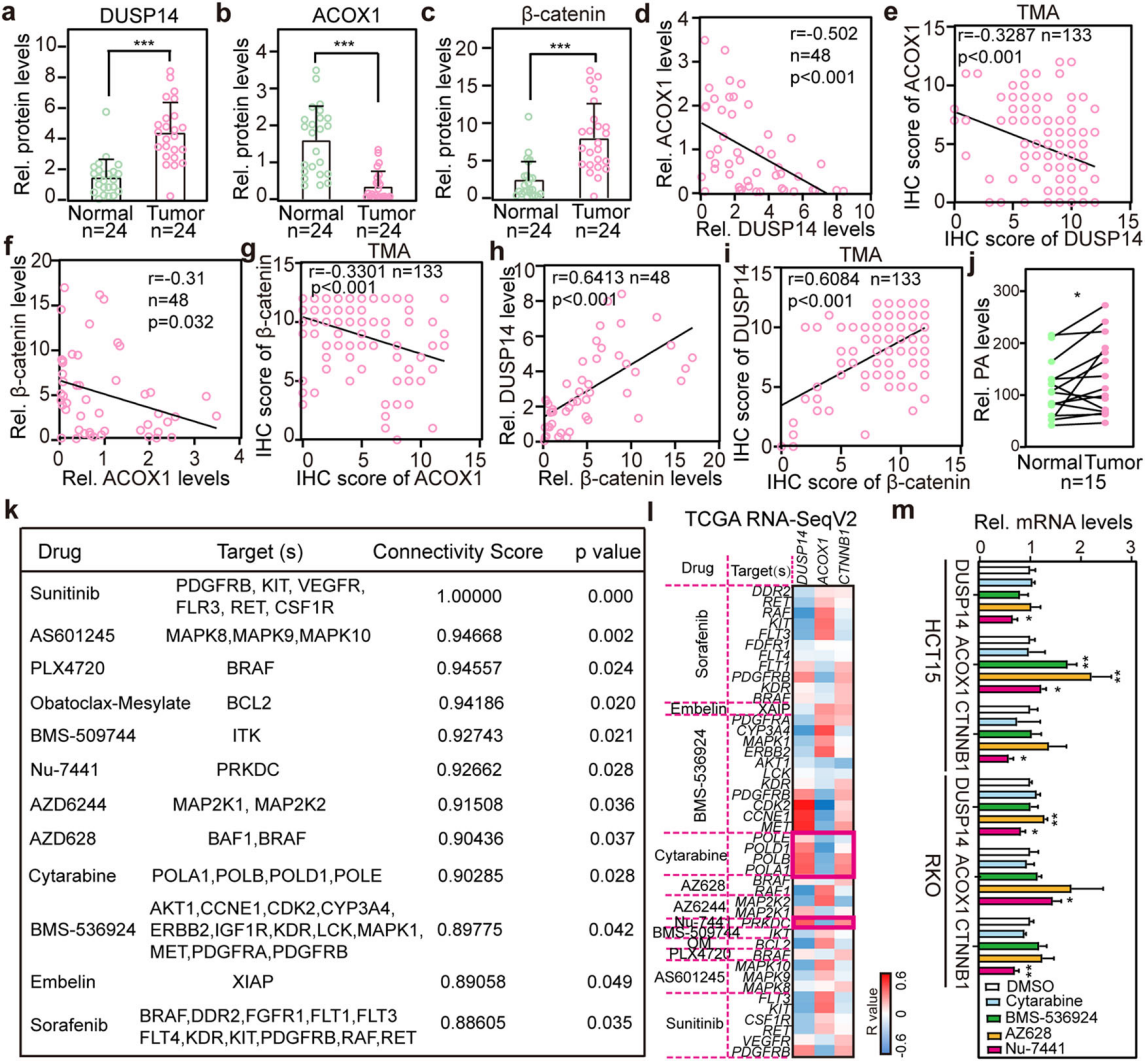
Dysregulation of the DUSP14-ACOX1-PA-β-catenin axis in human CRC. **a**–**c** Relative protein levels of DUSP14 (**a**), ACOX1 (**b**) and β-catenin (**c**). The proteins were quantified by densitometry, with β-actin as a normalizer, as shown in Supplementary Fig. S9d. **d**, **e** Pearson correlation analysis of DUSP14 and ACOX1 proteins from human CRCs (**d**) and CRC TMA (**e**). **f**, **g** Pearson correlation analysis of β-catenin and ACOX1 proteins from human CRCs (**f**) and CRC TMA (**g**). **h**, **i** Pearson correlation analysis of DUSP14 and β-catenin proteins from human CRCs (**h**) and CRC TMA (**i**). **j** Analysis of PA levels in adjacent normal tissues versus matched primary tumor tissues. **k** Predicted compounds and target genes for inhibiting DUSP14-ACOX1-β-catenin axis from DeSigN. **l** Drug sensitivity analysis for inhibiting DUSP14-ACOX1-β-catenin axis. Correlations of drug target genes and *DUSP14*, *ACOX1*, and *β-catenin* were shown by the heatmap, based on TCGA RNA-SeqV2. The red rectangular frame indicates high drug sensitivity. **m** Nu-7441 effectively inhibits the DUSP14-ACOX1-β-catenin axis in HCT15 and RKO cells. HCT15 and RKO cells were treated with DMSO, Cytarabine (10 nM), AZ628 (10 nM), BMS-536924 (5 nM) or Nu-7441 (20 nM) for 24 h, and cells were subjected to RT-qPCR. Data were analyzed using unpaired Student’s *t*-test (**a**–**c**, **m**) or paired Student’s *t*-test (**j**). Data are presented as means ± SD; *P < 0.05, **P < 0.01, ***P < 0.001; *n*, number of patient samples.

To illustrate the correlation among DUSP14, ACOX1, and β-catenin in CRC, we used IHC of matched patient samples from the HPA dataset (Supplementary Fig. S9a) and validated the negative correlation between ACOX1 and DUSP14 and the negative correlation between ACOX1 and β-catenin (Supplementary Fig. S9b, c). Interestingly, we found that the DUSP14-ACOX1-β-catenin axis is dysregulated in early-stage CRC (Supplementary Fig. S9d). To better validate this observation, 24 early-stage CRC samples (T) with adjacent normal colon tissues (N) were collected (Supplementary Fig. S9e). We observed that ACOX1 protein was significantly decreased in these CRC samples, whereas DUSP14 and β-catenin were markedly increased (Fig. 8a–c). Moreover, the negative correlations between DUSP14 and ACOX1, and ACOX1 and β-catenin, and the positive correlation between β-catenin and DUSP14 were also confirmed in our samples (Fig. 8d, f, h). These results were further validated in our CRC TMA (Fig. 8e, g, i; Supplementary Fig. S9f). More importantly, PA levels were higher in tumor samples than those in paired normal samples (Fig. 8j).

To explore whether there are known small molecules or drugs that inhibit the DUSP14-ACOX1-β-catenin axis, the DeSigN^54^ database (Fig. 8k) and TCGA dataset were used, and they showed that Nu-7441 and Cytarabine inhibitors may inhibit the DUSP14-ACOX1-β-catenin axis (Fig. 8l). Only Nu-7441 could effectively inhibit this signal axis in HCT15 and RKO cells (Fig. 8m). Moreover, Nu-7441 significantly inhibited the proliferation of HCT15 and RKO cells (Supplementary Fig. S9g). Taken together, these data support the idea that the DUSP14-ACOX1-PA-β-catenin axis plays a crucial role in CRC progression and that Nu-7441 may be a potential strategy for the treatment of CRC by inhibiting the DUSP14-ACOX1-PA-β-catenin axis.

## Discussion

In this study, we demonstrated a crucial role of dephosphorylation in regulating the stability of ACOX1 protein, which reveals the crosstalk of dephosphorylation and ubiquitination of ACOX1. Accumulation of PA induced by enhanced ACOX1 dephosphorylation promotes palmitoylation of β-catenin, providing an additional layer of regulation to enhance β-catenin signaling in cancer. These findings establish a link between cancer metabolism and the β-catenin signaling and reveal modulation of these post-translational modifications as a promising therapeutic strategy against cancer.

Metabolic reprogramming affects tumorigenesis and tumor progression by maintaining deregulated proliferation and preserving a dedifferentiated state^1^. Previous studies have shown that dysregulation of metabolic enzyme ME1 phosphorylation and acetylation promotes lipid metabolism and colorectal tumorigenesis^55^, and that hyperactivation of metabolic enzyme PKM2 methylation promotes aerobic glycolysis and tumorigenesis^56^. Metabolic enzyme ACOX1, a rate-limiting enzyme in peroxisomal fatty acid β-oxidation^6–8^, is expressed in multiple tissues. Accumulating evidence has revealed the aberrantly low expression of ACOX1 in many cancers such as lymphoma^57^, oral squamous cell carcinoma^13^, and bladder cancer^58^. Herein, through systematic bioinformatics screening and a series of molecular and cellular experiments, we revealed that reprogramming of PA induced by dephosphorylation of ACOX1 is critical for CRC progression. These findings extend our understanding of metabolic reprogramming induced by post-translational modifications of important metabolic enzymes in cancer.

Emerging evidence suggests that metabolites can regulate the epigenetic modification of proteins^59,60^. For example, acetyl-CoA derived from hepatic fatty acid oxidation promotes Raptor acetylation^59^, and S-adenosylmethionine provides methyl donor for protein methylation^60^. PA can modify protein palmitoylation^22–24^, which is known to regulate protein functions^24–27^. In this study, accumulated PA caused by the dephosphorylation of ACOX1 by DUSP14 modifies β-catenin C466 palmitoylation, and palmitoylation of β-catenin suppresses the phosphorylation of β-catenin by GSK3 and CK1, thereby preventing β-Trcp-mediated β-catenin trafficking to the proteasome, increasing the protein level and transcriptional activity of β-catenin (Supplementary Fig. S10). In summary, we discovered a novel β-catenin modification, palmitoylation, and the mechanism by which palmitoylation regulates β-catenin stability, which complements our knowledge of canonical β-catenin signaling.

In addition to being a potent DNA-PK inhibitor, Nu-7441 has also been shown to inhibit PI3K, mTOR, and non-homologous end joining pathway^61–63^, suggesting robust anti-cancer ability. Here, our data reveal that Nu-7441 significantly inhibits CRC cell growth by targeting the DUSP14-ACOX1-β-catenin axis. This finding shows that Nu-7441 may be a potential drug for the treatment of CRC.

Although popular models suggest that β-catenin phosphorylation/ubiquitination should be inhibited by *APC* mutations, it has been documented that different *APC* mutation types have different degradation efficiencies for β-catenin, which contributes to different levels of tumor progression^64–66^. Therefore, we believe that the DUSP14-ACOX1-β-catenin axis is still suitable for some CRC cell lines with *APC* mutations (such as HCT15 cell line). In addition, TCF/β-catenin not only stimulates gene transcription but can also repress it^67,68^. This is possibly mediated by directly recruiting repressive factors, such as Reptin or Fhit that associate with the TCF/β-catenin complex and thus repress β-catenin-mediated transcription^69,70^, which may explain the mechanism behind the repressive effect of the TCF/β-catenin complex on *ACOX1* expression in this study. Furthermore, the E3 ubiquitin ligase and protein kinase of ACOX1, as well as the palmitoyl transferase and de-palmitoyl transferase of β-catenin have not yet been discovered. Another limitation of this study is that specific antibody recognizing phosphorylated S26 of ACOX1 has not yet been developed. We will conduct follow-up research to address the above concerns in the future. In addition, given that fatty acid β-oxidation in peroxisome and mitochondria share some common substrates (mainly some long-chain fatty acids)^8,71^, we speculate that mitochondria-mediated PA β-oxidation may also affect CRC progression.

The results of our study have revealed that ACOX1 is a tumor suppressor and critical for the supervision of β-catenin signaling by regulating PA-mediated β-catenin palmitoylation and stabilization. We have also proposed that inhibition of the dephosphorylation of ACOX1 by DUSP14 or β-catenin palmitoylation may be a viable option for CRC treatment.

## Material and methods

### Cell culture and transfection

Human HCT15, RKO, HCT8, SW620, HCT116, HIEC-6, and HEK293T cells were obtained from the American Type Culture Collection (ATCC). Cells were cultured in DMEM medium (Gibco, NY, USA) supplemented with 10% fetal bovine serum (Gibco, NY, USA) and 1% penicillin-streptomycin (Gibco, CA, USA) at 37 °C in a 5% CO_2_ incubator. For transfection, after growing to 70% confluence, cells were transfected using Lipofectamine 3000 (Invitrogen, Carlsbad, CA) or HighGene (ABclonal, Wuhan, China), according to the manufacturer’s instructions.

### Reagents and plasmids

Proteasome inhibitor MG132 (HY-13259), Nutlin-3 (HY-50696), Cytarabine (HY-13605), BMS-536924 (HY-10262), AZ628 (HY-11004), and palmitic acid (HY-N0830) were purchased from MedChemExpress. Cycloheximide (R750107), Hydroxylamine (HAM, 467804), 2-BP (238422), and DAPI (D9542) were purchased from Sigma-Aldrich. iCRT14 (sc-362746) was purchased from Santa Cruz. A dual-luciferase reporter assay kit (DL-101-01) was purchased from Vazyme. *N*-Ethylmaleimide (NEM, A600450-0005) and BMCC-biotin ((1-Biotinamido)-4-[4′-(maleimidomethyl)cyclohexanecarboxamido]hexane, C100222-0050) were purchased from Sangon Biotech. Human palmitic acid ELISA kits (MM-51627H2) were purchased from MeiMian (Jiangsu, China). Anti-Flag agarose beads (23101) and Nu-7441 (503468-95-9) were purchased from Selleck (Houston, USA). RNase A (CW2105) was purchased from CWBIO. All antibodies used in this study are indicated in Supplementary Table S4. The human *DUSP14*, *ACOX1*, *CTNNB1*, and *c-Myc* coding sequences were amplified from HEK293T cDNA and cloned into pCMV-HA and pHAGE-CMV-MCS-PGK vectors. The human *GSK3β* and *CK1* coding sequences were amplified from HEK293T cDNA and cloned into the pHAGE-CMV-MCS-PGK vector. The human *ACOX1*-TBE and *DUSP14*-RE were amplified from HCT15 gDNA and cloned into the pGL3-basic luciferase vector. The mouse *ACOX1* coding sequence was amplified from mouse colon cDNA and cloned into pCDH-CMV-MCS-EF1-GFP+Puro vector. Mutations in the *DUSP14*, *ACOX1*, *β-catenin*, and *Ubiquitin* cDNAs were generated by overlap extension PCR. Deletion mutants from *DUSP14* and *ACOX1* were cloned into the pHAGE-CMV-MCS-PGK vector. Human *DUSP14*, *CTNNB1*, *ACOX1*, and mouse *Acox1* shRNAs were designed and synthesized by RuiBiotech (Guangzhou, China), subsequently annealed, and inserted into the pLKO.1-puro vector. All primers for construction are presented in Supplementary Table S5.

### Animal studies

All animal studies were approved by the Animal Care Committee of Sun Yat-sen University. All mice were maintained in micro isolator cages in the Experimental Animal Center of Sun Yat-sen University.

AOM/DSS-induced mouse CRC model was performed following previously described methods^38^. Briefly, eight-week-old C57BL/6 mice were injected intraperitoneally with 10 mg/kg AOM (Sigma-Aldrich). After 7 days, the mice were given drinking water containing 2.5% DSS (MP Biomedicals, Santa Ana, CA, USA) for a week, followed by regular drinking water for 2 weeks. Then, the mice were fed with 2.5% DSS water for two rounds for 1 week and sacrificed on the 120th day.

*APC*^*Min/+*^/DSS-induced mouse CRC model was performed following the previously described methods^39^. Briefly, the eight-week-old *APC*^*Min/+*^ mice were fed with 1.5% DSS water for 1 week and sacrificed on the 75th day.

Lentivirus production was performed following previously described methods^72^. Briefly, lentivirus was produced using polyethyleneimine-mediated transfection of a second-generation packaging system in HEK293T cells. Supernatant containing lentivirus was harvested at 72 h after transfection and filtered using a 0.45-μm filter. The lentivirus containing supernatant was then mixed with concentrate solution (5% PEG8000 and 0.5 M NaCl) overnight and concentrated by centrifugation at 4 °C. Virus titers were determined by ELISA kit.

For administration of lentivirus to *APC*^*Min/+*^/DSS-induced or AOM/DSS-induced CRC mice, the eight-week-old mice were randomly assigned to indicated groups. The control group of mice was treated with lentivirus-expressing Ctrl or shCtrl and the indicated group(s) was/were treated with lentivirus-expressing indicated protein or shRNA. Concentrated lentivirus was delivered intraperitoneally to indicated mice twice per week for 3 weeks^55^. On day 75 or 120, the indicated mice were sacrificed and their tumor burdens were evaluated. Tumors larger than 1 mm were counted and measured. Colon tissues were collected for RNA extraction, protein assays, and pathological studies.

For PDX transplantation, patients-derived tumor tissues were subcutaneously transplanted into BALB/c nude mice. When the tumors reached a certain size, the subcutaneous tumor was dissected and decomposed into a tumor of ~1 mm, and then inoculated into the subcutaneous of BALB/c nude mice again. One week after transplantation, mice were randomly assigned to 2 groups and injected with lentivirus expressing Ctrl or Flag-ACOX1 every 2 days for 9 total times. At day 21, PDXs were collected for tumor volume measurement and IHC analysis. Tumor volumes were calculated by the equation V (mm^3^) = a × b^2^/2, where a is the length and b is the width.

For subcutaneous xenograft model, the experiment was performed following previously described methods^43^. Briefly, four-week-old female BALB/c nude mice were purchased from GemPharmatech (Guangzhou, China), and 5 × 10^6^ HCT15 cells were suspended in 100 μL PBS and injected subcutaneously in the flanks of animals. Twelve days after transplantation, 2-BP (40 mg/kg) was delivered intraperitoneally to indicated nude mice once per day for 12 days. Tumor growth was monitored every two days for a total period of 24 days. Tumor volumes were calculated by the equation V (mm^3^) = a × b^2^/2, where a is the length and b is the width.

### Human CRC specimens

Forty-five early CRC samples and 51 normal adjacent tissues were used to analyze *ACOX1* transcript levels, and 24 pairs of these samples were used to analyze ACOX1, DUSP14, and β-catenin protein levels. Fifteen fresh CRC samples and matched normal adjacent tissues were used to analyze PA levels. 192 CRC samples were made into TMA to analyze indicated protein levels and overall survival. All samples were obtained from the Sixth Affiliated Hospital of Sun Yat-sen University. The diagnosis of CRCs was verified by histological review. Our study was approved by the Ethics Committee of the Sixth Affiliated Hospital of Sun Yat-sen University (2020ZSLYEC-232). All patients signed written informed consent forms before treatment.

### Stable cell lines

Stable cell line construction was performed as described previously^43^. Briefly, indicated lentiviral vectors were packaged in HEK293T cells. HCT15 or RKO cells were infected with lentiviruses in the presence of polybrene and were selected with 1 µg/mL puromycin for two weeks to obtain stable clones. The indicated protein expression in stable clones was validated by western blotting.

### Real-time quantitative PCR (RT-qPCR)

RT-qPCR assays were performed as described previously^43^. Briefly, total RNA was isolated from cells or tissues and subsequent reverse transcription was performed. qPCR was then performed with SYBR Green Supermix (Bio-Rad, Hercules, CA) using standard procedures. The β-catenin targets in this study were obtained from previous study^73^. All primer sequences used are listed in Supplementary Table S5. *GAPDH* was used as an internal control.

### Co-IP and immunoblot analysis

Co-IP and immunoblot analysis were performed as described previously^43^. Briefly, cells transfected with the indicated plasmids were lysed in 1 mL Lysis buffer. For immunoprecipitation, the anti-Flag agarose beads were washed with 1 mL lysis buffer three times, and then 0.9 mL of cell lysate was added into the indicated group and incubated overnight at 4 °C. The next day, the agarose beads were centrifuged and the supernatant was discarded. Subsequently, the agarose beads were washed three times and mixed in a 2× SDS sample buffer. Lysate samples were boiled for 10 min and were analyzed by immunoblotting with the indicated antibodies.

### Palmitoylation assays

For detecting protein palmitoylation, the acyl-biotin exchange (ABE) method was used^74,75^. Briefly, cells transfected with the indicated plasmids were lysed in 1 mL Lysis buffer containing 50 mM NEM, followed by centrifugation (20 min, 12,000 rpm, 4 °C) and immuno-precipitation overnight with anti-Flag agarose beads. After washing three times, precipitates were divided evenly into two sections, with 1/2 used for the –HAM control, and the remaining 1/2 was used for the +HAM for 1 h at room temperature. The precipitates were gently washed once with Wash Buffer (1 M Tris-HCl, pH 6.5), and incubated with BMCC-biotin Buffer (50 mM Tris-HCl, pH 6.5, 150 mM NaCl, 5 mM EDTA, 1% Triton X-100, and 5 μM BMCC-biotin) for 1 h at 4 °C. Then the precipitates were gently washed two times again with Wash Buffer. After washing samples were analyzed by SDS-PAGE and blotting, palmitoylated β-catenin was detected with HRP-conjugated streptavidin (Sangon Biotech; 1:200 in 0.5% BSA).

### Streptavidin pulldown-based quantification of palmitoylated β-catenin

Streptavidin pulldown-based quantification of palmitoylated protein was performed as previously described^76^. Briefly, cells were lysed in Lysis buffer, and 80 μL supernatant was saved as input. The remaining supernatant was used for ABE experiments. Then palmitoylated proteins were enriched using streptavidin agarose (Cytiva) with rotation overnight at 4 °C. Samples were centrifugated at 3000 rpm for 5 min. 80 μL supernatant was saved as output. Protein-bound streptavidin agaroses were washed three times with Wash Buffer and bound proteins were eluted with SDS loading buffer for 10 min at 95 °C. Samples were subjected to SDS-PAGE. The fraction of palmitoylated β-catenin was determined by western blotting and calculated based on the β-catenin protein level in input and output samples. β-actin was blotted as a loading control.

### ChIP assay

Cells were cross-linked in situ with 1% formaldehyde for 10 min, quenched with 0.125 M glycine for 5 min at room temperature, and lysed in SDS Lysis buffer. Total lysates were sonicated to smash chromatin DNA to a size range of 200–1000 bp. The supernatant was diluted 10 times in ChIP Dilution buffer and precleared with 50 μL agarose beads for 2 h at 4 °C. Then the supernatant was collected by centrifugation, and the indicated antibodies (2 μg) were added to the supernatant. Then, the mixture was rotated overnight at 4 °C. The next day, 50 μL agarose beads were added, and rotation was continued for 2 h at 4 °C. Subsequent de-crosslinked DNA was subjected to PCR analysis using specific primers listed in Supplementary Table S5.

### Ubiquitination assay

Ubiquitination assays were performed as described previously^43^. Briefly, HEK293T cells were transfected with the indicated plasmids and treated with 20 μM MG132 for 6 h before collection. The cells were then lysed in RIPA lysis buffer and denatured by heating at 95 °C for 5 min. Immunoprecipitation analysis was performed as described above. The samples were boiled for 10 min in SDS sample buffer and analyzed by immunoblotting with the indicated antibodies.

### Protein half-life assay

Protein half-life assays were performed as described previously^43^. Briefly, the cells were transfected with the indicated plasmids, and 36 h later, the cells were treated with cycloheximide (CHX, 100 µg/mL) for the indicated time periods before collection. The cells were lysed and proteins were detected by immunoblotting with the indicated antibodies.

### Luciferase reporter assays

0.3 μg pGL3 vector expressing *ACOX1*-TBE, *DUSP14*-RE, or indicated mutant and 50 ng Renilla luciferase reporter were transfected in triplicates into HCT15 or RKO cells. After 36 h, luciferase activities were determined by the Dual-Luciferase Reporter Assay System. The Renilla activity was used as an internal control.

### MS analysis

For protein qualitative analysis, HEK293T cells were transfected with the Flag-ACOX1 plasmid, lysed in Lysis buffer, and immune-precipitated with anti-Flag agarose beads. After SDS-PAGE and Coomassie Blue staining of the Flag-ACOX1-associated complexes, the bands were cut, subjected to in-gel trypsin digestion, and dried. The protein composition and protein site modification were analyzed by MS according to the protocols described previously^77^.

For protein quantitative analysis of CRC samples, five fresh CRC samples and matched normal adjacent tissues were collected from the Sixth Affiliated Hospital of Sun Yat-sen University, and lysed in Urea Lysis buffer. Extracted proteins were subjected to LC-MS/MS (Thermo Fisher Scientific, Rockford, IL, USA) analysis according to the standard protocols^78^. Proteins were identified by Firmiana, a one-stop proteomic data processing platform^79^. Briefly, Mascot (Matrix Science, version 2.3.01) and Nation Center for Biotechnology Information (NCBI) Ref-Seq human proteome database (updated on 04-07-2013) were used in the identification and quantification processes. Peptides (FDR < 0.01) were selected, and the proteins that contain high-quality and unique peptides were considered qualified. Length of minimal peptide was seven amino acids. Label-free intensity-based absolute quantification (iBAQ) was used to quantify proteins^80^. Fraction of total (FOT) was defined as the iBAQ value per protein divided by the sum of all protein iBAQ values. The FOT value was multiplied by 10^5^ for easy representation.

For cell proteome analysis, HCT15 cells were treated with PA (100 μM) or DMSO for 72 h, and lysed in Urea Lysis buffer. Extracted proteins were further digested, purified and measured. One microgram of protein per sample was subjected to LC-MS/MS analysis according to the standard protocols^78–80^.

### Organoid assays

Organoid assays were performed as described previously^81^. Briefly, human intestinal normal epithelial cells were maintained with organoid culture advanced DMEM/F12 containing growth factors (100 ng/mL Noggin (Peprotech), 500 ng/mL R-spondin (Peprotech), 50 ng/mL epidermal growth factor (Peprotech) and 10 μM Y-27632 (Abmole)) and treated with PA (300 μM). After spheroid organoid formation for 5 days, organoids were photographed and measured in diameter.

### IHC assays

Colon samples were fixed and embedded in paraffin according to standard protocols. H&E staining was performed in paraffin-embedded sections using hematoxylin and eosin (Servicebio). The analysis of IHC was performed using indicated antibodies against ACOX1 (Abcam) and Ki67 (Servicebio). The IHC staining results were scored considering both the intensity of staining and the proportion of tumor cells with positive reaction. The intensity of staining was scored as follows: 0, negative; 1, weak; 2, medium; and 3, strong. The frequency of positive cells was scored as follows: 0, < 5%; 1, 1%–25%; 2, 25%–50%; 3, 50%–75%; 4, > 75%. Total score ranging from 0 to 12 was determined by multiplying the score of staining intensity and the score of positive area.

### Statistical analysis

For data analysis, GraphPad Prism 8.3.0, Microsoft Excel, and IBM SPSS Statistics 26 were used. Statistical significance (*P* < 0.05) was performed using the unpaired or paired Student’s *t*-test or *χ*^2^ test. Data are presented as the means ± SD.

## Supplementary information

Supplementary Information

## Data Availability

TCGA CRC RNA-SeqV2, RNA-Seq, and indicated clinical data were downloaded from the University of California Santa Cruz Xena dataset (http://xena.ucsc.edu/). The CPTAC was obtained from the UALCAN website (http://ualcan.path.uab.edu/). Vasaikar’s CPTAC indicated clinical data and the information of *ACOX1* mutations were obtained from the cBioPortal (https://www.cbioportal.org/). CRC array datasets (GSE25070, GSE39582, GSE68468, GSE9348, GSE32323, GSE41258, GSE121128, GSE71187, GSE29623, GSE17537, GSE12945 and GSE17536) were available on GEO (http://www.ncbi.nlm.nih.gov/geo). IHC of HPA was available on the website (http://www.proteinatlas.org/).
