# Author Correction: Reprogramming of palmitic acid induced by dephosphorylation of ACOX1 promotes β-catenin palmitoylation to drive colorectal cancer progression

**DOI:** 10.1038/s41421-023-00540-4

**Published:** 2023-04-03

**Authors:** Qiang Zhang, Xiaoya Yang, Jinjie Wu, Shubiao Ye, Junli Gong, Wai Ming Cheng, Zhanhao Luo, Jing Yu, Yugeng Liu, Wanyi Zeng, Chen Liu, Zhizhong Xiong, Yuan Chen, Zhen He, Ping Lan

**Affiliations:** 1 The Sixth Affiliated Hospital, School of Medicine, Sun Yat-sen University, Guangzhou, Guangdong China; 2 Guangdong Provincial Key Laboratory of Colorectal and Pelvic Floor Diseases, Guangdong Institute of Gastroenterology, Guangzhou, Guangdong China; 3 Center for Synthetic Microbiome, Institute of Synthetic Biology, Shenzhen Institutes of Advanced Technology, Chinese Academy of Sciences, Shenzhen, Guangdong China

**Keywords:** Cancer metabolism, Post-translational modifications, Colorectal cancer, Post-translational modifications

Correction to: *Cell Discovery* (2023) 9:26 10.1038/s41421-022-00515-x published online 07 March 2023

In the original publication of this article^1^, we mistakenly used an incorrect image for the HCT15 (Ctrl+sh*ACOX1*-2#) group in Fig. 2b. The correct Fig. 2b is displayed as below.

**Figure 1:**
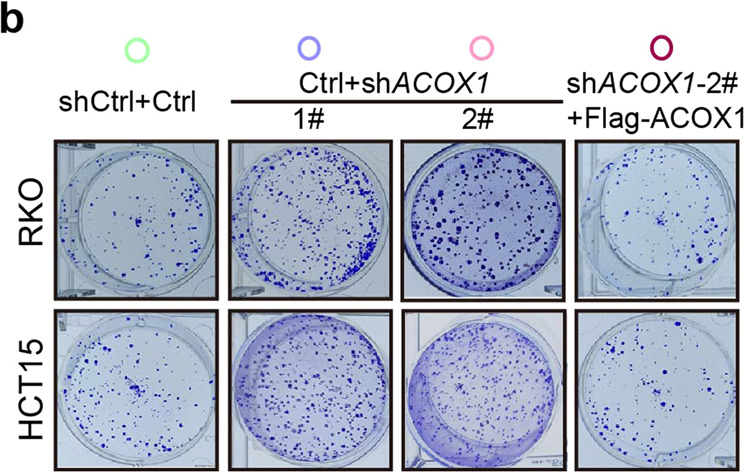
**b** Colony formation of RKO and HCT15 cells stably expressing the indicated vectors.

During the final submission of this manuscript, we inadvertently submitted an earlier version of the Supplementary Fig. S9, in which the DUSP14 blot of patient 14 is incorrect. The correct DUSP14 blot of patient 14 is shown below.

**Figure 2:**
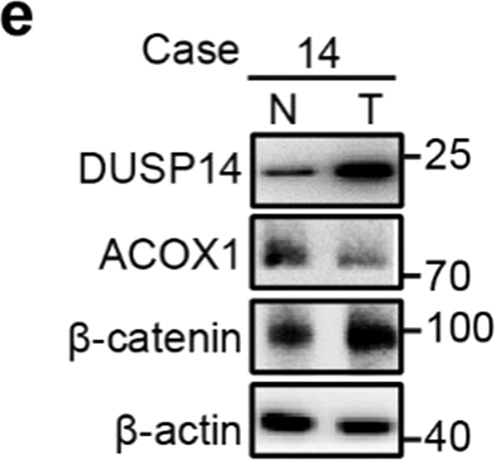
**e** Immunoblot analysis of the indicated proteins in early-stage CRCs from the Sixth Affiliated Hospital of Sun Yat-sen University.

In addition, there are a few errors in the figure legends. In the legend of Fig. 2a, the first “HCT15” should be corrected to “HCT116”; in the legend of Fig. 3h, the “K63R” should be corrected to “K643R”; in the legend of Fig. 8a–c, the “Supplementary Fig. S9d” should be corrected to “Supplementary Fig. S9e”. The correct figure legends are as below.

**Fig. 2a** Enhanced CRC cell viability by *ACOX1* depletion. Cell viability of *ACOX1*-depleted CRC cells (HCT116, RKO, SW620, HCT8, and HCT15) was analyzed for CCK-8.

**Fig. 3h** DUSP14 mediates ubiquitination of ACOX1 at K643. Myc-Ub was co-transfected with HA-DUSP14 and Flag-ACOX1 (WT, K29R, K241R, K255/260R, K446R, or K643R) into HEK293T cells, and the cell lysates were subjected to immunoprecipitation.

**Fig. 8a**–**c** Relative protein levels of DUSP14 (**a**), ACOX1 (**b**) and β-catenin (**c**). The proteins were quantified by densitometry, with β-actin as a normalizer, as shown in Supplementary Fig. S9e.

These corrections do not affect the results or the conclusion of this work. We are sorry for the inconvenience caused.
