# Supplementary material for: Reprogramming of palmitic acid induced by dephosphorylation of ACOX1 promotes β-catenin palmitoylation to drive colorectal cancer progression
Source: Cell Discov. 2023 Mar 7;9:26. doi: 10.1038/s41421-022-00515-x (PMC9988979; doi:10.1038/s41421-022-00515-x)
Supplement: Supplementary file 1 — Supplementary Information [file 41421_2022_515_MOESM1_ESM.pdf]

Supplementary Information

Reprogramming of palmitic acid induced by dephosphorylation of ACOX1 promotes β-catenin palmitoylation to drive colorectal cancer progression

Qiang Zhang, Xiaoya Yang, Jinjie Wu, Shubiao Ye, Junli Gong, Wai Ming Cheng, Zhanhao Luo, Jing Yu, Yugeng Liu, Wanyi Zeng, Chen Liu, Zhizhong Xiong, Yuan Chen, Zhen He, Ping Lan

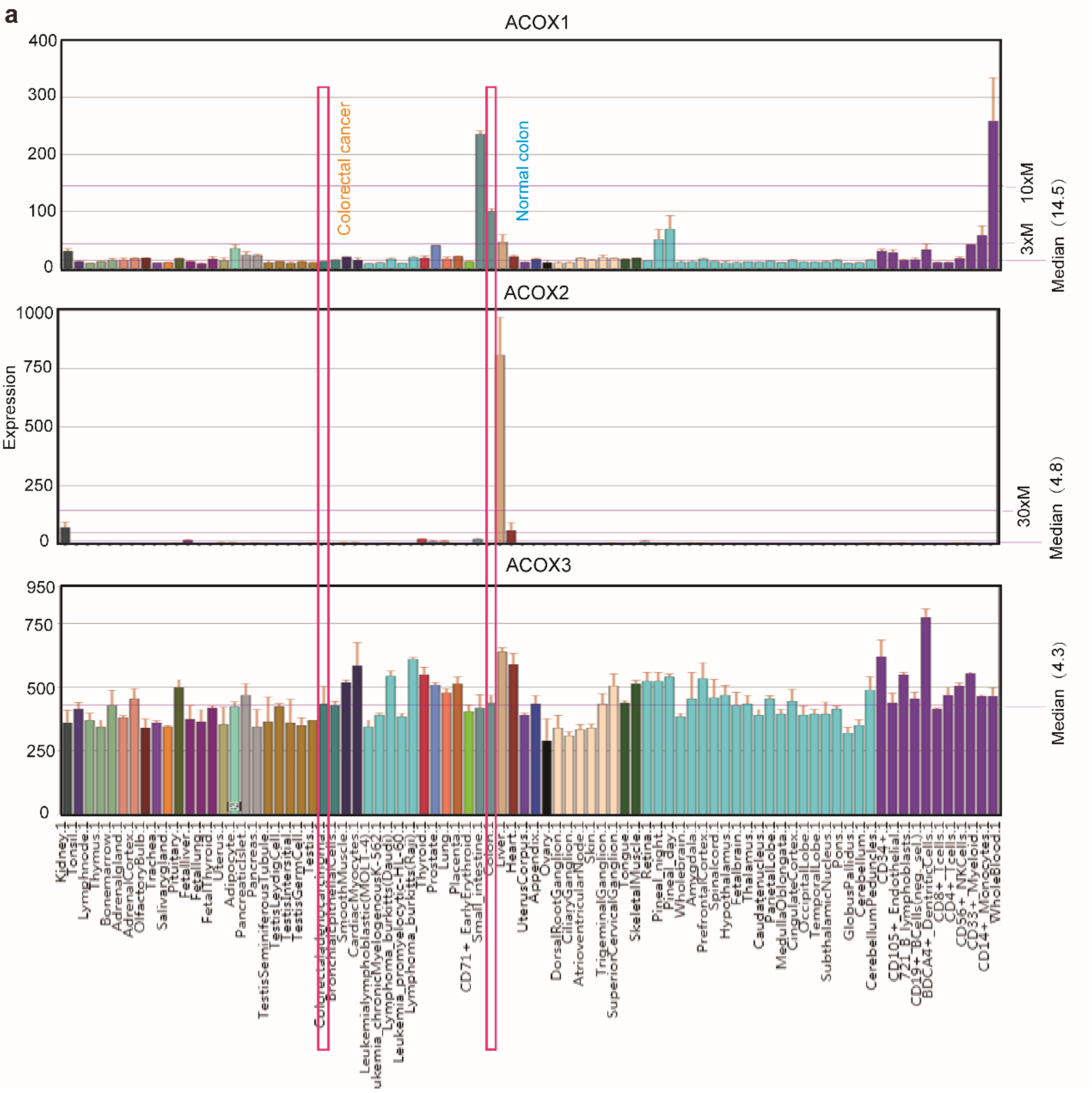
a
ACOX1
400
300
200
100
0
Colorectal cancer
Normal colon
10xM
3xM
Median（14.5）
ACOX2
1000
750
500
250
0
Expression
30xM
Median（4.8）
ACOX3
950
750
500
250
0
Median（4.3）

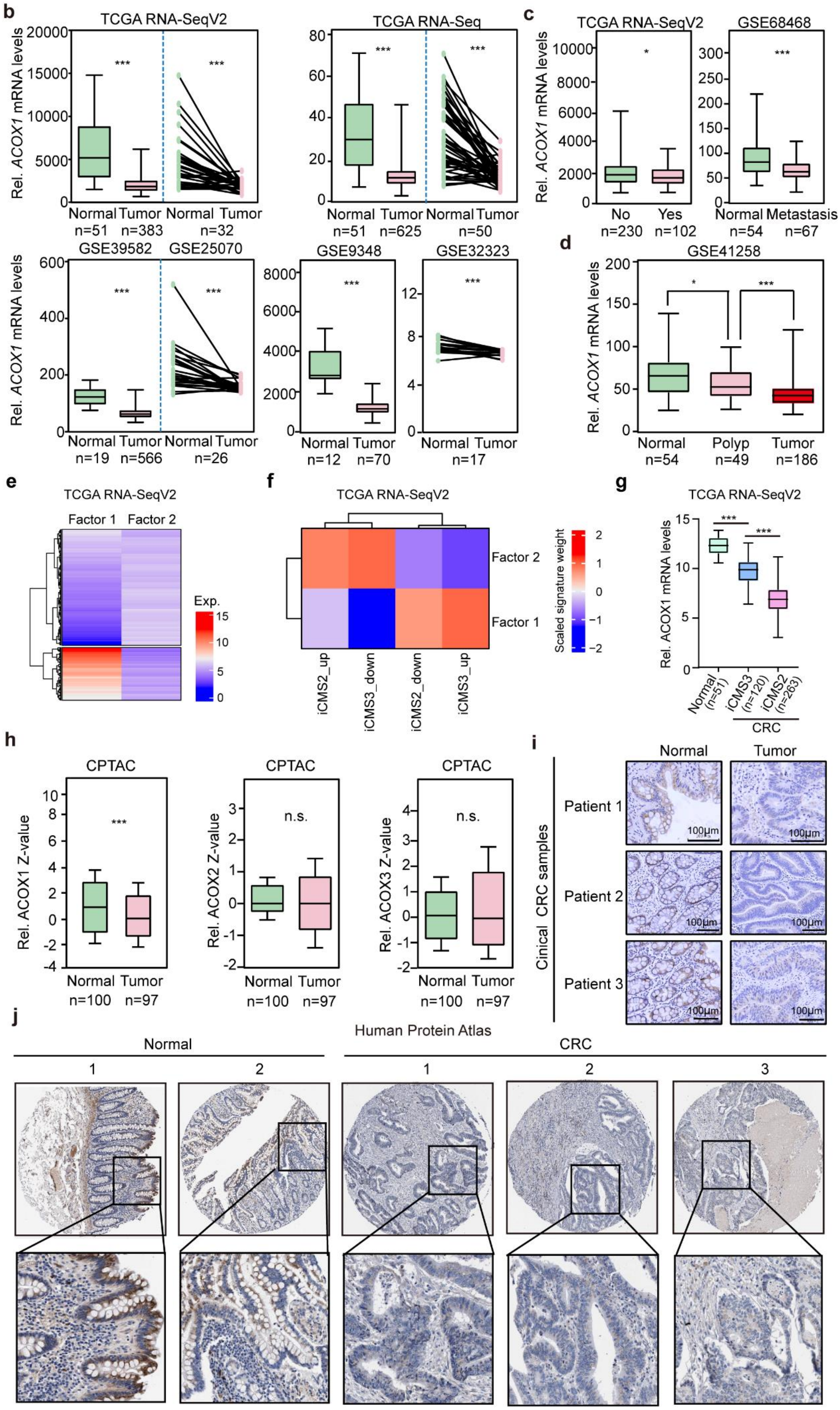
b
TCGA RNA-SeqV2
Rel. ACOX1 mRNA levels
Normal
Tumor
n=51
n=383
n=32
TCGA RNA-Seq
n=625
n=50
c
TCGA RNA-SeqV2
GSE68468
No
Yes
n=230
n=102
Metastasis
n=54
n=67
GSE39582
GSE25070
n=19
n=566
n=26
GSE9348
GSE32323
n=12
n=70
n=17
d
GSE41258
Polyp
n=49
n=186
e
Factor 1
Factor 2
Exp.
f
Factor 2
Factor 1
Scaled signature weight
iCMS2_up
iCMS3_down
iCMS2_down
iCMS3_up
g
Rel. ACOX1 mRNA levels
CRC
h
CPTAC
Rel. ACOX1 Z-value
Rel. ACOX2 Z-value
Rel. ACOX3 Z-value
n.s.
n=100
n=97
i
Cinical CRC samples
Patient 1
Patient 2
Patient 3
100μm
j
Human Protein Atlas
CRC

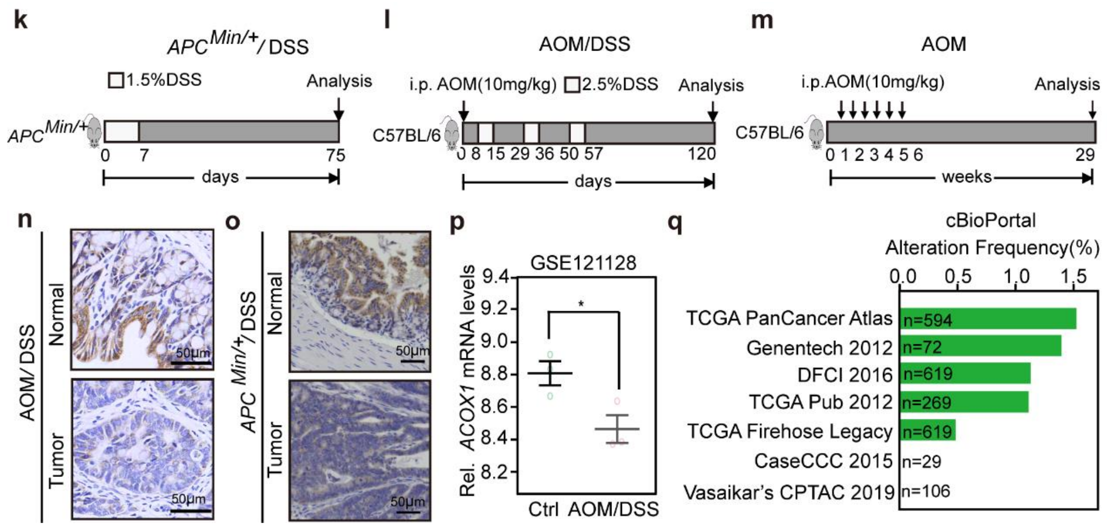

Supplementary Fig. S1 ACOX1 is downregulated in CRCs. Related to Fig. 1.

a *ACOX1* (not *ACOX2* and *ACOX3*) was downregulated in CRCs. Bar chart showing full expression profiles of *ACOX1*, *ACOX2*, and *ACOX3* from the BioGPS website (http://biogps.org/).

b Unpaired or paired analysis of *ACOX1* expression in adjacent normal tissues versus tumor tissues from TCGA RNA-SeqV2, TCGA RNA-Seq and GEO (GSE39582, GSE25070, GSE9348 and GSE32323).

c Analysis of *ACOX1* expression in samples with and without lymph node invasion and adjacent normal tissues versus metastatic tissues from TCGA RNA-SeqV2 and G68468. Yes, lymph node invasion; No, no lymph node invasion.

d Analysis of *ACOX1* expression in adjacent normal tissues, polyp tissues, and tumor tissues from GSE41258.

e Cluster analysis of TCGA RNA-SeqV2 transcriptomics based on iCMS classification[37].

f Determination of iCMS classification. Factor 1 refers to iCMS3; Factor 2 refers to iCMS2.

g Analysis of *ACOX1* expression in adjacent normal colon samples, iCMS2 tumor samples and iCMS2 tumor samples from TCGA RNA-SeqV2.

h Analysis of ACOX1, ACOX2 and ACOX3 expression in adjacent normal tissues versus primary tumor tissues from CPTAC dataset.

i Representative ACOX1 staining of paired CRCs from The Sixth Affiliated Hospital of Sun Yat-sen University. Scale bars, 100 μm.

j ACOX1 staining of unpaired CRCs from Human Protein Atlas (HPA) database.

k-m Schematic diagram showing the experimental design for mouse CRC models. Briefly, $APC^{Min/+}$mice (n=5, per group) were exposed to 1.5% DSS for a week and analyzed at 75 days (**k**). C57BL/6 mice (n=5, per group) were injected intraperitoneally with AOM (10 mg/kg), given drinking water containing 2.5% DSS, and analyzed at 120 days (**l**). C57BL/6 mice (n=5, per group) were injected intraperitoneally with AOM (10 mg/kg) for six times, and analyzed at 120 weeks (**m**).

n-o ACOX1 is poorly expressed in mouse CRCs. Representative ACOX1 staining of mice in control and CRC group. Scale bars, 50 μm.

p Analysis of *ACOX1* expression of control and AOM/DSS-induced mouse CRC group from GSE121128.

q Genetic alteration of *ACOX1* determined for the cBioportal database.

Data were analyzed using unpaired or paired Student's t-test (**b**, **c**, **d**, **g**, **h**, **p**). Data are presented as mean ± SD; *$P < 0.05$, ** $P < 0.01$, *** $P < 0.001$; n, number of patient or mouse samples; n.s., not significant.

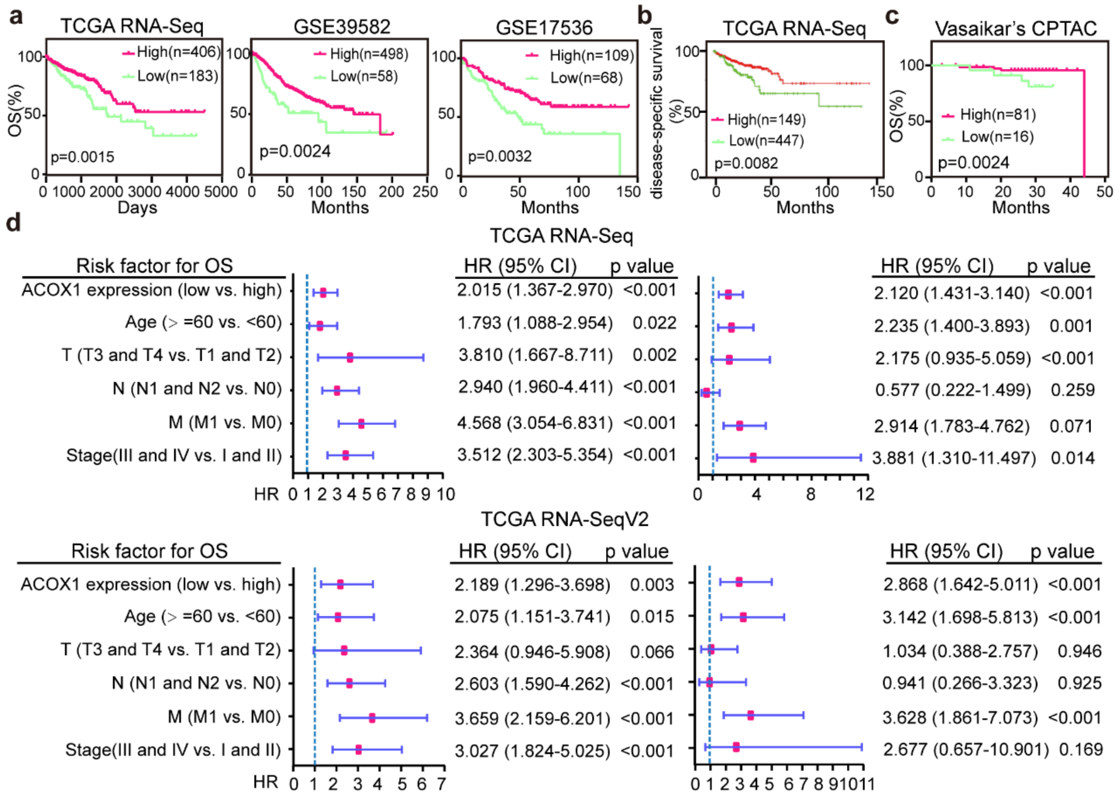

Supplementary Fig. S2 ACOX1 downregulation predicts poor survival in CRC. Related to Fig. 1.

a Kaplan–Meier overall survival curves of human CRC patients with low versus high *ACOX1* expression, based on TCGA RNA-Seq, GSE39582 and GSE17536.

b Kaplan–Meier disease-specific survival curves of human CRC patients with low versus high *ACOX1* expression, based on TCGA RNA-Seq.

c Kaplan–Meier overall survival curves of human CRC patients with low versus high ACOX1 protein expression, based on Vasaikar's CPTAC.

d *ACOX1* expression is an independent prognostic factor for poor survival. Forest plot showing univariate (**left**) and multivariate (**right**) Cox analysis of different clinical parameters for CRC patients in TCGA RNA-Seq (**upper**) and RNA-SeqV2 (**lower**). HR, hazard ratio; CI, confidence interval. n, number of patient samples.

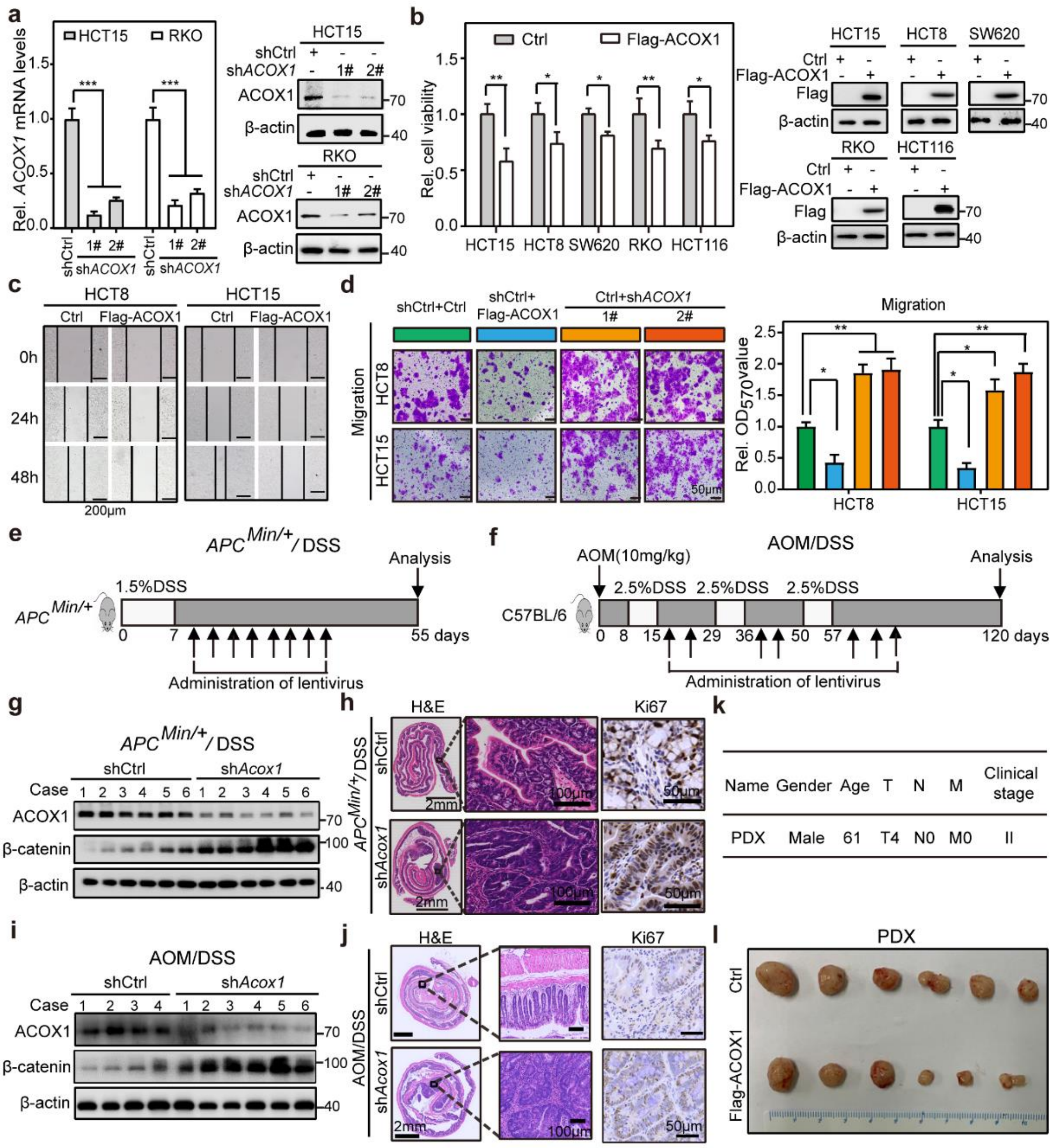

Supplementary Fig. S3 ACOX1 inhibits CRC cell proliferation and migration *in vitro* and tumor growth *in vivo*. Related to Fig. 2.

a Analysis of ACOX1 expression. HCT15 and RKO cells were transfected with ACOX1 shRNA plasmids and analyzed for RT-qPCR (**Left**) and immuno-blot (**Right**).

b Inhibition of CRC cell viability by ACOX1 overexpression. Cell viability of CRC cells (HCT15, RKO, SW620, HCT8, and HCT116) stably expressing Flag-ACOX1 was analyzed for CCK-8 (**Left**). Expression of ACOX1 was analyzed by immuno-blot (**Right**).

c-**d** Inhibition of CRC cell migration by ACOX1 overexpression. Cell motility was analyzed by wound-healing assay (**c**) and migration assay (**d**). Scale bars, 200 μm (**c**) and 50 μm (**d**).

e-**f** Schematic diagram showing the experimental design for mouse CRC models. Lentivirus was injected intraperitoneally as indicated during DSS treatment.

g Expression of ACOX1 and β-catenin protein in the colon from **e**. Representative colons from CRC mouse induced by $APC^{Min/+}$/DSS were collected for immuno-blot.

h Representative H&E and Ki67 staining of mouse CRCs from **e**. The scale bars are shown in the images.

i Expression of ACOX1 and β-catenin protein in the colon from **f**. Representative colons from CRC mouse induced by AOM/DSS were collected for immuno-blot.

j Representative H&E and Ki67 staining of mouse CRCs from **f**. Scale bars are shown in the images.

k Clinical information for PDX from CRC patient's tumor tissues.

l Tumor photographs from PDXs.

Data were analyzed using unpaired Student's t-test (**a**, **b**, **d**). Data are presented as mean ± SD; *$P < 0.05$, ** $P < 0.01$, *** $P < 0.001$.

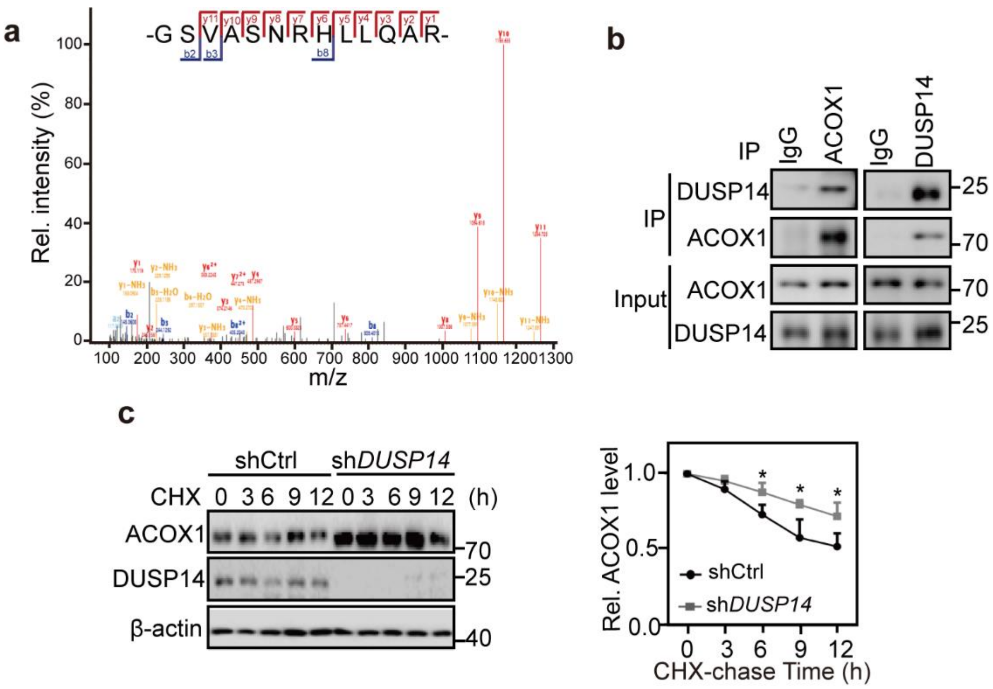

Supplementary Fig. S4 DUSP14 depletion enhances ACOX1 stability. Related to Fig. 3.

a Mass spectrometry analysis of the Flag-ACOX1-associated peptides corresponding to DUSP14.

b Endogenous interaction between ACOX1 and DUSP14. RKO cells were treated with MG132 (20 μM) for 6 h before harvest and cell lysates were analyzed for Co-IP.

c Time-course analysis of ACOX1 protein levels in DUSP14-depleted RKO cells (**left**). ACOX1 proteins were quantified by densitometry, with β-actin as a normalizer (**right**). Data were analyzed using unpaired Student's t-test (**c**). Data are presented as mean ± SD; *$P < 0.05$.

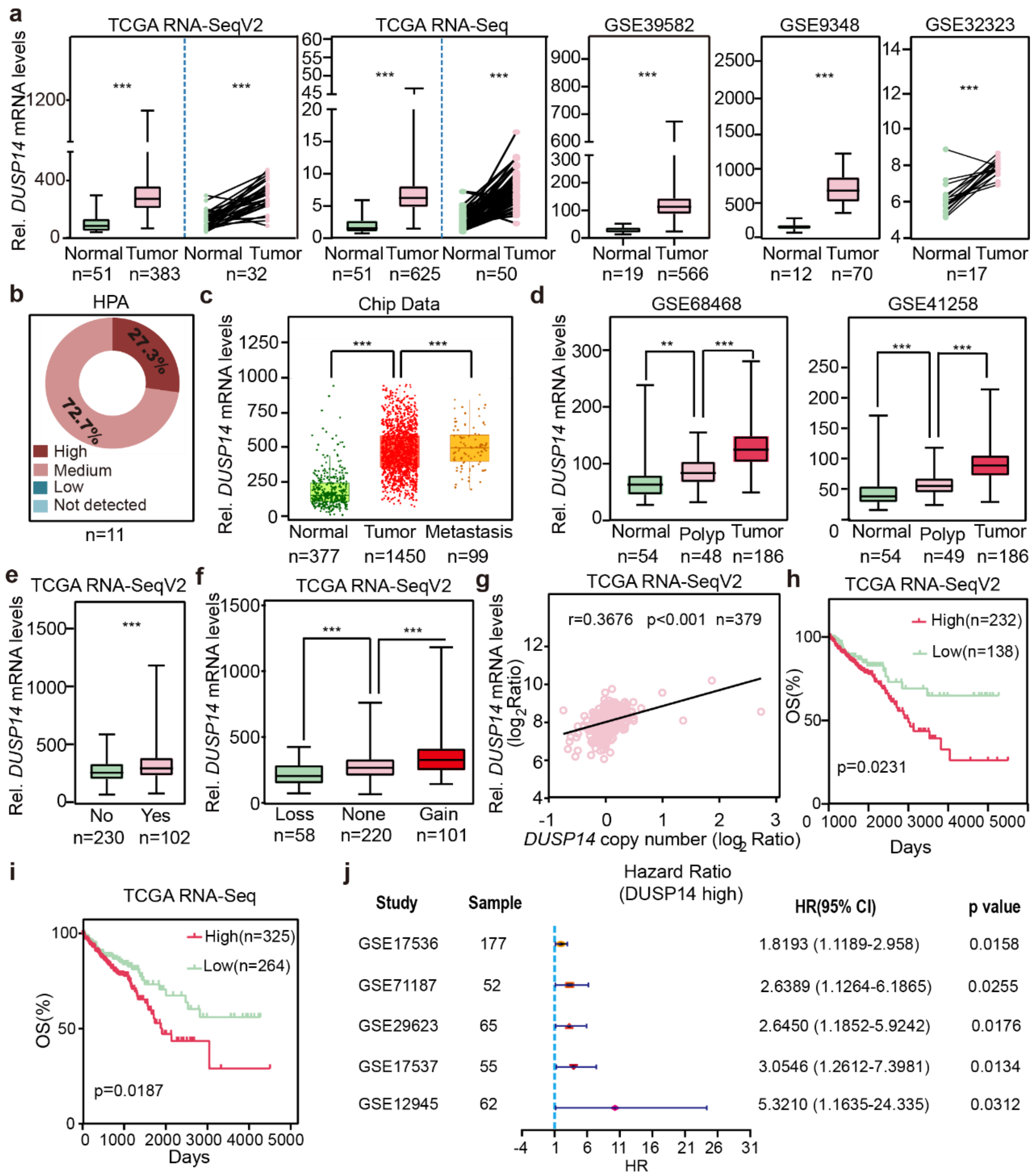

Supplementary Fig. S5 DUSP14 is highly expressed and associated with poor prognosis in CRC. Related to Fig. 3.

a Unpaired or paired analysis of *DUSP14* expression in adjacent normal tissues versus tumor tissues from TCGA RNA-SeqV2, RNA-Seq, and GEO (GSE39582, GSE9348, and GSE32323).

b Pie chart showing the IHC results of DUSP14 in CRCs from the HPA database. “Not detected” or “Low” means low expression of the indicated protein; “High” or “Medium”

means high expression of DUSP14. Antibody HPA019911 was used for DUSP14.

c Analysis of *DUSP14* expression in adjacent normal tissues, primary tumor tissues, and metastatic tumors from gene chip data.

d Analysis of *DUSP14* expression in adjacent normal tissues, polyp tissues and tumor tissues from GEO (GSE68468 and GSE41258).

e Analysis of *DUSP14* expression in samples with and without lymph node invasion, based on TCGA RNA-SeqV2 dataset. Yes, lymph node invasion; No, no lymph node invasion.

f Relationship between *DUSP14* expression and *DUSP14* copy number variations, based on TCGA RNA-SeqV2.

g Positive correlation between *DUSP14* expression and *DUSP14* copy number variations, based on TCGA RNA-SeqV2.

h-i Kaplan–Meier overall survival curves of human CRC patients with low versus high *DUSP14* expression, based on TCGA RNA-SeqV2 and TCGA RNA-Seq.

j *DUSP14* high expression predicts poor prognosis in CRC. Forest map showing the relationship between DUSP14 high expression and CRC patient prognosis, based on GEO (GSE17536, GSE71187, GSE29623, GSE17537 and GSE12945).

Data were analyzed using unpaired or paired Student's t-test (**a**, **c**, **d**-**f**). Data are presented as mean ± SD; $^{**}P < 0.01$, $^{***} P < 0.001$.

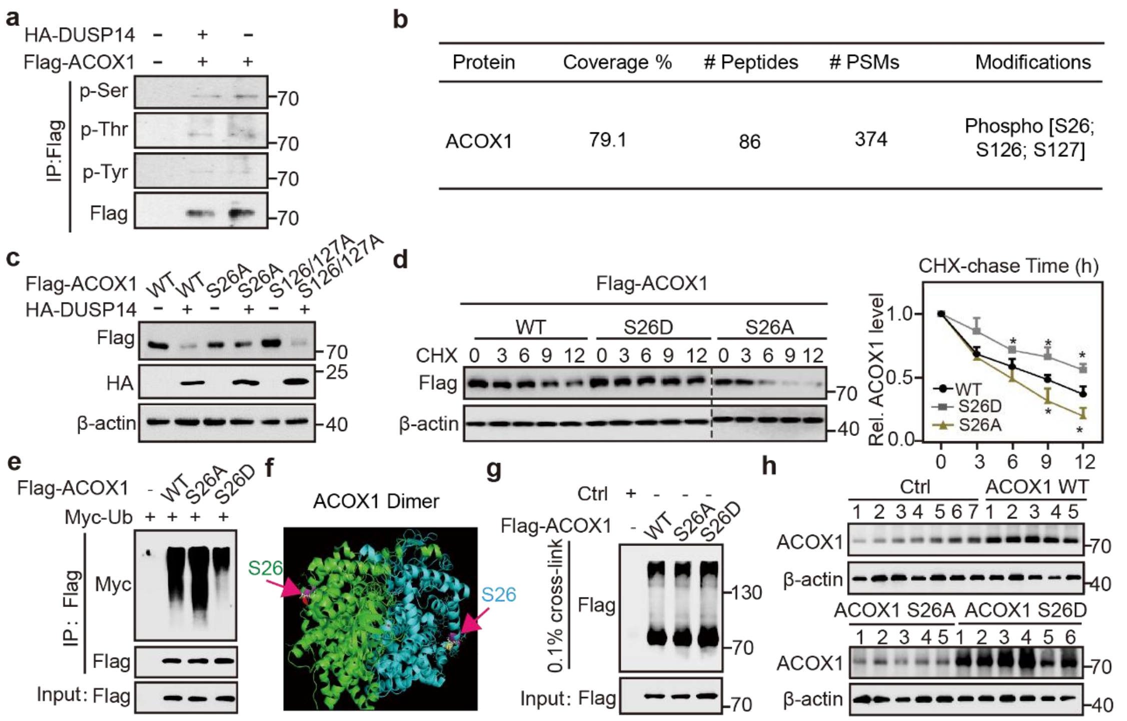

Supplementary Fig. S6 DUSP14 dephosphorylated ACOX1 at S26. Related to Fig 4.

a Decreased ACOX1 serine phosphorylation by DUSP14 overexpression. HEK293T cells were transfected with Flag-ACOX1 or/and HA-DUSP14 and the cell lysates were subjected to immunoprecipitation.

b Peptide information of ACOX1 serine phosphorylation, based on MS result.

c Un-alteration of ACOX1 S26A mutant stability by DUSP14 overexpression. HEK293T cells were transfected with Flag-ACOX1 (WT, S26A, or S126/127A) and/or DUSP14, and the cell lysates were analyzed by immunoblotting.

d Time-course analysis of ACOX1 protein levels in Flag-ACOX1 WT, S26A, or S26D-overexpressed HEK293T cells (**left**). ACOX1 proteins were quantified by densitometry, with β-actin as a normalizer (**right**).

e Polyubiquitination levels of ACOX1 WT, ACOX1 S26A, and ACOX1 S26D.

f ACOX1 S26 is located on the outer surface of dimeric ACOX1. The structure of dimeric ACOX1 in Rat, the arrows represent ACOX1 S26.

g The same dimer-forming ability between ACOX1 WT and ACOX1 mutants (S26A and S26D). HEK293T cells were transfected with Flag-ACOX1 WT, S26A or S26D and treated with MG132 (20 μM) for 6 h before harvest, then the cell lysates were treated with 0.1% glutaraldehyde and determined by immunoblot.

h Immunoblot analysis of the indicated tissues from Fig.4D.

Data were analyzed using unpaired Student's t-test (**d**). Data are presented as mean ± SD; $^*P < 0.05$.

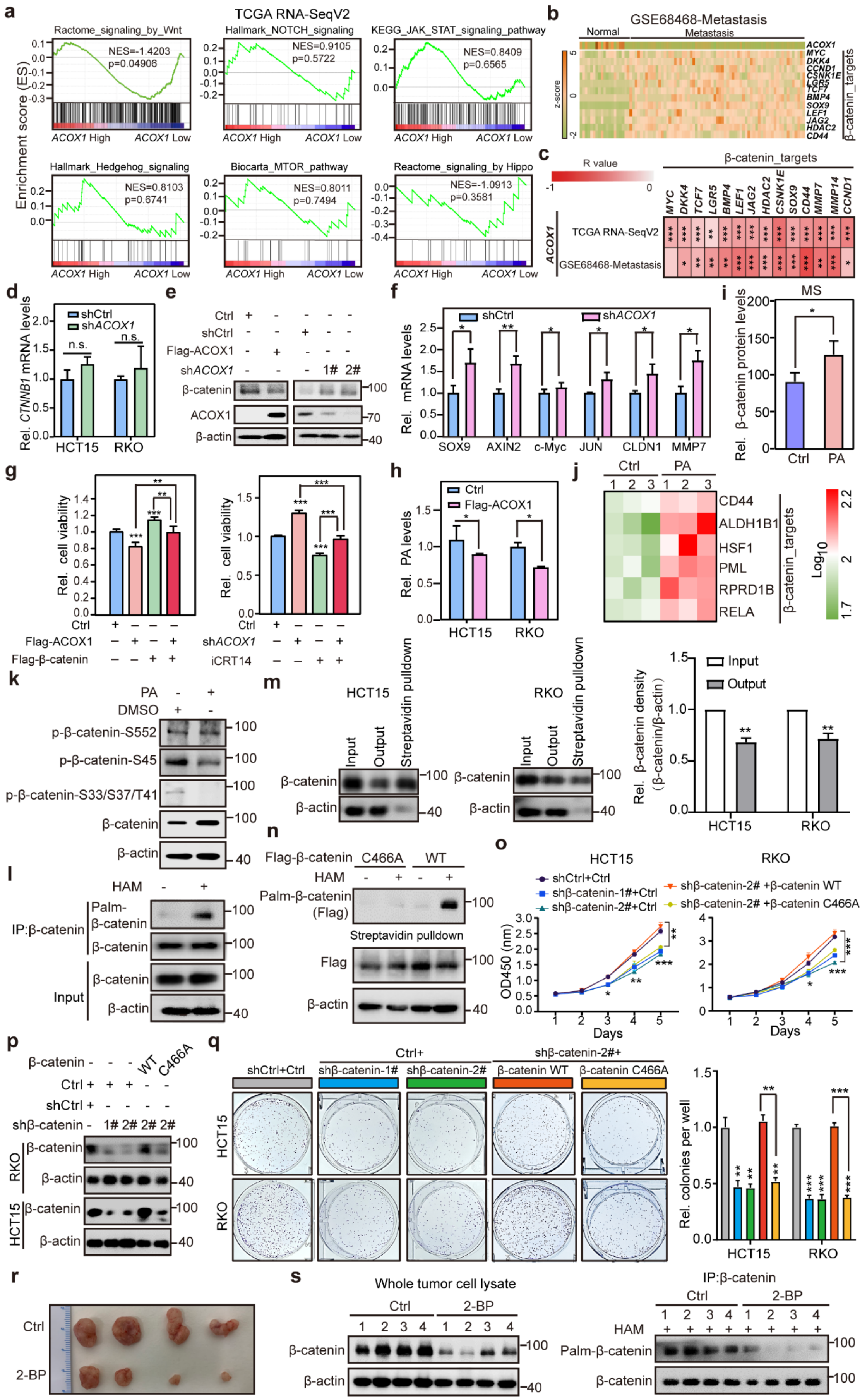
TCGA RNA-SeqV2
GSE68468-Metastasis
Enrichment score (ES)
Ractome_signaling_by_Wnt
NES=-1.4203
p=0.04906
Hallmark_NOTCH_signaling
NES=0.9105
p=0.5722
KEGG_JAK_STAT_signaling_pathway
NES=0.8409
p=0.6565
Hallmark_Hedgehog_signaling
NES=0.8103
p=0.6741
Biocarta_MTOR_pathway
NES=0.8011
p=0.7494
Reactome_signaling_by Hippo
NES=-1.0913
p=0.3581
ACOX1 High
ACOX1 Low
Normal
Metastasis
ACOX1 MYC DKK4 CCND1 CSNK1E LGR5 TCF7 BMP4 SOX9 LEF1 JAG2 HDAC2 CD44
β-catenin_targets
R value
β-catenin_targets
MYC DKK4 TCF7 LGR5 BMP4 LEF1 JAG2 HDAC2 CSNK1E SOX9 CD44 MMP7 MMP14 CCND1
ACOX1
TCGA RNA-SeqV2
GSE68468-Metastasis
Rel. CTNNB1 mRNA levels
shCtrl
shACOX1
HCT15 RKO
Ctrl
shCtrl
Flag-ACOX1
shACOX1
β-catenin
ACOX1
β-actin
Rel. mRNA levels
SOX9 AXIN2 c-Myc JUN CLDN1 MMP7
MS
Rel. β-catenin protein levels
Ctrl PA
Rel. cell viability
Flag-ACOX1
Flag-β-catenin
iCRT14
Rel. PA levels
Ctrl
Flag-ACOX1
CD44 ALDH1B1 HSF1 PML RPRD1B RELA
Log10
PA
DMSO
p-β-catenin-S552
p-β-catenin-S45
p-β-catenin-S33/S37/T41
β-catenin
β-actin
HCT15
RKO
Input
Output
Streptavidin pulldown
Rel. β-catenin density (β-catenin/β-actin)
HAM
IP:β-catenin
Palm-β-catenin
Input
Flag-β-catenin C466A WT
Palm-β-catenin (Flag)
Flag
shCtrl+Ctrl
shβ-catenin-1#+Ctrl
shβ-catenin-2#+Ctrl
shβ-catenin-2# +β-catenin WT
shβ-catenin-2# +β-catenin C466A
OD450 (nm)
Days
shβ-catenin
Ctrl+
shβ-catenin-2#+
shCtrl+Ctrl
shβ-catenin-1#
shβ-catenin-2#
β-catenin WT
β-catenin C466A
Rel. colonies per well
Ctrl
2-BP
Whole tumor cell lysate
IP:β-catenin

Supplementary Fig. S7 ACOX1 depletion enhances β-catenin stability and its transcriptional activity via PA-mediated β-catenin palmitoylation. Related to Fig. 5 and Fig. 6.

a Negative correlation between *ACOX1* and Wnt signaling. GSEA of cancer-related gene sets in the expression profiles of TCGA RNA-SeqV2 according to the expression of *ACOX1*.

b High expression of β-catenin target genes in metastatic tissues. Heatmap showing the expression of β-catenin target genes in normal tissues and metastatic tissues from GSE68468.

c Negative correlation between *ACOX1* and β-catenin target genes. The expression correlation between *ACOX1* and β-catenin target genes was analyzed, based on TCGA RNA-SeqV2 and GSE68468, and the results were presented as a heatmap. Person correlation was used.

d Analysis of *CTNNB1* mRNA expression in ACOX1-depleted CRC cells. CRC cells (HCT15 and RKO) stably expressing *ACOX1* shRNA were subjected to RT-qPCR.

e Expression of β-catenin and ACOX1 proteins analyzed by immunoblot. *ACOX1*-depleted or ACOX1-overexpressed RKO cell lysates were subjected to immunoblot.

f Increased β-catenin target genes expression by *ACOX1* depletion. RKO cells stably expressing control shRNA or *ACOX1* shRNA were analyzed for RT-qPCR.

g ACOX1-induced HCT15 cell viability inhibition is rescued by β-catenin. RKO cells stably expressing Flag-ACOX1 or Flag-β-catenin were cultured for 5 days and counted by CCK-8 (**left**). Suppression of sh*ACOX1*-induced cell hyper viability by β-catenin

inhibition. RKO cells (shCtrl or sh*ACOX1* stable expressed) were treated with iCRT14 (100 μM) for 5 days and counted by CCK-8 (**right**).

h PA, a substrate of ACOX1. PA levels of CRC cells (HCT15 and RKO) stably expressing Flag-ACOX1 were measured, using an ELISA kit.

i-j Enhanced β-catenin abundance (**i**) and β-catenin targets (**j**) by PA treatment. HCT15 cells were treated with PA (100 μM) for 72 h, cell lysates were subjected to MS, β-catenin protein (**i**) and β-catenin targets (**j**) were anayzed.

k Analysis of β-catenin phosphorylation by immunoblot. RKO cells were treated with PA (100 μM) for 24 h and the cell lysates were subjected to immunoblot.

l Palmitoylation of endogenous β-catenin. HEK293T cells were lysed for ABE assay.

m Quantification of endogenous palmitoylated β-catenin. HCT15 and RKO cells were lysed for ABE assay. Then endogenous palmitoylated β-catenin was captured by using streptavidin agaroses (**Left**). Input, output, and streptavidin pulldown were analyzed by immunoblot to quantify the percentage of palmitoylated endogenous β-catenin protein (**Right**). n = 3 experimental replicates.

n Palmitoylation of β-catenin WT and β-catenin C466A mutant. HEK293T cells were transfected with Flag-β-catenin WT or C466A mutant and cell lysates were analyzed for ABE assay. Palmitoylated β-catenin was then captured by using streptavidin agaroses, and analyzed by immunoblotting with anti-Flag antibody.

o Proliferation of HCT15 and RKO cells was measured by CCK-8 assay upon shRNA 3'UTR depletion of endogenous β-catenin in the presence or absence of ectopically expressed β-catenin WT or C466A.

p Immuno-blot of above stable cell lines (**o**).

q Colony formation of RKO and HCT15 cells stably expressing the indicated vectors (**left**), and bar graphs showing the relative colony numbers (**right**).

r Representative tumor photograph from **Fig. 6k**.

s Expression of total β-catenin protein (**left**) and palmitoylated β-catenin protein (**right**) in tumors from **Fig. 6k**. Representative tumors from **Fig. 6k** were performed for immuno-blot.

Data were analyzed using unpaired or paired Student's t-test (**d**, **f**, **g**, **h**, **i**, **m**, **o**, **q**). Data are presented as mean ± SD; *$P < 0.05$, ** $P < 0.01$, *** $P < 0.001$; n.s., not significant.

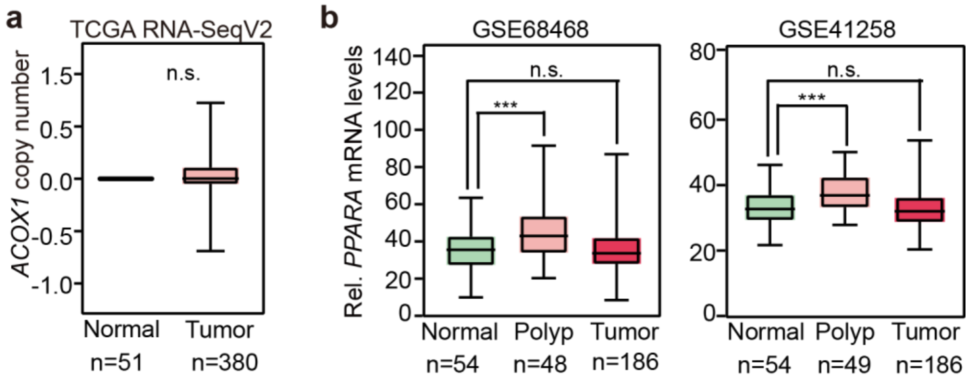

Supplementary Fig. S8 ***ACOX1*** **copy number variations and transcription factor** ***PPARA*** **dysregulation are not the cause of ACOX1 downregulation in CRC. Related to Fig. 7.**

a Analysis of *ACOX1* copy number variations in normal tissues and tumor tissues, based on TCAG RNA-SeqV2.

b Analysis of *PPARA* expression in normal tissues, colon polyps, and tumor tissues from GSE68468 and GSE41258.

Data were analyzed using unpaired Student's t-test (a-c). Data are presented as mean ±

SD; *** $P < 0.001$; n.s., not significant.

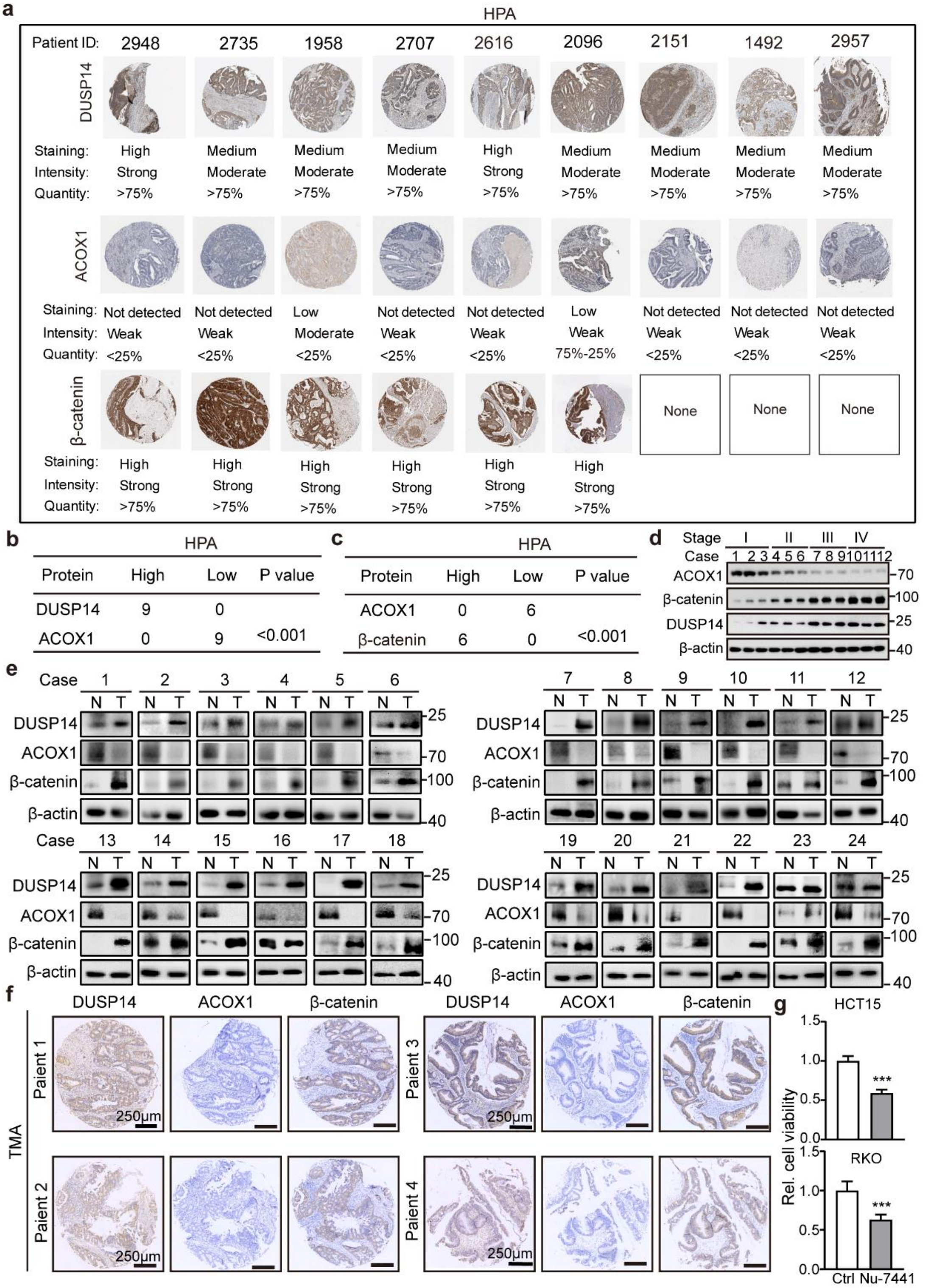

Supplementary Fig. S9 ACOX1 is downregulated, while DUSP14 and β-catenin are upregulated in CRCs. Related to Fig. 8.

a Images showing IHC of DUSP14, ACOX1, and β-catenin from the same patients,

based on the HPA dataset.

b Negative correlation between DUSP14 and ACOX1. Indicated protein expression from **a** was determined, correlation analysis was performed using a chi-squared test. "Not detected" or "Low" means low expression of the indicated protein; "Medium" or "High" means high expression of the indicated protein.

c Negative correlation between ACOX1 and β-catenin. Indicated protein expression from **a** was determined, correlation analysis was performed using a chi-squared test. "Not detected" or "Low" means low expression of the indicated protein; "Medium" or "High" means high expression of the indicated protein.

d CRC samples of different stages (TNM, Stage I, II, III and IV) were collected for immunoblotting.

e Immunoblot analysis of the indicated proteins in early-stage CRCs from The Sixth Affiliated Hospital of Sun Yat-sen University.

f Representative IHC for DUSP14, ACOX1 and β-catenin in our CRC TMA.

g Inhibition of CRC cell viability by Nu-7441 treatment. CRC cells (HCT15 and RKO) were treated with Nu-7441, and cell viability was analyzed for CCK-8.

Data were analyzed using Chi-square test (**b**, **c**) or unpaired Student's t-test (**g**). Data are presented as mean ± SD; *** $P < 0.001$.

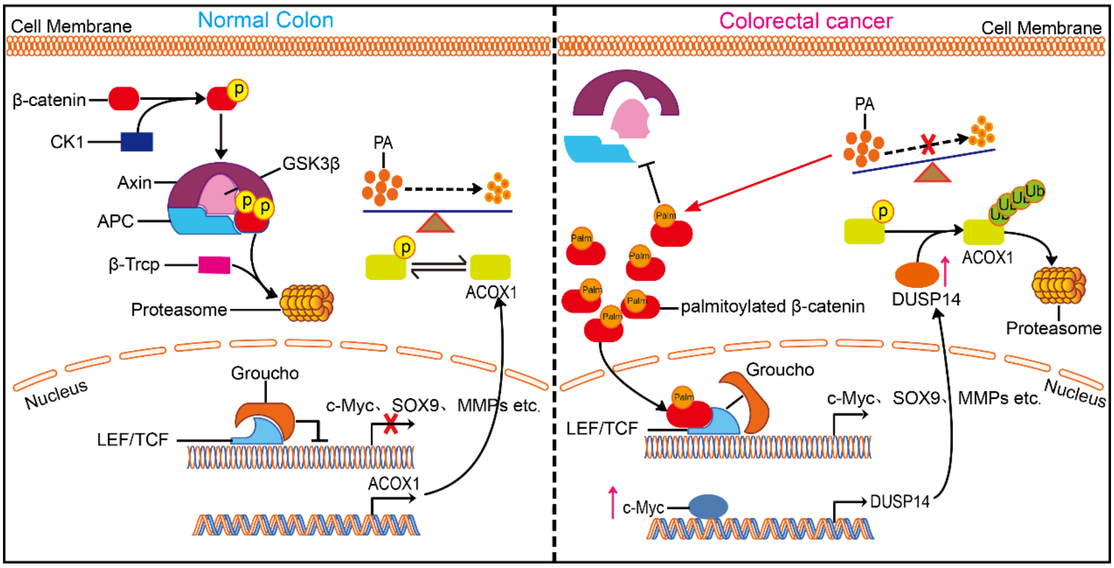

Supplementary Fig. S10 Schematic diagram of the mechanism by which ACOX1 inhibits β-catenin signaling via PA-mediated β-catenin palmitoylation. In the normal colon, β-catenin signaling is strictly regulated to ensure normal cellular activities. In CRC, β-catenin signaling abnormally activates *DUSP14* transcription and inhibits *ACOX1* transcription, resulting in high expression of *DUSP14* and low expression of *ACOX1*. Furthermore, DUSP14 dephosphorylates ACOX1 at serine 26 to promote its proteasomal degradation, thereby increasing PA levels, the substrate of ACOX1. Accumulated PA promotes β-catenin cysteine 466 palmitoylation, which inhibits CK1- and GSK3-directed phosphorylation of β-catenin and subsequent β-Trcp-mediated proteasomal degradation. Moreover, stabilized β-catenin translocates to the nucleus to regulate the transcription of many oncogenes, promoting CRC progression.

Supplementary Tables

Supplementary Table S1 Expression analysis of 2752 metabolism-related genes

Unpaired analysis in TCGA CRC RNA-SeqV2		
Metabolism-related genes	Tumor/Normal	P Value
AADACL2	0.028589145	1.6718E-152
DHRS7C	0.036862612	4.8897E-144
BEST4	0.338998875	1.8126E-115
SLC17A8	0.112804332	4.5259E-114
UGP2	0.846366477	3.1507E-112
DAO	0.117164251	3.2489E-108
MTHFD1L	1.323647169	2.0728E-105
CA7	0.300199637	7.7437E-104
CHAT	0.062857939	2.5411E-103
GRIK1	0.173020316	9.8367E-103
P2RX2	0.145855112	7.2833E-99
GLTP	0.859772012	3.25053E-94
SLC25A34	0.480392628	6.33469E-93
GRIN2D	2.138943194	9.72072E-88
SLCO4A1	1.476517323	3.49923E-84
SULT1A2	0.507711332	7.2833E-84
SLC30A10	0.209712127	2.62627E-80
CNGB1	0.189339303	2.87929E-80
ENPP6	0.349667921	3.82674E-80
ASPA	0.285675887	1.16857E-79
ETFDH	0.81722966	1.92942E-77
SLC7A5	1.334708451	1.63697E-75
AMPD1	0.242074691	1.7191E-75
PLCD1	0.778060362	2.80141E-75
HPSE2	0.27186277	5.73906E-75
ABCG2	0.482309568	1.50567E-74
SLC30A8	0.176049742	7.71111E-74
PPAT	1.22307089	1.50994E-73
RETSAT	0.857224792	3.33946E-73
ADH1A	0.214394028	3.57417E-72
GCNT2	0.517935774	1.69459E-71
ACADS	0.798513134	7.06311E-71
ABCB11	0.213406149	1.19719E-70
FUCA1	0.851457124	2.48968E-70
CA2	0.590019679	1.8242E-69
SMPDL3A	0.745472032	5.3033E-69
SLC22A5	0.820718504	7.96014E-69
DHRS11	0.759993047	4.84179E-68

SLC39A10	1.233799723	8.4884E-68
HMGCLL1	0.300144859	1.54195E-67
KCTD4	0.244971127	1.63744E-67
ST8SIA3	0.163799728	2.33285E-67
SLC17A7	0.398301073	8.93522E-67
CA1	0.247758632	4.43538E-66
MGAT4C	0.129836543	1.20768E-65
HTR3E	0.183015853	1.28609E-65
FUT1	1.533479191	1.60611E-65
PPAP2A	0.813311724	2.84337E-65
SLC6A6	1.356608969	3.72975E-65
PYCR1	1.178306032	4.54977E-65
GYLTL1B	1.878499272	1.72136E-64
PRDX6	0.896493576	7.13019E-64
SLC25A23	0.862697181	2.0643E-63
SLC6A19	0.251429828	1.39535E-62
AQP8	0.316580197	1.83771E-62
TAT	0.235307863	1.22725E-61
ABCA8	0.346192582	2.14492E-60
GBA3	0.329248039	4.3284E-59
SLC25A20	0.837588945	7.24203E-59
HS3ST6	0.144905814	9.92942E-59
SCN11A	0.331667811	1.37631E-58
SLC27A6	0.271879902	1.70941E-58
PLD5	0.117770372	1.7176E-58
SLC7A14	0.224252839	1.97258E-58
SCN7A	0.294557059	1.04953E-57
UGDH	0.851405303	1.18165E-57
HSD3B2	0.216210702	1.53547E-57
ADHFE1	0.43048122	2.10212E-57
DHDDS	0.879421772	6.14616E-57
TPH1	0.268450182	6.38078E-57
GABRD	2.292163389	7.13268E-57
ENTPD5	0.764372401	1.06109E-56
HADHA	0.933032995	1.76881E-56
ATP11A	1.238225518	1.78597E-56
SLC5A7	0.207771773	3.55447E-56
CAD	1.146986914	6.02896E-56
AHCYL2	0.781800522	9.51091E-56
SLC36A1	0.831706082	9.67538E-56
GABRG1	0.085485921	1.23645E-55
SLC2A13	0.812830568	1.31038E-54
CPT2	0.87709087	1.45442E-54
ATP6V0D1	0.906295411	1.9091E-54

SLC16A12	0.349881681	3.10024E-54
SLC26A2	0.682261077	5.01804E-54
AMPD2	1.116129021	6.1163E-54
SCN9A	0.498857122	6.51762E-54
PDE6A	0.387837211	7.50675E-54
NAT10	1.115838071	1.14495E-53
SMOX	1.317086497	1.8724E-53
GABRG2	0.184510128	2.28124E-53
AGPAT9	0.703288937	3.46755E-53
GPD1L	0.870338224	4.5116E-53
B3GALT1	0.325647061	1.86901E-52
SLC4A10	0.324813002	1.88503E-52
SLC4A4	0.495076754	3.26008E-52
SULT2B1	1.627957524	7.36E-52
CA4	0.342208796	8.76676E-52
KCNE2	0.415770389	9.00805E-52
ABHD3	0.824617254	1.03173E-51
CYBASC3	0.913800147	1.03453E-51
ATP6V1G2	0.518831954	1.5578E-51
GMPS	1.10082766	2.06787E-51
ABCE1	1.106315433	2.49493E-51
SLC22A18AS	0.715772583	3.16014E-51
ATP1A2	0.37799236	4.4759E-51
HADHB	0.91795777	5.5923E-51
GRIK3	0.474785435	1.02199E-50
GSTM5	0.52836108	1.19577E-50
P2RX4	0.859446233	1.40931E-50
RDH5	0.714150938	2.47418E-50
SMPD1	0.827867217	2.86735E-50
PLCE1	0.788263311	3.14098E-50
ATP2B3	0.180613839	5.4389E-50
CLCA4	0.354818306	5.47378E-50
SQRDL	0.866761437	6.65268E-50
ACO2	0.912207191	1.00347E-49
ABCC13	0.417610219	1.06775E-49
BCHE	0.398734191	1.17396E-49
HSD11B2	0.803264551	1.20182E-49
NAALADL1	0.636765799	2.41576E-49
PYGM	0.445555909	2.45104E-49
GPT	0.605128037	3.19251E-49
SLCO4C1	0.367836899	3.22593E-49
UGT1A8	0.432181941	3.49626E-49
CHST9	0.172418128	3.82289E-49
ATIC	1.098359241	4.07356E-49

OGFOD1	1.078021885	5.61054E-49
ST6GALNAC6	0.766315239	5.61413E-49
IMPDH1	1.15833744	5.86272E-49
SUCLG2	0.899041921	9.26111E-49
ACOX1	0.884698586	9.48522E-49
ABHD5	0.884450695	1.16404E-48
OSTBETA	0.505578654	2.28208E-48
SLC7A11	1.402427048	2.84952E-48
CNGA3	0.33331424	1.73674E-47
SCD	1.240428442	1.94733E-47
ASPG	0.323790813	1.99243E-47
GLRA4	0.282315739	2.3702E-47
TTYH3	1.142161224	2.46953E-47
GRIN2A	0.315909422	2.92797E-47
KLB	0.568504247	3.00035E-47
ATP8B1	0.875606597	3.40315E-47
RHAG	0.050178988	3.71004E-47
MGST3	0.909241019	4.34671E-47
ADH1B	0.419452586	4.57268E-47
SLC12A8	1.165916988	4.91961E-47
PAICS	1.121289564	4.97392E-47
HSD17B2	0.626117497	5.85486E-47
SULT1A1	0.764083436	6.40492E-47
GLS2	1.757024105	1.6096E-46
RYR3	0.563194818	3.75142E-46
NME1	1.163439678	3.89471E-46
GDPD2	0.488086228	4.62992E-46
CHPF	1.153402351	6.5039E-46
SLC30A4	0.735958056	6.81358E-46
STRA6	3.610325505	7.70584E-46
HSD17B11	0.893112329	7.76589E-46
GALNT6	1.235368429	7.9115E-46
FUT9	0.083432225	1.81325E-45
PGM1	0.857496448	1.96256E-45
TRPM6	0.538204423	3.91986E-45
KCNB1	0.264126945	5.23548E-45
DPEP1	2.132646144	5.46348E-45
LDHD	0.6484206	6.37185E-45
ACAA2	0.862995731	6.51568E-45
PDE8A	0.893563106	6.52046E-45
PDE2A	0.648653846	6.62482E-45
SCN2B	0.371819331	6.77769E-45
SCNN1B	0.478851846	7.28967E-45
HPGD	0.711905853	9.96449E-45

TRPV3	0.402855759	1.67116E-44
BEST2	0.335755039	2.1567E-44
ABCD3	0.903026348	4.04089E-44
DPYSL5	0.264570615	6.65412E-44
CYP27B1	1.422700418	7.1862E-44
B3GALT4	0.817737435	9.27526E-44
ABCB5	0.231435572	1.45419E-43
ABCA9	0.448944496	1.95523E-43
CPT1A	0.894103925	4.41246E-43
TST	0.87377273	6.41586E-43
SIAE	0.860865806	7.16409E-43
PTGS1	0.738221226	8.87862E-43
GRIA3	0.393663715	9.82777E-43
SLC17A4	0.567161606	2.04119E-42
B4GALNT2	0.325727462	2.09192E-42
B3GNTL1	1.280134031	2.38788E-42
CA10	0.278542713	3.11059E-42
HTR3C	0.173721867	4.1135E-42
SLC10A2	0.089203793	4.52699E-42
SDHD	0.915727689	5.65537E-42
OSTalpha	0.509795314	6.48871E-42
PPAP2B	0.857807298	2.79215E-41
SHMT2	1.125368496	2.99263E-41
INPP5A	0.909279545	3.21546E-41
MTHFD2	1.150171517	3.71433E-41
HTR3B	0.059845715	4.77216E-41
PLCL2	0.691249792	5.10237E-41
ADCY9	0.856054711	7.54233E-41
SLC9A9	0.658272923	1.30135E-40
SCP2	0.921734683	1.54119E-40
GRIA4	0.402633953	1.87718E-40
CPOX	1.10385709	1.89971E-40
SLC13A1	0.08398207	1.99991E-40
FLVCR2	0.741213815	2.3187E-40
NIT1	0.9163177	3.3471E-40
P2RX1	0.601380597	6.04752E-40
MFSD11	0.915214498	9.45E-40
SLC5A6	1.177947606	1.24423E-39
SQLE	1.192026166	1.36875E-39
AQP7	0.596399434	1.73073E-39
LTC4S	0.484301716	1.75869E-39
SLC8A3	0.429605722	2.22629E-39
CTPS	1.1394989	2.45354E-39
DGAT2	1.259502815	2.48362E-39

TRPC7	0.119121592	2.66003E-39
PPA1	1.096636075	2.98848E-39
ADH1C	0.543730472	3.02082E-39
GPT2	1.178986748	3.12267E-39
SORD	1.163452879	3.29572E-39
KCNA4	0.108885003	4.54937E-39
SLC3A2	1.097719593	4.7939E-39
ACSM5	0.342591841	5.84806E-39
ABCC8	0.335858549	7.06149E-39
AGXT2L2	0.880624697	7.80297E-39
PDE7B	0.628794681	7.95868E-39
FMO4	0.750479221	1.06309E-38
SLC25A32	1.121256816	1.42046E-38
KCTD9	0.875151154	1.56472E-38
ACSBG1	0.606711494	2.43522E-38
GDPD3	0.722878486	2.85272E-38
GART	1.092927993	3.1323E-38
SMPD4	1.068250216	3.76096E-38
ATP1B2	0.590154099	4.00586E-38
GPX3	0.751732036	4.20054E-38
NAT9	1.125579633	4.52085E-38
TKT	1.083405019	6.19808E-38
ATP5A1	0.920219129	6.47348E-38
CLIC5	0.816020543	8.55797E-38
ACAT1	0.875470795	9.90289E-38
UGT1A10	0.599891288	1.04795E-37
BTD	0.879881499	1.07602E-37
ACACB	0.805740362	1.35295E-37
PLCXD3	0.321547011	2.34586E-37
ENOPH1	1.091644817	2.66791E-37
SLC9A1	0.884923813	2.81939E-37
SLC5A11	0.314994996	3.15594E-37
SCN4B	0.647445133	3.2078E-37
KCNK3	0.454969608	3.85076E-37
MGLL	0.891935579	4.04037E-37
SLC35D1	0.890686818	4.13342E-37
ADCY5	0.535984694	5.66707E-37
ABCA6	0.474542654	7.87461E-37
B3GNT7	0.658403077	8.1939E-37
PIK3C2G	0.139620099	9.11871E-37
SLC23A1	0.546801461	1.00987E-36
SLC7A6	1.114267597	1.88342E-36
ATP13A4	0.441394434	1.92386E-36
HAGHL	1.788664074	2.27556E-36

KCNG3	0.414365793	2.4702E-36
AHCY	1.113838553	3.10349E-36
CYP4B1	0.378202499	3.83463E-36
KCNS2	0.302890945	5.15496E-36
CES2	0.840479563	5.17264E-36
SLC4A11	2.303061655	5.69906E-36
GRIA1	0.328242536	5.94941E-36
PAPSS2	0.825817626	7.43154E-36
SRM	1.115446552	7.70306E-36
GALNTL1	0.498053506	1.01536E-35
DHRS9	0.565781013	1.05269E-35
KCNJ14	1.448840893	1.20285E-35
ZADH2	0.908876891	1.55283E-35
PDE9A	0.738094667	3.12145E-35
SLC22A23	0.862444562	3.61605E-35
SLC26A3	0.564497153	4.13116E-35
GRIK5	0.493763152	4.24173E-35
FOLR2	0.641004994	6.13145E-35
TGDS	1.151243776	7.14102E-35
TCN2	0.815633786	8.12254E-35
CA14	0.50651169	8.9502E-35
SCN3A	0.539820412	9.15154E-35
PSPH	1.179954742	9.26908E-35
CHST8	0.281489291	1.24761E-34
PDE5A	0.808197963	1.39109E-34
GALNT12	0.866390349	1.45195E-34
MAOA	0.822294241	1.46245E-34
RBKS	0.820329359	2.01664E-34
SLC35F1	0.57387608	2.55629E-34
ABCC1	1.112894809	3.41047E-34
GRIA2	0.230906168	4.29876E-34
GDPD5	1.362799524	4.50281E-34
CA9	2.546888554	5.67665E-34
SLC41A2	0.822986327	9.51571E-34
SLC6A15	0.26332372	1.17927E-33
ACAA1	0.910247733	2.41542E-33
NEU4	0.662945785	2.62933E-33
SDHA	0.926444913	2.63245E-33
ALG3	1.084102401	2.70872E-33
PLCD3	0.866220848	3.76002E-33
IDH3A	0.919300076	3.8512E-33
SLCO2A1	0.789833345	3.90665E-33
LIPC	0.389933472	4.22371E-33
SLC2A4	0.695422689	4.22753E-33

FMO5	0.773541863	4.4571E-33
SLC18A3	0.237940404	5.99955E-33
NIT2	1.10652173	9.89575E-33
KCNAB1	0.713464364	9.97931E-33
SCLY	1.113240039	1.06172E-32
RRM2	1.145666373	1.08588E-32
ABHD6	0.886660528	1.14408E-32
UQCRFS1	0.922887888	1.39224E-32
ABP1	0.850066016	1.51424E-32
HMGCL	0.909531908	1.70118E-32
HTR3A	0.358595035	1.7035E-32
MGAT4A	0.894331252	2.28668E-32
CLCNKB	0.281663234	2.30432E-32
SLC32A1	0.080793107	2.76842E-32
UGT2A3	0.528909392	3.00883E-32
KCNC1	0.416452461	3.05639E-32
SLC29A1	1.121261069	3.60513E-32
ACCN2	1.300070504	3.85777E-32
HS6ST3	0.302221139	3.93548E-32
KCNA1	0.253184813	4.15402E-32
SULT1B1	0.67308679	4.19972E-32
DIRC2	0.88918365	4.4945E-32
UMPS	1.070833818	7.13406E-32
ATP2B1	0.915556226	7.44371E-32
ACADM	0.88125915	9.03695E-32
ENTPD3	0.504892717	1.04992E-31
PGM5	0.646499889	1.11607E-31
NMNAT1	0.898734324	1.28398E-31
SVOP	0.339781083	1.60192E-31
UGT1A7	0.144930027	2.30703E-31
ATP2B2	0.34956828	3.19245E-31
TPO	0.38624202	3.39445E-31
MOCS1	0.79834338	3.42284E-31
PYCRL	1.176128481	3.51207E-31
ACBD6	1.09419976	4.01905E-31
ATP6V1D	0.934424536	4.21769E-31
KCNMA1	0.652006397	4.27816E-31
NANP	1.173322533	4.46426E-31
ENTPD8	0.676848215	5.49112E-31
DPEP3	0.258331078	5.50125E-31
AKR1B10	0.60980972	6.38816E-31
MBOAT1	0.877864657	7.86463E-31
AASDHPPT	1.073628477	9.68697E-31
CHI3L1	1.698775685	9.78163E-31

AQP4	0.218129079	1.05241E-30
PDE4D	0.842761048	1.10404E-30
SCNN1G	0.347649626	1.29665E-30
LARGE	0.866579135	1.43112E-30
GDE1	0.937131952	1.5931E-30
GCNT4	0.577581027	1.6153E-30
SLC10A4	0.46795682	1.77377E-30
LPCAT3	0.909378705	1.84258E-30
KCNMB2	0.466886005	1.86205E-30
GBA2	0.923552734	2.16459E-30
HADH	0.91317072	2.51004E-30
PANK3	0.890104277	3.16708E-30
MFSD4	0.73959707	3.51806E-30
FASN	1.119024908	4.12066E-30
LRAT	0.342718338	4.80268E-30
UAP1	0.923335729	5.36349E-30
GAD1	5.224939319	5.8184E-30
CRYZL1	0.911933715	5.97281E-30
IMPA2	0.888253698	6.66151E-30
PLA2G10	0.73020416	7.59632E-30
CA12	0.801382736	8.22535E-30
GNPTAB	0.916312573	8.50186E-30
NAALAD2	0.613804259	8.57532E-30
NAT1	0.815733336	8.82398E-30
PRDX4	1.104345271	9.17755E-30
GLT25D1	1.066395822	1.11111E-29
DHCR7	1.126593678	1.15695E-29
SLC7A1	1.110245458	1.27479E-29
MOGAT2	0.579028481	1.47783E-29
ABCD2	0.528932394	1.67035E-29
GRIN3A	0.691851278	1.7483E-29
KCNA3	0.52279996	1.86617E-29
SLC44A1	0.939529113	2.21961E-29
DPM2	1.088845941	3.03954E-29
DCTD	1.059843575	4.49888E-29
KCNQ5	0.417428933	5.41717E-29
G6PC	0.15093532	5.99827E-29
B3GNT8	0.83845882	6.07793E-29
PIGS	0.924746362	7.22586E-29
NUDT5	1.089896319	8.45055E-29
KCNMB1	0.660711509	8.57266E-29
PCK1	0.716950353	8.62554E-29
SLC1A5	1.088075016	9.05664E-29
UGT1A1	0.419861769	9.17283E-29

NT5DC2	1.142908352	9.25001E-29
IMPA1	0.893471653	9.46797E-29
ATP2A3	0.835154375	9.47611E-29
UGT1A5	0.133634402	9.87784E-29
HMOX1	0.837454845	1.12265E-28
ATP6V1C2	1.331452087	1.38104E-28
GSTA1	0.459492757	1.3963E-28
SLC35A3	0.890844628	1.53035E-28
CYP11A1	0.393921136	1.54753E-28
SLC25A42	0.857352689	1.59758E-28
KCNA5	0.578279145	2.17142E-28
SFXN3	1.118798384	3.04991E-28
FPGS	1.083278597	4.07701E-28
MPI	0.898321394	4.18853E-28
NUDT16	0.924199671	4.3969E-28
CACNG7	0.207424461	5.4751E-28
UGT2B15	0.547780754	5.53027E-28
ACSF2	0.846707526	6.27014E-28
XDH	0.738461417	7.50371E-28
ACER3	0.902416284	7.61645E-28
ATP5B	0.945462075	8.22828E-28
GRIN2B	2.248539185	8.78538E-28
SLC6A9	0.840160988	8.80197E-28
PLD1	0.877770358	9.18651E-28
CAT	0.917792884	1.01376E-27
SLC25A4	0.902165607	1.08904E-27
ALAD	0.930302688	1.43952E-27
SLC23A3	0.663009771	1.5375E-27
SCN3B	0.561246901	1.58332E-27
CLCN2	0.858689193	1.69344E-27
SLC35A4	0.948415061	1.74159E-27
ADCY3	1.11169842	1.76241E-27
PECI	0.850082441	1.79397E-27
ATP1B3	0.933996686	2.21814E-27
SLC22A17	0.742423935	2.42038E-27
COX5A	0.92641595	2.76443E-27
ACSS2	0.911910711	2.93244E-27
ASNS	1.141778457	3.03258E-27
CES3	0.751383644	3.35152E-27
EPHX2	0.836882707	3.3771E-27
DHRS2	2.666196788	3.79707E-27
NOX4	2.422658648	4.03128E-27
EBPL	1.121194916	4.04666E-27
MUT	0.924407297	4.50523E-27

FECH	0.875603413	5.66884E-27
SUCLG1	0.931175262	5.97722E-27
AFMID	1.107673301	8.37939E-27
ATP6V1E2	1.261269102	8.39254E-27
AUH	0.912509105	8.6403E-27
ADH5	0.939347685	9.30787E-27
SLC18A2	0.576137338	9.59611E-27
SLC25A15	1.116290475	9.8092E-27
UGT1A9	0.455312204	9.91906E-27
MTAP	1.106766713	1.41376E-26
NPR1	0.729904946	1.61176E-26
GNPDA1	1.091309837	1.67763E-26
ATP5F1	0.936546263	1.86138E-26
B3GALT5	0.626002173	1.89223E-26
PDE3A	0.700766905	1.89568E-26
AQP11	0.812353292	1.90327E-26
SLC9A2	0.734030038	2.41292E-26
SLC4A2	1.07127896	3.56453E-26
GBA	0.928269939	4.49596E-26
PLCG2	0.762345224	4.57359E-26
CLCA1	0.514280739	6.15302E-26
SLCO1C1	0.45460399	6.23724E-26
NUDT1	1.130953837	7.32312E-26
HCN4	0.355167714	8.84335E-26
SLC25A22	1.115375146	1.01856E-25
PDE1C	0.582029438	1.09068E-25
SLC22A3	1.285532668	1.1434E-25
UCKL1	1.103317566	1.1685E-25
SMPD3	0.85811851	1.26637E-25
KCNK2	0.399356402	1.32461E-25
SLC20A1	0.907710185	1.42213E-25
ATP6V0D2	0.616960671	1.58871E-25
PIGZ	0.80620649	1.61794E-25
ATP6V1F	1.08293263	1.76838E-25
RPIA	1.09343696	1.8719E-25
DERA	0.926046201	2.31E-25
PLA2G2C	0.078158727	2.42883E-25
CYBRD1	0.812000627	2.72325E-25
ACBD7	1.424599254	2.95809E-25
MAN1A1	0.862875219	2.9947E-25
LPCAT4	0.909562765	3.85544E-25
CP	0.528477686	4.88616E-25
KCNN3	0.646386301	5.52197E-25
SLC44A4	0.880071999	6.9484E-25

SLC35B2	1.059928843	1.20339E-24
UQCRC2	0.944928977	1.228E-24
COMT	1.076285489	1.23901E-24
KCNB2	0.372966824	1.30519E-24
NAT15	0.951481359	1.37241E-24
CYP2S1	1.125810975	1.62701E-24
OGDH	0.947854328	1.70833E-24
AOC3	0.769980434	1.7421E-24
GCNT3	0.75620957	1.82248E-24
MGAM	0.552488104	1.96698E-24
INPP5K	0.915689068	2.52378E-24
CYP21A2	0.590639838	2.57248E-24
DTYMK	1.107042814	3.01931E-24
GALK1	1.123838401	3.08613E-24
SI	0.499522662	3.1667E-24
ETFA	0.929007334	3.21437E-24
ACOT2	0.883188254	3.2291E-24
MCOLN1	0.915416589	3.26572E-24
KCTD8	0.257769322	3.7458E-24
SLC4A5	1.164211592	3.82733E-24
HSD17B7	1.126725951	4.24459E-24
NSDHL	1.101389651	4.70088E-24
CLCA2	0.374751353	5.10482E-24
AK5	0.514581491	5.11862E-24
ITPKA	0.785527432	5.35133E-24
KCNH8	1.570246225	5.74276E-24
ABCC10	1.080029082	6.44194E-24
ATP5J	0.930699449	7.78355E-24
TRPM4	0.896834903	8.18779E-24
SLC12A2	1.109173051	8.71662E-24
DPYD	0.733699019	9.12707E-24
PIP5K1B	0.890703275	9.14338E-24
MSRA	0.869468507	9.90903E-24
PLCD4	0.811481408	1.12251E-23
HYAL3	1.21005303	1.42297E-23
ST8SIA1	0.669689107	1.46157E-23
KCNK10	0.63344413	1.69419E-23
SDHB	0.938360121	1.83534E-23
DHRS1	0.893013763	1.90578E-23
MTHFD1	1.070694184	2.51898E-23
SLC35B3	0.929662307	2.7859E-23
ADCY2	0.606714315	2.81248E-23
PIK3CG	0.680769961	2.82162E-23
ARSA	0.897986249	3.23958E-23

GSTM2	0.768909825	3.356E-23
SLC24A4	0.55792204	3.41064E-23
PNLIPRP3	0.072484932	3.48795E-23
CHRNA1	0.636601874	3.57635E-23
STAR	0.641925538	3.80612E-23
UQCRC1	0.942690593	4.5091E-23
SLC19A2	1.119112133	4.65667E-23
EHHADH	0.885652725	4.90948E-23
ACLY	1.059343649	5.11055E-23
SLC6A16	0.507655944	5.71302E-23
CHRNA3	0.608213757	5.77971E-23
KCNG1	0.57423118	5.85253E-23
ABCA5	0.84786395	5.99312E-23
DSEL	0.729302178	6.44457E-23
PYCR2	1.057970832	6.75971E-23
ST6GALNAC3	0.731428339	6.90904E-23
CACNB2	0.738644175	7.0679E-23
CHST5	0.627399868	8.50162E-23
LIPH	0.865972875	8.89479E-23
NANS	0.925152559	9.46936E-23
ACAD8	0.939207317	1.02518E-22
NDUFA6	0.931499642	1.09696E-22
ATP8B4	0.77404346	1.14119E-22
GCK	0.555860297	1.14701E-22
SLC16A9	0.735662439	1.24746E-22
DGKZ	1.06989652	1.33117E-22
EXTL1	0.462404691	1.48869E-22
SLC25A11	0.935082313	1.64759E-22
ACOT11	0.895083905	1.69337E-22
HYAL2	1.063339911	1.91634E-22
BDH1	0.90714667	2.53636E-22
KCTD14	1.149726011	2.68618E-22
CACNB1	0.848002439	3.02987E-22
ABCA10	0.614665661	3.05909E-22
CKB	0.813309179	3.26491E-22
CHRNB4	0.492372575	3.39066E-22
ADCY6	0.929470626	3.63821E-22
SLC9A3	0.622863583	3.66174E-22
SLC11A2	1.097636821	3.6799E-22
B3GNT6	0.505363938	3.95094E-22
NOS1	0.334144322	4.64765E-22
COX7A1	0.766923904	4.73013E-22
ALDH4A1	1.184946619	4.84477E-22
CLIC2	0.796104498	4.8537E-22

KCNT2	0.561017465	5.28959E-22
NAT6	1.117688038	5.37087E-22
GLA	1.102696645	6.38469E-22
SLC17A5	0.912933305	6.9742E-22
CROT	0.864530702	7.21234E-22
ACADVL	0.934378217	7.49923E-22
SLC15A2	0.815621115	7.59525E-22
SLC31A2	0.861946995	7.62674E-22
CYP3A4	0.470340446	7.89901E-22
GALNTL2	0.672529791	8.24978E-22
ATP5G3	0.944252294	8.31696E-22
SLC46A3	0.869862264	8.49077E-22
COQ9	0.945840242	1.16131E-21
BHMT2	0.570478828	1.22478E-21
GOT1	0.924908768	1.22774E-21
SGPP1	0.892254285	1.30125E-21
AKR1C2	0.739868914	1.37143E-21
ATP2B4	0.865598914	1.48037E-21
SMS	1.071965433	1.52137E-21
BPNT1	0.925412794	1.58093E-21
HPRT1	1.088452993	1.88032E-21
DPEP2	0.658829352	1.88158E-21
COX19	1.083574232	3.06561E-21
NME2	1.059658631	3.09416E-21
NDUFS2	0.951679873	3.13028E-21
ALDH6A1	0.871851061	3.48257E-21
MFNG	0.844779293	3.48902E-21
SLC44A2	0.948147301	3.66041E-21
ATP8A1	0.857864841	4.23854E-21
PC	0.875810534	5.2355E-21
CKMT1B	0.893132558	5.29044E-21
CMBL	0.824975781	5.89073E-21
PTDSS1	1.067566003	6.31598E-21
NDUFB1	0.923587841	6.65898E-21
HVCN1	0.797324349	6.90132E-21
ST3GAL3	0.852600505	7.53441E-21
ALG8	1.07641285	7.93165E-21
ADO	1.051655154	8.24255E-21
HLCS	1.073182547	8.33108E-21
PDE7A	1.108343844	9.26391E-21
LASS1	0.510583371	1.02985E-20
ABCF2	1.049867637	1.27894E-20
SLC13A2	0.576119866	1.2872E-20
GABRA1	0.139888096	1.29984E-20

COX8A	0.939953669	1.30935E-20
ACADSB	0.914346569	1.39128E-20
SV2B	0.593704045	1.53244E-20
PHYHD1	0.673579035	1.57676E-20
KL	0.66189323	1.65115E-20
SCN4A	0.500277536	1.71769E-20
GLOD5	0.735664634	1.91745E-20
GSTM4	0.893943286	1.99175E-20
MAOB	0.69935249	2.14737E-20
UST	0.6926063	2.19738E-20
NAGA	0.942333252	2.22219E-20
ATP8A2	0.544394028	2.54016E-20
WBSCR17	0.647807417	2.58119E-20
ABCD4	0.928952034	2.64181E-20
MSRB3	0.786885929	2.67791E-20
GALM	0.922576614	2.83706E-20
SLC20A2	0.91670288	3.18554E-20
PHYH	0.90116719	3.48944E-20
DOLPP1	0.906847712	3.86072E-20
SLC1A1	0.799512278	3.92111E-20
PRPS1	1.093265162	3.93068E-20
AOX1	0.653526596	4.2127E-20
MFSD7	0.757931698	4.51999E-20
SRD5A1	1.097478523	4.96669E-20
GABRE	1.316861562	5.4826E-20
LIPG	1.158298578	5.64736E-20
IMPDH2	1.062423119	5.79518E-20
PLCG1	1.097758782	6.61131E-20
CCBL1	1.183972624	7.01972E-20
NDUFS1	0.948587222	7.54804E-20
SLC25A14	1.122317666	7.61841E-20
ELOVL4	0.683655218	8.45314E-20
PGAM5	1.082503492	8.88014E-20
ODC1	1.079853252	9.44407E-20
SLC35C2	1.067658854	9.48702E-20
SLC22A4	0.768064941	9.60924E-20
GPD2	0.949011568	9.82477E-20
SCN1B	0.772815727	9.85984E-20
GLRX3	1.071478763	1.03892E-19
ITPR1	0.834350767	1.17702E-19
NAT2	0.728615022	1.23866E-19
CLCN5	1.130048513	1.29608E-19
TCN1	4.510952001	1.59055E-19
SLC12A9	1.072835019	1.61998E-19

SLC19A1	1.103462482	1.74511E-19
EXT1	0.941153487	2.18074E-19
B3GALT6	1.081518989	2.45583E-19
SYNJ1	0.906029268	2.57087E-19
PMM1	0.908720439	2.58046E-19
PDXP	0.912149504	2.74236E-19
HIBCH	0.931000019	2.8745E-19
C1GALT1	1.080540236	2.93057E-19
ABHD8	0.857314011	3.05649E-19
MOSC2	0.842867366	3.17779E-19
SLCO1B3	4.642489454	3.6584E-19
ATP9B	0.916574052	3.75334E-19
UGCG	0.889497641	3.98637E-19
MFSD10	1.066361134	4.53824E-19
GABRB3	0.488885521	4.8456E-19
SLC38A7	1.086323799	5.16423E-19
SLC25A29	1.127933921	5.48681E-19
ALAS2	0.333214061	5.86538E-19
GALNT13	0.419362381	6.39465E-19
ACOT1	0.893458722	7.25946E-19
CACNA1A	0.637726172	7.45197E-19
MGAT5B	0.513397624	8.52967E-19
SLC27A4	0.936539745	1.03219E-18
RYR1	0.590921323	1.06201E-18
DHODH	1.099695698	1.09203E-18
SLC29A2	1.105849801	1.14164E-18
KCTD12	0.840899738	1.20777E-18
ME2	0.905643055	1.43391E-18
SLC5A10	0.688533858	1.46339E-18
KCNH1	0.693975142	1.77737E-18
HS2ST1	1.060881732	1.98462E-18
HMGCS2	0.732697729	2.35811E-18
SLC8A1	0.840145111	2.47647E-18
ENOX2	1.099267068	2.50673E-18
EPHX1	0.895115501	2.81695E-18
CYP4F12	0.819404087	2.99256E-18
ECH1	0.918248109	3.51894E-18
GLO1	1.063517239	3.88592E-18
FRRS1	0.787397663	3.96412E-18
AGL	0.918239815	4.16554E-18
DPM1	1.093826686	4.66596E-18
NUDT22	0.935678425	5.08598E-18
ENPP2	0.800043757	5.21691E-18
HYI	0.903556717	5.3645E-18

ACOT9	1.071710613	5.58634E-18
PDE1A	0.713901307	5.64553E-18
B3GNT1	0.937275198	6.3928E-18
B3GNT5	0.926715049	6.84048E-18
PCK2	0.928731274	7.08566E-18
SLC25A24	0.933590178	7.47156E-18
SLC2A5	0.753861298	7.68511E-18
ATP2C1	1.034751394	8.42157E-18
HK2	0.914266998	8.69524E-18
AKR1C1	0.792127594	8.78165E-18
PLA2G5	0.618631562	9.08183E-18
SLC6A20	1.56286047	1.08293E-17
HDC	0.626044989	1.10986E-17
ASNSD1	1.049644694	1.31196E-17
MDH1	0.958213254	1.3272E-17
SMPDL3B	0.897653898	1.42339E-17
G6PC3	1.0611864	1.50573E-17
KCTD2	0.947754488	1.57569E-17
NADK	0.953541689	2.12412E-17
PDE6B	0.64216344	2.2297E-17
MTHFR	0.920560112	2.29227E-17
NAT8B	0.489591179	2.5056E-17
TREH	0.575485065	2.63288E-17
CYP4V2	0.914319787	2.69232E-17
SLC47A1	0.685323469	2.73319E-17
MGAT2	0.949504519	2.78803E-17
GFOD2	0.954888489	2.95418E-17
SLC35E4	1.156869874	3.66252E-17
PDE6G	0.634303295	3.90781E-17
TPMT	0.936190265	4.0084E-17
ACSL6	2.081573452	4.01252E-17
SULT4A1	0.44582771	4.62212E-17
UGT3A2	0.408634978	4.95265E-17
SLC37A2	0.758010014	5.31243E-17
SLC35A5	0.943752943	5.74959E-17
SLC35A1	0.92084092	6.98256E-17
GRID2	0.154340281	7.10235E-17
SLC22A11	3.322162164	7.19892E-17
HPSE	0.821556403	7.71711E-17
CNGA1	0.726455254	8.09146E-17
PNPLA7	0.797709125	8.66227E-17
NUDT10	0.594461957	8.71441E-17
CRAT	0.888594114	9.53944E-17
PTGES3	1.036753727	9.79903E-17

KCNJ16	0.431044559	9.99155E-17
PPAPDC3	0.745496159	1.00828E-16
TH	2.95807873	1.03648E-16
GGT6	0.80667206	1.05738E-16
PCBD1	1.058006314	1.06393E-16
SLC39A6	1.088191866	1.07985E-16
NUDT3	1.059868532	1.08201E-16
FLAD1	1.056075001	1.11837E-16
CDO1	0.558388493	1.13762E-16
PAH	6.38152665	1.20027E-16
ACACA	1.075872721	1.20901E-16
KCNJ11	1.223283637	1.32145E-16
COQ10B	0.949925193	1.34645E-16
GSTZ1	0.887963978	1.36075E-16
SLC25A12	0.936302282	1.39425E-16
THTPA	0.92554696	1.48487E-16
PDE1B	0.719707773	1.50587E-16
TRPC6	0.748231395	1.55877E-16
DECR1	0.944112251	1.5646E-16
CACNA2D1	0.57525882	1.77163E-16
ATP5O	0.949236652	1.7853E-16
ST3GAL2	1.090836595	1.83739E-16
PIGU	1.085481847	2.11713E-16
MICAL2	0.940965992	2.31499E-16
SLC35F3	0.415312408	2.34867E-16
SLC10A1	0.385183648	2.42733E-16
CHRNA7	0.478373423	2.48125E-16
PTEN	0.939490004	2.69733E-16
GPI	1.043662938	2.82016E-16
CECR1	0.827386937	2.89849E-16
PFAS	1.081114515	2.92177E-16
KCNA6	0.652936825	3.35862E-16
MBOAT4	0.582911588	3.36521E-16
ADK	1.093029173	3.43177E-16
SLC25A17	1.058467122	3.89314E-16
OTC	0.538816708	3.94248E-16
TSTA3	1.088744295	4.00726E-16
ELOVL6	0.903035885	4.09731E-16
SLC2A1	1.124312886	4.21479E-16
MGAT1	0.95355592	4.33122E-16
IP6K3	0.613538727	4.4983E-16
SLC4A1	0.278410626	4.71766E-16
THNSL1	1.093213027	5.1434E-16
SLC11A1	1.399999536	5.39011E-16

FOXRED2	1.1116854	5.42033E-16
DCI	0.935130175	5.44708E-16
PLD4	0.654789264	5.74837E-16
CTPS2	1.080816883	6.08917E-16
PTGDS	0.775301255	6.20712E-16
SLC8A2	0.556996505	6.36382E-16
NDUFS4	0.938138106	7.02738E-16
SLC25A19	1.079778887	7.09479E-16
PNPO	1.064997903	7.21959E-16
ST6GALNAC1	0.817910582	7.86615E-16
BST1	0.788629071	8.26688E-16
PRDX1	1.042788323	8.72766E-16
NDUFV1	0.954912259	8.96638E-16
SLC35D2	0.939444624	9.82897E-16
COX10	0.941284373	1.07222E-15
SEPHS2	0.940564202	1.07873E-15
MCOLN2	0.760234064	1.18776E-15
AKR1C4	19.69225343	1.20213E-15
AKR7A2	0.938658436	1.24348E-15
ST8SIA6	0.534863843	1.25654E-15
FMO2	0.556851546	1.31191E-15
GRIK2	0.564997393	1.56732E-15
NDUFB10	0.948487947	1.73361E-15
PECR	0.890154931	1.84189E-15
NDUFB8	0.950849997	1.89638E-15
ADH6	0.726530867	2.03429E-15
ATP13A3	1.047050188	2.21512E-15
CYP26B1	0.748094299	2.36424E-15
DPAGT1	1.049133808	2.5088E-15
PTGR1	0.916573272	3.50027E-15
RDH16	2.086581436	3.57162E-15
NDST1	0.93539027	3.83495E-15
NNT	0.934180612	3.96166E-15
KCNK12	0.541523184	4.41681E-15
CACNA1H	0.827869893	4.43705E-15
SPTLC3	0.764128841	4.645E-15
PKM2	1.051592009	4.69756E-15
CACNA1D	1.203725705	4.70186E-15
STARD5	0.891468055	5.01724E-15
PIGW	1.089368152	6.18193E-15
ST8SIA4	0.841142003	6.54187E-15
VDAC2	0.953882481	6.65086E-15
ALDH3A2	0.932075781	6.66764E-15
SLC28A2	0.486723754	6.77657E-15

UPP1	0.903724945	6.89941E-15
PLCL1	0.810195794	7.1228E-15
PNLIPRP2	0.55680719	7.36062E-15
CACNA1B	0.440360379	7.88967E-15
CDS1	0.932015048	8.9362E-15
UEVLD	0.93425057	9.4605E-15
HGD	0.787784673	1.00679E-14
CANT1	0.946968424	1.01984E-14
GPLD1	0.677456963	1.05212E-14
CEL	1.861121116	1.07049E-14
SLC47A2	0.521385142	1.07929E-14
LPGAT1	1.06530152	1.16407E-14
MLYCD	0.946240028	1.21901E-14
TTYH1	0.628627479	1.25741E-14
HSD17B10	1.061315532	1.28009E-14
ACOT4	0.841078015	1.33275E-14
UXS1	1.042520122	1.37116E-14
RPE	1.051769479	1.47662E-14
GFOD1	0.844031881	1.48375E-14
TRPA1	0.711386379	1.53183E-14
AGK	1.069916907	1.57496E-14
SLC10A5	0.773774588	1.73019E-14
SLC6A8	0.872727923	1.87483E-14
PYROXD1	0.934000442	1.87539E-14
PIGN	0.934138163	2.08063E-14
BDH2	0.902743475	2.16065E-14
COX7A2	0.950793888	2.20227E-14
ACYP1	1.123416066	2.28186E-14
CLCN7	1.054766288	2.55145E-14
MGAT4B	0.955113379	2.60105E-14
PPAPDC1A	2.083051723	2.69683E-14
DIO2	1.286886739	3.14846E-14
AMT	0.800854032	3.55069E-14
ACBD4	0.917920562	3.66462E-14
NDUFC1	0.943666796	3.73237E-14
ENO1	1.046961844	3.82476E-14
PAOX	0.879648355	4.45474E-14
GNPNAT1	1.065115708	4.63578E-14
ITPKB	0.924252076	4.77417E-14
SLC9A5	1.374259816	4.90275E-14
PANK4	0.951922102	4.97049E-14
KCTD10	0.954460247	5.03556E-14
KCNE3	1.094970278	5.03873E-14
ALDH3B2	1.966004011	5.27563E-14

SLC38A4	0.659395067	5.90496E-14
GSTCD	1.076471837	6.60393E-14
GALNT7	0.93708461	6.91171E-14
LPCAT1	1.100143027	7.14174E-14
NUDT21	1.036740727	7.17791E-14
FAAH2	1.112637002	7.70122E-14
MOCS3	1.098359039	7.76185E-14
SLCO5A1	1.426786779	7.91729E-14
CACNA2D3	0.591217618	8.20284E-14
B3GALNT2	1.05011875	8.35711E-14
TYRP1	0.637259159	8.52955E-14
SLC35D3	1.962733709	8.77338E-14
RFK	0.933791046	8.79509E-14
PIP5K1C	0.947243294	9.11045E-14
GLYATL1	2.016618894	9.60755E-14
NUDT9	0.953379564	1.03181E-13
ALAS1	0.949987717	1.03431E-13
NDUFV2	0.940480229	1.08014E-13
CLIC1	1.034315913	1.13931E-13
CLCNKA	0.450622289	1.14464E-13
CYB5A	0.932675504	1.16828E-13
TPK1	0.879816897	1.17913E-13
ALG11	1.234717014	1.20258E-13
PDE4C	0.777194326	1.32712E-13
HSD17B6	0.782817183	1.35938E-13
SLC41A3	1.050558742	1.40008E-13
COX7C	0.954540971	1.46321E-13
DGKA	0.893441543	1.46854E-13
PIK3R5	0.801022634	1.52202E-13
ETNK1	0.938455799	1.52353E-13
MFSD1	0.947032332	1.53727E-13
LPO	11.73258787	1.63E-13
CYP2C18	0.712994162	1.82695E-13
INPP5E	1.071522908	1.83215E-13
VKORC1L1	1.040578804	2.00472E-13
GMPPB	0.935492794	2.25079E-13
SLC24A6	0.945553502	2.32762E-13
SLC9A7	1.587492708	2.40911E-13
CLC	0.462228118	2.51738E-13
TTYH2	0.831560954	2.52684E-13
AKR7A3	0.884690719	2.58962E-13
MAN1C1	0.822351782	2.79546E-13
SFXN1	0.938697346	2.82522E-13
MAT2B	0.957905598	2.86893E-13

CERK	0.91313326	2.90765E-13
ACHE	0.805030982	3.01701E-13
SLC16A7	0.634589896	3.13839E-13
GSTP1	1.061366901	3.14008E-13
DGKB	0.513539462	3.49079E-13
PI4KA	0.951271565	3.49529E-13
PIK3C3	0.942813086	3.81571E-13
CHST7	0.848251434	3.83876E-13
ENPP1	0.848392791	4.01532E-13
ABCC3	0.928248315	4.11143E-13
ACADL	0.339421665	4.24998E-13
ABCB1	0.800476452	4.25222E-13
NDUFS7	0.945181033	4.64172E-13
CTBS	0.941374025	4.64304E-13
NDUFB3	0.954840064	4.84604E-13
P2RX6	0.605747534	4.89516E-13
LDHAL6B	0.588931225	5.07154E-13
GAPDHS	2.008627966	5.07605E-13
PRPS2	1.057654651	5.10719E-13
ALG12	0.954110954	5.2576E-13
PLA2G2D	0.603199139	5.27189E-13
PIK3CD	0.84162197	5.5632E-13
PPCS	0.958223007	5.61171E-13
ACSM1	0.64295381	5.90488E-13
ATP13A1	1.036319965	5.98158E-13
ABCA1	0.885708111	6.4956E-13
PPAP2C	1.070695789	6.74714E-13
CHRNA5	1.149259569	6.94968E-13
UGT2B11	0.271178341	7.06284E-13
COQ7	0.960057323	7.42801E-13
SLC43A1	1.116093919	7.91107E-13
SGPP2	0.892206505	8.20374E-13
MFSD5	0.963133742	8.22101E-13
FH	0.957038583	8.78177E-13
ACOX3	0.948125863	9.03284E-13
PTGIS	0.7354546	9.04927E-13
GSTA2	0.42651606	9.43394E-13
KCNF1	0.624491377	9.58258E-13
SLC6A2	0.305381263	9.94329E-13
SLC26A7	0.607226142	9.97819E-13
ATP8B2	0.852659979	1.04366E-12
SLC1A2	0.650205818	1.16519E-12
COX5B	0.948290277	1.18769E-12
INPP5J	0.889086183	1.20505E-12

ACSL4	1.088386493	1.24146E-12
C9orf103	0.895360748	1.34651E-12
COX4I1	0.959559321	1.44758E-12
GCSH	1.353787449	1.47142E-12
ECHS1	0.950873296	1.48214E-12
RRM1	1.042683663	1.67609E-12
ECHDC1	0.953156592	1.70628E-12
TF	0.526565769	1.7285E-12
CYP27A1	0.884658103	1.82945E-12
MAN2B1	0.950381662	1.86774E-12
NDUFS3	0.958519546	2.03383E-12
OASL	0.845033944	2.24367E-12
HMBS	1.060848633	2.37873E-12
SLC29A3	1.071059036	2.42934E-12
PGM3	1.061117921	2.52846E-12
B4GALNT3	0.905328872	2.53153E-12
NUDT11	0.722525933	2.88705E-12
ABCB6	1.091839882	2.91093E-12
DHRS4	0.934951173	3.01947E-12
ABCC2	1.800453208	3.16639E-12
COX6A1	0.955461927	3.21262E-12
CHI3L2	0.610318308	3.3302E-12
FXN	1.074334243	3.39266E-12
SULT1C2	1.451238469	3.52643E-12
CDA	0.824025062	3.91232E-12
SRXN1	1.070225643	3.93204E-12
AGPAT3	0.964658381	4.23513E-12
GAL3ST3	0.211123613	4.35651E-12
G6PD	1.055597457	4.45939E-12
NDUFAF2	1.084221052	4.65037E-12
NDUFA4L2	1.218344765	4.76843E-12
GAMT	0.784715649	5.01221E-12
KCNN4	1.138942456	5.04367E-12
STARD8	0.845720762	5.08522E-12
CYP8B1	0.435219021	5.29549E-12
POMGNT1	1.047476744	5.36544E-12
AQP1	0.886735967	5.73216E-12
SLC7A3	0.419151896	6.59222E-12
ALDH1A1	0.841538737	6.77284E-12
SLC15A1	0.624494616	6.87672E-12
ALDH9A1	0.964719347	7.07788E-12
DHRS4L2	0.921782509	7.916E-12
PMM2	1.043482112	9.27637E-12
DMGDH	0.613927289	9.53582E-12

NQO2	1.096481549	9.90651E-12
ECHDC2	0.922845682	1.00502E-11
NDUFA2	0.945426257	1.05762E-11
SLC1A4	1.067111305	1.07613E-11
LBR	1.046647391	1.07735E-11
PNLIPRP1	0.344798042	1.12394E-11
BAAT	3.919894795	1.12937E-11
KCNQ4	0.690802848	1.15934E-11
CHID1	1.040898115	1.19812E-11
SLC41A1	1.090675499	1.25884E-11
NFS1	1.060587539	1.30231E-11
BLVRB	0.93636826	1.42152E-11
UROD	0.955025944	1.44181E-11
SDSL	0.919811745	1.4758E-11
CYB5D2	0.922049513	1.53396E-11
DHRS4L1	0.769491581	1.57342E-11
SLC39A5	0.838009461	1.68293E-11
SLC22A18	0.918104676	1.68977E-11
SLC5A8	0.318657491	1.70303E-11
GNE	0.877807976	1.84815E-11
GLRX5	0.957738953	1.93986E-11
PIGL	1.124248257	1.99123E-11
SEPHS1	1.038655215	2.09045E-11
PIK3R2	1.039852431	2.13553E-11
SLC25A39	1.044436669	2.17121E-11
PIGX	1.047511356	2.17438E-11
P2RX7	0.729683379	2.21307E-11
VDAC1	1.035473597	2.21651E-11
SLC25A3	0.970931637	2.33826E-11
SLC39A12	0.288053337	2.43738E-11
MAN2B2	0.957636396	2.46911E-11
IDI2	1.096249503	2.54255E-11
INPP5D	1.11032854	2.58232E-11
KCTD13	1.073055566	2.61706E-11
SLC25A35	0.913007184	2.63445E-11
TALDO1	1.036773335	2.64432E-11
GRID1	0.759225854	2.71575E-11
TM7SF2	1.095257342	2.73521E-11
COX6B2	0.744516816	2.7552E-11
GSR	0.931587336	2.88292E-11
THEM4	1.077305928	3.00795E-11
CACNG6	0.324136778	3.07595E-11
A1CF	0.832891894	3.08947E-11
ADSSL1	0.757870593	3.15733E-11

MICAL3	0.950116616	3.27778E-11
NUDT19	1.057676148	3.86305E-11
ABHD4	0.929078211	3.94378E-11
CYB5R3	0.962481073	4.04163E-11
SULT1A3	0.928397735	4.33205E-11
SLC2A12	1.19292017	4.51861E-11
MAN2A1	0.923066018	4.61939E-11
B4GALT7	1.050678978	4.69039E-11
NDST4	0.250852887	4.72045E-11
NDUFA1	0.954185163	4.78125E-11
AKR1C3	0.883729144	4.88252E-11
NMRAL1	1.062440622	5.08482E-11
NDUFA10	0.967088398	5.25261E-11
NUDT6	0.874034045	5.25461E-11
KCNJ9	0.469304022	5.25626E-11
IP6K1	0.968129796	5.36729E-11
CHSY3	1.302542459	5.96143E-11
NDUFA5	0.958889842	6.34718E-11
XYLT1	0.922629823	6.37309E-11
SLC46A1	0.906303667	6.41711E-11
SLC6A1	1.416668842	6.46302E-11
PLD6	1.120287307	6.48315E-11
ABCG1	0.914241339	6.57025E-11
TPCN1	0.958288409	6.97525E-11
SLC34A3	2.089819865	7.04577E-11
IPMK	0.881176035	7.05131E-11
CLCN4	1.232961032	7.35775E-11
CHST6	0.780782137	7.42293E-11
MFSD9	0.94671885	7.61909E-11
KCNS3	0.856360239	7.69243E-11
AK3	0.960234012	7.77406E-11
NUDT14	1.073694954	8.51132E-11
CLYBL	0.896129022	8.9911E-11
PNPLA3	2.795614329	8.99166E-11
ITPA	1.065715458	9.09497E-11
B4GALT2	1.041806572	9.33306E-11
KCND3	0.660091871	9.42501E-11
KCNK5	0.919274709	9.49483E-11
AQP6	1.876340076	9.53596E-11
STARD10	0.948720184	9.96607E-11
AGPAT6	1.04669226	1.00543E-10
MME	1.334341615	1.05859E-10
FDX1	0.954229611	1.06624E-10
INPP4A	0.949480716	1.10003E-10

UCK1	0.95907882	1.1548E-10
HPD	0.450398012	1.1717E-10
SLC25A46	0.94925731	1.18939E-10
DBT	0.954517741	1.19711E-10
ALG5	1.057818251	1.25411E-10
ASAH1	0.951402527	1.26695E-10
KCNJ12	0.683173378	1.30453E-10
CRYL1	0.928330983	1.31658E-10
FIG4	0.955719995	1.3242E-10
PNPLA2	0.949761496	1.37287E-10
ATP6V1E1	0.9697639	1.37686E-10
ABO	0.863002387	1.44152E-10
ALOXE3	3.495099222	1.46899E-10
CMAS	0.956200177	1.48386E-10
CHRNB2	0.629214824	1.51924E-10
ITPK1	0.960738129	1.54211E-10
SLC14A2	0.442526501	1.54328E-10
DLD	0.965906063	1.59573E-10
GRIK4	0.51993841	1.63861E-10
KCNK9	2.238521608	1.68558E-10
SLC12A6	0.94258906	1.70973E-10
SLC5A12	0.554019311	1.71318E-10
TRPC1	0.804792918	1.89556E-10
NPR2	0.894027622	1.98378E-10
SLC7A2	0.768046468	2.01029E-10
PDE3B	0.923309504	2.14322E-10
ATP1A3	0.745400089	2.27358E-10
AKR1B1	0.882347659	2.28222E-10
SLC25A5	0.95791382	2.34123E-10
SLC30A1	0.941945579	2.4374E-10
CHRNA4	0.233191658	2.61499E-10
PLA2G4E	2.278960591	2.64288E-10
ST3GAL4	0.84694295	2.66502E-10
B4GALT3	1.034972071	2.71404E-10
ALG10	1.076641963	2.77159E-10
SLC13A3	1.820418635	2.91162E-10
B4GALT4	0.957247361	3.04081E-10
PIGM	1.048483942	3.32181E-10
GLUD2	0.943936136	3.33334E-10
TRPV4	1.344139518	3.63437E-10
HKDC1	1.114427772	3.72509E-10
SLC25A27	1.214379258	3.8378E-10
ALOX12	0.725952862	3.86323E-10
PEMT	1.068308606	3.90784E-10

COQ4	0.951241826	3.9464E-10
SLC39A13	1.050996157	4.01902E-10
SLC29A4	0.81354424	4.19399E-10
SV2A	0.83099107	4.37552E-10
CHST2	0.868370792	4.45757E-10
FLVCR1	1.085888016	4.75476E-10
FAR2	0.9404002	4.81931E-10
GAPDH	1.032470705	5.10564E-10
RDH10	1.058604111	5.83316E-10
ALG1	1.040307129	6.01915E-10
GLOD4	0.962549942	6.21778E-10
CYB5B	1.051437065	6.374E-10
KCNH4	1.846636637	6.50913E-10
SLCO2B1	0.923149808	6.72186E-10
AADACL4	0.223231562	6.78776E-10
ATP13A5	0.23539433	7.02101E-10
NDUFB7	0.954720971	7.27788E-10
AGPAT1	0.970436714	7.63392E-10
GPX2	1.075263525	8.15698E-10
B4GALT1	0.959967926	8.60687E-10
SLC26A4	0.71573815	8.87886E-10
HSD17B8	0.923970182	9.34336E-10
PISD	1.039502898	9.35679E-10
NUDT16L1	0.954871958	1.01577E-09
KCNV1	0.569731135	1.03528E-09
LASS5	1.03780979	1.04229E-09
CNDP2	0.956851208	1.0705E-09
TK1	1.071677945	1.07308E-09
NT5C3L	1.085131062	1.12681E-09
SAT2	0.945979116	1.13603E-09
CLIC6	0.793142833	1.18974E-09
FAHD1	0.953386113	1.21644E-09
LDHA	1.035726343	1.30065E-09
PFKFB2	0.933400272	1.30317E-09
SLC15A4	1.045779935	1.40995E-09
CYP39A1	1.168186112	1.50848E-09
ACCN5	0.199798506	1.59828E-09
ENO3	1.201271601	1.60092E-09
CLCN1	2.133075785	1.67412E-09
SULF1	1.158821331	1.73352E-09
KCNN1	0.590855741	1.75853E-09
HS3ST5	0.507013548	1.8378E-09
ENTPD4	0.933729929	2.13006E-09
ISCA1	0.961912354	2.22212E-09

STS	0.911738879	2.29845E-09
GALNT1	0.954409562	2.35806E-09
OAS3	1.080912286	2.365E-09
ACSM3	0.883867292	2.38304E-09
SLC22A15	1.193453017	2.50854E-09
OGDHL	0.657989969	2.5137E-09
SLC25A44	0.9653812	2.62843E-09
ATP6V0A1	0.964575593	2.63887E-09
GLUD1	0.972212051	2.71448E-09
GABRB2	0.625316432	2.71723E-09
ENPP4	0.950042421	2.77936E-09
SUOX	0.954288307	2.79571E-09
HAGH	0.961967401	2.8994E-09
B3GALT2	0.742368285	2.98214E-09
SLC3A1	0.811801426	3.04024E-09
GPX1	1.039655612	3.22125E-09
RDH11	1.049941261	3.29631E-09
ELOVL5	1.09556095	3.49015E-09
KCTD18	0.962327903	3.56466E-09
NOS3	1.11620377	3.78224E-09
DHRS7	0.95538572	3.85155E-09
SLC25A18	0.790610426	3.96665E-09
TRPM5	0.732069393	4.06898E-09
ADC	0.877636131	4.1781E-09
KCNA2	0.569607706	4.19275E-09
GABRA2	0.678885686	4.21288E-09
LPIN2	0.947372215	4.38348E-09
SGMS2	0.927653981	4.41072E-09
DHRS7B	0.944154499	4.44276E-09
CYBB	0.876370055	4.84317E-09
LPCAT2	1.078093597	5.11835E-09
ADPGK	1.033478667	5.62002E-09
PGK1	1.041356865	5.63237E-09
SLC6A14	1.527636982	5.92155E-09
ST3GAL6	0.808363348	5.96432E-09
PDE8B	0.80611753	6.15027E-09
SLC35B1	1.037118859	6.22623E-09
PIK3R6	0.792287954	6.24388E-09
CYCS	0.961197979	6.27243E-09
G6PC2	0.434897202	6.30547E-09
CACNG5	0.166593531	6.72133E-09
ABHD2	1.063407187	6.87019E-09
PFKFB3	1.057505801	6.93979E-09
RDH12	1.795681419	7.18081E-09

SLC4A8	1.22719456	7.26924E-09
ALDH2	0.962576775	7.32311E-09
FDX1L	1.059526718	7.40355E-09
SLC27A2	0.926792108	7.62609E-09
FADS1	1.155075175	8.61945E-09
CYP2J2	0.923135943	8.72973E-09
MMAA	0.945962546	8.85783E-09
SLC26A9	6.050025413	8.90576E-09
SLC4A1AP	1.028507206	8.98472E-09
SLC25A16	0.946164501	9.18807E-09
SLC27A5	1.166299074	9.33945E-09
ATP5L	0.967388726	1.00153E-08
HEPH	0.940007419	1.01263E-08
CYP24A1	2.77030736	1.01805E-08
KCNK6	0.927022361	1.03219E-08
CYP2U1	0.909176639	1.10924E-08
CBR1	0.940366291	1.11645E-08
ATP6V1C1	1.039922455	1.11652E-08
PIGA	1.055958652	1.15686E-08
PCYT1B	0.637106851	1.18805E-08
GSTM3	0.858720925	1.19156E-08
BCAT2	0.949984373	1.25657E-08
ATP6V1G1	1.03285771	1.3038E-08
POFUT1	1.06349194	1.36974E-08
ABCG4	0.710262132	1.41766E-08
NUDT7	0.922505257	1.4292E-08
ATP5C1	0.969414276	1.4397E-08
SCNN1A	0.920394119	1.45267E-08
CHSY1	1.056779399	1.51496E-08
CYP27C1	0.705888865	1.58576E-08
SRR	0.931151025	1.62111E-08
NAGK	0.960104037	1.62657E-08
KCNJ5	0.685358769	1.66643E-08
ABCC6	0.872836176	1.67677E-08
KCNK13	0.778322833	1.68475E-08
KMO	0.784637298	1.71224E-08
SFXN5	0.933036455	1.76175E-08
LPIN1	1.061335428	1.79129E-08
ASL	0.951945666	1.87175E-08
C9orf95	0.941627683	1.88445E-08
PCYT1A	0.96263912	1.98404E-08
CYP2W1	1.476041443	1.99079E-08
LST-3TM12	22.93577568	2.08395E-08
RHBG	10.66253671	2.11512E-08

APRT	1.041648245	2.13007E-08
NAGPA	0.958319162	2.18967E-08
LASS4	0.810739859	2.24044E-08
SLC35A2	1.036633059	2.27345E-08
SLC17A1	0.391735955	2.36938E-08
MAT1A	1.641908685	2.49924E-08
SLC40A1	0.933707895	2.58502E-08
LIPJ	0.36260653	2.65438E-08
PI4KB	0.975619099	2.94606E-08
MCEE	0.940461123	3.0631E-08
LYPLA1	1.051756482	3.10013E-08
UGT2B7	0.744860932	3.12598E-08
ISCA2	0.952269084	3.34992E-08
CACNA2D2	0.791452843	3.41429E-08
SLC35F5	0.956958475	3.55351E-08
CYP46A1	0.666429281	3.64317E-08
ALDH1L1	0.707438474	3.90926E-08
ATP5G1	0.952300402	3.99253E-08
NUDT12	0.875418325	3.99957E-08
ENOX1	0.777951457	4.13454E-08
PLA2G16	1.123456291	4.79455E-08
ATP5D	0.95383775	4.91848E-08
PLA2G3	3.429379145	4.93275E-08
GATM	0.865947133	5.04201E-08
ENPP3	0.74833043	5.04919E-08
KCTD1	0.882484824	5.18948E-08
TRPM2	1.157063862	5.30914E-08
FADS3	1.093462165	5.53657E-08
RHCG	1.681552733	5.58404E-08
LIPA	0.947259919	5.61065E-08
SLC6A17	0.690975152	5.75976E-08
HS6ST2	1.416343562	6.60153E-08
FOLR1	1.846930159	6.65661E-08
SPNS1	1.033377399	6.84337E-08
AGXT	0.702649246	7.35248E-08
SLC4A9	0.544867962	7.70391E-08
GALNT8	0.767688183	8.15512E-08
SPHK1	1.186326342	8.34853E-08
ADAL	0.911944738	8.66082E-08
CHST4	2.818218271	8.78307E-08
KCNV2	2.308378635	8.85679E-08
PFKL	0.969231016	9.1375E-08
PIP4K2C	0.969734172	9.43784E-08
AQP9	1.469525823	9.66118E-08

AADAT	1.133993786	9.79504E-08
CRLS1	1.053288224	1.03279E-07
STARD13	0.930759948	1.06334E-07
PMVK	0.960983086	1.13747E-07
EDEM1	0.964132748	1.1949E-07
PNPLA6	0.969921328	1.2384E-07
PPOX	1.052887593	1.25155E-07
COX7B	0.958352657	1.25622E-07
FUT8	1.072410904	1.28278E-07
SLC25A36	1.036699309	1.35495E-07
NPC2	0.964382323	1.40615E-07
ELOVL1	0.972340878	1.55958E-07
GUCY1A3	0.873173238	1.67816E-07
GLS	1.041814648	1.68173E-07
CYB561	1.030438832	1.74878E-07
MTR	1.039938522	1.75761E-07
KCTD5	0.970894777	1.76246E-07
NPC1L1	1.731412869	1.7804E-07
CA11	0.857098404	1.79779E-07
MANEAL	1.091872136	1.81776E-07
CES1	0.791219504	1.83078E-07
GLT8D1	1.027728241	1.92288E-07
GMPR2	0.969542041	2.01758E-07
AHCYL1	0.975508327	2.1715E-07
PCTP	0.954766629	2.192E-07
PLA2G4F	0.888377243	2.24447E-07
SLC35C1	0.967775971	2.25651E-07
ACAD10	0.960595275	2.27342E-07
SLC6A18	0.52259038	2.37863E-07
B4GALNT1	0.822016095	2.51917E-07
GAL3ST4	0.914298258	2.65325E-07
CS	0.977078168	2.66231E-07
PIK3R1	0.952721407	2.67142E-07
GAL3ST1	0.865678537	2.70721E-07
COASY	1.028211887	2.76093E-07
PAPSS1	1.034001618	2.76199E-07
CYP19A1	1.565436846	2.80643E-07
SLC25A1	0.964104734	2.98418E-07
PLA2G2A	0.79849528	3.12024E-07
SLC37A1	0.951131876	3.12268E-07
MCART6	0.813360213	3.15309E-07
NDUFA13	0.966259743	3.1874E-07
SFXN4	1.044011386	3.22289E-07
BCMO1	0.855828756	3.49696E-07

ME1	1.098323373	3.55128E-07
PGD	1.038202271	3.56312E-07
AK7	0.848398132	3.60211E-07
NUDT18	0.9312531	3.62066E-07
SLC45A1	0.832151648	3.66708E-07
SLC38A2	1.028955796	3.66855E-07
ABHD1	1.512358313	3.69442E-07
UGT1A4	0.254745297	3.72224E-07
CYP2C8	0.633246646	3.73731E-07
PDHB	0.971941363	3.83024E-07
KCNJ15	1.496511161	3.86776E-07
CUBN	0.814069477	3.99271E-07
PCYOX1L	1.069797916	4.10569E-07
GGH	1.088386376	4.14388E-07
PCYOX1	0.969341892	4.24779E-07
MBOAT7	0.967510323	4.32249E-07
CYP4F22	0.624377723	4.45639E-07
AADAC	2.102702096	4.57089E-07
ATP1A1	0.969558619	4.68384E-07
POMT1	0.96535704	4.87778E-07
CHPT1	0.960975979	5.08321E-07
SGPL1	0.973506933	5.39184E-07
COQ5	0.968410376	5.39438E-07
BEST3	3.609357639	5.46137E-07
PHGDH	1.132831001	5.90027E-07
DGKH	1.10603217	6.11102E-07
ETFB	0.958952909	6.48266E-07
EBP	1.046037216	6.53236E-07
SLC18A1	0.652177407	6.7128E-07
CACNG2	0.123657439	6.84481E-07
ATP5H	0.97127176	7.2898E-07
PPA2	0.966225777	7.41958E-07
CLIC4	0.943870255	7.47711E-07
NQO1	1.074206397	7.77922E-07
BCO2	0.808896142	7.94947E-07
SLCO1B1	19.52732793	8.25332E-07
CRYM	0.863320014	8.36239E-07
MGST1	0.937082373	8.91935E-07
NDUFB2	0.968555824	8.91997E-07
IDS	0.959904012	9.18994E-07
HSDL1	1.037693184	9.2182E-07
CYP20A1	0.974018058	9.30831E-07
SLC6A12	0.776644488	9.40129E-07
DGKQ	0.95413178	9.57508E-07

POFUT2	1.036187316	9.90494E-07
ATP7B	1.06907577	9.99242E-07
HSD17B14	0.852682072	1.01966E-06
KCNQ1	1.072978922	1.04044E-06
HSD17B12	1.038551259	1.04897E-06
SDHC	0.974752129	1.0538E-06
SLC16A1	0.949668097	1.07339E-06
SC4MOL	1.051821788	1.13678E-06
SLC2A8	1.065995789	1.13814E-06
ACSS3	0.748509978	1.14375E-06
PLA2G4A	0.810063638	1.14468E-06
HS3ST3B1	0.769966352	1.16743E-06
DOLK	1.02889176	1.17128E-06
SOAT1	0.923672213	1.17203E-06
EDEM3	0.958680928	1.17636E-06
GAL3ST2	0.838848706	1.21466E-06
KCNJ2	0.904466537	1.22237E-06
KCNE1	0.612644981	1.29656E-06
NAT8L	0.682467976	1.31218E-06
ATP6V1A	0.975829012	1.31896E-06
ENOSF1	0.941983107	1.39646E-06
MIOX	4.598962858	1.44842E-06
DDT	0.957855381	1.47402E-06
SLC39A1	1.023593754	1.49413E-06
SLC16A14	0.880984758	1.49901E-06
SLC16A13	1.073170204	1.52219E-06
SLC24A2	3.119587661	1.59728E-06
NPR3	0.652908704	1.64074E-06
DHFRL1	0.955156294	1.65511E-06
SLC16A4	1.140566518	1.66417E-06
KCNMB3	1.121920392	1.68109E-06
LASS2	1.020520739	1.76965E-06
MAN2A2	1.0342835	1.92185E-06
NDUFB4	0.97465927	1.9286E-06
ADSS	1.025332476	1.93625E-06
CATSPER1	1.854686772	1.97316E-06
ALG14	0.959959941	1.98483E-06
MCCC2	1.034660224	2.00929E-06
CACNA2D4	0.89418159	2.0234E-06
ACAD9	1.023895809	2.02799E-06
CYP4X1	1.326957226	2.05641E-06
CYP4F8	3.303744611	2.0891E-06
PTGES2	1.033088326	2.09901E-06
SLC28A3	1.546080505	2.1369E-06

NEU3	1.090970547	2.15893E-06
ATP2A1	1.351963381	2.21648E-06
GNPTG	0.969690751	2.2243E-06
CYP1B1	0.797206479	2.22774E-06
ABCB4	0.830101246	2.29276E-06
PLD3	0.9678496	2.3785E-06
UGT2B10	0.609999396	2.40924E-06
FA2H	0.944748401	2.43734E-06
INMT	0.876123169	2.44881E-06
CYB5R4	0.960456115	2.5916E-06
PDSS2	0.968441391	2.61931E-06
ACO1	0.969756203	2.65019E-06
ADA	1.108010026	2.6779E-06
PIP4K2A	0.957330748	2.84347E-06
UCK2	1.031044055	2.93122E-06
PFKM	1.043821332	2.95563E-06
PGAM2	0.84854891	2.9571E-06
GANAB	1.020024831	3.13294E-06
SULT1C4	0.74283477	3.33312E-06
PPAPDC2	0.960323514	3.36082E-06
NUDT15	1.039717702	3.38521E-06
CHRNG	2.771742839	3.40122E-06
UQCRQ	0.962927188	3.73648E-06
WWOX	1.070956019	3.92993E-06
MPST	0.964271366	3.96741E-06
HMGCR	0.963927812	4.07627E-06
AQP12A	0.587164893	4.1394E-06
FPGT	0.963630513	4.17768E-06
GANC	0.961996118	4.24994E-06
ATP6V1B1	1.674834501	4.3758E-06
GLRA2	0.655344623	4.44976E-06
NME7	1.055594516	4.50763E-06
KCNG2	0.667031622	4.65358E-06
FAR1	0.972597219	4.86012E-06
KCTD6	1.056419742	5.18787E-06
CTNS	0.960978594	5.55343E-06
DHRS3	0.958856461	5.71118E-06
TRPM7	0.969406537	5.75248E-06
NAT14	1.104636926	5.78631E-06
GSS	1.033359618	5.96897E-06
MTHFSD	1.0358784	6.11751E-06
OXCT1	0.916194619	6.44561E-06
FMO3	1.28886907	6.53432E-06
LGSN	0.467593831	6.73232E-06

PGAM4	0.935845765	6.88472E-06
DDO	0.813203684	6.92614E-06
NUDT4	1.042564327	6.93808E-06
ATP5SL	0.97558503	7.44036E-06
HSD17B13	0.785437858	7.52814E-06
GGPS1	1.027438903	7.55554E-06
AS3MT	0.827544144	7.75636E-06
SLC2A7	0.366535976	7.92724E-06
HNMT	0.957445395	7.98619E-06
EDEM2	1.031667843	8.00262E-06
ST6GALNAC2	0.85127153	8.66922E-06
SLC22A20	1.343198258	8.69707E-06
IVD	0.965952579	8.72238E-06
DHFR	1.051193742	9.01631E-06
FN3K	0.947548953	9.05835E-06
PTDSS2	1.037143685	9.21246E-06
GLCE	1.040833502	9.29729E-06
NAGS	0.881375481	9.37011E-06
CACNG8	10.93609815	9.48793E-06
DAK	1.039833355	9.57996E-06
ST6GALNAC4	0.941437802	9.61945E-06
SLC16A5	0.937350878	9.66196E-06
CLCN3	0.968037023	9.90998E-06
AQP12B	0.76971912	1.06835E-05
PTS	1.038604366	1.07535E-05
NDUFB5	0.97088038	1.09244E-05
ATP11B	0.973381869	1.10351E-05
IPPK	1.030339857	1.11408E-05
FUT4	1.045181629	1.12847E-05
CYP4F3	1.142020672	1.1466E-05
PIGG	0.974331372	1.14744E-05
ATP6V0A4	7.450263035	1.14991E-05
PI4K2B	1.031107071	1.15354E-05
ATP5I	0.962946459	1.15426E-05
AQP2	5.325141655	1.20094E-05
MECR	1.035618138	1.30174E-05
XYLT2	1.030695016	1.31358E-05
ATP6AP1	1.021092073	1.42794E-05
PRPSAP2	0.967602745	1.44765E-05
CYP51A1	1.035280744	1.49215E-05
B3GNT4	1.326167685	1.5013E-05
GSTM1	0.676792968	1.55447E-05
ADSL	1.032402137	1.61722E-05
FUT3	0.93422491	1.62637E-05

RENBP	0.889911175	1.705E-05
NOX5	0.57187002	1.7494E-05
SRD5A3	1.05858725	1.77391E-05
AQP5	2.471705889	1.90372E-05
CTH	0.920160875	1.90599E-05
KCTD15	0.915500556	1.94578E-05
ABCF1	1.022675316	1.99013E-05
LPIN3	1.057239424	1.99382E-05
KCNE1L	1.552922763	2.0089E-05
GALNT2	1.024607763	2.12407E-05
ST8SIA5	0.64700985	2.1394E-05
SLC24A3	0.885190937	2.15475E-05
TPH2	0.338030377	2.16601E-05
TDO2	1.245730837	2.22285E-05
KHK	1.069996291	2.2437E-05
DPYSL3	0.917084907	2.2588E-05
B3GAT3	1.032144666	2.35443E-05
GLYAT	0.331485326	2.37792E-05
CYP26A1	2.256427682	2.38014E-05
SLC9A10	1.714967581	2.42539E-05
PIGV	0.967288739	2.5717E-05
GALC	0.910978099	2.58638E-05
SLC30A7	1.026533524	2.60784E-05
PIGB	0.965112788	2.67344E-05
CYB5R2	1.099885185	2.79772E-05
SLC25A26	1.031594882	2.98199E-05
GLT1D1	0.738551674	2.98704E-05
GLDC	0.716558958	3.05567E-05
NMNAT2	0.814286414	3.13429E-05
SLC12A3	0.6379955	3.15511E-05
GALT	0.967408367	3.18666E-05
DDOST	1.018845787	3.20363E-05
ALOX5AP	0.911371797	3.26299E-05
PCCB	0.972746651	3.29173E-05
CHRNA6	2.708104055	3.41284E-05
GLRX	0.947574612	3.49515E-05
KCNC4	1.051972979	3.68017E-05
INPP5B	0.970517604	3.73499E-05
DAGLB	1.026019414	4.02882E-05
ST3GAL5	0.904461238	4.04098E-05
ALDH1B1	1.041142808	4.07244E-05
HIBADH	1.030382575	4.23982E-05
BEST1	0.89295714	4.25298E-05
ECHDC3	0.768048001	4.26415E-05

A4GALT	0.909395809	4.48734E-05
CLIC3	1.185867302	4.61742E-05
ACCN4	2.02713594	4.91989E-05
ACSF3	0.970096128	4.97161E-05
ACSBG2	1.965439198	5.00778E-05
SPTLC2	0.970192903	5.23198E-05
DPM3	0.951227203	5.4393E-05
SLC25A41	0.712345261	5.72872E-05
GCLM	1.038693634	5.87045E-05
KCNJ10	0.814181898	5.96615E-05
SLC9A6	0.972658608	6.04457E-05
ALDOC	1.066924484	6.06548E-05
ALDH1A2	0.695609021	6.27325E-05
TRPV2	0.922105519	6.40033E-05
SCN5A	0.767418503	6.50542E-05
AGA	0.961570795	6.61515E-05
FADS2	1.115702245	6.73544E-05
SLC6A5	0.098755547	6.82571E-05
NME5	0.714036536	6.89025E-05
ARSF	0.451676294	6.93814E-05
OAT	0.955314861	7.0795E-05
DLAT	0.975129599	7.11497E-05
ACER2	0.90900295	7.24593E-05
MOGAT1	4.488681277	7.38934E-05
SLC35B4	1.037849799	7.44986E-05
GLT25D2	0.771787483	7.53011E-05
FUT6	0.931843627	7.79537E-05
HK1	0.973381988	8.00198E-05
ARG1	0.519475532	8.05709E-05
FUT2	0.957226007	8.15505E-05
PLA2G12B	0.715801802	8.16141E-05
COX6B1	0.975903752	8.72402E-05
CBR4	0.967185132	8.74081E-05
B3GAT1	0.79287648	8.85265E-05
PLA2G15	1.029802813	9.21126E-05
NEU1	1.042318873	9.38041E-05
NME4	1.041671634	9.59388E-05
ACSM2A	0.323964106	9.77439E-05
OLAH	2.830132441	0.000101472
KCTD21	0.968413716	0.000103319
SLC25A2	1.899974842	0.000105887
UROC1	4.057060867	0.000106407
KCNJ6	0.588658545	0.000107027
ABHD10	1.026318305	0.000108254

SLC16A2	0.897334215	0.00010973
AK1	0.948206353	0.000110137
CHPF2	1.022914243	0.000110527
AGMAT	1.052224682	0.000114484
ABCA3	0.869459712	0.000116008
LTA4H	1.023646483	0.000120531
ATP5E	1.027489497	0.000121556
DGAT1	0.966050347	0.000123103
FDXR	1.052758695	0.000130678
ARSH	1.834270579	0.000133458
ILVBL	0.975225121	0.000135137
SLC38A3	1.759107101	0.000135823
SLC6A3	2.427431835	0.000138604
ABCA12	2.323789122	0.000140064
IMPAD1	1.022070006	0.000140307
ATP6V0C	0.976874735	0.000140566
SDR42E1	0.878914594	0.000141405
COQ6	0.969256906	0.000143101
ABHD11	1.033863464	0.000144026
FDPS	1.026854225	0.000147573
SLC27A3	0.955640355	0.000150587
COX15	0.980785952	0.000157497
SLC19A3	0.854248057	0.000160419
NAT8	0.596567977	0.000162202
B3GALTL	1.039507913	0.000164763
BHMT	0.561783378	0.000168428
OXA1L	0.979105872	0.000168563
PGM2	1.02763371	0.000169759
ACE	0.94818568	0.000170727
KCNA10	4.548433678	0.000172828
LDHAL6A	0.675700684	0.000176518
PDE4B	0.914469508	0.000177066
KCTD19	1.702710035	0.000178362
GCAT	1.057016637	0.000181875
MOGAT3	0.902172013	0.000190877
NDUFA9	0.97270659	0.000196227
PFKFB4	1.072650161	0.000196346
SLC5A1	1.061402976	0.000197182
HYAL1	0.859495878	0.000201162
ALG9	1.018978326	0.000203149
GNMT	1.53778746	0.000209806
GUCY1B3	0.912930971	0.00023068
ORAI2	1.045827019	0.000234302
MANBAL	1.024191408	0.000236969

SLCO1A2	2.974826131	0.000238349
SLC25A45	0.945487309	0.000245637
ST8SIA2	0.772630481	0.000248498
LDHB	1.053727616	0.000258109
GLT8D2	0.908450064	0.000264386
CACNA1I	0.753091524	0.000267288
PKD2L1	0.722699278	0.000270889
ATP1A4	0.576433614	0.000280044
AOC2	1.210012355	0.000280346
TPCN2	0.971450672	0.000282145
PGS1	1.028949109	0.000286854
NDUFAF1	0.969072945	0.000287413
SLC45A3	0.941124773	0.000292284
HCN3	1.05554805	0.000301124
GAA	0.953483046	0.000304002
ENTPD6	1.03152743	0.000306599
SLC39A8	0.950553374	0.000309267
B3GNT2	0.976551127	0.000311467
PLCB4	1.08765264	0.000315993
B4GALT6	1.059039577	0.000317534
ORAI1	1.026233068	0.000324839
ACAD11	1.045466267	0.000325337
SLC38A5	1.124283227	0.000326972
GGT1	0.914308299	0.000332658
CDADC1	1.033876873	0.000339169
SLC15A3	0.936518972	0.000340417
MGAT3	0.902168973	0.00034167
DIO1	0.621401533	0.0003531
PIP4K2B	1.022087427	0.000356429
MINPP1	0.967437796	0.000361665
SLC25A10	1.032711191	0.000363201
KCND2	0.754133551	0.000365196
ABAT	0.921121341	0.000375921
CKM	0.726531436	0.000389329
CHST13	0.822838952	0.000421834
GRIN1	0.813586286	0.000424896
HYAL4	0.589446975	0.00042757
PIK3CB	0.981608863	0.000434232
HCCS	0.973399682	0.000436631
GSTA4	0.952670939	0.000446822
ATP10B	0.95307629	0.000450841
CDIPT	0.981553464	0.000451716
LYG1	1.295464216	0.000452409
HPDL	1.10618301	0.000457527

SLN	1.802262921	0.000458982
SLC39A2	0.711226936	0.000476553
LASS6	1.028409887	0.000484078
GRIN3B	1.982278762	0.000491176
DGKD	0.973098964	0.000503333
UAP1L1	1.056636775	0.000524468
IDO2	0.709986618	0.000527743
PRPS1L1	2.6312652	0.000535218
SLC30A9	0.981191271	0.000538969
CACNA1G	0.749349198	0.000540786
HS3ST1	1.053343639	0.000542954
NDST2	0.976481378	0.000546012
PNPLA1	1.335278951	0.000564093
COX6A2	0.301266168	0.00056548
GK5	0.973790376	0.000586544
SLC34A1	2.750742754	0.000602692
SLC2A10	0.925074577	0.000609336
GUCY2C	0.939012255	0.000627939
HAAO	0.905623601	0.00062958
OAS1	0.962792245	0.000661181
CYP2C9	0.70285541	0.000665194
HS6ST1	0.969652554	0.000666504
CACNG4	1.443917596	0.000675825
MDH2	1.016299768	0.000682464
MFSD3	1.047729038	0.000712232
GCLC	1.021740523	0.000724033
SLC22A12	12.13059259	0.000727519
CHDH	1.045120211	0.000746296
GABRB1	1.717151593	0.000797547
FOLH1	1.154812673	0.000804648
MOCOS	1.071418331	0.000820131
NT5M	0.896310977	0.00082277
MMAB	1.029763035	0.000824379
LYZL4	8.424147298	0.000832444
GPX4	1.025892012	0.000840914
DHDH	1.234913957	0.000882032
SLC39A9	0.985188593	0.000886389
PANK1	0.96118917	0.000923364
CYC1	0.974882836	0.000925554
B4GALNT4	1.279592666	0.000942499
NEU2	0.373574144	0.000961724
LIPI	0.524026192	0.000970578
KCNH6	0.734969722	0.000975817
CYP4Z1	3.363932161	0.001001138

ARG2	1.044996225	0.001006289
AGPAT2	0.971047316	0.001007202
NT5C	0.972540003	0.001011717
P2RX5	0.898547223	0.001027185
ATP11C	1.032570222	0.001042876
ACOT8	0.966720723	0.0010575
PKD2	0.947862668	0.001057611
GCH1	0.973621717	0.001058615
DGUOK	1.022824077	0.001061478
ABCC9	0.87408881	0.001098217
ASAH2B	1.246642413	0.001103769
KCNAB2	1.062595037	0.001109681
ATP5S	0.964807639	0.00112352
GTDC1	0.975074671	0.001160693
CA6	12.76697247	0.001169029
INPPL1	1.015753673	0.001171933
KCNK1	0.934227373	0.001173721
GK	0.951150087	0.001218679
ENTPD2	1.061150937	0.001275376
HCN2	0.815016581	0.001294978
SRD5A2	3.168035572	0.001313476
ATP6V1B2	0.973895246	0.001314887
DDHD1	1.029179226	0.001316019
PIGC	1.023617156	0.001318071
GLB1	1.017674832	0.001344111
ASRGL1	0.929975487	0.00135056
SLC14A1	0.800357376	0.001351242
KCND1	0.920008693	0.001366665
CYB5R1	0.975465284	0.001374212
MGAT5	1.057642128	0.001377132
CHST10	0.895461707	0.001409842
GALNT4	1.038852699	0.001411059
ATP10D	0.952989507	0.001458226
QDPR	1.024344996	0.001459721
NADSYN1	0.977562767	0.00147346
ADI1	0.982433209	0.001475221
LIAS	0.969393805	0.001479365
AGPS	0.984636982	0.001488191
ACOXL	0.760903457	0.00149803
CYP2C19	0.69626609	0.001498687
MCAT	1.028702682	0.001501801
FUCA2	1.016213884	0.001504557
GPX8	1.067016503	0.001511029
HACL1	1.026720329	0.00152439

CHST1	1.112816609	0.001536334
CERKL	0.921152849	0.001553957
NDUFA12	0.979341682	0.001606301
GLB1L3	0.438738285	0.001617917
ATP5J2	1.026207399	0.001625774
PDE10A	1.13642087	0.001630594
CYB5RL	1.048041851	0.001650912
NNMT	1.07393198	0.001666322
PPCDC	0.965713951	0.001684556
ACCN1	0.751100893	0.001712164
SLC6A7	0.876207904	0.001727751
SPHK2	0.970404898	0.001751624
CHST14	1.035581621	0.001760362
ARSB	0.95884412	0.001782823
RPE65	0.643554246	0.001787753
DGKE	1.03574932	0.001796639
COX6C	0.977121053	0.001849252
ABHD14B	0.977293414	0.001853257
LYPLAL1	1.034858513	0.001860443
NDUFA4	1.021049935	0.001864736
XYLB	1.0401358	0.001878548
PLA1A	1.133416995	0.001950434
ACSS1	1.039355852	0.001962545
ABCB8	1.022202197	0.00196612
MFSD8	0.978119034	0.002021261
GLRB	0.842988395	0.00206559
INPP5F	0.978688933	0.002106503
NDUFB9	0.97805487	0.002141456
TBXAS1	1.063416318	0.002145303
SLC22A13	1.423201837	0.002159689
NT5E	0.944026054	0.002163317
SCN10A	0.370596213	0.002166745
PGM2L1	1.044782766	0.002173485
PYGL	0.935358916	0.002178624
SLC24A1	1.035063384	0.002193703
ATP6V1H	1.020280105	0.002213899
IDH1	0.981101249	0.00221952
HAS1	0.738572911	0.002276193
PLA2G12A	0.980052718	0.002280925
CEPT1	1.018086048	0.002288701
CSGALNACT2	1.039199941	0.00233161
TAP1	1.033494468	0.002377076
SLC39A3	1.02209755	0.002415993
MVK	1.028939265	0.002419294

CACNA1C	0.925346455	0.002433898
SLC25A30	0.960648351	0.00248255
KCTD20	1.022126037	0.002547707
GABRP	1.334543005	0.002550471
CLCN6	0.966995265	0.002556813
PIGT	1.017539591	0.002603102
SLC37A3	1.018040214	0.002638707
ADCY7	0.959495675	0.002712592
CYP2F1	1.802840266	0.002749716
ACSL3	1.018608239	0.002761748
SULF2	1.038499691	0.002889348
MGST2	0.976622994	0.002966513
SLC26A6	0.972918353	0.002976068
KCTD16	1.474977464	0.002999517
PIP5KL1	1.28747251	0.003087113
SGMS1	0.969616369	0.003145802
SLC13A5	1.847288653	0.003239523
SLC27A1	1.047770671	0.003244424
SLC12A5	0.774085534	0.003255211
PLCB2	0.943853757	0.003269658
ALOX12B	0.75597478	0.003433563
GYG2	1.079934997	0.003486257
ADCY8	0.444870626	0.003520541
SLC5A2	1.497375501	0.003544498
PHOSPHO1	0.853155287	0.003601003
SLC9A4	0.67645906	0.003623494
PIK3C2A	1.018377788	0.003729978
TRPC3	0.828469081	0.003739672
SLC23A2	0.963652609	0.003751338
MCCC1	0.975278205	0.003784953
NDUFB6	0.978816749	0.003814037
SLC35E3	0.97512465	0.003836374
SULT1C3	2.226312337	0.003854788
HSDL2	0.97875633	0.003914209
MANBA	1.024428402	0.003939733
PDE6C	0.559046416	0.003956954
KCNH5	0.371104505	0.004010949
GDA	0.94318739	0.004161719
CABC1	1.022931125	0.004170754
AGPAT4	0.926715271	0.004173205
CYP11B1	0.302175835	0.004176584
HEXA	0.977158968	0.00422639
TRPC4	0.869192536	0.004267208
B3GAT2	0.902008779	0.004282465

ALDH3A1	0.908215933	0.00437624
MVD	1.030505326	0.004382946
PIGQ	1.020548654	0.004397672
CKMT2	1.317591474	0.004441381
CHRNB3	#DIV/0!	0.00444801
HCN1	1.401681458	0.004481572
SLC39A14	0.97650667	0.004500903
GYS1	0.984255975	0.004520241
B3GNT3	0.977681888	0.004542744
UCP2	0.958911603	0.00459583
DCT	0.563907156	0.00460456
MOSC1	0.95972145	0.004613944
PIK3CA	0.976604831	0.004698403
L2HGDH	1.029883694	0.004702378
CLCC1	1.015082301	0.004734596
PTGES	1.072655169	0.004789098
SLC4A7	1.03037956	0.004863315
MAN1B1	1.015859688	0.004901374
SLC35E2	0.943480003	0.004910448
CSAD	1.057891016	0.004933513
GNPAT	1.015170184	0.005069231
NT5C2	0.985089605	0.005161571
MMACHC	1.030569974	0.005213995
MOCS2	0.979395132	0.005227334
UCP3	0.92006707	0.005295926
UGT2B4	3.343540609	0.005310434
SARDH	0.899291661	0.00535102
PLA2G4C	0.923406291	0.005382703
DDC	0.937591691	0.005392551
ESD	1.018955788	0.005596189
GGTLC2	0.908011661	0.005605973
GUCY1A2	1.240984289	0.005631157
EPX	0.737863007	0.005710531
LNPEP	0.964656379	0.005814369
SLC39A4	1.03953663	0.00583815
CHKA	0.972687649	0.005922268
ABCF3	1.011352664	0.005965688
GSTA5	0	0.00600279
CACNG1	4.19373902	0.00603391
CPT1B	1.067747261	0.006044506
CA8	0.844923557	0.006168525
BCKDHB	0.97546264	0.006213302
KCNA7	5.732116852	0.006234446
ABHD12	1.025179868	0.006355554

PDE11A	0.885632813	0.006440895
ENPP5	1.07129492	0.006619006
MSRB2	0.976338792	0.006624895
RDH13	1.023600598	0.006647885
CYP17A1	2.870065257	0.006744779
GALNTL6	0.68665176	0.006771536
NIPA1	1.023765512	0.006801967
ACOX2	0.937828574	0.006863054
GALNT11	1.024208837	0.006881495
GYG1	0.97900258	0.007111334
GALNS	0.974857832	0.007225442
GLUL	0.976118946	0.007315776
FOLR3	0.658355815	0.007459331
NDUFAB1	0.98241363	0.007545998
HSD11B1	0.871201317	0.007592025
ABHD13	1.020097172	0.007710763
MTHFD2L	1.031876332	0.008103454
PIP5K1A	1.015494141	0.008296832
EXTL3	1.02651195	0.008679679
PIGK	0.982021988	0.00883077
C9orf98	1.16540965	0.009006006
GSTK1	0.981479872	0.009156531
SLC44A3	0.972453056	0.009312986
CYP7B1	0.870717125	0.009379984
UGT1A3	0.653820068	0.00972645
AKR7L	0.944165236	0.009806854
DUOX1	1.116474425	0.00991613
GSTO1	1.021071017	0.010003298
CRYZ	1.03690526	0.010037473
HSD3B7	1.029756395	0.010106175
NT5C3	0.983527968	0.010176111
GOT2	1.013821706	0.010722956
CHST12	0.973183488	0.011021116
ABCG5	1.326601234	0.011187374
DDAH2	0.973872064	0.011211676
SLC10A3	1.01802624	0.011534605
SCN1A	0.563893572	0.011683464
ABHD14A	0.967572146	0.011709669
PLCB1	1.065849502	0.011792748
NUDT13	0.951055134	0.011818899
UGT1A6	1.128012583	0.012215652
KCNH2	0.937595039	0.012342785
SLC30A2	1.272622944	0.012684539
PLCH1	1.031238724	0.012767256

GFPT2	0.898702469	0.012929004
POMT2	1.022095475	0.012981369
PGK2	11.7885531	0.013422591
ITPR3	1.020134043	0.01382621
TYMS	1.032037026	0.013868319
ATP13A2	1.020324091	0.013915956
ALDOA	0.987399042	0.01396992
CYP2D6	0.894380824	0.013984492
ADAD2	0.639167	0.01407147
MBOAT2	0.95747845	0.014352566
PTGR2	0.966397545	0.01443735
POR	1.016340177	0.014629466
SHMT1	0.976778343	0.015392258
ELOVL3	1.282577639	0.01585456
PDXK	1.018022061	0.015878886
ACCN3	1.209403774	0.016042523
KCNQ2	0.624380384	0.016045521
ABCD1	0.972896763	0.016124604
PLA2G4D	1.218358742	0.016201357
NT5C1A	0.506061016	0.016253002
NME3	0.973829567	0.016313663
NDUFA8	0.984369309	0.016940644
HEXDC	1.029998617	0.017805971
GUK1	1.016643656	0.017975261
PIGP	1.024566051	0.01799632
ATP12A	0.665752986	0.018281144
SLC1A6	0.393813341	0.01840619
ABHD12B	0.760801061	0.018535779
SLC22A2	0.728469676	0.018753446
TRPV6	0.715006493	0.018867813
LYZL2	#DIV/0!	0.019052259
ATP2C2	0.974973635	0.019319653
ACOT6	0.729042052	0.019329058
PLB1	0.925329098	0.019366719
GLRX2	1.025251621	0.019946134
SC5DL	1.018181618	0.020026618
EXTL2	1.025088012	0.020037465
TKTL1	0.662206775	0.020426802
SLC39A11	1.020547463	0.020559868
HSD3B1	0.786489085	0.021230338
ACSM2B	0.366541868	0.021390355
STARD6	0.264389967	0.021490771
UCP1	6.667976593	0.021523603
INPP1	0.977667706	0.021818165

SLC30A6	1.019792032	0.021858701
HTR3D	0.292579951	0.022543035
B3GNT9	1.03191067	0.022566521
NDUFC2	0.986155966	0.022573129
CATSPER2	1.072393893	0.022717275
CBS	0.920809559	0.023575187
KCNJ13	0.693833881	0.024433641
PDE12	1.013093919	0.024450347
LIPE	1.038956045	0.024558594
GUCY2D	1.570666801	0.024962046
PIGY	0.987655257	0.025379278
PRDX2	1.013728297	0.025661748
SCD5	0.944085334	0.026016635
LCTL	1.21215417	0.026116001
ALG2	1.012441146	0.026413416
SLC7A8	1.03909901	0.026531094
CYBA	0.979550344	0.02653497
PGAM1	0.985793504	0.027023006
NDUFB11	1.015209917	0.027221076
CYP4F2	0.878161265	0.027230951
FUT11	0.965908912	0.027244982
ACSL5	0.98241435	0.027674521
SLC45A2	0.821374827	0.027763558
PKD1	0.978365564	0.027793864
BCKDHA	0.985637748	0.028036339
PLA2G7	0.940723087	0.028290658
KCNS1	1.312515862	0.028599875
FUT7	1.121257527	0.029073279
C6orf192	0.974498493	0.029221173
UROS	1.017198833	0.029487629
KCNAB3	1.144712386	0.029929998
TMEM104	1.01605322	0.030358939
CYP3A43	2.102966049	0.030491775
BLVRA	0.957611734	0.030608385
ALOX15B	0.865939382	0.030739831
KCNQ3	1.22989603	0.031289183
KCNC3	1.071220453	0.031313848
HMOX2	1.011925671	0.031608974
ATP5L2	0.785747524	0.032307517
GPHN	1.024871371	0.032373696
HS3ST4	1.900474343	0.033908483
COQ2	0.978899146	0.033972656
CYP3A5	0.958039456	0.034158073
ASS1	0.97543775	0.034729147

LPPR4	1.085452217	0.0353211
NDUFA3	0.981140245	0.035979285
NDUFS8	0.984820667	0.036303558
ATP1B1	0.98072541	0.036428605
OXCT2	0.926107782	0.036429856
UPB1	0.825131075	0.037337973
CYB5D1	0.97814302	0.037764088
ST6GAL2	0.878071681	0.037856131
GALK2	1.017199822	0.038865274
SLC22A14	1.45215429	0.039644059
STARD3NL	1.01514183	0.040289631
ACY1	0.980522307	0.040456292
SCN2A	0.813050191	0.040526081
ABCA2	1.023236747	0.041114455
BPGM	0.983671181	0.041236661
SLC26A1	1.044568679	0.041733443
SLC12A7	1.012672817	0.042170356
ATP6AP2	1.012303038	0.042428986
GMPR	1.064879717	0.043328349
SV2C	0.755263045	0.044279196
NME6	1.01360477	0.044465562
MAT2A	1.010878309	0.044932158
CSGALNACT1	1.044333734	0.044991603
IDH3B	0.983535493	0.045442589
SLC37A4	0.984410259	0.045508184
ST3GAL1	1.04388546	0.045866688
PRPSAP1	0.990057897	0.04604472
GALNT10	0.982322716	0.046392877
DEGS2	0.957784702	0.04674213
NAPRT1	0.977719958	0.046961483
PLA2G1B	0.697728405	0.047203638
ABCA13	1.14339082	0.048183037
CYP4F11	1.085597791	0.048346404
ADH7	0.318000557	0.04860776
SLC2A3	1.044330233	0.048831312
CYP2A7	0.605466645	0.048884619
MPO	1.181778824	0.048937386
ABCC4	0.966459416	0.049442988

Unpaired analysis in TCGA CRC RNA-Seq		
Metabolism-related genes	Tumor/Normal	P value
SLC4A4	0.045725615	1.4632E-218
ABCA8	0.048270791	4.9804E-201
GCNT2	0.084099504	1.6849E-184
BEST4	0.019657688	1.3507E-182
UGP2	0.254872146	6.6826E-181
SLC25A34	0.073675903	1.2617E-174
CA7	0.021148217	6.7911E-171
GLTP	0.362267632	1.5926E-170
ENPP6	0.10476523	6.9367E-165
CA1	0.012343771	2.0366E-162
CA4	0.042758336	4.0573E-159
ADH1C	0.06959841	4.6371E-156
SLC26A3	0.048821585	8.5541E-155
AHCYL2	0.169542942	2.6745E-153
SMPDL3A	0.178199781	4.8863E-152
ETFDH	0.302894131	2.3822E-150
SCN9A	0.094758116	3.2793E-150
ENTPD5	0.193734188	1.8095E-149
UGT1A8	0.059386396	5.4709E-147
UGT1A10	0.09434879	3.6262E-144
SLC26A2	0.067376142	2.5506E-142
B3GALT1	0.110340798	1.5083E-141
LDHD	0.146785021	5.9574E-136
SULT1A2	0.08283481	8.8274E-136
RETSAT	0.297957335	1.8642E-135
TRPM6	0.065056045	4.6842E-133
AMPD1	0.06932847	3.5112E-132
RDH5	0.231890091	2.6735E-130
SLC25A23	0.334994714	1.9163E-128
SLC22A18AS	0.218646299	6.3821E-127
FUCA1	0.319745847	9.1345E-126
PLCE1	0.266032804	3.112E-125
GSTM5	0.135723859	1.2002E-123
PLCD1	0.245866143	2.8691E-123
ACADS	0.253600916	1.2425E-120
SLC17A4	0.111268119	2.6812E-118
DHRS11	0.236884168	5.5297E-118
HSD11B2	0.231882939	3.4547E-117
MOGAT2	0.162095611	5.974E-117
ASPA	0.071129779	2.4125E-116

SULT1B1	0.169263831	1.0252E-115
NAAA	0.267075202	1.0442E-114
SCNN1B	0.070840321	1.1248E-111
ADHFE1	0.15509286	1.7751E-111
SLC22A5	0.316994538	2.1888E-110
SLC36A1	0.286164343	1.0017E-109
PLCL2	0.203913772	5.1665E-108
BEST2	0.087915488	4.5271E-106
CPT2	0.41009448	1.2877E-104
HSD17B2	0.104808742	8.9793E-104
CLCA4	0.027887163	7.2022E-103
DHRS7C	0.02966074	9.9695E-103
PDE9A	0.183362139	1.0984E-101
PAPSS2	0.277167493	1.9021E-100
PRDX6	0.403541081	3.8574E-100
CA12	0.22270223	5.3244E-100
UGDH	0.312556468	1.55189E-98
GPT	0.151195481	1.49094E-97
HPGD	0.154959361	1.67559E-97
GDPD2	0.122652623	2.40393E-96
UGT2B17	0.067176845	2.7983E-96
AKR1B10	0.130586516	1.44728E-95
ABHD3	0.292434376	3.42627E-95
PDE6A	0.106622329	1.0717E-93
DHDDS	0.473775204	3.02421E-93
ATP8B1	0.341104263	1.37734E-92
GPD1L	0.405969344	3.19063E-92
SLC30A10	0.026544233	1.12829E-90
SLC25A20	0.392357571	6.50077E-90
AQP8	0.009751018	2.17001E-88
SLC9A9	0.235761123	6.59574E-88
HMGCLL1	0.148327006	5.14905E-87
SMPD1	0.323825429	4.07875E-86
SCN11A	0.102965904	8.26357E-86
CES3	0.242807241	2.14593E-85
ST6GALNAC6	0.176227872	2.43308E-85
MAOA	0.273944023	4.98911E-84
ACACB	0.311784455	7.29422E-84
DHRS9	0.109166119	7.77662E-81
ABCG2	0.04460317	6.85746E-80
SLC9A2	0.234135246	3.42686E-79
FMO4	0.337784038	8.86655E-79
SLC17A7	0.140197141	9.34171E-78
ADCY9	0.403962419	2.37755E-77

CLIC5	0.261594729	3.25535E-76
PDE7B	0.232659665	4.49026E-76
SIAE	0.371590652	3.47572E-75
P2RX1	0.243940396	8.05674E-75
SUCLG2	0.437014006	9.84157E-75
ADCY5	0.150352471	3.88365E-74
SLCO2A1	0.266402115	9.07786E-74
FMO5	0.270359231	1.56659E-73
ACOX1	0.393117382	5.7656E-73
CMBL	0.332090848	9.74128E-73
B3GALT4	0.397915854	1.34408E-72
ACAA2	0.355889953	1.47586E-72
HPSE2	0.109281158	1.44637E-71
PDE2A	0.172257651	1.60931E-71
SCN4B	0.244900005	1.92579E-71
SCN2B	0.083401239	3.81198E-71
TRPV3	0.149105578	2.4309E-70
SLCO4C1	0.098385625	2.87384E-70
ATP6V1G2	0.193241166	4.08018E-70
UGT2A3	0.122156068	4.24041E-70
B4GALNT2	0.062576157	4.35585E-70
GRIK3	0.083654664	5.64539E-70
SLC2A13	0.330856721	1.01534E-69
PGM1	0.40983871	2.43558E-69
CES2	0.275426134	1.28769E-68
SULT1A1	0.249969337	6.21614E-68
GGT6	0.325080686	8.79316E-67
FLVCR2	0.287074011	3.08348E-66
TST	0.409705158	2.88489E-65
HADHA	0.587884335	3.47278E-65
HADHB	0.557397364	4.1432E-64
SLC41A2	0.362731447	2.15407E-63
PTGS1	0.147692718	2.24765E-63
MGLL	0.417854056	2.51423E-63
KCNE2	0.166246172	9.39788E-63
B3GNT7	0.121586158	4.92845E-62
SLC44A4	0.401410474	7.007E-62
GLRA4	0.172286035	2.01207E-61
B3GALT5	0.170392656	6.19904E-61
SLC16A9	0.214402826	1.15158E-60
TCN2	0.333024806	1.87273E-60
SLC9A1	0.42082748	2.9569E-60
ATP13A4	0.187389659	3.25428E-60
SLC16A12	0.149976205	5.43596E-60

BCHE	0.053934336	7.22369E-60
SLC5A7	0.0562173	2.04492E-59
DAO	0.036222217	2.46216E-59
PLCD3	0.373679312	3.60689E-59
KCTD9	0.423903787	7.4549E-59
ATP6V0D2	0.231370685	2.73769E-58
HSD17B11	0.442035611	4.91171E-58
SCP2	0.506176086	5.22207E-58
PGM5	0.093486625	1.1241E-57
SCN7A	0.031471097	6.70008E-57
FOLR2	0.209014829	7.46499E-57
SLC9A3	0.0957034	7.47662E-57
PDE8A	0.491007746	8.79095E-57
SLC22A23	0.395226155	2.09523E-56
CPT1A	0.482567357	2.55382E-56
SLC35D1	0.445946423	1.13412E-55
PCK1	0.162887046	1.48052E-55
NEU4	0.239978232	2.3126E-55
CHST5	0.19096541	7.62408E-55
GALNT12	0.402654993	1.69452E-54
P2RX4	0.450969348	4.76198E-54
INPP5A	0.572483951	1.64342E-53
CNGB1	0.095249611	2.31601E-53
KCNN3	0.201503276	2.68335E-53
ABCA9	0.130788191	6.68704E-53
ATP1B2	0.209104493	3.46107E-52
KCNMA1	0.122158439	3.41089E-51
ABHD5	0.502542558	4.07465E-51
ACO2	0.525240476	7.33697E-51
KCNA5	0.176603324	8.78522E-51
ATP1A2	0.035665953	9.53486E-51
XDH	0.2182399	9.94477E-51
MOCS1	0.358049877	3.3204E-50
RBKS	0.462675496	4.05518E-50
GDPD3	0.258207709	4.13444E-50
ACAT1	0.448147314	5.85391E-50
LPCAT3	0.486455935	3.04128E-49
B3GNT8	0.405111263	2.42297E-48
UGT2B15	0.132922696	6.71059E-48
ABCD3	0.523666264	8.19807E-48
ABCB11	0.042550933	9.8225E-48
ACADM	0.442530273	1.01808E-47
MGAT4A	0.458299104	1.2247E-46
PIGZ	0.348442765	1.65334E-46

PDE5A	0.272277489	2.42478E-46
PLCG2	0.304962426	2.80877E-46
HTR3E	0.111985803	1.09272E-45
ATP2A3	0.351315182	1.21509E-45
SDHD	0.502684578	1.27112E-45
SCN3A	0.232164448	4.37965E-45
MTHFD1L	5.616989074	4.38051E-45
P2RX2	0.077716212	2.24052E-44
CROT	0.470775077	6.29007E-44
CHAT	0.053956742	1.24366E-43
KCNA3	0.237862018	1.49183E-43
GCNT3	0.256335717	2.03657E-43
HMOX1	0.376992488	2.14104E-43
HMGCS2	0.238046738	6.12261E-43
PDE3A	0.272407086	7.68409E-43
KCNB1	0.081549606	1.0343E-42
HS3ST6	0.06578894	1.11189E-42
PYGM	0.07372121	5.90202E-42
SLC17A8	0.027290362	1.07967E-41
BTD	0.567920829	1.48014E-41
SLC20A1	0.398796028	1.91562E-41
SLC2A4	0.170440359	2.27298E-41
GRIK5	0.148975821	2.46009E-41
NAT2	0.339667533	6.73785E-41
MAN1A1	0.429951216	7.14854E-41
EPHX2	0.393522558	1.13574E-40
MSRB3	0.193995178	3.8689E-40
KCNMB1	0.115095297	5.15787E-40
ACSF2	0.43148346	2.10471E-39
ABCD2	0.264967185	3.70893E-39
NAT1	0.401576355	6.77439E-39
SLC22A17	0.328593302	7.62968E-39
ASPG	0.160812949	1.14042E-38
CYP4B1	0.15774989	1.77745E-38
GSTM2	0.384732322	2.35928E-38
ATIC	2.29004131	2.93131E-38
RYR3	0.207130809	4.48238E-38
GRIA4	0.172191427	5.11265E-38
SLC1A1	0.309488405	6.5939E-38
IDH3A	0.575512798	7.21793E-38
PLA2G5	0.195807766	1.18502E-37
GPX3	0.221587675	1.23741E-37
SMPD3	0.440022014	2.60013E-37
PIK3CG	0.304785384	1.0193E-36

AOC3	0.172347872	4.13033E-36
SCNN1G	0.056897752	4.52975E-36
AADACL2	0.047207242	4.58647E-36
PIP5K1B	0.523029369	6.39071E-36
ATP2B4	0.232668136	1.18186E-35
SLC35F1	0.239865728	1.7279E-35
MGST3	0.598984491	3.19524E-35
CLCN2	0.461589106	4.35877E-35
LIPH	0.453219658	4.90165E-35
PLA2G2C	0.110734604	5.33537E-35
ATP2B1	0.520572038	1.46548E-34
CYBRD1	0.263572209	1.67632E-34
MFSD11	0.622202716	3.29061E-34
ADH1B	0.065580653	4.19894E-34
NPR1	0.330346205	1.93675E-33
DPYD	0.326846903	2.1427E-33
CA14	0.247751362	2.3468E-33
ZADH2	0.596227282	2.43052E-33
SLC25A42	0.511667592	5.57257E-33
SLC46A3	0.44971786	6.12918E-33
NMNAT1	0.594256364	1.41534E-32
ACBD6	2.078203826	6.79712E-32
IMPA1	0.507340384	9.40198E-32
PLD1	0.492692124	1.20893E-31
GNPTAB	0.578688731	1.58118E-31
ENOPH1	2.095215452	2.02661E-31
NAT10	2.353534065	2.76635E-31
EHHADH	0.496703546	3.7039E-31
AMPD2	2.32827815	6.91069E-31
HK2	0.460835542	8.88256E-31
ADCY2	0.23777518	1.11767E-30
PYCR1	3.706170393	1.23576E-30
TKT	2.435689737	1.32903E-30
KCNK10	0.320497033	1.90748E-30
SLC6A9	0.438260483	2.37008E-30
HMGCL	0.612751501	3.64449E-30
SLC30A4	0.385828304	5.74797E-30
HVCN1	0.425062575	7.18812E-30
ABHD6	0.584811838	8.72277E-30
MCOLN2	0.333045882	1.04695E-29
NAT9	2.43891934	1.22504E-29
ACSS2	0.569567395	2.02484E-29
ABCA6	0.189185824	2.27542E-29
PDE1A	0.337695814	4.12803E-29

HSD3B2	0.021204049	5.3871E-29
HTR3A	0.182872219	6.23288E-29
GBA	0.626015336	8.27562E-29
PDE4D	0.396219243	1.05318E-28
KLB	0.244964919	1.09986E-28
DCTD	1.746363884	1.11953E-28
CAD	2.967074311	1.46035E-28
AQP7	0.294076948	1.85119E-28
ST3GAL3	0.546471691	3.47375E-28
ITPR1	0.382622981	3.81174E-28
SLC31A2	0.448551733	4.90625E-28
KCNA1	0.073215871	5.87248E-28
NOS1	0.110667975	6.00804E-28
PLD5	0.11159129	6.51302E-28
ATP6V0D1	0.599266682	9.43342E-28
ATP6V1D	0.646342777	1.38534E-27
SLC8A1	0.336872218	1.54162E-27
SLC44A1	0.634607869	1.70084E-27
GMPS	1.989177029	2.16919E-27
PLCD4	0.371580154	2.5397E-27
SLC25A4	0.56938368	2.88688E-27
ACAA1	0.608549954	3.27515E-27
MOGS	1.628464349	4.0525E-27
COX7A1	0.358444614	4.15747E-27
CACNA2D1	0.257187516	5.53219E-27
ATP8A2	0.305665324	6.44128E-27
ALAD	0.659176192	7.45066E-27
CLIC2	0.401387141	9.24319E-27
CHRNA3	0.262309134	1.16523E-26
B3GNT5	0.542798975	1.26071E-26
SLC12A8	2.400356902	1.72292E-26
HADH	0.594524858	1.86021E-26
GART	2.114652019	1.93942E-26
GRIA3	0.240357919	2.36403E-26
PDE1C	0.367602481	2.74955E-26
GRIN2D	34.94927402	3.42697E-26
CKMT1B	0.501304917	4.20633E-26
TXNRD3	2.454827535	4.40817E-26
CAT	0.604152564	4.97962E-26
PPA1	2.47377807	6.05027E-26
ATP1B3	0.629886346	7.22448E-26
GDE1	0.667115661	7.76345E-26
KCNK3	0.151007219	7.93502E-26
PLA2G10	0.405609224	8.98028E-26

SLC3A2	2.457288609	9.77573E-26
ITPKA	0.426746081	1.02759E-25
OGFOD1	1.672333134	1.54259E-25
IMPDH1	3.37654126	1.54472E-25
UQCRFS1	0.624918545	1.58895E-25
PAICS	2.596394123	2.1617E-25
ST8SIA1	0.322289751	2.66223E-25
ABCB1	0.341090336	3.1402E-25
SV2B	0.268905436	4.24779E-25
UMPS	1.670840183	5.10632E-25
GCNT4	0.254141039	5.36257E-25
MPI	0.606764329	6.56976E-25
KCTD12	0.389945175	6.85583E-25
PPAT	3.137169884	6.8677E-25
NANS	0.614791132	9.15846E-25
B3GNTL1	3.149147375	1.07537E-24
PIGS	0.656862451	1.10069E-24
ATP8A1	0.43378017	1.34486E-24
UAP1	0.620696255	1.96211E-24
NIT2	2.110763324	2.49722E-24
CACNA1H	0.297266534	3.07002E-24
SLC38A7	1.858936162	3.55938E-24
GLOD5	0.452890258	4.17324E-24
NIT1	0.699327463	1.25125E-23
GALM	0.605045916	1.364E-23
ENTPD3	0.234658987	1.45918E-23
CPOX	1.962238289	2.11473E-23
PHYH	0.555915293	2.3094E-23
ABCF2	1.636444013	2.43225E-23
DOLPP1	0.632844646	2.54631E-23
SLC17A5	0.591540253	2.67674E-23
NUDT10	0.284262253	2.89108E-23
ITPKB	0.546054579	4.30968E-23
ADCY6	0.621347139	5.78577E-23
GNPDA1	2.122591447	6.00414E-23
SLC23A3	0.331760057	6.44024E-23
A1CF	0.47110956	6.53888E-23
RPIA	2.013862626	7.43949E-23
TRPM4	0.538103804	9.04041E-23
ALDH6A1	0.556177704	9.39519E-23
SLC13A2	0.24598743	1.04992E-22
PGAM5	2.226097016	1.17921E-22
PANK3	0.538237958	1.20105E-22
SORD	2.858443111	1.38517E-22

ST6GALNAC3	0.414130649	1.39428E-22
GRIN3A	0.305111461	1.66264E-22
NME1	3.502393241	2.3041E-22
SLC8A2	0.15777178	2.6267E-22
NUDT5	1.867615797	3.56173E-22
SLC22A4	0.372737692	3.64842E-22
DSEL	0.363395384	3.90154E-22
MSRA	0.587976814	3.92305E-22
ELOVL6	0.561391281	3.93219E-22
ARSA	0.586808364	4.2462E-22
CNGA1	0.375495739	5.26687E-22
SGPP1	0.557323027	6.18559E-22
EBPL	2.420016652	6.67153E-22
DPEP2	0.418294545	7.02042E-22
MGAT4C	0.102975182	7.48571E-22
MTHFD2	2.797900923	8.62012E-22
AFMID	2.041169981	1.00764E-21
IMPDH2	1.994879372	1.0578E-21
SLC39A10	4.060651619	1.08406E-21
ATP6V1F	2.085332	1.66541E-21
AUH	0.673515014	2.47497E-21
MBOAT1	0.565373173	2.47698E-21
SLC27A6	0.156368892	2.65034E-21
SMPD4	1.670101227	2.9019E-21
SLCO4A1	12.26230892	3.51721E-21
DGAT2	4.14048577	3.79428E-21
ALG3	2.278938082	7.17293E-21
SLC35B3	0.671966855	7.63581E-21
SLC3A1	0.376870697	1.01308E-20
CHPF	3.704819296	1.08345E-20
SLC18A2	0.434328107	1.08732E-20
SDHA	0.63434106	1.16317E-20
SLC7A5	9.066379614	1.24414E-20
PCBD1	1.771530841	1.26451E-20
GOT1	0.644830579	1.7652E-20
SLC7A14	0.086150648	1.76937E-20
TTYH3	3.324884562	1.97634E-20
KCNG3	0.261931665	2.00145E-20
ACADSB	0.58735651	2.30101E-20
ME2	0.59467573	2.65566E-20
GALNT6	4.109863346	3.07951E-20
SLC39A5	0.491474717	3.37232E-20
MAOB	0.223574216	3.61614E-20
SLC35C2	1.934657322	4.28679E-20

SLC35A3	0.591588034	4.43207E-20
FUT1	8.054595681	4.56459E-20
RRM2	2.814885612	4.75455E-20
SFXN3	2.216555694	4.9165E-20
LPCAT4	0.603521952	5.28836E-20
TRPC7	0.239768259	6.31966E-20
SLC5A6	4.014943676	6.74999E-20
NDST1	0.640146313	7.47594E-20
ABCC1	2.282946696	7.82446E-20
SLC17A1	0.225150679	7.88404E-20
CYP27B1	4.215039183	8.96972E-20
GLA	2.254618424	9.07988E-20
CYP2C18	0.273935055	9.28491E-20
SHMT2	2.993167994	1.04822E-19
SCLY	2.903191979	1.07116E-19
NUDT16	0.676377	1.20491E-19
DPEP3	0.227164092	1.25166E-19
CYP4F12	0.494890832	1.36034E-19
EPHX4	23.81993321	1.83996E-19
ENTPD8	0.246517211	2.17885E-19
FECH	0.582977231	2.2135E-19
SLC44A2	0.714586441	2.26493E-19
FLAD1	1.801023794	2.51746E-19
SLC15A2	0.506140339	2.98299E-19
CHRNA1	0.331596494	3.29298E-19
MICAL2	0.652337447	4.00917E-19
DTYMK	2.263801676	4.30008E-19
FPGS	2.231333142	4.50951E-19
ATP6V1E2	2.386601544	6.1272E-19
ETNK1	0.522078745	6.20346E-19
AGL	0.61528463	6.24409E-19
ATP11A	5.005483175	7.47626E-19
ACLY	1.731612229	7.49379E-19
DPM2	2.033595011	7.54985E-19
DHCR7	2.774469931	7.97098E-19
SLC7A6	2.510743056	8.41496E-19
SMOX	5.487254283	1.04531E-18
ABCB5	0.115022144	1.1891E-18
TPH1	0.10997928	1.21292E-18
SLC22A3	4.337269752	1.38455E-18
ENOX1	0.40639658	1.38871E-18
SUCLG1	0.662794463	1.44332E-18
ST3GAL2	1.996034158	1.71621E-18
DHODH	1.746062511	1.74788E-18

PECR	0.592774238	1.84514E-18
AHCY	3.429210141	2.11017E-18
EXT1	0.701531886	2.47104E-18
ADO	1.539898861	2.70159E-18
KCNJ14	4.343718637	2.8984E-18
SRM	3.000570228	3.19878E-18
PSPH	2.935461957	3.57293E-18
TPO	0.301054955	3.78642E-18
SLC46A1	0.499718027	3.85332E-18
ABCE1	2.062536775	4.08129E-18
PSAT1	7.30769922	5.04957E-18
NEU3	1.902804092	6.17412E-18
UGT1A1	0.160493919	6.19006E-18
MTHFD1	1.759790996	6.31228E-18
SLC4A2	1.888186549	7.0727E-18
COX19	1.802685603	9.91674E-18
CHID1	1.636530686	1.19304E-17
ECH1	0.636200886	1.21102E-17
NSDHL	2.237428359	1.3101E-17
ST6GALNAC1	0.396790952	1.68643E-17
ENPP2	0.400889009	1.70979E-17
DPAGT1	1.585112898	1.76711E-17
ADCY3	2.272086598	1.93845E-17
TRPC6	0.45549444	2.22413E-17
GPI	1.692835924	2.2334E-17
B3GALT6	2.121012843	2.81119E-17
HLCS	1.683286117	3.07961E-17
GBA2	0.678017133	3.09888E-17
MTHFR	0.639763026	3.15792E-17
BDH2	0.573193958	3.27202E-17
KCTD14	2.435890501	3.58987E-17
SLC25A24	0.627328675	3.71266E-17
G6PC3	1.893698377	3.75062E-17
CACNB2	0.408679534	4.07069E-17
NANP	2.630741458	4.14675E-17
SCD	5.369784869	5.73502E-17
NAALADL1	0.12467908	6.34473E-17
POMGNT1	1.633122214	8.18861E-17
CLCNKB	0.129421285	8.22848E-17
AGK	1.627973916	8.33507E-17
ENPP1	0.48460639	8.5453E-17
SLC6A6	8.277392571	8.69219E-17
GABRG2	0.135253618	9.52521E-17
SLC25A22	2.379919716	9.78033E-17

BDH1	0.648619252	9.84764E-17
AQP11	0.509396829	1.20017E-16
NMRAL1	1.949846354	1.39032E-16
GPT2	3.535258014	1.53796E-16
PYCR2	1.619903241	1.59915E-16
OGDH	0.72438206	1.67471E-16
HDC	0.410253233	1.69166E-16
PRDX4	2.559405382	1.74428E-16
PYROXD1	0.641538674	1.86043E-16
PTDSS1	1.923785777	1.93442E-16
ODC1	2.213369699	2.45753E-16
MFSD10	1.998497919	2.49055E-16
SLC15A4	1.55361266	2.62855E-16
INPP5K	0.684758201	2.75039E-16
SLC25A15	2.060806518	2.80136E-16
TALDO1	1.605689173	3.24E-16
SLC20A2	0.638010607	3.45503E-16
MTAP	2.031663404	3.55826E-16
FXN	1.799266587	4.1413E-16
CRYZL1	0.698983724	5.15482E-16
SYNJ1	0.639632082	5.45033E-16
PISD	1.500897518	6.45377E-16
SLC25A32	2.215142875	6.52978E-16
SLC41A3	1.626710782	7.47774E-16
GRIN2A	0.177606366	7.94996E-16
NUDT12	0.561127009	8.28989E-16
IMPA2	0.609603046	8.29318E-16
SLC25A14	1.977672462	8.80906E-16
SLC7A1	2.432539855	9.11609E-16
HYAL3	2.793153326	9.28814E-16
HIBCH	0.672127912	9.54849E-16
ELOVL4	0.368789668	1.04966E-15
GSTM4	0.592382844	1.06087E-15
SEPHS1	1.469670533	1.06384E-15
ABCA5	0.468335712	1.06682E-15
NDUFS1	0.738396354	1.07005E-15
CYP2S1	2.821139683	1.07226E-15
KCNT2	0.309219401	1.31985E-15
MOCS3	2.281893649	1.34495E-15
PRPS1	1.96524842	1.43614E-15
TECR	1.514698255	1.51558E-15
ACOT11	0.602769409	1.59311E-15
NQO2	2.05583973	1.66064E-15
SLC6A19	0.032326436	1.71089E-15

NUDT1	2.37725936	1.76566E-15
SLC25A12	0.710696916	2.48818E-15
SLC19A1	2.141940923	2.69564E-15
CDS1	0.667541487	2.71887E-15
PNPO	1.744468365	2.74106E-15
SLC5A11	0.107666018	2.97741E-15
GSTP1	2.517404402	3.73064E-15
HSD17B7	2.078072102	3.86017E-15
SLC25A39	1.831402184	3.89699E-15
TPMT	0.669140801	4.14171E-15
SLC23A1	0.166755149	4.35707E-15
ENO1	1.878646454	4.39444E-15
CTPS2	1.924943352	5.06688E-15
ALG8	1.74074654	5.35237E-15
INPP5E	1.852968272	5.47672E-15
CLIC1	1.510146811	5.4839E-15
SULT2B1	9.869095528	5.54692E-15
RFK	0.601745465	5.59898E-15
KCTD13	1.825387417	5.92293E-15
PLCXD3	0.160720195	6.14918E-15
SLC35B2	1.668213481	6.83575E-15
ABCA10	0.357128525	7.09318E-15
OASL	0.425087151	7.52988E-15
SLC25A19	1.643716676	7.92814E-15
B4GALT2	1.551512469	8.23597E-15
TGDS	2.435135969	8.55864E-15
NFS1	1.754597284	9.1836E-15
HMBS	1.81211437	9.63245E-15
PIGU	2.029935443	1.10544E-14
GPD2	0.701494534	1.13355E-14
ABCC8	0.249842259	1.2262E-14
KCNMB2	0.251528441	1.35357E-14
ADH5	0.692953962	1.53051E-14
DGKZ	1.836131726	1.69589E-14
SLC29A3	1.704270088	1.97623E-14
PFAS	1.841606706	2.27169E-14
SLC25A17	1.556560218	2.28656E-14
ACADVL	0.68538556	2.55463E-14
CYP11A1	0.31557856	2.94771E-14
ETFA	0.686383578	2.97847E-14
ASNS	2.941625864	3.26132E-14
ABCC10	1.853195294	3.34219E-14
KCNAB1	0.319368587	3.49858E-14
NUDT19	1.734631791	3.62706E-14

PRDX1	1.526502445	3.72879E-14
DECR1	0.702790526	4.25389E-14
GLRX3	1.859212602	4.79321E-14
SLC35A4	0.754892055	4.85681E-14
NNT	0.701924962	5.11563E-14
SQLE	3.229449623	5.23048E-14
DERA	0.681228218	5.27517E-14
UXS1	1.416103891	5.4667E-14
PLCG1	2.274978216	6.25775E-14
UCKL1	2.708654693	6.29942E-14
CYP26B1	0.430618865	6.44838E-14
ALG1	1.517920013	7.90624E-14
KCTD8	0.168348518	7.915E-14
ACAD9	1.389711601	8.2671E-14
SMS	1.763331744	8.92502E-14
CKB	0.402975833	8.95526E-14
SLC35E4	3.077919314	9.56566E-14
NT5DC2	3.453113833	9.66797E-14
PPOX	1.76806968	9.85989E-14
INPP5D	2.277410776	1.02054E-13
ENOX2	1.908608222	1.03167E-13
MAN1C1	0.514281281	1.07887E-13
CERK	0.648048874	1.13621E-13
NDUFB1	0.66799373	1.22935E-13
BPNT1	0.67300307	1.27729E-13
ITPA	1.868756092	1.32184E-13
SLC18A3	0.176233698	1.39872E-13
KCTD2	0.766565554	1.46483E-13
SLC30A8	0.184839413	1.56178E-13
B4GALNT3	0.56782727	1.60497E-13
STARD9	0.450272943	1.91727E-13
SCN1B	0.487464591	2.16277E-13
TSTA3	2.540163821	2.25252E-13
SLC1A5	2.452464018	2.48219E-13
SLC29A2	1.830188666	2.49893E-13
SLC35D2	0.708338562	2.5151E-13
TRPM2	3.327212866	2.6472E-13
TRPC1	0.46315617	2.75671E-13
FASN	2.914656098	3.06074E-13
HPRT1	1.873367329	3.13016E-13
SMPDL3B	0.6418032	3.59075E-13
GALK1	2.821177889	3.90419E-13
CYP4V2	0.614401232	3.99533E-13
ACOT9	1.655056279	4.00057E-13

SLC43A1	2.468213194	4.04903E-13
GRIA1	0.217722726	4.44934E-13
SLC19A2	1.98774281	4.50594E-13
SGPP2	0.633858063	4.5187E-13
HYAL2	1.91210784	4.79082E-13
NDUFA5	0.670378343	5.05322E-13
SLC12A9	1.877263552	5.49346E-13
ACYP1	1.784498521	5.71197E-13
VKORC1L1	1.460420111	5.96964E-13
PTGR1	0.544371715	6.18583E-13
ADK	1.978633275	6.32849E-13
KCNH1	0.428092708	6.88263E-13
KDM1A	1.914274907	6.88541E-13
COX5A	0.695172479	6.94554E-13
ATP13A1	1.528989662	7.29957E-13
XYLT1	0.622109506	7.99944E-13
SCN4A	0.275259635	8.88388E-13
ATP8B2	0.526870475	8.8929E-13
PEMT	2.013264326	9.21646E-13
FRRS1	0.56121916	9.92175E-13
GPX2	2.325637345	1.00517E-12
GLS2	6.252004817	1.1213E-12
CHRNB4	0.349340502	1.28375E-12
EXTL1	0.340738236	1.33368E-12
PGD	1.62671478	1.3571E-12
HSD17B10	1.966481771	1.48326E-12
GABRG1	0.110314024	1.67151E-12
HPSE	0.54806939	1.83723E-12
SLC25A29	2.650363101	1.86232E-12
SLC38A5	4.481969194	1.91573E-12
SLC35A1	0.642762114	2.04269E-12
SLC12A2	2.966358964	2.13952E-12
KCNN4	3.305131136	2.17784E-12
PRPS2	1.673381651	2.1875E-12
SLC1A4	1.806409116	2.29934E-12
ALDH4A1	3.989154845	2.39887E-12
FAAH2	1.94589192	2.46171E-12
ABCB6	2.528284432	2.62376E-12
PDE4C	0.478196161	3.02493E-12
PIP5K1C	0.724194559	3.19063E-12
ENGASE	3.079256025	3.42674E-12
MECR	1.430385342	4.50976E-12
SLC29A1	2.283430238	4.75808E-12
GLT8D1	1.343707293	4.95586E-12

NDUFC1	0.741667379	5.45578E-12
SDHB	0.738350459	5.83949E-12
COX7C	0.738185168	6.78691E-12
PLD4	0.488583806	6.97275E-12
ADPGK	1.424179243	7.66038E-12
UGGT2	2.477892159	8.71234E-12
KCNK5	0.626776806	9.32117E-12
COQ10B	0.743992294	1.02728E-11
SRD5A1	1.756953541	1.02807E-11
PC	0.553028633	1.03063E-11
CLCN7	1.771155231	1.17089E-11
NAA20	1.923927767	1.23903E-11
PMM1	0.68370189	1.2869E-11
MT-CO1	0.634437037	1.3173E-11
THNSL1	1.785668028	1.41006E-11
UST	0.380654224	1.45865E-11
ATP2C1	1.372020125	1.46872E-11
DPM1	1.971245752	1.51795E-11
KCNS3	0.535290417	1.60043E-11
SLC39A13	1.561135514	1.98462E-11
LRAT	0.108179423	1.98484E-11
VDAC1	1.411388465	2.01251E-11
PTEN	0.69152391	2.1952E-11
CACNA1D	2.874951077	2.237E-11
B4GALT1	0.728267706	2.23706E-11
PTGDS	0.42876526	2.26645E-11
SLC35A2	1.499266391	2.29149E-11
TK1	2.040412039	2.32351E-11
MDH1	0.764027927	2.42304E-11
ABCC3	0.658117667	2.46955E-11
SI	0.133540496	2.7096E-11
NUDT7	0.686040259	2.81715E-11
UQCRC2	0.752444064	2.87618E-11
POFUT2	1.412836645	2.89604E-11
MDH2	1.420054141	2.99553E-11
COASY	1.413791859	3.06889E-11
SFXN4	1.511681878	3.35446E-11
PCK2	0.659090901	3.40216E-11
PGK1	1.786127556	3.79201E-11
CHST15	0.505388764	3.83899E-11
DDOST	1.389838641	3.92303E-11
NAPEPLD	0.703989372	4.04315E-11
THEM4	1.662920498	4.04903E-11
MBOAT4	0.498516921	4.30627E-11

ELOVL5	2.077947762	4.70281E-11
B4GALT7	1.798260354	5.01057E-11
NUDT9	0.770282848	5.09401E-11
AASDHPPT	1.577965005	5.52666E-11
DOLK	1.390495835	5.61565E-11
KCNQ1	2.203650476	5.7437E-11
SLC17A9	5.628403453	5.79257E-11
APRT	1.678242381	6.00055E-11
ACAD8	0.799693617	6.76428E-11
SLC39A6	1.841134333	6.79535E-11
DGKA	0.617632067	6.83435E-11
LDHA	1.568109438	9.25774E-11
SLC25A26	1.40745818	9.39261E-11
SLC16A1	0.659796867	9.60811E-11
AKR1C2	0.366479354	9.61315E-11
SLC4A10	0.136969707	9.8165E-11
PIGL	1.629597592	1.1302E-10
SCN3B	0.293210345	1.23717E-10
KCNE3	1.824371496	1.28963E-10
CLCN5	2.222173096	1.32992E-10
CRLS1	1.847375357	1.4295E-10
ACBD7	4.526453451	1.47909E-10
TTYH2	0.575166314	1.50224E-10
SPTLC3	0.502020389	1.57699E-10
SLC2A5	0.382334568	1.58115E-10
SFXN1	0.720843771	1.62078E-10
ALAS1	0.748816419	1.75849E-10
PTGES3	1.481035277	1.7805E-10
PIK3CD	0.537695865	1.78658E-10
NPR2	0.660738889	1.82536E-10
GABRE	5.778918692	1.85947E-10
MFSD1	0.728404024	1.88892E-10
HGD	0.510194173	1.90171E-10
GALNT7	0.633991027	1.90897E-10
EDEM2	1.505508446	1.91904E-10
PCYT1A	0.804639216	2.08282E-10
NUDT14	2.048638296	2.1157E-10
B4GALT3	1.462320413	2.13163E-10
ACSBG1	0.519119245	2.33397E-10
BST1	0.498278477	2.34216E-10
ADH6	0.442996107	2.38713E-10
CLCA1	0.186802602	2.63985E-10
TM7SF2	2.200020127	2.64476E-10
SPNS1	1.670599118	2.67354E-10

G6PD	1.77853677	2.72762E-10
ACACA	1.803561595	2.75334E-10
HAGHL	12.75988177	2.83166E-10
GSTCD	1.665346813	2.84057E-10
ALDH3A2	0.695020761	2.85562E-10
SEPHS2	0.709660121	3.0794E-10
ST8SIA4	0.54082576	3.378E-10
DAGLB	1.324768432	3.57444E-10
PDE7A	1.886840119	3.63283E-10
KCNH8	6.240322612	3.83387E-10
MCAT	1.567789144	3.85414E-10
RRM1	1.455691023	4.00827E-10
ADSL	1.578492209	4.1601E-10
LPCAT1	2.360319095	4.21391E-10
LIPG	2.69608212	4.38567E-10
NAGA	0.78336407	4.5575E-10
ACSM1	0.511352691	5.02836E-10
SLC24A4	0.437666997	5.29562E-10
UEVLD	0.748643294	5.34207E-10
SLC35B1	1.378226478	5.37146E-10
ECHDC1	0.746541256	5.76918E-10
LDHB	2.068843303	5.7771E-10
MCCC2	1.444609533	6.6422E-10
SLC39A1	1.35624329	6.95955E-10
MGAT2	0.699361424	7.88728E-10
COMT	2.379286853	8.45304E-10
PDE3B	0.648632409	8.50891E-10
GLO1	1.809175249	8.75621E-10
UGCG	0.701917961	9.03153E-10
ST8SIA6	0.457013266	9.37008E-10
AGMAT	1.735374909	1.02225E-09
MANEAL	2.242367728	1.10457E-09
SLC27A2	0.668693654	1.13045E-09
ATP8B4	0.506553063	1.1629E-09
GNE	0.555693234	1.21933E-09
MGAT5	2.11673082	1.28933E-09
ABHD11	1.576753125	1.36591E-09
HS2ST1	1.579807305	1.52067E-09
EBP	1.716968229	1.54525E-09
QDPR	1.394569166	1.68956E-09
GAPDH	1.774524515	1.75705E-09
SLC11A2	2.385477948	1.77062E-09
PIGN	0.727745887	1.85686E-09
GDPD5	7.932594483	1.89229E-09

PLD6	1.850604675	1.89797E-09
ASNSD1	1.403242262	1.95885E-09
GSS	1.563271799	1.9733E-09
CACNA1A	0.420251354	2.00628E-09
HKDC1	1.92777303	2.1402E-09
PGS1	1.419668327	2.1698E-09
NQO1	2.205388945	2.26802E-09
THTPA	0.748500261	2.32239E-09
ENO3	2.035465387	2.32433E-09
CHRNA5	2.057305441	2.39755E-09
MTHFSD	1.35008288	2.43239E-09
STARD8	0.605378696	2.63425E-09
GABRD	8.276061343	2.6422E-09
PFKM	1.505107086	2.65324E-09
SLC25A35	0.704668166	2.74831E-09
PGM3	1.505640581	3.01369E-09
PIGW	1.667157006	3.09078E-09
HPDL	2.866329115	3.16188E-09
MANBAL	1.440272658	3.42251E-09
B3GNT6	0.321028996	3.43388E-09
SCNN1A	0.638280668	3.5553E-09
UGGT1	1.410429075	3.61205E-09
ATP6AP1	1.412213813	3.72678E-09
HS6ST2	11.54475488	3.72746E-09
B3GAT3	1.455194854	4.12883E-09
FLVCR1	1.675781683	4.23729E-09
NUDT21	1.423807866	4.23996E-09
NUDT11	0.398891977	4.45777E-09
PTDSS2	1.74575833	4.61689E-09
RDH11	1.541522625	4.7915E-09
DHRS1	0.700668703	4.91421E-09
SLC2A8	2.219563966	5.02642E-09
GPLD1	0.509958032	5.0953E-09
MAN2B1	0.756683371	5.11642E-09
POFUT1	1.993136077	5.35002E-09
PTGES2	1.786434776	5.58054E-09
CACNB1	0.512398891	5.66411E-09
AK3	0.755278605	5.88018E-09
GANAB	1.285886427	5.99732E-09
INPP5J	0.614230421	6.04983E-09
STARD5	0.712148792	6.1134E-09
SLC9A7	3.224208234	6.14114E-09
ATP6V1C2	5.680306326	6.24479E-09
PI4KA	0.781608663	6.87741E-09

GSTO2	1.8371946	7.37047E-09
ST3GAL4	0.479074351	7.44408E-09
PTGIS	0.386477425	7.49812E-09
UROS	1.39804772	8.43957E-09
SLC16A13	1.678356752	8.85777E-09
GSTM1	0.410967745	8.98357E-09
KCNK2	0.28358803	9.36814E-09
CYP2U1	0.676974394	9.58472E-09
CYCS	0.70599576	9.95362E-09
ACOT13	0.722349439	1.0105E-08
AKR1E2	2.912436822	1.02051E-08
GLB1	1.339183192	1.06687E-08
AADAT	2.309488453	1.08212E-08
HEPH	0.721029287	1.14735E-08
DHRS4	0.721572079	1.14808E-08
ABCG1	0.675106576	1.16519E-08
PAPSS1	1.339161305	1.19256E-08
NDUFB3	0.757374907	1.19912E-08
ACSL4	1.921560926	1.2323E-08
CLYBL	0.692535463	1.25989E-08
ALG5	1.670097125	1.28572E-08
CANT1	0.749814554	1.33949E-08
B3GALNT2	1.565927394	1.34418E-08
SLC37A2	0.28465265	1.42544E-08
GNPNAT1	1.626112555	1.43337E-08
SLC38A4	0.354227574	1.47463E-08
PLA2G15	1.366546875	1.48505E-08
CYB5B	1.728207706	1.49721E-08
LIPM	2.0797683	1.51821E-08
CYP2J2	0.660138487	1.53986E-08
ABO	0.585068809	1.58093E-08
IP6K3	0.365102446	1.58608E-08
MICAL3	0.678766605	1.6288E-08
GCSH	1.704416177	1.80287E-08
WWOX	1.582642444	1.81445E-08
RDH10	1.803225414	1.82154E-08
CRAT	0.585060788	1.8823E-08
GOT2	1.342869542	1.96813E-08
INPPL1	1.305707208	2.03605E-08
KCTD10	0.798848313	2.07156E-08
MAN2A1	0.734982355	2.09233E-08
LIPI	0.283711584	2.26218E-08
PIK3R5	0.558677358	2.32961E-08
TPK1	0.671794932	2.41797E-08

SLC27A4	0.740478207	2.49194E-08
CHI3L2	0.402441769	2.52264E-08
COX10	0.790381171	2.58026E-08
KCND3	0.475090099	2.59791E-08
PIK3R2	1.703274536	2.63161E-08
MAN1B1	1.323196617	2.71501E-08
GPX4	1.678376895	2.74746E-08
NDUFS4	0.760383411	2.79363E-08
PIGX	1.40782143	2.84707E-08
ABCB8	1.423132166	2.96046E-08
SLC39A3	1.76302285	2.97968E-08
SLC41A1	1.662892762	3.01027E-08
CHDH	1.589188791	3.13932E-08
ACOT4	0.632480654	3.35048E-08
PIK3C3	0.746985497	3.40958E-08
VDAC2	0.790912589	3.571E-08
SLC6A8	0.557146429	3.58444E-08
UQCR10	0.727174587	3.59021E-08
SLC15A1	0.198590449	3.73751E-08
EPHX1	0.631718348	3.87504E-08
KCNC1	0.287308542	4.06611E-08
DGUOK	1.365712638	4.18601E-08
CLCN4	3.650202488	4.28209E-08
XYLT2	1.374769646	4.34274E-08
P2RX7	0.535439052	4.3892E-08
COX8A	0.750899187	4.51655E-08
PAOX	0.71033265	4.53988E-08
NDUFS2	0.808595336	4.65365E-08
NAA50	1.389749916	4.76487E-08
KCNAB2	1.856798182	4.78101E-08
ME1	2.139114627	5.38966E-08
FOXRED1	1.42760658	5.71772E-08
ABCF1	1.282825099	5.81739E-08
PDXK	1.482287006	5.83834E-08
PCYOX1L	1.584257957	6.14781E-08
FADS3	2.193632911	6.47911E-08
TRPC4	0.536087698	6.55222E-08
MVK	1.452838066	6.72252E-08
TYRP1	0.452003052	6.7342E-08
FUT8	1.945810141	7.03302E-08
HMOX2	1.31455883	7.08868E-08
ABHD8	0.681826834	7.68129E-08
GSTZ1	0.686886447	7.68471E-08
PRDX2	1.447532869	7.88217E-08

ACBD4	0.751541332	7.95963E-08
ADA	2.385710483	8.12916E-08
PLCL1	0.475435291	8.18408E-08
SLC26A7	0.448293026	8.21581E-08
RPE	1.427662266	8.36693E-08
ACER3	0.724386136	8.41987E-08
NEU1	1.734283941	8.57522E-08
MOCOS	1.892569538	8.88286E-08
NUDT3	1.335782314	8.91407E-08
KCTD4	0.179482544	9.13885E-08
DBT	0.770987728	9.26764E-08
CYP39A1	2.548724439	9.5102E-08
FAR2	0.742755184	9.5385E-08
SLC12A6	0.734401194	9.66665E-08
NDUFAF2	1.572779756	9.8171E-08
MFSD3	2.132409446	1.0352E-07
NME2	1.502353071	1.04373E-07
SLC4A11	18.81450097	1.10675E-07
GFOD1	0.707134625	1.16352E-07
ABCF3	1.211696986	1.16838E-07
DDT	0.681141028	1.18531E-07
KCNQ5	0.259245251	1.21439E-07
GCAT	1.875764684	1.28407E-07
SLC9A5	2.456928907	1.37587E-07
ABHD4	0.734137631	1.44698E-07
MAT2B	0.78871632	1.45854E-07
SMPD2	1.388137967	1.46878E-07
KCNJ11	2.93491331	1.52822E-07
PHGDH	3.531922252	1.53595E-07
ATP7B	1.911929376	1.53609E-07
ATP6V0C	0.749521595	1.59073E-07
CTBS	0.734013857	1.61533E-07
ACOT2	0.740010489	1.7019E-07
SLC2A1	3.760070801	1.77792E-07
KCNJ5	0.484289009	1.80832E-07
FOXRED2	2.234671081	1.81758E-07
SLC39A12	0.286184417	1.82431E-07
CYP4F3	2.792270351	1.87145E-07
COQ9	0.806924506	1.89358E-07
ST8SIA3	0.120773579	1.91087E-07
PNPLA7	0.583253311	1.9156E-07
NAALAD2	0.430563608	1.92909E-07
NME6	1.224479268	1.9603E-07
MFSD9	0.799887601	1.97995E-07

CLIC4	0.631486192	1.99041E-07
ISCA1	0.778847337	1.99496E-07
SLC22A15	2.519895028	2.0725E-07
CHPF2	1.279503495	2.21819E-07
PIGM	1.382160386	2.23705E-07
HSD17B12	1.491232986	2.41618E-07
SLCO2B1	0.701534753	2.45252E-07
HSD3B7	1.641512346	2.51718E-07
GUK1	1.739412405	2.54202E-07
NDUFV2	0.700945607	2.57806E-07
CMAS	0.777381848	2.60001E-07
GALNT2	1.313856659	2.6484E-07
ORAI1	1.522413486	2.83713E-07
HSDL1	1.359937605	2.84394E-07
DPYSL3	0.545109632	2.90009E-07
FDXR	1.921940653	2.90943E-07
AQP1	0.543210013	2.9338E-07
IPMK	0.702659655	3.13684E-07
SLC25A27	2.651393986	3.27346E-07
DLD	0.805795304	3.31901E-07
SLC25A10	1.652331537	3.33602E-07
CHSY3	2.840527158	3.38511E-07
DIO2	4.520128872	3.57837E-07
HACL1	1.351956476	3.58845E-07
CACNA2D3	0.449718747	3.80251E-07
GSTM3	0.557096832	3.80375E-07
SLCO5A1	3.797839156	3.92777E-07
CYB561	1.373340253	3.98187E-07
GGH	2.53544422	4.02589E-07
SRD5A3	1.746404867	4.11728E-07
CHI3L1	7.976545275	4.15029E-07
ALG2	1.263449381	4.23667E-07
LPGAT1	1.620186514	4.47213E-07
MMAB	1.555961554	4.68967E-07
PMM2	1.36475476	4.95647E-07
AMT	0.634257216	5.13027E-07
HIBADH	1.3855476	5.17915E-07
QPRT	2.323196094	5.29222E-07
NDUFB11	1.412235682	5.33424E-07
PLA2G16	2.369975173	5.39089E-07
KCTD20	1.308044999	5.87347E-07
LPIN3	1.738748225	6.00949E-07
SLC25A11	0.785998067	6.02615E-07
SLC28A2	0.299019794	6.09467E-07

ACSL6	21.50335541	6.29496E-07
PLA2G4B	2.594441045	6.32566E-07
KCTD6	1.4699919	6.75133E-07
LTA4H	1.30764712	7.47358E-07
ATP1A1	0.758347542	7.51646E-07
INPP4A	0.77616999	7.69475E-07
CYP27A1	0.633315175	7.72385E-07
PIGQ	1.478306571	7.79827E-07
PDE1B	0.470453323	7.9429E-07
SV2A	0.560633124	8.27106E-07
FUK	1.409735674	8.27175E-07
PTS	1.378310054	8.42745E-07
DPEP1	7.45398255	8.45403E-07
SLC7A11	6.244442834	8.6709E-07
SLC5A2	2.965433564	8.79439E-07
ENTPD6	1.666320716	8.89381E-07
ABCA1	0.565480915	8.99909E-07
TBXAS1	2.076747514	9.15847E-07
AKR7A3	0.672701184	9.17102E-07
HCN3	1.637562525	9.69989E-07
ALDH1B1	1.68415322	9.82236E-07
PHYHD1	0.421234933	9.83956E-07
GALNT1	0.74800121	1.03589E-06
PFKFB3	1.56008571	1.03832E-06
ATP6V1C1	1.442550875	1.03976E-06
ATP13A2	1.497045798	1.04984E-06
ABCD4	0.811875142	1.17963E-06
PLPPR5	0.412003619	1.18845E-06
PIGT	1.377681042	1.25589E-06
FUCA2	1.269930042	1.28405E-06
MMACHC	1.357940719	1.30779E-06
LBR	1.475640919	1.33697E-06
LIPA	0.709771771	1.36575E-06
B4GALT4	0.798729593	1.40782E-06
DHRS4L2	0.744322468	1.41914E-06
KCTD18	0.833470664	1.43976E-06
CYB5A	0.73766013	1.46773E-06
NAT14	1.946058661	1.49409E-06
NDUFA1	0.7731418	1.50914E-06
CA11	0.627208213	1.51326E-06
CBR1	0.708884196	1.51556E-06
GCDH	1.299167451	1.51586E-06
NOS3	2.038426524	1.57501E-06
CYB5R2	1.987479176	1.65719E-06

OAS3	1.983099314	1.71104E-06
ABHD10	1.310907999	1.72718E-06
PLCB4	3.044260785	1.80623E-06
GLCE	1.574829876	1.83142E-06
UAP1L1	1.82426019	1.851E-06
CACNA1C	0.502799815	1.87386E-06
PLA2G2D	0.363595892	1.89805E-06
POMT2	1.313735282	1.90323E-06
SLC35A5	0.774336918	1.90919E-06
GYG2	2.120701839	1.93504E-06
MCOLN1	0.759837891	2.05541E-06
ADAL	0.750260591	2.13168E-06
GALNT11	1.272051876	2.1714E-06
SLC4A8	2.829804027	2.17533E-06
GRIK1	0.2798968	2.23444E-06
ABCC9	0.472658102	2.39511E-06
ASAH1	0.784986635	2.43737E-06
FADS1	2.990277661	2.64452E-06
NDOR1	1.46633065	2.70628E-06
SLC30A1	0.760839598	2.73442E-06
GMPPA	1.239659763	2.89118E-06
SGMS2	0.667222431	3.1042E-06
STARD13	0.701605016	3.10523E-06
NDUFA6	0.767119337	3.27346E-06
FUT9	0.134449947	3.39131E-06
GRIN2B	10.14618077	3.44958E-06
COQ7	0.846735144	3.57275E-06
ATP6V1H	1.327599363	3.64028E-06
KCNQ4	0.586346544	3.65517E-06
FDPS	1.356860987	3.68834E-06
ECHS1	0.798299009	3.7867E-06
NOX4	8.072502471	4.07293E-06
PCYT2	1.426290559	4.08323E-06
SLC11A1	5.544442282	4.13785E-06
NUDT15	1.416510793	4.27614E-06
ABHD12	1.520568051	4.5813E-06
SULF2	1.628277414	4.81834E-06
TRPA1	0.48092503	4.84745E-06
ARSE	2.046861222	5.15225E-06
SDR42E1	0.713329533	5.33105E-06
FUT4	1.520763561	5.93857E-06
SRXN1	1.795779889	5.94833E-06
C1GALT1	1.496198345	5.96073E-06
KCNS2	0.26686366	5.96926E-06

DHRS7	0.78806128	5.96962E-06
SLC14A2	0.204923627	6.07346E-06
MAN2A2	1.34795818	6.16128E-06
UGT1A5	0.104283193	6.18223E-06
NADK	0.839078378	6.52175E-06
LPCAT2	1.921074721	6.68323E-06
HEXB	1.268235863	7.14326E-06
TREH	0.251254134	7.1609E-06
SLC28A3	5.103618648	7.41567E-06
CNDP2	0.777963117	7.57546E-06
SLC6A20	5.691288435	7.70703E-06
FMO2	0.304193596	8.09169E-06
ENTPD2	1.952053714	8.10191E-06
IDUA	1.947319406	8.33002E-06
SLC2A12	3.611036188	8.51387E-06
ALDH9A1	0.830909658	8.69192E-06
CSAD	1.603100037	8.88317E-06
IDH2	1.460868175	8.96827E-06
CYP2F1	3.961483092	9.16024E-06
TPI1	1.327377712	9.22696E-06
PPCS	0.824181329	9.24688E-06
SRR	0.764842612	9.3076E-06
FDX1	0.820673792	9.43896E-06
DHRS13	1.537265181	9.59565E-06
CHSY1	1.445784392	9.72911E-06
CHST14	1.376100108	9.8916E-06
GSTA1	0.085686654	9.89406E-06
HS3ST1	1.793071401	1.08024E-05
ASAH2B	1.426023806	1.0922E-05
SLC35B4	1.33115164	1.10952E-05
MAN2B2	0.823306368	1.14319E-05
GXYLT1	1.418338014	1.15728E-05
SLC37A3	1.264710884	1.24859E-05
LPIN1	1.555358283	1.29232E-05
SDSL	0.756757387	1.3201E-05
SLC13A1	0.018981382	1.36869E-05
PCYOX1	0.802831847	1.37022E-05
ADSS	1.252919384	1.3867E-05
SLC10A3	1.364358885	1.40089E-05
COX6A1	0.794503363	1.40916E-05
NME7	1.405214686	1.43272E-05
NDUFB8	0.813991863	1.43404E-05
ATP13A3	1.453866045	1.49266E-05
PIGC	1.291419889	1.54657E-05

PGM2	1.282208576	1.55695E-05
CYB5D2	0.777329921	1.62794E-05
PI4K2B	1.385834706	1.723E-05
AKR1C3	0.584382385	1.77612E-05
CKM	0.437717091	1.7901E-05
SLC39A4	1.988507459	1.80042E-05
KL	0.394158108	1.80373E-05
B4GALNT4	7.503723708	1.90787E-05
ATP2A1	2.644440394	1.94246E-05
SULF1	3.657763598	1.94277E-05
CA10	0.234792167	1.96793E-05
COQ6	0.843714942	2.01829E-05
ENTPD4	0.777490054	2.06531E-05
SPHK1	4.031389889	2.10369E-05
SFXN5	0.780731461	2.12738E-05
GFOD2	0.856335168	2.14289E-05
SLC45A1	0.662927201	2.16166E-05
PLA2G4E	3.712890594	2.2352E-05
ALDH2	0.812660046	2.25186E-05
GAD1	41.90328829	2.29636E-05
SAT2	0.773692202	2.30908E-05
SLC30A6	1.241095725	2.34383E-05
STARD7	1.162870761	2.42701E-05
PIK3R6	0.681897066	2.45029E-05
BCKDHA	0.818435109	2.62752E-05
NMNAT3	1.605916548	2.67208E-05
SLC22A11	27.8330588	2.7768E-05
CDADC1	1.279984538	2.78764E-05
B3GNT4	2.574512773	2.80651E-05
SLC24A3	0.640653666	2.83339E-05
ENPP5	1.716240765	2.8366E-05
CACNG8	12.36834288	2.86753E-05
ARSH	3.720262661	2.88718E-05
PIGA	1.406933178	2.98722E-05
ABHD1	2.56598123	3.10288E-05
GLB1L2	1.417926132	3.13297E-05
ACOT1	0.692168001	3.20441E-05
NME4	1.435650585	3.37997E-05
GLRB	0.558727589	3.38067E-05
SFXN2	1.37206424	3.47509E-05
CDA	0.564358423	3.48829E-05
MANBA	1.302192889	3.60584E-05
PFKFB2	0.727311557	3.63795E-05
GMDS	1.444734973	3.65072E-05

AQP12A	0.480457924	3.68412E-05
MMAA	0.817636162	3.75862E-05
NT5DC4	6.013294963	4.14564E-05
TXNRD2	1.348307106	4.18474E-05
SLC16A4	1.983614703	4.20783E-05
SLC27A1	1.57048985	4.53597E-05
DHRSX	1.364757017	4.69965E-05
SLC10A4	0.300803146	4.78661E-05
CLC	0.40833364	4.97721E-05
ACSS1	1.686249253	4.98727E-05
ALDOC	1.762805361	5.01878E-05
SLC12A7	1.326583356	5.11963E-05
ATP6V1G1	1.270747369	5.21114E-05
LPIN2	0.751764075	5.30007E-05
PLA2G4F	0.704779141	5.39379E-05
UGT2B11	0.273090045	5.42585E-05
ALDH1A1	0.5386285	5.44352E-05
AQP4	0.185632395	5.45545E-05
KCNJ16	0.337712939	5.53711E-05
COX6B2	0.555690299	5.56044E-05
RDH12	2.728580573	5.5621E-05
SLC6A16	0.502054449	5.64691E-05
L2HGDH	1.336248404	5.6772E-05
PDSS1	1.259590736	5.838E-05
HS6ST3	0.322184343	5.86808E-05
IPPK	1.233942609	6.01378E-05
POR	1.405556213	6.08236E-05
GCLC	1.254499085	6.11212E-05
B3GNT9	1.411623513	6.56177E-05
GRID1	0.622129077	6.61208E-05
ABCB7	1.260287031	6.63157E-05
NUDT22	0.818918815	6.92004E-05
GGPS1	1.239745443	7.10928E-05
SLCO1B3	98.76140164	7.41353E-05
NUDT4	1.430115621	7.54192E-05
EXT2	1.16851927	7.68482E-05
ALDH3B2	14.63718021	7.90462E-05
KCTD5	0.857844192	8.01232E-05
PDE6B	0.555477391	8.51967E-05
NOX1	1.877217158	8.52985E-05
PCBD2	1.275339905	8.53059E-05
LYPLA1	1.616893458	8.5397E-05
NIPA1	1.289478575	8.73559E-05
AGPAT3	0.861311504	8.76456E-05

TRPV4	3.487273744	9.07504E-05
HTR3B	0.161844293	9.16065E-05
STRA6	60.72287536	9.46149E-05
FADS2	3.751455362	9.69755E-05
GRIA2	0.269040627	9.85943E-05
CYP27C1	0.543500281	0.000102612
PFKFB4	1.729735047	0.000106169
AS3MT	0.577230723	0.000106433
TH	12.12004669	0.000108011
ABCA2	1.524770229	0.000109721
SLC40A1	0.700168246	0.00011358
ACOX3	0.858116214	0.00011564
SLC27A5	3.502781976	0.000117857
PIP4K2C	0.859326586	0.000119408
APEH	1.243111857	0.0001218
SLC26A1	1.535354077	0.000126718
SULT1C2	5.758670647	0.000130569
ORAI3	1.306869554	0.000131444
MGAT1	0.846355221	0.000133203
SLC13A3	20.89055002	0.000134428
PNPLA1	2.772790084	0.000134514
XYLB	1.420512761	0.000137985
SLC47A1	0.489196469	0.00013902
CYB5RL	1.3464395	0.000139785
PNPLA3	13.38641622	0.00014036
HMGCR	0.796733972	0.000140939
CERKL	0.726741112	0.000144357
ORAI2	1.421729652	0.000145347
AKR1B1	0.704288567	0.000147319
MGAM	0.03567346	0.000149007
EXTL3	1.314961879	0.000152394
FUT11	1.320609416	0.000156089
ENPP4	0.770967696	0.000158244
TMEM104	1.251449566	0.000162093
IDS	0.747579498	0.000162125
LCLAT1	1.253361265	0.000163419
PIP5K1A	1.196068849	0.000164032
ACHE	0.56703427	0.000164398
FUT3	0.775927043	0.000167978
CPT1B	1.844485812	0.000172701
SLC35D3	22.19761778	0.000173054
OAT	0.655889623	0.000178804
DUT	1.319736604	0.000179125
CYB5R3	0.835670878	0.000179543

KCNC4	1.447703716	0.000183705
TYMS	1.480698344	0.000187033
ASNA1	1.307811239	0.000189007
NT5C2	0.841955925	0.000194959
SLC10A2	0.00608321	0.000198974
ESD	1.270388356	0.0002003
CKMT2	12.16693041	0.000200629
FAHD1	0.832652107	0.000201734
FIG4	0.855643239	0.000202938
KDM1B	1.288593022	0.000208165
GCK	0.564903368	0.000211197
COX18	1.315885544	0.000214534
ALG10	1.485493265	0.00021594
VKORC1	1.211835864	0.000227838
ALOXE3	8.128988279	0.000236353
UPP1	0.680961076	0.000242585
MICAL1	1.294468217	0.000255082
KCNA2	0.515013875	0.000260464
GLUD1	0.863562293	0.000271692
GMPR	2.250570123	0.000272179
ACSM3	0.655673616	0.000274967
GCLM	1.311181926	0.000276689
SLCO3A1	1.59446363	0.000280201
HSD11B1L	1.515685193	0.000284686
SLC7A8	1.769635154	0.000286853
CYP3A4	0.061751057	0.000289178
KCNG1	0.453533275	0.000295893
KCNK9	7.494292875	0.000300557
MGST1	0.766970215	0.0003009
B4GALNT1	0.650370162	0.000301802
DCXR	1.417197041	0.000303304
ACE	0.421972087	0.000303904
PIK3R1	0.784746144	0.000306979
SLC5A12	0.094325088	0.000309913
MTR	1.286131711	0.000309921
HS3ST3A1	2.153169232	0.000330044
LYG1	2.376412893	0.000331739
AGPAT1	0.870602273	0.000334933
KCNH4	4.967125068	0.00034824
ACOT7	1.320828514	0.000357259
CUBN	0.040478004	0.000361348
FUT10	1.35152955	0.00037272
SLC25A44	0.875140467	0.000374279
GRHPR	1.254885669	0.000385166

KCTD1	0.674903405	0.000395486
AMPD3	1.333662467	0.000396364
CNGA3	0.149340965	0.0004034
MAT2A	1.197326784	0.000405725
RDH13	1.193741819	0.000416486
CYP4X1	8.216572911	0.00041871
CRYZ	1.406103537	0.00042146
HYI	0.836267496	0.000421484
GLYATL1	9.984847809	0.000422779
IAH1	1.21911642	0.000424849
UCK2	1.178226398	0.000430456
CHST1	2.091333197	0.000436783
D2HGDH	1.488257038	0.000442759
NAA40	1.217181508	0.000448128
FHIT	1.306723272	0.000448642
SLC24A1	1.275450748	0.000454196
ARG2	1.738763514	0.000454871
GSTA2	0.056522246	0.000459247
ATP9A	1.429221817	0.000465457
PRPSAP1	1.154386035	0.000468332
NUDT8	1.728732355	0.000487424
TCIRG1	1.390105503	0.000490791
CATSPER3	1.425672202	0.000492591
SLC39A11	1.255957952	0.000495057
ABCA7	1.49735935	0.000506245
NDUFS8	1.365800907	0.000514862
GRIN3B	3.67307996	0.000515149
HTR3C	0.220898603	0.000516092
GPHN	1.42312036	0.000516772
GNPAT	1.205094654	0.000518477
MCEE	0.838850712	0.000520724
RHCG	6.193180341	0.000526496
CYP46A1	0.563147361	0.000530783
ST3GAL6	0.665831053	0.000534369
SOAT1	0.768595433	0.000545692
LYZL4	20.15970512	0.000549441
CA9	48.97705944	0.000585036
SULT1A3	1.824284799	0.00060133
NDUFS5	1.316038513	0.000632884
GAL3ST2	0.688957938	0.000633518
CLIC3	4.222341407	0.000637775
CYP2R1	1.224193899	0.000647624
GSR	0.803537749	0.000650012
PDE12	1.174710413	0.000667565

GLOD4	0.851360689	0.000694954
AQP6	4.162172759	0.00070256
TPCN1	0.857234403	0.00071978
MFNG	0.627829026	0.000737246
GLRX5	0.872026395	0.000743369
CYP2B6	2.053276058	0.000749365
MFSD5	0.886208416	0.000781623
ECHDC2	0.816981778	0.000783122
ATP9B	0.808220354	0.000784323
GLUD2	0.743816064	0.000827565
TAP1	1.572943258	0.00085542
PTGES	2.129088207	0.000863925
GALK2	1.21803622	0.000882089
COMTD1	1.707184813	0.00089443
NDUFA4	1.279774829	0.000918809
PIP4K2B	1.205690629	0.000954297
NDUFA10	0.8905385	0.00097628
SLC25A6	1.550822122	0.000979724
KCTD15	0.743975483	0.000986787
PPIP5K2	0.805486177	0.000989988
CYBB	0.674449754	0.001045213
PKD2	0.722104328	0.001064272
MVD	1.488531869	0.001081233
SLC2A11	1.245317613	0.001093196
EDEM3	0.778781201	0.001101341
KCNJ13	0.05486817	0.00112811
PIP5KL1	2.302083229	0.001132469
SLC7A9	0.08986862	0.001145191
SLC25A46	0.826256966	0.001154907
AACS	1.20412926	0.001160448
DHFR	1.321443573	0.001172996
CLIC6	0.568199604	0.001189073
CACNG5	0.232989751	0.001197461
IDH3G	1.433366352	0.00119755
SLC6A14	4.983907573	0.001239118
NAGLU	1.207707915	0.001248934
ATP6AP2	1.221009942	0.001254085
AK5	0.406033991	0.001257758
ANKH	1.278531376	0.001281153
CYBA	1.43105254	0.001282929
GAL3ST4	0.774955195	0.001291049
KCNJ2	0.722652971	0.001318014
PLA1A	1.900656539	0.001329702
SAT1	1.3186384	0.001334625

SUOX	0.857199954	0.001334796
PI4K2A	1.138707341	0.00135913
KCNK13	0.719522409	0.001398052
SLC4A1AP	1.165911387	0.001411427
HSD17B14	0.733097256	0.001414414
GPX8	1.603735125	0.001420847
SCCPDH	1.252023205	0.001426773
BHMT2	0.292349181	0.001436763
ITPR3	1.271000472	0.001455633
CEL	77.56061506	0.001458431
ACSM4	0.573853515	0.001468172
GRIK2	0.43503297	0.001497885
LYPLAL1	1.310887713	0.001535977
GLS	1.368949228	0.00157837
AANAT	3.067851439	0.001607242
SLC47A2	0.589908738	0.001617586
AK1	0.776349387	0.001691095
SLC30A5	1.15913974	0.001699352
SLC1A7	2.938926944	0.001703132
NME3	1.34874069	0.001730893
ALDOB	0.137311392	0.001756469
ALDH3B1	1.382529504	0.001781672
GLRX2	1.348340051	0.001791002
ATP6V0A1	0.879000324	0.00181885
GSTO1	1.296508109	0.001843772
FH	0.866460844	0.001850046
CYB5D1	0.802230647	0.001869993
PLA2G3	21.96987121	0.001874304
SLC38A2	1.292715432	0.001907667
AOC2	1.993059701	0.001927264
SLC30A7	1.320503183	0.001930273
AQP12B	0.556419507	0.001940966
KCNB2	0.356651363	0.001954964
ST3GAL1	1.730914361	0.001956527
CYP2D6	0.290923989	0.001975998
UQCRC1	0.826479978	0.001986369
PIP4K2A	0.831358942	0.002033998
ATP10B	0.799850107	0.002054098
SCNN1D	1.714443793	0.00207574
ABCC2	5.212432258	0.002083529
ACAD10	0.867812171	0.002098189
DGKE	1.351779449	0.00211283
FAH	1.27814883	0.002135152
PRDX5	1.375994824	0.002227761

SYNJ2	1.193303752	0.002286823
CYP2W1	17.40864268	0.002288093
CACNG4	12.9080155	0.002298131
NPC1L1	17.48925778	0.00232421
PANK2	1.192273135	0.002344891
ABCC6	0.740176446	0.00240928
GLB1L	1.264982126	0.002429677
COX7A2	0.826426043	0.002431151
EDEM1	0.851522255	0.002547253
CATSPER2	1.542418298	0.002554886
NT5DC3	0.745129088	0.002625814
CATSPER1	2.554249481	0.002674403
TMCO3	1.260595049	0.002676713
NAGK	0.875109151	0.002692007
NDUFA4L2	5.165401709	0.002709476
AKR1A1	1.184395906	0.002733488
OAS2	2.017352305	0.002794544
CHRNA7	0.500469476	0.002826793
SDHC	0.884448262	0.00284232
NUDT6	0.80391731	0.002844829
HS6ST1	0.83505602	0.002900237
STARD3NL	1.213762599	0.002922012
MGAT4B	0.853905901	0.002939557
KCNJ15	4.467879118	0.002960323
ATP6V0B	1.295051523	0.003007667
SLC25A36	1.357077026	0.003033102
ALDH5A1	1.261727101	0.003041887
SLC25A16	0.854790459	0.003064127
CYB5R4	0.840137525	0.00308708
SLC37A1	0.823615896	0.003096194
DHRS7B	0.843915192	0.003117421
RHBG	13.0422027	0.003133475
PLCXD2	0.750629938	0.003147095
TAP2	1.352493074	0.003351911
CHRNA10	1.390106901	0.003377863
KCNMB3	1.567866898	0.003389699
SLC4A5	1.409669512	0.003407781
KCNK6	0.825701509	0.003426767
SLC25A28	1.167787368	0.00358241
MT-ND5	0.58541743	0.00365733
AMDHD2	1.364669526	0.003783626
ATP11B	0.845768708	0.003790144
PLCH1	1.269028063	0.003809965
KCTD19	3.40859628	0.003866276

CLCN3	0.852893883	0.003872215
TRPM5	0.62530426	0.003906713
PDHB	0.895596978	0.003914454
ENPP7	0.191834548	0.004001313
AHCYL1	0.900197675	0.004002076
SLC16A3	1.301236532	0.004255863
SLC28A1	0.11797381	0.004315921
HNMT	0.818457157	0.004326902
STS	0.69546907	0.004333926
FDFT1	1.289685465	0.00434365
NDUFS3	0.872047601	0.004390423
SLC30A2	3.960339488	0.004400767
SLC33A1	1.15941405	0.004430184
KCNAB3	1.541949122	0.00453424
PIK3R3	1.265577608	0.004572975
GGT2	0.425830391	0.004575894
SLC25A37	1.288464033	0.00458702
OCA2	3.616551972	0.004607016
ALG9	1.169250306	0.004640987
MT-ND6	0.486982298	0.004733934
CHST4	37.92650402	0.00473773
IMPAD1	1.222461143	0.004928971
KCTD17	1.245620183	0.004944057
SLC34A1	2.8994974	0.005096151
NPC2	0.866622429	0.005107635
COQ10A	1.212061919	0.005144985
AGPAT5	1.266453168	0.005294237
ARSI	1.879035582	0.005480613
SLN	3.403189788	0.005622098
DECR2	1.265253221	0.005646385
ABCA12	12.43681138	0.005670108
CHST2	0.775106029	0.005723329
CLCN1	3.697569784	0.005791106
KCNK12	0.640499446	0.005878672
FAR1	0.843562549	0.006009988
AKR7A2	0.861193948	0.006031976
KMO	0.672295208	0.006174145
ABHD13	1.225636098	0.006180199
ALAS2	0.429296703	0.00629577
CNP	1.129078481	0.006333504
PIGO	1.155423877	0.006361024
TRPM7	0.841327067	0.00662742
PLA2G2A	0.541718069	0.006649035
SDR16C5	2.05237676	0.006743186

FAHD2B	1.318174579	0.006801416
NT5DC1	1.1643189	0.006855553
PGM2L1	1.394331828	0.006940338
FAHD2A	1.141700855	0.007013587
SLC9A8	1.205136212	0.007016739
MAN2C1	1.211523165	0.007041148
ME3	1.276484175	0.00708368
NAGS	0.721801216	0.007087158
GPX7	1.433513071	0.007185023
NDUFB2	0.878326693	0.007429153
MAGT1	1.204383652	0.0074732
FAAH	1.231869579	0.00754393
GUSB	1.176939797	0.007778519
ATP6V1E1	0.901746657	0.007813478
PDE6D	1.138071002	0.007832227
GANC	0.872945174	0.007854129
SLC16A10	1.526773489	0.007931659
MLYCD	0.885981456	0.008002237
PIGP	1.19439112	0.008024205
OTC	0.481436627	0.008114154
GUCY2C	0.824815837	0.008140657
OXSM	1.163044297	0.008141357
ABCG8	0.320626489	0.008170863
PDE8B	0.779614734	0.008389984
SLC16A8	2.601271256	0.008463041
DUOX1	1.868007124	0.008474353
NDUFA2	0.842660244	0.008497789
B3GNT2	0.867489262	0.008639822
AK7	0.762680099	0.008688314
SLC35C1	0.887095586	0.008721402
SLC5A10	0.649419602	0.008723295
KLK8	638.3292012	0.008728962
SLC24A2	3.144133132	0.00881166
LSS	1.251253234	0.008847323
SLC45A4	1.182125745	0.008851236
BPHL	1.153676397	0.008937874
AKR1C1	0.544977104	0.008986194
PCTP	0.871019006	0.009041373
ABHD2	1.90452214	0.009192317
SLC35F5	0.84197959	0.00933808
HSD3B1	0.468877023	0.009340893
ABHD14A	1.2344049	0.009437141
NDUFA7	1.223046184	0.009522899
CRYL1	0.790645867	0.009893658

ALG6	1.167853527	0.009908418
SOD3	2.175203379	0.010117265
LYZ	2.757596148	0.010172811
SLC5A4	0.444109314	0.010209053
UGT1A9	0.305128042	0.010249628
FMO1	0.361103223	0.010326845
UGT3A2	0.459368813	0.010339285
RYR1	0.530425901	0.010539047
DAGLA	1.239153222	0.010561928
NDUFS6	1.332650739	0.010840426
HSD17B6	0.430979207	0.010865341
PIK3C2G	0.234622126	0.01090141
GRID2	0.349236707	0.010901643
ABCB9	1.240638834	0.010958264
PIGB	0.874261132	0.011301286
PAH	57.95184733	0.011457047
NPR3	0.515235211	0.011494171
KCNC3	1.607363879	0.011511874
KCNF1	0.578325624	0.011595159
UGT1A6	3.766149058	0.011679766
DHRS12	1.230452465	0.011787615
ITPK1	0.876817825	0.011801043
SLC22A2	2.457242932	0.011813985
ABCG5	0.34106442	0.012086605
SLC2A3	1.646784755	0.012141915
ACSL3	1.174947922	0.012201378
NAT8	0.316341319	0.01246567
PDHA1	1.175286923	0.012478015
KCNS1	2.725437462	0.012591823
SLC1A2	0.505554627	0.012630849
CBS	2.7737304	0.01266321
SULT1A4	0.513240419	0.012759478
MTRR	1.141479589	0.012787361
SLC16A14	0.765864546	0.012795352
PLD2	1.157663685	0.012818527
MOGAT1	3.641854088	0.012893254
ACADL	0.371690029	0.012906659
KCNH2	0.726081563	0.012947405
SLCO1C1	0.422540728	0.013071514
LDHAL6B	0.581919219	0.013160326
ATP6V1B1	4.584681277	0.013318952
ALG11	1.223245972	0.013319187
SLC22A18	0.814403249	0.013485074
FUT7	2.25001749	0.013584956

PANK4	0.904051926	0.013701076
ENPP3	0.664554809	0.013726444
GSTA4	0.837685489	0.014320352
SLC7A4	1.546220471	0.014382716
CHST7	0.808167962	0.014505412
DDHD1	1.289812535	0.014519624
ST6GAL1	1.31678176	0.014546533
FN3KRP	1.123084427	0.014616834
PFKP	1.227184629	0.015020263
SLC6A1	2.685370612	0.015071382
PLCB1	1.456168291	0.015274112
KYNU	1.937391456	0.015419594
KCNK15	3.13487152	0.015650674
IVD	0.884141056	0.015870311
PIGF	1.140171264	0.016157553
OXCT1	0.782805049	0.016324361
GLYCTK	1.293258237	0.016377974
AQP9	5.098347268	0.016423319
SLC6A12	0.666921773	0.016532691
SDR39U1	1.146914019	0.016570578
ITPKC	1.174508637	0.016878339
G6PC	0.109407009	0.016911205
PGK2	6.580669368	0.01712675
PON2	1.199160939	0.017255833
KCNT1	4.466386309	0.017408781
NNMT	1.904181058	0.017413106
SULT4A1	0.511450826	0.017495461
PLB1	0.610170771	0.017668993
EPHX3	4.267837714	0.018128922
GALE	1.161956284	0.018572417
DLAT	0.890796152	0.018643789
ATP11C	1.29311023	0.018753504
CYP51A1	1.239817761	0.018902118
ACSBG2	1.890773439	0.018934485
PKD2L1	0.662425411	0.019005959
PDE10A	2.377805862	0.019034864
AQP2	11.16780329	0.019068155
LPO	32.0694355	0.019100304
SLC4A9	0.598016423	0.01912504
SLC5A1	1.338620375	0.019241544
ACER2	0.788649983	0.019522411
THNSL2	1.343398528	0.01980585
SLC43A3	1.584016897	0.019987157
CYP26A1	6.876668122	0.020035726

PMVK	0.872933793	0.020119122
FA2H	0.851245147	0.020237075
ALDH16A1	1.21956321	0.020252714
CSGALNACT1	1.491182372	0.020253257
PANK1	0.851141812	0.020298723
CYP4Z1	6.183818395	0.020341008
CBR3	1.433384951	0.020735999
PLA2G6	1.213617035	0.021364316
CYC1	1.276792613	0.021862412
IDH3B	1.176469883	0.022374593
ABCA13	2.117227089	0.022716965
MTHFD2L	1.263569667	0.02339861
CACNB3	1.142121732	0.023414723
TCN1	168.0405296	0.023709689
ALDOA	1.16426297	0.023976246
SLC26A10	0.697945537	0.024369232
CHST9	0.264710683	0.024592397
ISCA2	0.884376485	0.024627355
LTC4S	0.59173676	0.024915069
CMPK1	0.878359151	0.025030698
NDUFB5	0.864907815	0.02623973
DGKH	1.796607867	0.026335253
GK	0.802232702	0.026861646
ATP2B3	0.225525146	0.027130363
SLC16A7	0.556123502	0.027241348
FUT2	0.861914622	0.027255921
ABCG4	0.700622877	0.027564042
SGSH	1.14112547	0.027646869
CSGALNACT2	1.256002869	0.028171022
GYG1	0.874981994	0.028395571
SLC6A17	0.564930286	0.028407665
ALDH1A3	1.434017052	0.028527832
PCYT1B	0.571895828	0.028904875
GABRB3	0.570206909	0.029146254
ELOVL3	2.116319499	0.029688523
CLCC1	1.10961579	0.029792674
GUCY2D	2.641168431	0.029878059
DUOX2	1.916143913	0.029954815
NUDT18	0.865918211	0.030710164
GNMT	2.717788116	0.030889599
CHPT1	0.885857701	0.031388227
MPO	1.935601634	0.031518957
DEGS2	1.389995383	0.031728397
BCO2	0.745906295	0.031911452

AADAC	0.2408669	0.032032648
CS	0.919959534	0.032285054
NPC1	1.139648403	0.032765663
CATSPERB	1.444107313	0.0332741
ACY1	1.177930999	0.033450892
ASL	0.867530055	0.033480425
DEGS1	1.166250646	0.033574246
DAD1	1.123833516	0.034049377
SLC1A3	1.710730597	0.034161607
OLAH	3.364611493	0.034162938
HCN1	5.557424309	0.034278035
SLC26A6	1.17163068	0.034405091
PNLIPRP1	0.696665745	0.034453636
ETFB	0.859295355	0.034475472
SULT2A1	0.259070227	0.034593435
ISYNA1	2.089185293	0.034630348
KCNV2	4.157337668	0.034671988
CYP26C1	2.777465957	0.034743257
ST3GAL5	0.800161427	0.034921685
SLC7A2	0.604448226	0.034948085
COX4I1	0.885573488	0.035521792
FOLR1	23.97825343	0.035765988
SCN8A	1.411489688	0.03697123
LYZL2	#DIV/0!	0.037531281
PIPOX	2.755137761	0.03767993
MOCS2	0.896793694	0.038244231
SPTLC2	0.890706447	0.038259738
SPR	1.108920233	0.038922069
PLCH2	2.087804006	0.039275645
AASDH	1.127794286	0.03971576
KCNA10	8.719449965	0.0397712
SLC35E1	1.075042965	0.039907533
B4GALT6	1.37362788	0.0403482
MSRB2	1.14775095	0.040609027
MT-ND1	0.81216254	0.040723903
ATP6V0E1	1.100087597	0.040796968
UQCR11	0.890621661	0.042015397
MINPP1	0.9006978	0.042040028
GALNT3	1.158398507	0.042321408
PPIP5K1	0.908243117	0.042626433
SLC13A4	1.460890381	0.042820848
LCAT	1.358676686	0.042825367
AKR1C4	75.2511144	0.043211879
AOAH	1.301580953	0.04355234

DGKQ	0.875983036	0.043565265
A4GALT	0.783027837	0.044029876
LCTL	1.706823742	0.044075521
SLC25A3	0.923287357	0.044425279
ARSJ	1.402751019	0.04447401
SLC6A18	0.618056275	0.044914298
PIK3R4	1.093783145	0.045220741
UCP1	8.848345551	0.045414313
PIGH	1.108760484	0.045560171
PRPS1L1	20.17057854	0.046392128
CYP4F2	0.56560054	0.047180613
GLT8D2	0.797984987	0.04719401
OGFOD2	1.117411327	0.047452005
VDAC3	1.167251631	0.048071356
SLC22A13	2.084411381	0.048144096
PLCB3	1.174469878	0.048442021
ILVBL	1.146483346	0.048945762
ALOX15	1.678992514	0.049075966
HK3	1.510894587	0.049106275
SLC2A9	1.187273424	0.049135791
SLC10A5	0.738856138	0.049373791
CBR4	0.881239203	0.049795134
PLA2G2E	8.597853007	0.049987488
ST6GALNAC5	1.556784275	0.050140313
KCNA4	0.244692007	0.050594183
CYP24A1	4.753682994	0.051096186
MCOLN3	1.75978126	0.051528854
CYP19A1	3.202820465	0.051744909
CA5B	1.240888383	0.051755421
TYMP	1.554591859	0.052122844
MIOX	11.6476064	0.052677726
FPGT	0.88101975	0.053016301
GABRR1	4.736509227	0.054978797
ENO2	1.313953004	0.054999729
ABHD12B	2.187567701	0.05528122
PDXP	0.811554214	0.057329682
GGT7	1.231702105	0.057530045
ASMTL	1.137784448	0.057540423
SLC39A2	2.584645952	0.057863021
DPYSL2	1.2820441	0.058445789
PGAP1	1.284847509	0.058483025
P2RX6	0.656762471	0.059280914
KCNMB4	1.465286842	0.059646788
UCK1	0.91864553	0.059860564

FUT6	0.868373281	0.060162917
KCTD16	4.353085437	0.06263903
AMD1	1.123793952	0.062834101
GLB1L3	0.453900884	0.063050264
SLC25A2	3.060112406	0.063296874
NDUFAB1	1.12218926	0.063753472
GK5	0.877563292	0.06401916
SLC22A14	1.894671302	0.064189604
MT-ND4L	1.238226202	0.06479236
NDUFA3	1.209878451	0.064884834
NT5C1B	0.747711904	0.065200861
ALOX5	1.394673709	0.065687406
ATP6V0E2	1.185829595	0.067714627
GGT1	0.787212108	0.067899601
SLC16A6	1.403811851	0.069011029
GAL3ST1	0.805662872	0.07037121
GABRA3	5.240039841	0.070428471
KCNJ8	0.807438892	0.07073416
CA5A	5.157979602	0.070859112
SLC25A30	0.878624121	0.07396955
GABRB1	4.087754059	0.074016303
SLC26A11	1.128776366	0.074665813
GGT5	1.298218467	0.074847104
ACO1	0.921851519	0.075280462
ATP6V0A4	21.69814497	0.075688151
PDSS2	0.92328993	0.076394786
ETNK2	1.371742872	0.076576774
SLC12A4	1.149686666	0.077486642
BCAT2	0.900962758	0.077792933
GABRA1	0.325364797	0.079089015
SLC7A3	0.557383282	0.079274824
ACSL1	1.249398226	0.079336788
COX4I2	1.242863657	0.079619305
PLA2G4C	0.816826874	0.07992928
CA8	1.77602116	0.079992477
GADL1	3.025145778	0.080807874
SLC16A11	1.401177605	0.080812241
DGKD	0.907990203	0.080897516
CHRNB3	6.056757337	0.081434647
TDO2	4.559394839	0.081474522
SULT1C3	3.903090822	0.081805725
COQ5	0.923152328	0.082351109
BEST3	23.16407713	0.082494257
B3GALT2	0.669206397	0.08341538

SLC29A4	0.710224545	0.083975567
PIK3C2A	1.1574573	0.084692102
KCNE4	1.335229086	0.084864271
SLCO1A2	17.70382415	0.085049464
ATP10D	0.870353407	0.086345536
SLC25A38	1.071688158	0.086648658
SLC25A33	1.111320603	0.087241138
ATP8B3	1.830454897	0.089176188
MT-CO3	0.875571255	0.089779959
SLC2A6	1.249369562	0.090300893
CHST12	1.120269882	0.092453611
PYGB	1.255068945	0.093381992
PNPLA4	1.186876438	0.094440729
NDUFA11	1.166799155	0.095183215
ALG13	1.153022766	0.095260289
SLC9A6	0.921570817	0.095904297
DPYSL5	0.282783907	0.096391863
KCNK7	1.443840161	0.096450646
KCTD21	0.928387551	0.098322171
GMPR2	0.936273683	0.099881224
GMPPB	0.908374963	0.100333169
NADSYN1	1.094897736	0.101101863
LIPE	1.37109572	0.101331747
CEPT1	1.104192075	0.102535807
LIPJ	0.566346529	0.102540778
GRIK4	0.732560723	0.102602831
ACBD3	1.091529537	0.102642751
SLC26A8	1.403718585	0.103128784
UGT1A3	0.40508172	0.103577978
CDO1	0.39557079	0.105008859
ACSM5	0.29143793	0.10522672
ASMT	1.6142291	0.105832349
COQ3	1.115388502	0.106175456
AMY2B	1.327479698	0.106574565
CLCNKA	0.6449946	0.107457498
CACNA1F	1.44662725	0.107828062
ATP6V0A2	1.08721615	0.10822184
SLC38A6	1.148726381	0.108412153
GALNT9	2.893568689	0.109102453
CP	0.350769382	0.109694206
ABCC11	1.362223187	0.109980951
DDO	0.800515021	0.110830138
KCNK17	2.127634998	0.110936534
PLA2G2F	7.600163152	0.111113466

STARD4	1.174665827	0.111865898
PRPSAP2	0.923884984	0.113308452
HK1	0.926737747	0.114221933
UGT1A7	0.547382008	0.115439214
CRYM	0.824866437	0.1155314
GDA	0.839869895	0.115987705
GCH1	0.910402469	0.116360605
IDH1	0.922525843	0.117771183
LIPT1	1.090384196	0.119833773
AOX1	0.404069139	0.122684876
HS3ST3B1	0.838859859	0.123062599
CLCN6	0.914466031	0.123133611
SOD2	1.158618947	0.123503942
TRPC3	0.820885133	0.123660353
G6PC2	0.522951287	0.123674145
ALG14	0.924201642	0.123678071
LYPLA2	1.080587662	0.123795473
ACY3	1.347657711	0.126346715
IP6K2	1.072187869	0.128121851
NME5	0.735192309	0.128474421
NUDT17	1.132169498	0.13011491
CYP4F11	1.194774534	0.130127706
SLC6A15	0.451397393	0.130206543
SVOP	0.407922806	0.130574691
KCNH5	0.458767702	0.131887054
GDPD4	1.547926696	0.1325061
SLC38A1	1.101595572	0.133277654
SLC43A2	1.109831666	0.133522602
TMLHE	1.099327751	0.133705028
SLC23A2	0.893245108	0.134118676
ACSL5	0.916290813	0.135542252
SLC39A8	0.869704302	0.135562789
STARD3	1.52201546	0.136507339
AQP5	54.52629346	0.137376503
CACNG1	6.897111134	0.137591329
DPM3	1.232943108	0.138606645
GCNT1	1.15054471	0.139534162
IYD	1.173842287	0.140092388
MT-CO2	1.153421476	0.1407645
PTGR2	0.905360588	0.141659004
CYP3A5	0.840426762	0.142074642
NT5C	1.090592005	0.144753313
CNGB3	2.278984752	0.145328789
SLC6A3	6.080563742	0.145520288

SLC14A1	3.409427911	0.146113557
PIGV	0.931443061	0.147330427
SLC25A18	0.733670691	0.147644881
PIK3CA	0.900769481	0.150437611
SLC30A9	0.933574209	0.150874696
SLC26A4	0.624740943	0.151119304
DDAH1	1.086735125	0.151502396
STARD10	0.897767121	0.152742272
GNS	1.086082866	0.153111071
ATP2B2	0.393915076	0.153514396
NDUFA12	1.078565313	0.153542483
ABCC5	1.108942539	0.153821245
ADCY1	1.457456321	0.154242084
SLC36A4	1.194194372	0.1547358
FOLH1	1.883163739	0.155201874
SLC38A3	9.759813939	0.155439712
P2RX3	1.966307529	0.155799177
KCNV1	0.719153588	0.157861882
NDUFA8	1.073630222	0.159298149
ATP1A4	0.642250256	0.161033316
GABRP	11.44375747	0.161112111
ALG10B	1.184336026	0.161849848
ALDH7A1	1.087782122	0.163058767
BPGM	0.927768331	0.163851559
CA13	1.159571437	0.164058107
SLC22A12	17.68381719	0.16670405
KCND1	0.857597613	0.166880576
ALDH1L1	0.74401996	0.167432003
HSD17B3	1.537615325	0.168125017
MMEL1	1.32527469	0.168504444
AQP3	1.769968793	0.168947159
GALNT4	1.181418014	0.1721635
SLC35E3	1.065171468	0.172454196
ATP6V1A	0.934360363	0.173110781
TRPV6	3.565792377	0.173331202
SOD1	1.087040624	0.174557533
LIAS	0.92760001	0.174948407
MGAT3	0.829543891	0.177180494
INPP5B	0.938771699	0.178031356
ADSSL1	0.786793362	0.178299515
CNDP1	3.731184541	0.178680826
TYR	18.32107912	0.178863022
ADCY4	1.144935301	0.179276321
NDUFV1	0.921094652	0.180019052

IP6K1	0.953339015	0.180904593
COX7A2L	1.069402615	0.183208189
EXTL2	1.09856112	0.183742209
CTH	0.878125046	0.184121476
PCCA	1.316837008	0.184279391
KCNJ3	1.484477819	0.184835884
PI4KB	0.954738113	0.185063489
SLC45A3	0.888349674	0.185333264
GALC	0.869606647	0.185495862
NDUFB10	0.920954102	0.188715679
ENTPD1	1.124083441	0.189814538
PTGS2	1.505566713	0.19096872
SLC8A3	0.387668521	0.191005215
UPP2	1.687880987	0.191482606
PLA2G4D	3.156144774	0.194935663
ALG12	0.901118573	0.195024182
ATP2C2	0.910506131	0.1955023
KCNG2	1.33271708	0.200051993
SGPL1	0.948455029	0.203441491
AK2	1.061787094	0.205905056
SLC35F2	1.082856087	0.206161389
KCNJ4	1.681620052	0.206897101
ABAT	0.850709838	0.207704516
NCEH1	1.087543395	0.208421418
UCP3	0.895707675	0.20990816
KCNJ10	0.786301634	0.210789455
ACSF3	1.073216705	0.211933872
LIPK	#DIV/0!	0.213602096
MT-ATP6	1.123600929	0.216648555
CA6	14.37699807	0.216878161
NDUFV3	1.054660706	0.217794945
SLC2A10	0.872419326	0.219861485
BLVRA	0.888693314	0.221378066
DHRS3	0.926012475	0.223218667
GRIN2C	1.818379616	0.223518789
PIGK	0.934883696	0.223599241
SLC25A43	1.072768331	0.22376428
GABRA4	3.658779449	0.224316533
CHST10	0.845298226	0.225329895
MMADHC	1.060809078	0.22721564
SLC4A7	1.161492118	0.228653465
CYP4F22	0.726525669	0.229140931
SOAT2	0.541207743	0.229498798
SLC25A45	0.917912694	0.229959313

B3GALNT1	1.182183485	0.230465389
NDUFS7	0.906711449	0.232600313
PDHA2	3.444849974	0.233482983
ITPR2	1.182352133	0.234339446
TTYH1	0.697928791	0.234356414
GTDC1	0.941015285	0.23651436
GXYLT2	0.838730015	0.237457967
GBGT1	1.148070041	0.239095208
AMY1A	0.538646199	0.241863545
BSND	1.678642296	0.242308695
ALDH1L2	1.235901522	0.24385951
MT-ND3	0.890871894	0.244813373
OAS1	0.909706286	0.245992261
KCNH3	2.206496439	0.24697685
AWAT2	3.832163207	0.247884044
TRPM1	4.670672925	0.248499092
ATP10A	1.166033835	0.249139774
CHRNB1	1.131568718	0.249167423
CHRNE	1.275999774	0.252419984
ATP1A3	0.687140732	0.254194181
PGAM1	0.940448775	0.255747105
SLC2A14	1.474625441	0.256365706
GCNT7	2.359745672	0.258903389
HAS2	1.187941709	0.258945339
RHAG	0.509069571	0.260347824
MIP	1.171750006	0.261700932
HCN2	1.614017145	0.261795452
SLC30A3	1.868287014	0.263659984
GALNTL6	1.912785309	0.264224221
HS3ST4	27.2597537	0.264341012
PKD1	0.921308427	0.265291753
PRDX3	1.076749404	0.266286327
SLC16A5	0.907966276	0.267464036
TK2	1.061164825	0.267983423
SLC2A2	0.21762487	0.268272467
SLC35F3	0.634966197	0.269704799
SLC36A2	3.521894395	0.269850736
PON3	1.482315744	0.270114279
GALNTL5	#DIV/0!	0.271452845
KCNA7	3.321483807	0.271628872
GATM	0.750727671	0.272191029
STAR	0.619480278	0.274699535
GRXCR2	0.665087633	0.274968076
H6PD	0.942310405	0.275083526

COX7B	0.910252569	0.275253696
CHRNG	4.180978915	0.275305623
DMGDH	0.587951991	0.277418189
ARSF	0.682573139	0.277963457
HSD17B8	0.915855561	0.279256249
PHOSPHO2	1.099857509	0.279442978
SLCO6A1	7.025490513	0.279605996
MGAT5B	0.636484563	0.279882886
DCT	3.406933566	0.280775192
P2RX5	0.839670773	0.281445975
HYAL1	1.336880561	0.281554068
RDH8	3.636884412	0.286259912
MTTP	0.606634593	0.28706014
GLRX	0.906481238	0.287667929
SLC37A4	1.060750263	0.289928754
ENTPD7	1.083381469	0.291334778
RDH16	6.903186934	0.295175063
COX6C	0.936778228	0.295509399
NT5C1A	0.642510032	0.297482972
HCN4	0.467444537	0.298525745
SLC4A3	1.338331766	0.298858944
DGKG	1.146144828	0.299455742
INMT	0.889203608	0.300144384
KCTD11	0.941538469	0.30091652
MT-ND2	1.151898903	0.301185338
BAAT	37.99038352	0.302726765
SLC6A13	1.840934251	0.303370016
BCKDHB	0.940789054	0.304054774
ST6GAL2	0.754382461	0.304398015
UROD	0.943106849	0.304429482
KCTD3	1.060290366	0.304995122
GLUL	0.927226152	0.305046012
GLRA3	0.616818033	0.305067052
A4GNT	2.308367117	0.305954983
SLC26A5	1.572072024	0.306461372
PNPLA2	0.930401057	0.307008042
IDI2	1.770768899	0.307039463
CYP4F8	10.40086413	0.307445133
AGA	0.942154377	0.308748909
PDE6C	1.219962039	0.310239877
PKLR	1.388833548	0.312038442
ELOVL7	1.108603719	0.313486443
NAA30	1.066480496	0.314108249
CHRNA6	6.218952039	0.316200259

HAL	1.706566748	0.316730809
GALNT13	0.632428592	0.317060485
AMDHD1	1.787869887	0.317440992
SLC2A7	0.577432733	0.318316386
SLC6A4	0.755173557	0.318406069
SLC25A31	2.29379229	0.318548492
UQCRB	0.943238845	0.320630234
CFTR	1.105884582	0.321375848
COX16	0.948522528	0.322475505
PGAM4	1.186295876	0.326909942
GUCY1A2	1.366855187	0.328517979
SLC32A1	0.49675491	0.329178529
SLC25A5	0.931791864	0.332459862
GDPD1	1.08755393	0.334288003
GSTA3	0.552221555	0.33567642
SLC4A1	0.5431492	0.337370336
CYP3A43	2.200613151	0.337383855
PPCDC	0.950681049	0.337723331
SGMS1	0.936788361	0.337737457
SULT1E1	3.895597947	0.337757908
ACOX2	1.113247069	0.338765368
GAA	0.918973394	0.338783834
SLC12A5	1.258313718	0.338966732
KCNJ12	0.771313422	0.339031795
GPX5	14.62751766	0.342301057
ABCA4	1.831518845	0.344528712
AGPAT4	0.891642754	0.34527835
CPT1C	1.227697548	0.345679223
SLC39A7	0.953188158	0.345878444
TRPV5	2.640606586	0.345936847
UROC1	11.33541577	0.346839828
CA3	1.176037236	0.347419832
NDUFA13	1.115053373	0.347748111
KHK	1.145963351	0.348523682
CNGA4	1.215239579	0.350756542
UGT2A1	6.370363436	0.350979798
GLRA1	2.805731394	0.351186258
KCNK1	0.916912747	0.352385786
GABRR2	1.158420531	0.353918383
ALDH18A1	0.960316348	0.355275871
MAT1A	13.68619801	0.355798214
ALDH1A2	1.817632748	0.356771227
TRPM8	3.896286462	0.360258537
FMO3	2.875899047	0.360960893

CYP1B1	0.76368077	0.364838766
CACNA1E	3.079013739	0.365014165
GABRB2	0.787301701	0.366887447
CHST13	1.289724032	0.36794203
INPP5F	0.934843679	0.370439512
PLD3	0.949125663	0.371285618
ALOX12	0.893047428	0.371862913
SLC16A2	0.817752965	0.372412251
PNLIPRP3	0.497514353	0.374536954
LNPEP	0.916634314	0.378809574
COQ2	1.050923379	0.3811392
LIPN	1.56942021	0.38399479
DGKK	2.2204257	0.386072495
ST8SIA2	0.834793579	0.386403234
NDST2	1.045122376	0.387833951
GAMT	0.766935586	0.388371992
DHRS2	31.71363413	0.389298309
AMACR	0.91457175	0.394716789
SULT6B1	4.392115151	0.396203361
DDC	0.929613384	0.400233885
ATP13A5	0.54650629	0.400349576
DPYSL4	1.270844077	0.401614049
ABCB10	1.046937805	0.403143563
CH25H	1.112011648	0.404179661
PPA2	0.95219921	0.404645031
EPX	1.257899111	0.410155774
SLC25A13	1.048300617	0.412339323
MFSD8	0.953161013	0.412728973
GABRQ	1.562687255	0.41326931
RPE65	2.622693402	0.414891185
SUCLA2	1.063126014	0.415811178
ACMSD	3.671970055	0.416620141
FBP1	0.914272739	0.418873931
THEM5	1.12893567	0.419059994
TXNRD1	1.041612149	0.421016406
ATP7A	1.07280698	0.421645261
DGKI	1.487869824	0.424934148
CES1	0.779509328	0.426161716
CHKA	0.948619301	0.426384051
ASS1	0.908860878	0.426488714
SLC26A9	137.1158407	0.427201846
GALNT14	1.624435107	0.428437314
HSD17B1	0.923339611	0.428846631
ACOT6	1.458552236	0.429407816

ZACN	1.111124766	0.431912023
UQCRQ	1.072419108	0.432140616
BBOX1	3.995797719	0.434307071
CHRNA9	1.875139399	0.43841741
COX7B2	136.6013063	0.441395022
SLC22A6	#DIV/0!	0.442555261
NDUFAF1	0.961659567	0.442585932
ACOT8	1.061107266	0.443917844
CHST8	0.420567577	0.445465158
NDUFB6	0.954070035	0.445762162
GALNS	1.046937999	0.448214922
SLC34A2	2.598846373	0.449757473
IDO1	1.734550524	0.45067391
MGST2	0.957219309	0.45127875
ATP12A	2.059197392	0.451335324
NMNAT2	0.810714098	0.453717561
CDS2	0.952501109	0.455578676
SCD5	0.908331072	0.456629318
AQP10	1.406795801	0.457957337
SLC5A9	0.827964037	0.458051417
NUDT16L1	1.059255311	0.458255023
PIKFYVE	1.066361138	0.459909077
SLC25A40	1.053259699	0.461164087
PLA2G4A	0.870999439	0.461740391
CHST6	0.789533626	0.463842363
HCCS	0.962702638	0.466287745
PDE4B	0.899236197	0.467885422
UGT8	1.056714361	0.468180425
IDI1	0.950317097	0.468537736
AADACL3	4.0124681	0.468861647
SLC22A25	9.143562804	0.470577546
GLT1D1	0.854114103	0.4710168
ADCY8	2.725500743	0.472934211
NDST3	1.189811261	0.474903283
BCAT1	1.196091302	0.475747918
NDUFB4	0.966760396	0.477653417
SLC39A14	0.954504866	0.478567999
B4GALT5	1.038586473	0.478666145
AGPS	0.963570371	0.479082987
RENBP	0.913802683	0.481801179
TRPV2	0.936121957	0.482522598
NALCN	1.224123514	0.482652251
SLC39A9	1.029826468	0.482958258
PRODH	1.255282755	0.4842596

SCN2A	1.837894037	0.484356639
SLC25A21	1.235357145	0.487268776
ACYP2	1.042628041	0.487661807
SDS	1.161300839	0.489812086
KCNH7	1.313059462	0.490542502
BEST1	0.933367582	0.491674095
TKTL1	12.27769796	0.492929997
DBH	2.25538339	0.493203485
GLDC	1.370143878	0.495515706
SULT1C4	2.213131528	0.497152805
COX6A2	0.615487487	0.497484059
ELOVL1	0.969435278	0.498593491
FBP2	1.135716535	0.500309573
CHST11	1.103104466	0.500399542
KCND2	0.824554943	0.500840106
DHDH	0.836169915	0.50360658
NT5M	0.93057364	0.503608851
DDHD2	0.954917526	0.503953129
HSD17B4	1.037762958	0.50582733
KCNU1	6.706396266	0.506937808
SLC6A11	0.731369708	0.508102692
HAS1	0.790174674	0.5084568
ABCC12	9.821986982	0.508595096
ASAH2	0.927362924	0.508733823
GGTLC2	0.820599279	0.510706047
MANEA	1.058768859	0.5141807
MME	1.28617934	0.516216956
SLC7A7	0.921124619	0.516473029
CYP11B2	15.02969402	0.516598052
NDST4	0.662213711	0.517646453
LIPC	0.534154178	0.518214979
PLA2G12B	0.886271645	0.52049405
ARSK	1.04159226	0.520810434
HS3ST5	0.770423772	0.520920198
NUDT13	1.06665383	0.522546277
PDHX	1.028641286	0.524614401
MBOAT7	0.968570173	0.524672208
HYAL4	0.739067031	0.524738235
ARSG	0.952237222	0.52596926
ATP6V1B2	0.961781964	0.527937555
CYP17A1	1.96047343	0.528877648
UCP2	1.063801393	0.531184517
GABRG3	2.717983747	0.531587515
KCNK16	2.758264254	0.533201955

HEXA	0.968792603	0.536349604
DIO3	3.007749285	0.539521195
COX6B1	0.964386091	0.539605914
CYP2A13	2.087704861	0.539697632
SLC12A3	0.80740231	0.539866332
PNPLA6	1.048688135	0.540901596
CACNA1S	2.341041849	0.541492438
NAALADL2	1.089200195	0.541634145
CHRND	40.60429414	0.542620686
PIGG	1.025036547	0.543801341
CYB5R1	0.972480976	0.544171163
GPX6	8.206345272	0.544412302
ATP4A	2.035063867	0.545141003
AMY1B	1.445999099	0.546823301
SLC25A1	1.045038278	0.547284988
SLC12A1	1.720551784	0.549668934
GBE1	1.035930886	0.549710408
LYZL6	2.81379384	0.555602629
CYP3A7	1.253735898	0.55751406
HSD11B1	1.254821032	0.558008668
ADI1	1.028411257	0.559593242
POMT1	0.970954708	0.561256966
SLCO1B1	112.7503908	0.564724917
TRPM3	1.464704689	0.56682652
TRPC5	4.364710936	0.568195165
UGT2B4	43.68704519	0.568699552
SLC22A24	4.59696527	0.570395446
KCNK4	2.130190549	0.571603349
ATP2A2	1.022782054	0.57327116
HSDL2	0.969447093	0.575565143
SLC25A41	0.905854466	0.576437137
NEU2	0.626188882	0.579104009
NPL	1.047460571	0.581368917
GABRA2	0.875600778	0.582794412
ST6GALNAC2	0.912580306	0.583653877
MPST	1.041166502	0.584154178
ADH7	0.584301743	0.587983438
NUDT2	1.043896293	0.589785578
DSE	0.931482587	0.589792399
TKTL2	1.419931063	0.591425491
LIPF	13.42861311	0.596254219
LGSN	1.399310948	0.597537041
ALDH3A1	1.111835722	0.59783737
CHIA	2.205468074	0.598106285

PNMT	5.371503679	0.600417243
B3GNT3	0.969226964	0.601444836
ACBD5	1.032017268	0.601515508
PON1	4.32326743	0.601603698
ENOSF1	0.958374011	0.602735155
PYGL	0.939543658	0.602831662
PLA2G7	0.940899104	0.603428309
GYS1	1.022176941	0.604476828
HTR3D	4.248177386	0.604694895
CYP7A1	1.864857753	0.60521673
ABCD1	1.045832949	0.606162988
ACSS3	0.827846941	0.607278636
HMGCS1	1.052166829	0.610038213
INPP4B	1.065465148	0.611451884
MT-ND4	1.055179438	0.611462394
DDAH2	0.96197054	0.613279315
GGTLC1	1.171165294	0.617034526
ABCC4	1.049888008	0.617364735
AASS	0.93932063	0.61873318
AMY2A	3.815516217	0.619026615
DCK	1.03365549	0.619910297
GLYATL2	2.929326588	0.623232119
KCNQ2	1.844506332	0.623934471
CDIPT	1.023189939	0.62541299
LYZL1	8.522013936	0.626190557
UPB1	2.216035218	0.626684548
OXA1L	0.980659021	0.632874998
SRD5A2	11.7680084	0.635054221
KCNE1	1.169636176	0.63524884
SPHK2	0.965456237	0.63547113
PDE11A	0.931866668	0.636840356
SLC36A3	2.031885012	0.637272152
KCNQ3	1.101357413	0.637284246
CPS1	1.747709773	0.63779055
ST8SIA5	0.803704696	0.637834166
CACNG7	0.542531792	0.637913959
SLC46A2	1.092934624	0.638448843
ADCY10	1.117973004	0.641510898
CNGA2	4.440877642	0.641558582
CYP21A2	0.858140829	0.642584363
COX11	1.026076758	0.643572275
ADCY7	0.951605515	0.645822689
KCNK18	7.01353105	0.646237889
SLC5A5	1.626026352	0.650107268

GALT	1.024884544	0.650510833
SLC17A6	7.373857705	0.650551586
AGXT2	0.474932483	0.652634343
AGPAT2	1.039603042	0.652897086
GOT1L1	2.359823931	0.653872835
NOS2	1.117022466	0.654768125
GUCY2F	2.46067188	0.655118368
LCT	1.181550563	0.655296597
PRODH2	15.08147365	0.65741845
KCNC2	5.36679111	0.657674953
SLC27A3	1.033063241	0.658210065
LDHAL6A	0.852985043	0.662593701
KCNJ9	0.732647018	0.663163298
ELOVL2	1.81679462	0.663567439
NT5E	0.944622066	0.66413365
PTER	1.028730012	0.66439351
SLC6A7	0.92432972	0.665669771
GNPDA2	0.970349631	0.667747707
NDUFA9	0.973241916	0.668673012
SLC13A5	10.53642525	0.669130529
SATL1	3.217983038	0.669453093
SLC44A3	0.971979766	0.674481243
CACNG3	3.864136441	0.676032995
GRIN1	1.113652394	0.677442973
DGKB	0.725981091	0.678305812
CACNB4	1.121290036	0.678985166
COX8C	3.811129969	0.679440475
DHCR24	0.97323113	0.679954997
HAS3	1.073358609	0.68222788
MTHFS	1.027134762	0.684687878
PNLIP	18.43821383	0.685888461
BLVRB	0.956420215	0.687934159
PNPLA5	4.102766925	0.688678492
ATP1B4	2.845120644	0.689474642
IDO2	0.863887867	0.690413808
TECRL	#DIV/0!	0.690754815
CCDC102B	0.945505663	0.692136133
GAPDHS	2.504263083	0.693422701
KCNG4	5.16737559	0.694057655
FUT5	2.032496666	0.695253581
SLC5A3	1.050793662	0.695909786
SLC22A8	4.209078585	0.696514322
AMY1C	#DIV/0!	0.696596059
HAAO	1.054146474	0.698240121

SLC22A1	3.185121997	0.698868253
PAM	1.029443301	0.699028511
GALNT8	0.891753017	0.702489292
TRPV1	1.0680137	0.703235527
GRXCR1	5.843484496	0.703648379
GALNT10	1.023375952	0.704988002
SLC1A6	2.163120145	0.705348624
SLC18A1	1.19197796	0.705784362
GABRA6	2.998404903	0.705861728
CACNA1B	0.834308853	0.706770901
KDSR	1.022194918	0.706979801
ACAD11	1.052621196	0.709473911
ALDH8A1	1.520650569	0.713446209
CACNA1G	0.874858979	0.719788008
STARD6	0.882873739	0.720253228
SHMT1	1.024051738	0.721767797
ABCB4	0.893015821	0.725570024
KCNN1	0.821932941	0.727112848
PIK3C2B	0.980927988	0.728076176
CHRNB2	1.456939853	0.729078091
UGT2B10	1.568505981	0.729952006
FTCD	3.423198365	0.730476758
ADH4	2.973992753	0.73270661
CYP20A1	1.015870483	0.735310279
B3GAT2	0.94550917	0.737223423
CHIT1	1.124572037	0.740182606
SLC6A5	0.666186122	0.740964076
BHMT	1.950860988	0.744468433
CYP2E1	7.21338176	0.747205754
GLT6D1	5.929848547	0.747556836
UPRT	1.01991139	0.749249413
CYP4A22	23.56637081	0.749334772
CYP11B1	3.506894666	0.754762327
SLC22A7	20.35457884	0.756558366
ALOX15B	1.24141452	0.759457102
CYP1A2	17.18502031	0.759682552
SLC5A8	1.795178449	0.759729898
SLC17A2	31.12881969	0.761621443
HAO1	48.65888994	0.762263089
DGAT1	0.976649119	0.76822724
CYP2C19	0.889949571	0.770608371
SLC9A4	1.720633674	0.772372968
CTNS	1.013646669	0.772840524
HGSNAT	1.019051063	0.77310684

ADAD1	2.047716577	0.774007743
OGDHL	0.928973979	0.77407009
SLC22A9	2.177507354	0.774077397
GALNT5	0.968717725	0.774145889
ADAD2	1.998141901	0.777811472
COX15	1.011446101	0.778879794
CHST3	1.034833179	0.780388672
GAD2	1.6893558	0.781470555
ALOX5AP	0.964631487	0.782253013
ECHDC3	0.938709097	0.783440721
CYP2A6	7.879096028	0.785699222
CYP4A11	9.222521655	0.785969993
CLCA2	0.706855437	0.786130142
HAO2	2.786897565	0.786225423
CHRNA4	2.085383989	0.787090584
SCN10A	1.974632205	0.788635866
DPYS	3.818064041	0.790265004
CYP2A7	1.491091448	0.790371545
TF	3.615351905	0.790895416
UGT2B28	1.579442738	0.791205174
MT-CYB	0.972053612	0.793991918
KCTD7	1.019591002	0.797679669
PFKFB1	1.04626593	0.799012816
KCNJ6	0.871752262	0.800123304
KCNN2	1.047755079	0.800961377
ACSM2B	4.468196529	0.801807564
ACSM2A	4.723030623	0.802337596
ARG1	2.211451974	0.80458179
GYS2	2.108263028	0.810521194
CYP8B1	4.211926283	0.811524714
NOX5	0.913056263	0.812801343
SLC6A2	0.805953071	0.814335548
AKR1D1	2.614676246	0.815713373
ALOX12B	0.954256709	0.816704694
SLC44A5	0.959387184	0.817784279
SLC15A3	1.026260072	0.818184604
GPD1	1.053259537	0.821914195
GFPT2	1.050025837	0.822592239
GK2	1.40548707	0.827322212
SLC24A5	0.878171626	0.830584051
GPAM	1.018111003	0.831884622
SLC10A7	1.01346636	0.831969851
DIO1	1.424923241	0.831987298
RRM2B	0.98265846	0.832859061

CYP1A1	1.185840196	0.833650338
HPD	2.084488793	0.836266111
SLC7A10	1.229129514	0.836570373
PLA2G1B	0.924524078	0.836678576
SPTLC1	1.009203882	0.836965308
GAL3ST3	0.771750381	0.837106516
PFKL	0.985182606	0.837934132
UGT3A1	1.676736301	0.838206808
SCN5A	1.073326455	0.840862432
SV2C	0.938048772	0.84323961
ARSD	1.016345546	0.843811361
B3GAT1	1.06813818	0.844147973
NDUFB9	0.987751834	0.851123184
KCNH6	0.914413909	0.851749028
AWAT1	1.179803592	0.855474657
CYP7B1	0.956431661	0.855548028
ACOT12	1.972099359	0.857360979
SLC34A3	0.926269159	0.857762559
COX5B	1.014488051	0.860089024
ADH1A	2.327577753	0.860367357
SLC17A3	0.871628307	0.861783958
SLC35F4	1.230471515	0.863540209
LPL	1.040885966	0.864393638
PLCZ1	1.423194567	0.86493618
ACE2	0.97081621	0.866417669
ABHD14B	0.99117122	0.871618552
PIK3CB	1.114952644	0.872772323
GLRA2	1.055785881	0.873513764
ATP1B1	0.985770362	0.873741254
PLCXD1	1.013484187	0.876614154
CHRNA2	1.271521456	0.877343994
COQ4	1.008818156	0.881361751
ACER1	1.054362349	0.884292351
MDH1B	0.975788068	0.884869781
SCN1A	1.138349493	0.888158186
ATP6V1G3	1.493665395	0.888812832
CYP2C8	1.55831463	0.894092677
ST6GALNAC4	1.012670024	0.894578738
RDH14	0.995164672	0.897629445
HAGH	1.00741899	0.897932176
MT-ATP8	1.012073868	0.898525717
TAT	0.719481493	0.900196439
FN3K	0.990185609	0.900481141
CACNA2D2	0.959863784	0.904023353

ABCA3	0.974326795	0.905070614
GABRA5	0.917368425	0.905160981
SLC45A2	1.024787127	0.905969563
DGAT2L6	0.924186007	0.906006764
UGT1A4	1.592719634	0.906654075
HSD17B13	1.209505594	0.910836541
PLCB2	0.988768173	0.910992246
SLC25A25	0.993197644	0.914059286
RYR2	1.024323582	0.916846043
CACNG2	1.136936061	0.92107706
LDHC	1.031094674	0.92121519
AGXT	1.137506966	0.926402958
NAGPA	0.994650315	0.927634717
UQCRH	1.005304154	0.929869193
SLC10A1	1.176739101	0.934145518
UGT2B7	0.95631728	0.936838528
TPCN2	1.003643198	0.938160867
GFPT1	1.005161805	0.939770553
HS3ST2	1.015705327	0.940481252
MAN1A2	1.005332152	0.942434152
MBOAT2	1.007413454	0.9451507
TPH2	0.916466252	0.945999696
PLA2G12A	1.003640366	0.94650294
FOLR3	1.025771511	0.947231711
MCCC1	1.003338663	0.948388305
ACAT2	1.004706179	0.948527843
PHOSPHO1	1.010722411	0.948844496
MFSD2A	0.993766281	0.949548428
ACOXL	0.987765155	0.951451096
DHTKD1	1.002924692	0.954869652
ASRGL1	0.992240018	0.956912339
SLC31A1	0.99740536	0.957954924
GSTA5	0.8953217	0.961893662
NAT8L	0.979110448	0.967170417
GLYAT	1.102929353	0.967494868
NAA35	1.001876406	0.96780686
GNPTG	0.998135476	0.968108525
KCNJ1	0.983435024	0.968417817
ARSB	1.003212503	0.970321771
CACNG6	1.070603346	0.970487297
SLC22A16	0.992432798	0.972272501
COX17	1.001874496	0.973035549
CACNA1I	0.987698223	0.974443268
ATP4B	1.022768623	0.974477903

UGT2A2	0.95006569	0.975228145
LALBA	0.975110064	0.975295064
NDUFC2	1.001811418	0.976715605
NDUFB7	0.997420176	0.977044713
PCCB	1.001745797	0.977243322
PRG2	0.98273761	0.978844526
NOX3	0.973971894	0.979775139
INPP1	1.001542782	0.981142379
SLC7A13	1.040878552	0.981552607
GABRR3	1.023855062	0.981828948
SLC10A6	1.002742609	0.982485905
SLC19A3	1.004572169	0.982526863
SARDH	1.003475955	0.987713534
PNPLA8	0.999244964	0.988828483
OXCT2	0.998726593	0.99119394
CYP2C9	1.020204822	0.991610565
CACNA2D4	1.001553204	0.992716755
MOGAT3	0.999117535	0.993727855
SDR9C7	0.991957881	0.994546831
GSTK1	0.999846225	0.997778617
AADACL4	1.005682224	0.998247964

Paired analysis of TCGA CRC RNA-SeqV2		
Metabolism-related genes	Tumor/Normal	P Value
GRIN2D	2.312867014	1.53458E-21
UGP2	0.841662528	5.92186E-21
ETFDH	0.820076755	6.41032E-21
CA7	0.319354043	9.87471E-21
BEST4	0.357867174	1.92666E-20
ADH1A	0.280004662	1.19659E-19
GCNT2	0.53871885	4.12174E-19
HADHB	0.917982325	3.174E-18
GLTP	0.865308432	3.23933E-18
SLC17A8	0.136121958	5.6122E-18
MTHFD1L	1.321990203	5.74162E-18
DAO	0.135259555	1.2509E-17
SLC39A10	1.218186993	3.96631E-17
SLCO4A1	1.489651007	4.34813E-17
ENPP6	0.39827655	4.77969E-17
SULT1A2	0.48575391	4.92824E-17
PRDX6	0.891935942	8.35898E-17
CA2	0.608312755	9.67611E-17
ACADS	0.802796308	1.56434E-16
PGM1	0.865910449	2.06063E-16
HPSE2	0.341378332	2.21051E-16
SULT2B1	1.738696971	2.32438E-16
ASPA	0.331811598	2.70376E-16
SLC6A6	1.359649464	2.72261E-16
SCN9A	0.552659618	3.21017E-16
AMPD2	1.124911638	3.61183E-16
SLC25A20	0.836267952	3.78939E-16
GRIK1	0.219336195	3.90026E-16
CHPF	1.168944366	4.31019E-16
P2RX2	0.116103245	6.36579E-16
HMGCLL1	0.357247521	7.32086E-16
PPAP2A	0.826849962	7.58615E-16
SLC22A18AS	0.702637407	9.19357E-16
GABRD	2.205958993	1.19052E-15
PPAT	1.210854398	1.35423E-15
ADHFE1	0.471517667	1.88654E-15
SQRDL	0.869787184	2.07784E-15
UGDH	0.838243585	2.48091E-15
AGPAT9	0.708163722	2.55342E-15
SMPDL3A	0.774008975	2.88185E-15

SLC7A5	1.339580141	3.08799E-15
ABCA8	0.442849307	3.14255E-15
RDH5	0.724394305	3.69251E-15
B3GALT1	0.372477953	3.89614E-15
ATP5A1	0.908506163	3.97684E-15
SLC27A6	0.323014359	4.08335E-15
SHMT2	1.128559267	4.43521E-15
AADACL2	0.017531348	6.29883E-15
SULT1A1	0.763801111	7.60796E-15
SLC25A34	0.494845693	8.47627E-15
HADHA	0.932325756	9.78596E-15
CHST9	0.143936057	1.16317E-14
MUT	0.931195049	1.42295E-14
LDHD	0.662197564	1.69506E-14
SDHA	0.924693835	1.78742E-14
ACAT1	0.87471587	1.9724E-14
SMOX	1.320369105	2.09373E-14
FUT1	1.518058437	2.23427E-14
B3GNTL1	1.245940504	2.28541E-14
CA10	0.277695144	2.90652E-14
GPD1L	0.882582637	2.93491E-14
GRIA3	0.419982578	3.01403E-14
GMPS	1.096150468	3.32459E-14
RETSAT	0.874439241	3.47155E-14
SUCLG2	0.898109614	3.50947E-14
CPT2	0.884722344	3.59348E-14
CA1	0.34101416	3.62001E-14
NAT10	1.111311415	4.27793E-14
SMPD1	0.842821074	4.44923E-14
CPOX	1.105200097	4.54564E-14
GYLTL1B	1.790658556	4.86014E-14
BCHE	0.456595993	5.46482E-14
ACO2	0.927780198	6.0794E-14
UQCRC1	0.935417165	6.70611E-14
SLC30A10	0.297329516	6.82866E-14
SLC4A4	0.512194886	8.29489E-14
OSTBETA	0.507509706	8.80254E-14
ATP6V0D1	0.915425937	8.89296E-14
SLC7A14	0.218681548	9.32872E-14
SLC25A23	0.875902852	1.07329E-13
GLS2	1.70397559	1.11907E-13
STRA6	3.416506847	1.14247E-13
FUCA1	0.859949534	1.14336E-13
MTAP	1.115144285	1.26082E-13

AQP8	0.344290489	1.38886E-13
SCP2	0.920250617	1.69494E-13
DHDDS	0.896188475	1.73607E-13
SLC3A2	1.104967741	1.7766E-13
DHRS11	0.759819082	1.82068E-13
SLC4A11	2.322020589	1.82093E-13
PYCR1	1.167768434	2.11349E-13
GRIA4	0.410304057	2.14983E-13
TTYH3	1.17200308	2.26778E-13
HPGD	0.719887512	2.43785E-13
HTR3E	0.131808242	2.45444E-13
SFXN3	1.126498324	2.47681E-13
SLC2A13	0.831199526	2.52767E-13
UGT1A8	0.460865353	2.71134E-13
CKB	0.819357194	2.78355E-13
EPHX2	0.851831324	2.79849E-13
GPT	0.567255082	2.85992E-13
CLCA4	0.412444593	2.93817E-13
SLC17A7	0.448311761	3.18807E-13
SLC6A15	0.21765697	3.38884E-13
ACADM	0.889896047	3.42178E-13
SLC16A12	0.378619071	3.8997E-13
TKT	1.077538855	4.00766E-13
ABCC13	0.439808216	4.59151E-13
GBA3	0.381410202	4.64457E-13
PDE5A	0.82251546	4.9296E-13
GPT2	1.191763021	5.02858E-13
PAICS	1.115446347	5.06866E-13
ABCG2	0.531485929	5.14663E-13
SCN7A	0.365946512	5.21139E-13
CNGB1	0.189153795	5.2226E-13
ABCD3	0.908467577	5.61924E-13
AHCYL2	0.802884875	5.68711E-13
ATP6V1G2	0.510199783	5.74636E-13
MOSC2	0.84547561	5.98165E-13
MGST3	0.910134269	6.15332E-13
CA4	0.38797854	6.3931E-13
MGAT4C	0.109882958	6.42783E-13
SLC25A32	1.125847049	6.82322E-13
ABHD3	0.834433325	7.05887E-13
PLCD1	0.794310441	7.33591E-13
DHRS7C	0.055437561	7.72534E-13
PDE6A	0.406074548	7.93792E-13
UGT1A10	0.628737653	8.22568E-13

DHRS9	0.579599369	8.30525E-13
UQCRFS1	0.919217599	8.71906E-13
PDE1C	0.642370914	9.17217E-13
HAGHL	1.889091074	9.64448E-13
ADCY5	0.562804981	9.6563E-13
SULT1B1	0.696629055	1.01587E-12
KCNE2	0.399664391	1.03166E-12
PDE2A	0.66666715	1.06647E-12
GSTM5	0.582959155	1.0951E-12
AKR1B10	0.573630476	1.11398E-12
ADH1B	0.524790789	1.13909E-12
COX8A	0.936216762	1.14228E-12
INPP5A	0.907497367	1.16462E-12
SLC9A9	0.699812975	1.19296E-12
HSD11B2	0.82127707	1.20718E-12
SCN3A	0.559047116	1.21641E-12
SLC4A10	0.333103757	1.30556E-12
TST	0.884169507	1.32093E-12
SCN11A	0.361425763	1.40218E-12
KCTD9	0.888881007	1.47755E-12
HADH	0.913736105	1.51412E-12
ATP1B2	0.617071916	1.56809E-12
PLCXD3	0.357115281	1.76769E-12
ENTPD5	0.780713539	1.86775E-12
KCTD4	0.241531655	1.97244E-12
PLCE1	0.788010538	1.97754E-12
ACAA2	0.863034646	1.9836E-12
GLT25D1	1.071766196	2.00426E-12
PLD5	0.114228067	2.11799E-12
GABRG2	0.191941792	2.12061E-12
HSD17B2	0.655540143	2.17457E-12
PANK3	0.893090817	2.18558E-12
PDE7B	0.665893368	2.19627E-12
HMGCL	0.906665446	2.2041E-12
ST6GALNAC6	0.788028332	2.77199E-12
ZADH2	0.905483591	3.06277E-12
GRIK3	0.507573861	3.22756E-12
SLC6A19	0.290706406	3.44392E-12
SDHD	0.913622392	3.44489E-12
GALNTL1	0.499863561	3.59795E-12
GRIN2A	0.327107124	3.65194E-12
TAT	0.216298993	3.68724E-12
SCN2B	0.405683293	4.05616E-12
ATP6V1D	0.934093457	4.24326E-12

ATP1A2	0.40722511	4.38089E-12
ABCE1	1.09580738	4.66365E-12
ABCB11	0.236369159	4.80433E-12
CP	0.579792301	5.00048E-12
SCNN1B	0.508363744	5.43658E-12
PPAP2B	0.877432079	5.67708E-12
UGT2A3	0.533373153	5.83348E-12
ACAD8	0.93933492	5.9361E-12
GDE1	0.938749748	6.2327E-12
PIK3C2G	0.082284371	6.9434E-12
ATIC	1.087414194	7.40474E-12
SLCO4C1	0.377600838	7.42442E-12
TRPV3	0.423555709	7.53851E-12
CACNB2	0.723857586	7.80028E-12
SRM	1.115989312	8.58658E-12
NUDT1	1.125757628	8.8386E-12
ASNS	1.148599221	9.17737E-12
SCN4B	0.693707546	9.82047E-12
CAD	1.150072859	9.99261E-12
CYP27B1	1.410467624	1.00988E-11
AASDHPPT	1.068951365	1.03759E-11
PYCRL	1.173209364	1.07644E-11
B3GALT4	0.822397048	1.10054E-11
GSTM4	0.896199708	1.1109E-11
PDE9A	0.752020645	1.16689E-11
SLC8A3	0.485096862	1.17507E-11
ABHD5	0.890168593	1.19849E-11
ATP8B1	0.885248404	1.30618E-11
KCNK3	0.506350503	1.30751E-11
LARGE	0.88619104	1.34094E-11
ACLY	1.067734508	1.4113E-11
SLC17A4	0.600232205	1.44802E-11
NAALAD2	0.590046715	1.57042E-11
SCN3B	0.560466795	1.57157E-11
GDPD3	0.736057586	1.61226E-11
SLC26A2	0.690635761	1.72185E-11
SLC25A22	1.129925326	1.73971E-11
HYAL3	1.192346101	1.75651E-11
ATP11A	1.231891253	1.80079E-11
NIT1	0.912174359	1.80523E-11
SLC30A8	0.265224805	1.9284E-11
AMPD1	0.326769342	2.11603E-11
NDUFS2	0.944832136	2.18051E-11
SLC7A11	1.384858914	2.20086E-11

DPM2	1.085726182	2.20786E-11
ABCF2	1.063880711	2.30978E-11
PLCD3	0.87283375	2.34428E-11
TPH1	0.328181169	2.38273E-11
KCNQ5	0.424499558	2.41046E-11
SLC5A6	1.179475474	2.4832E-11
DPEP1	2.383020753	2.50923E-11
PYGM	0.468927579	2.58944E-11
GART	1.092933822	2.65658E-11
ABCC1	1.124479733	2.80322E-11
ACAA1	0.907697301	2.90415E-11
BEST2	0.417424457	2.94247E-11
SIAE	0.868525596	2.99338E-11
ADH1C	0.568473577	3.10027E-11
ABCA9	0.483665678	3.11197E-11
CHAT	0.075265525	3.1923E-11
NAALADL1	0.658087133	3.64082E-11
ST8SIA3	0.1778399	3.73276E-11
ADO	1.058356195	3.86317E-11
PSPH	1.173131985	3.98268E-11
ATP5B	0.950042481	4.04264E-11
ALG3	1.082924623	4.08705E-11
PECI	0.848943778	4.21315E-11
SLC23A1	0.518929492	4.25655E-11
DPYD	0.800296782	4.26692E-11
NDUFV1	0.948840757	4.33536E-11
CAT	0.913221813	4.80306E-11
ACADVL	0.928528245	4.82661E-11
KCNN3	0.688401012	5.14635E-11
CA9	2.727125319	5.56871E-11
GALNT13	0.359553906	5.70895E-11
TRPM6	0.535489502	5.91032E-11
FECH	0.881427691	5.93381E-11
PTGS1	0.761274436	6.059E-11
ENTPD3	0.523966157	6.10105E-11
GOT1	0.921330674	6.20013E-11
ABCD2	0.599530836	6.34714E-11
ALDH3A2	0.925468443	6.51063E-11
FMO4	0.769653542	6.53331E-11
AHCY	1.119765263	7.59278E-11
KCNG3	0.427363662	7.77104E-11
ACACB	0.803325563	8.62576E-11
BTD	0.89101978	8.63464E-11
NDUFS1	0.943783077	8.67924E-11

GDPD2	0.528921451	8.7079E-11
MGAT5B	0.485945638	8.72177E-11
KL	0.676544407	8.78916E-11
ABCC8	0.384156152	8.81407E-11
SLC5A7	0.210596248	9.1158E-11
SLC9A2	0.765608415	9.46619E-11
GDPD5	1.34266371	9.69115E-11
SCLY	1.117558799	9.90153E-11
GALNT6	1.257261494	9.92834E-11
ATP6V1C2	1.306101977	1.00421E-10
KCNB1	0.285800986	1.07591E-10
CES3	0.782625392	1.11828E-10
KCNB2	0.416057946	1.1476E-10
EBPL	1.100004089	1.22133E-10
P2RX4	0.87403871	1.2575E-10
ABCA6	0.53214819	1.32344E-10
P2RX1	0.668381801	1.49075E-10
COQ9	0.934450912	1.55063E-10
FMO5	0.788154142	1.56174E-10
ALAD	0.933167424	1.57808E-10
SUCLG1	0.92328872	1.64803E-10
B4GALNT2	0.408583941	1.64855E-10
SLC26A3	0.574549545	1.65816E-10
FOLR2	0.736408813	1.70663E-10
PPA1	1.087236411	1.71026E-10
GRIK5	0.493523613	1.76111E-10
ACSM5	0.407962165	1.79377E-10
SLC10A4	0.422897366	1.88948E-10
CMBL	0.863349219	1.9479E-10
RYR3	0.533813273	2.04228E-10
LTC4S	0.576492765	2.09634E-10
HTR3C	0.125079295	2.25909E-10
MAOA	0.839740319	2.3153E-10
VKORC1L1	1.048739635	2.37002E-10
MTHFD2	1.139857005	2.38159E-10
SLC35A4	0.952501272	2.39928E-10
ABP1	0.868477372	2.52065E-10
GPX3	0.775458089	2.5469E-10
NDUFS7	0.943643573	2.57389E-10
SLC6A16	0.409439193	2.65349E-10
TCN2	0.841889692	2.69011E-10
DGAT2	1.2440436	2.73584E-10
UGT1A1	0.42309796	2.75316E-10
KCNMA1	0.698841735	2.91843E-10

SLC30A4	0.794445966	3.11951E-10
ST6GALNAC3	0.766081658	3.27563E-10
XDH	0.743377797	3.42578E-10
CES2	0.842514394	3.50037E-10
HTR3A	0.382997176	3.54215E-10
SCNN1G	0.345906962	3.68343E-10
NAT9	1.105598131	3.74178E-10
HS3ST6	0.168262644	3.82115E-10
SLC22A17	0.751847628	4.09453E-10
NUDT16	0.923700244	4.1129E-10
UGT1A9	0.40079618	4.28494E-10
SORD	1.162816143	4.31015E-10
PGM5	0.694002412	4.37376E-10
PAPSS2	0.854640386	4.50913E-10
OGFOD1	1.068803439	5.0124E-10
PDE4D	0.855427933	5.174E-10
PTEN	0.951799986	5.17606E-10
ATP5J	0.91326483	5.39472E-10
GALM	0.908129882	5.74692E-10
DHRS1	0.905290145	5.98181E-10
SLC44A4	0.881366455	6.29921E-10
NEU4	0.682163796	6.51976E-10
HMGCS2	0.718867482	6.53273E-10
STAR	0.64511263	6.63093E-10
NAT1	0.847580791	6.645E-10
KCNAB1	0.686201197	6.75326E-10
GSTM2	0.829140235	6.83627E-10
AGXT2L2	0.887749194	7.21182E-10
ATP9B	0.928096645	7.46022E-10
RPIA	1.093261897	7.47932E-10
B3GNT8	0.858233482	7.51202E-10
HSD17B11	0.902408366	7.51941E-10
SLC12A8	1.17318309	7.57106E-10
SMPD4	1.060785439	7.58534E-10
IMPDH1	1.14325397	7.6829E-10
IMPA2	0.896181426	8.02148E-10
ATP5F1	0.930809716	8.39845E-10
IDH3A	0.924980372	8.53989E-10
HIBCH	0.920592242	8.80495E-10
TGDS	1.13882153	8.83337E-10
SLC12A2	1.115903045	8.97123E-10
PTGR1	0.916219907	9.40622E-10
PLCL2	0.75178954	9.85792E-10
PHYH	0.897270326	9.90629E-10

COX5A	0.928116384	1.02313E-09
UCKL1	1.101749173	1.13223E-09
CYP11A1	0.410178779	1.13262E-09
CA12	0.810031459	1.21634E-09
PTDSS1	1.070637247	1.25917E-09
NT5DC2	1.12658816	1.29002E-09
SLC22A5	0.849729317	1.33075E-09
AQP4	0.142916516	1.35234E-09
PDE3A	0.711565569	1.3798E-09
NAT6	1.120801283	1.39496E-09
KLB	0.624899164	1.46458E-09
NMNAT1	0.904300834	1.46979E-09
CPT1A	0.9014383	1.53106E-09
SLC7A1	1.119263518	1.54073E-09
SLC4A2	1.086961817	1.61312E-09
DHRS2	2.746910579	1.6414E-09
ATP2B2	0.34702	1.67778E-09
MPI	0.917568507	1.81526E-09
PGAM5	1.087576111	1.82129E-09
SLC35F1	0.583998777	1.90566E-09
B3GNT7	0.719015012	1.94848E-09
SLC35D1	0.901065599	1.95431E-09
SLC41A2	0.839479657	2.08385E-09
KCTD2	0.941621932	2.08891E-09
GAD1	4.796642711	2.12337E-09
SLC1A5	1.100686775	2.14819E-09
ATP2C1	1.038960309	2.1588E-09
IMPDH2	1.058352209	2.17166E-09
CYP4F12	0.814341051	2.18506E-09
COX19	1.085502889	2.19361E-09
MFSD11	0.921839177	2.21394E-09
NME1	1.143443076	2.26962E-09
AGL	0.917784526	2.28893E-09
DGKZ	1.068881482	2.31688E-09
LIPG	1.146309367	2.3326E-09
NANP	1.170943757	2.33811E-09
CHST8	0.289971401	2.35454E-09
LIPC	0.427034404	2.52418E-09
ASPG	0.392580931	2.59149E-09
SLC2A1	1.151663638	2.60504E-09
SLC7A6	1.110171312	2.65041E-09
RBKS	0.834536644	2.70505E-09
MFSD4	0.758463324	2.7332E-09
UQCRC2	0.94820664	2.7516E-09

ACADSB	0.914587323	2.76862E-09
KCNJ14	1.411031915	2.84911E-09
ATP13A4	0.49090659	3.03158E-09
AKR1C2	0.726088018	3.03349E-09
AUH	0.921984945	3.04165E-09
ATP5G3	0.936794565	3.16251E-09
HCN4	0.313077974	3.25226E-09
NOX4	2.202157006	3.29797E-09
ATP2B3	0.206716224	3.3547E-09
DHCR7	1.122675314	3.56318E-09
B3GALT5	0.678354105	3.68378E-09
SLC46A3	0.859116307	3.72743E-09
NUDT9	0.94841078	3.73644E-09
MTHFD1	1.077404096	3.89657E-09
DSEL	0.747007166	4.05807E-09
CHRNB2	0.554842717	4.08351E-09
SDHB	0.93627705	4.09386E-09
ADCY3	1.119688364	4.15648E-09
SLC12A9	1.08553112	4.21188E-09
PDE8A	0.907607815	4.33169E-09
DPYSL5	0.32455773	4.55407E-09
NAT2	0.767636883	4.57894E-09
EPHX1	0.889409572	4.60612E-09
SLC35E4	1.157548043	4.6174E-09
KCNH8	1.555632654	4.67638E-09
HSD3B2	0.261028204	4.74093E-09
SLC38A7	1.100283127	4.7606E-09
GRIN2B	2.088790913	4.82042E-09
ITPKA	0.793351716	5.11397E-09
CYBRD1	0.829405442	5.25467E-09
ACSL6	2.195253209	5.34501E-09
PIGS	0.936007395	5.45293E-09
TPO	0.461871693	5.49225E-09
BHMT2	0.596205308	5.49455E-09
UAP1	0.931828367	5.52951E-09
MOGAT2	0.608031465	5.55584E-09
ST8SIA1	0.683327775	5.7508E-09
ECHS1	0.953149177	5.7728E-09
BDH1	0.908161048	5.90585E-09
CKMT1B	0.892251012	6.00105E-09
FPGS	1.084126206	6.15025E-09
ACER3	0.927694603	6.21296E-09
PLA2G10	0.781081781	6.37085E-09
ENPP2	0.847596961	6.39277E-09

AKR1C4	43.42058115	6.71146E-09
SLC25A35	0.88909961	6.71606E-09
GCNT4	0.676686957	6.78274E-09
AQP7	0.652744666	7.04519E-09
GRIA1	0.395772352	7.23957E-09
CRYZL1	0.905053833	7.4073E-09
SLC19A2	1.107323182	7.47956E-09
GNPTAB	0.928706785	7.61193E-09
ADH6	0.714519906	7.61534E-09
AKR1C1	0.750457552	7.94066E-09
SLC11A2	1.098914858	8.1301E-09
PCK1	0.731575334	8.13026E-09
KCNG1	0.593190114	8.19876E-09
ABHD6	0.902119506	8.23421E-09
ATP5D	0.948286751	8.85627E-09
MSRA	0.8932445	8.90854E-09
SLC16A9	0.722310577	8.9589E-09
SVOP	0.342290625	9.49025E-09
RRM2	1.141499399	9.53224E-09
GABRA2	0.626076472	9.71309E-09
HS2ST1	1.063717725	9.73253E-09
PIGZ	0.805090761	9.82474E-09
GCNT3	0.760066094	9.96824E-09
GLA	1.130380437	9.97081E-09
KCNC1	0.473920846	1.02195E-08
IMPA1	0.906464622	1.02391E-08
B3GALT6	1.072765879	1.07914E-08
ELOVL4	0.659830264	1.10085E-08
TCN1	4.795513881	1.12109E-08
GBA	0.935814768	1.15283E-08
ATP2A3	0.854682954	1.16214E-08
GAPDHS	2.202536401	1.20713E-08
PHYHD1	0.686499161	1.20916E-08
GALNT12	0.885657266	1.21583E-08
PDE1B	0.73885045	1.23637E-08
SLC35A3	0.90048363	1.2398E-08
MAOB	0.713904959	1.34242E-08
EXTL1	0.495640618	1.35453E-08
FH	0.94997388	1.38648E-08
UMPS	1.063417558	1.39779E-08
GRIK2	0.540468399	1.42719E-08
ST3GAL2	1.094198934	1.42815E-08
CNGA3	0.426018123	1.46408E-08
KCNMB1	0.666185269	1.47772E-08

PLCG1	1.099432709	1.51149E-08
ABCB5	0.305599341	1.56093E-08
KCNS3	0.82384162	1.57385E-08
SLCO2A1	0.838076447	1.60362E-08
AOC3	0.792003261	1.66386E-08
ETFA	0.931625246	1.70572E-08
THNSL1	1.097366595	1.72417E-08
FUT9	0.038536223	1.73698E-08
CYP4V2	0.9180224	1.74297E-08
PFAS	1.090640682	1.76621E-08
LASS2	1.036055465	1.78576E-08
NIT2	1.091204629	1.80992E-08
NANS	0.917761263	1.83606E-08
ALG10	1.101926518	1.84614E-08
EHHADH	0.891503285	1.85757E-08
SLC35B2	1.055936114	1.87547E-08
GRIA2	0.19287859	1.88051E-08
NPR1	0.754372432	1.91389E-08
CYP21A2	0.545234601	1.94116E-08
MDH1	0.94438826	1.97744E-08
DHODH	1.089110078	2.04371E-08
GNPDA1	1.095765706	2.10624E-08
COQ7	0.954877272	2.17586E-08
CYP4B1	0.375349307	2.26292E-08
COMT	1.076441475	2.27561E-08
BPNT1	0.931211332	2.28871E-08
ENOPH1	1.07866539	2.40847E-08
FOXRED2	1.127676514	2.436E-08
KCNA3	0.616508334	2.50657E-08
HS6ST3	0.291768697	2.51999E-08
CLCN7	1.072927831	2.55242E-08
KCNT2	0.623345229	2.56733E-08
OSTalpha	0.539053374	2.57809E-08
CLIC5	0.836831515	2.6933E-08
NDUFS3	0.942813765	2.69445E-08
MBOAT1	0.892844514	2.69469E-08
BAAT	3.897096419	2.69564E-08
FLVCR2	0.770289901	2.77853E-08
GALK1	1.131713246	2.81157E-08
GLRA4	0.39374566	2.88873E-08
LRAT	0.285971199	2.92342E-08
SLC2A4	0.687879053	2.93369E-08
SLC18A3	0.26717117	2.94363E-08
MOCS1	0.804764652	2.97473E-08

ELOVL6	0.888552103	2.97754E-08
PIK3R2	1.052073528	3.00888E-08
CACNA2D3	0.536524367	3.03769E-08
CACNB1	0.857307896	3.0743E-08
COX7C	0.936110128	3.1983E-08
FIG4	0.947648506	3.46E-08
GABRE	1.354321411	3.488E-08
ME2	0.907910732	3.4975E-08
MFSD10	1.057904412	3.50463E-08
TRPC6	0.755519441	3.54051E-08
ATP8A1	0.87031108	3.57541E-08
SLC29A2	1.105424044	3.58006E-08
SCD	1.190452324	3.60763E-08
GSTA1	0.569479873	3.62653E-08
KCNK10	0.721870798	3.70021E-08
NSDHL	1.110810202	3.83676E-08
CHRNA3	0.653849996	3.83802E-08
OGDHL	0.505525372	3.86773E-08
ECH1	0.915572748	3.91646E-08
ACBD4	0.914571408	4.09462E-08
ACOX1	0.90218222	4.19809E-08
GFOD2	0.959225615	4.20773E-08
ATP6V1F	1.088618891	4.31005E-08
GABRG1	0.109856681	4.34764E-08
SLC36A1	0.860280647	4.42491E-08
NDUFB1	0.90840675	4.57836E-08
ALDH6A1	0.889648989	4.63005E-08
G6PC3	1.0524365	4.70658E-08
ALDH4A1	1.181447737	4.8491E-08
ATP5O	0.93425028	4.87911E-08
SLC22A18	0.907158601	4.90184E-08
ADH5	0.930755608	5.03327E-08
MAN1A1	0.90078942	5.19002E-08
CLIC2	0.829841524	5.19141E-08
ENO1	1.052515841	5.21977E-08
FASN	1.119556687	5.29076E-08
SLCO1B3	4.101734861	5.53923E-08
NDUFA6	0.926893123	5.56353E-08
SLC25A12	0.924230623	5.64105E-08
KCTD14	1.137674606	5.7474E-08
KCNA5	0.551021612	5.75132E-08
CLCN5	1.129183458	5.95678E-08
SGPP1	0.915783006	6.03468E-08
SLC35C2	1.071361251	6.42337E-08

SQLE	1.188505861	6.49058E-08
TTYH1	0.56623368	6.54401E-08
ITPR1	0.849134077	6.61494E-08
ATP1B3	0.935221912	6.82288E-08
CTPS	1.12560392	7.16604E-08
CHI3L1	1.701853646	7.16995E-08
SLC25A15	1.1099441	7.30832E-08
KCTD12	0.862223298	7.83779E-08
SLC6A2	0.247605782	8.07866E-08
GCK	0.565495257	8.20231E-08
ARSA	0.922342909	8.22288E-08
CYP2S1	1.136742409	8.37158E-08
MGAT4A	0.902306045	9.0137E-08
PLCD4	0.761320307	9.08138E-08
HGD	0.794545268	9.11994E-08
DIRC2	0.912077295	9.29676E-08
LPCAT1	1.104051618	9.43506E-08
MMAA	0.930808821	9.6445E-08
PTGES3	1.042984227	9.69769E-08
SMPD3	0.877846055	9.93303E-08
HYAL2	1.06659728	9.98186E-08
DLD	0.963452905	9.99582E-08
CYBASC3	0.930273119	1.03901E-07
AOX1	0.684911424	1.06602E-07
RYR1	0.60233996	1.08243E-07
VDAC2	0.959125204	1.10161E-07
SLC39A6	1.075908285	1.16073E-07
SLC11A1	1.445558301	1.16342E-07
ACSF2	0.856230592	1.16485E-07
ACSS2	0.919513842	1.17725E-07
PRPS1	1.112998651	1.1832E-07
LASS1	0.47257999	1.19266E-07
SLC44A1	0.946846926	1.19478E-07
NUDT5	1.088213359	1.20137E-07
GLOD5	0.779365278	1.24289E-07
CA14	0.477960599	1.2433E-07
GRIN3A	0.725174139	1.24609E-07
LPO	12.84558135	1.25231E-07
ACSBG1	0.620308741	1.25621E-07
PYCR2	1.048076585	1.2731E-07
UGT2B15	0.654850541	1.28813E-07
INPP5K	0.928822301	1.34059E-07
CLCA1	0.645720433	1.37169E-07
ACBD7	1.385084554	1.37172E-07

DTYMK	1.095494873	1.37931E-07
GBA2	0.931183624	1.38162E-07
ACOT1	0.885135502	1.38471E-07
GPI	1.048101219	1.41916E-07
NUDT22	0.939546649	1.42206E-07
ADCY2	0.623645254	1.46152E-07
PDE7A	1.090700763	1.46428E-07
COX10	0.93836064	1.47912E-07
ADCY9	0.8881868	1.47991E-07
PRDX1	1.042083934	1.53548E-07
ABHD8	0.864289948	1.53758E-07
SLC25A11	0.931622016	1.54949E-07
SPNS1	1.054836973	1.56053E-07
PNPLA3	2.65381637	1.56495E-07
ATP6V1E2	1.226351703	1.6349E-07
HYI	0.891629016	1.66571E-07
ATP8A2	0.580223207	1.68454E-07
BDH2	0.879547086	1.70945E-07
RDH16	2.296832271	1.7105E-07
BCO2	0.756187831	1.71393E-07
AMT	0.813085835	1.74324E-07
CACNG7	0.176161001	1.7465E-07
NDUFA10	0.955781034	1.7559E-07
MOCS3	1.114748162	1.76457E-07
CLYBL	0.887087697	1.81802E-07
GLRX5	0.952297788	1.83352E-07
ALAS2	0.249242785	1.8404E-07
MLYCD	0.941291997	1.85466E-07
CHST5	0.684265955	1.874E-07
COX7A1	0.760820177	1.87497E-07
PIK3CG	0.760542416	1.92232E-07
CLCNKB	0.270286458	1.94525E-07
SLC25A24	0.948255805	1.965E-07
DCTD	1.05214006	1.97522E-07
PECR	0.867265076	1.9768E-07
KCTD8	0.235656905	2.00091E-07
SLC25A14	1.121005215	2.01485E-07
ATP2B1	0.930086784	2.0284E-07
KCNA4	0.047914341	2.05022E-07
MGLL	0.914914983	2.07039E-07
SEPHS1	1.044005025	2.09835E-07
NUDT10	0.60929934	2.11259E-07
CHSY3	1.33299783	2.114E-07
SLC29A3	1.085814414	2.13419E-07

SLC1A1	0.82263613	2.16121E-07
ACBD6	1.071369972	2.28902E-07
KCNA1	0.241597291	2.29712E-07
CLCA2	0.443765589	2.30158E-07
NDUFB3	0.93179882	2.32332E-07
ENTPD8	0.742556993	2.32571E-07
ACOT2	0.885766894	2.35513E-07
CDO1	0.560496263	2.35856E-07
AK5	0.502520992	2.39507E-07
INPP5D	1.136105165	2.43202E-07
NAGA	0.949181729	2.52901E-07
SCN1B	0.807243073	2.63329E-07
ACADL	0.262877677	2.66308E-07
SMS	1.06936366	2.68395E-07
MSRB3	0.790448481	2.77468E-07
KCTD18	0.954270231	2.77609E-07
PRDX4	1.101561096	2.82437E-07
PIP5K1B	0.907485136	2.84632E-07
SRR	0.908711092	2.86648E-07
DIO2	1.342056552	2.90692E-07
AQP11	0.835313314	3.00182E-07
GGT6	0.786401882	3.0089E-07
CACNA1D	1.211706112	3.06997E-07
SLC4A5	1.139358194	3.109E-07
SLC19A1	1.124579948	3.13955E-07
GSTP1	1.055442095	3.18073E-07
DOLPP1	0.93004123	3.23055E-07
GNPNAT1	1.076249645	3.24225E-07
KCNMB2	0.487899404	3.41244E-07
ST8SIA6	0.427032853	3.44187E-07
SLC5A11	0.332194491	3.52005E-07
SLC25A4	0.883678673	3.52923E-07
ACSM1	0.617753467	3.61434E-07
ABCA10	0.639538014	3.64068E-07
SCN4A	0.483803211	3.64979E-07
KCNJ11	1.211445984	3.73903E-07
SLC24A4	0.634361212	3.78564E-07
SLC10A2	0.07437675	3.80523E-07
GALNTL2	0.704864275	3.80728E-07
ACSL4	1.091998602	3.91664E-07
CACNA1A	0.670671442	4.04521E-07
SPHK1	1.263930543	4.12904E-07
MFSD7	0.786890086	4.20939E-07
TRPM2	1.227839465	4.23849E-07

CNGA1	0.725705077	4.31548E-07
TRPV4	1.462817743	4.32155E-07
HSD17B6	0.758929347	4.38345E-07
LPCAT4	0.924076757	4.41741E-07
GABRB2	0.549031555	4.43525E-07
NDUFB10	0.94738606	4.49414E-07
DPAGT1	1.048275657	4.64515E-07
CDS1	0.92949644	4.76703E-07
NDUFB8	0.939767368	4.81246E-07
NEU3	1.115269611	4.87092E-07
KCNF1	0.594908337	4.98531E-07
UGT3A2	0.366557609	5.0125E-07
SLC13A2	0.597862178	5.03068E-07
NDUFS4	0.919725558	5.05742E-07
ATP5I	0.932756356	5.21514E-07
SLC9A7	1.633260847	5.42273E-07
SLC47A1	0.716978977	5.60649E-07
UST	0.728897299	5.65137E-07
HPRT1	1.100957455	5.6997E-07
CLCN2	0.862726016	5.81379E-07
SRD5A1	1.105316796	5.84744E-07
SLC10A1	0.333019284	5.87919E-07
MCOLN2	0.792994329	6.20722E-07
ACOT11	0.896412497	6.26684E-07
HMOX1	0.866353491	6.31171E-07
ENOX2	1.109555888	6.33158E-07
SLC41A1	1.091340753	6.36438E-07
SLC22A23	0.883009565	6.45829E-07
SLC17A5	0.926637254	6.61941E-07
AFMID	1.095710006	6.86004E-07
KCNS2	0.385194044	6.88988E-07
ABCC2	1.761219621	6.9346E-07
CTPS2	1.072417004	7.00626E-07
KCTD10	0.954956126	7.04855E-07
PLCL1	0.798004151	7.15896E-07
SYNJ1	0.915632961	7.49602E-07
LIPH	0.892302033	7.72818E-07
SLC35D3	2.026396713	7.82232E-07
PIGL	1.145581796	7.88401E-07
CHRNA4	0.091459309	8.00571E-07
SLC22A11	3.110753255	8.01395E-07
SMPDL3B	0.904881768	8.03182E-07
RHCG	1.982347217	8.23011E-07
SLC25A42	0.860144769	8.29463E-07

GRID1	0.792980778	8.54052E-07
ALDH1A1	0.824139005	8.94045E-07
NDUFC1	0.936700032	9.17568E-07
PAH	5.824994263	9.57363E-07
PDE4C	0.806762414	9.79289E-07
CHPF2	1.044364068	9.84858E-07
GPLD1	0.69605501	9.92709E-07
ATP13A3	1.048495479	9.9973E-07
SV2B	0.679370505	1.00152E-06
SLC6A20	1.600970941	1.04008E-06
PNPO	1.065135726	1.07127E-06
RHBG	17.51045595	1.07969E-06
SLCO5A1	1.489296047	1.081E-06
TF	0.486104489	1.09153E-06
ABCC10	1.077544583	1.09989E-06
ADAL	0.849235238	1.12941E-06
G6PD	1.069237577	1.13369E-06
CYP26B1	0.824367633	1.16376E-06
SLC15A2	0.837033646	1.18473E-06
CES1	0.767055833	1.18714E-06
CCBL1	1.162966329	1.20183E-06
SLC22A3	1.262964763	1.20896E-06
FAAH2	1.129952372	1.22217E-06
PIK3C3	0.950228375	1.22551E-06
PLCG2	0.818239775	1.23822E-06
PDHB	0.955133028	1.23917E-06
BST1	0.809151309	1.26212E-06
MFNG	0.87462258	1.27727E-06
PDE3B	0.909454066	1.30755E-06
CLCNKA	0.219507625	1.32665E-06
GPX2	1.085968449	1.35869E-06
SPTLC3	0.780428966	1.36749E-06
COX4I1	0.9498541	1.36957E-06
NDUFV2	0.929173198	1.39849E-06
SLC38A4	0.612664805	1.41156E-06
MCOLN1	0.940301412	1.42434E-06
DHRS4L2	0.914521402	1.42826E-06
SLC39A5	0.848805625	1.45106E-06
SLC6A1	1.375330665	1.4635E-06
PTDSS2	1.039580931	1.47102E-06
SLC5A8	0.258518474	1.49857E-06
ATP8B4	0.819856427	1.52016E-06
PPAPDC1A	1.968057117	1.56048E-06
SLC25A18	0.715221669	1.5788E-06

SRXN1	1.081053868	1.58576E-06
LPCAT3	0.926360067	1.60385E-06
DMGDH	0.606944557	1.62265E-06
CACNA2D1	0.602523468	1.62754E-06
ATP5L	0.950450953	1.6851E-06
C1GALT1	1.070532231	1.69177E-06
SLC6A9	0.869981378	1.71951E-06
MBOAT4	0.573025874	1.74567E-06
IP6K3	0.632657182	1.75105E-06
SLC13A1	0.067473247	1.76466E-06
PDE1A	0.748434456	1.79981E-06
DPEP3	0.378291862	1.81495E-06
GLRX3	1.070747146	1.84365E-06
PLA2G5	0.614038912	1.85386E-06
KCNH1	0.728743627	1.89548E-06
SULT4A1	0.473182488	1.89996E-06
MTHFR	0.928161501	1.94274E-06
SLC4A1	0.132384833	1.97303E-06
ATP6AP1	1.03294433	2.0024E-06
PKM2	1.049592724	2.02651E-06
AKR7A2	0.948095452	2.02847E-06
GALNT1	0.949461752	2.03482E-06
TH	2.543676747	2.03803E-06
ATP6V1C1	1.048900162	2.08384E-06
SLCO1C1	0.482497312	2.09445E-06
ATP2B4	0.854101516	2.10072E-06
PISD	1.040036167	2.11384E-06
SLC39A12	0.212610071	2.12982E-06
ACSM2A	0.169406287	2.13777E-06
DPEP2	0.719443084	2.15119E-06
ODC1	1.074160598	2.21759E-06
CHST4	3.051972724	2.22042E-06
SLC9A3	0.591133754	2.26683E-06
PGK1	1.059088648	2.34158E-06
DHRS4	0.928863909	2.35312E-06
SLC44A2	0.958082993	2.37388E-06
NME2	1.049782951	2.3901E-06
CROT	0.863296816	2.39772E-06
SLC25A17	1.056379844	2.41295E-06
ENOSF1	0.926323025	2.4441E-06
SI	0.581067371	2.45683E-06
AADAC	2.903311848	2.45938E-06
HTR3B	0	2.55923E-06
KCNK2	0.506929902	2.57148E-06

PIGW	1.083070185	2.61121E-06
ST3GAL3	0.839012096	2.61672E-06
NDUFA5	0.950979059	2.65846E-06
NDUFA1	0.946672576	2.69898E-06
MDH2	1.028049124	2.70576E-06
SLC35A5	0.956365196	2.72571E-06
ALDH2	0.961294854	2.73485E-06
WBSCR17	0.665574774	2.76676E-06
POMGNT1	1.042742859	2.82635E-06
SLC9A1	0.910750593	2.83937E-06
DDT	0.942683745	2.84294E-06
NME5	0.588050718	2.91512E-06
NMRAL1	1.061679097	2.9155E-06
LPGAT1	1.062684652	2.92475E-06
RDH10	1.076295942	2.95045E-06
SLC18A2	0.673364201	2.95548E-06
SLC20A2	0.924241403	3.02693E-06
A1CF	0.861510236	3.05303E-06
FXN	1.072264764	3.1934E-06
ALAS1	0.954607881	3.2244E-06
CECR1	0.874926869	3.2495E-06
SLC35F3	0.441140512	3.27274E-06
ATP5H	0.954862383	3.38719E-06
NOS1	0.348764525	3.40887E-06
SLC13A3	1.916343344	3.43465E-06
FLVCR1	1.115786039	3.52899E-06
KCNA2	0.56184018	3.53104E-06
UEVLD	0.942558318	3.74184E-06
HPD	0.417712147	3.7478E-06
SLC28A2	0.541676998	3.804E-06
AKR1C3	0.864784534	3.84232E-06
CYB5A	0.927668302	3.91967E-06
SLC17A1	0.232411342	3.99794E-06
LPCAT2	1.086630281	4.00865E-06
B4GALT3	1.040535125	4.02284E-06
CYP3A4	0.502717743	4.1432E-06
ATP5G1	0.939957752	4.16356E-06
ALG1	1.049253801	4.2006E-06
MAN2B2	0.95260607	4.33591E-06
PFKFB3	1.066264907	4.43991E-06
MME	1.32188699	4.50597E-06
SLC15A1	0.603666746	4.50601E-06
PNLIPRP2	0.637892206	4.52338E-06
PCYOX1	0.962068332	4.56552E-06

KCNA6	0.701295687	4.57843E-06
ACOX3	0.940462936	4.63941E-06
PIGU	1.077106275	4.6689E-06
SLC31A2	0.892646415	4.6894E-06
SLC41A3	1.041858197	4.74029E-06
CHRNB4	0.512917166	4.87545E-06
TALDO1	1.031109162	4.93605E-06
ACCN2	1.243769245	5.01725E-06
UXS1	1.03579909	5.05569E-06
RHAG	0.02704083	5.30753E-06
HVCN1	0.839671476	5.35859E-06
PLD1	0.919381293	5.49332E-06
ALG12	0.957470276	5.52722E-06
ABCD4	0.937928933	5.63258E-06
PCBD1	1.051844638	5.63689E-06
FMO2	0.619258742	5.73861E-06
KCNK9	2.287223346	5.89846E-06
SLC2A5	0.793843512	5.91276E-06
OGDH	0.953875493	5.9594E-06
PIGN	0.932813415	6.10701E-06
HSD17B7	1.109039398	6.11527E-06
ALDH3B2	2.106773253	6.12841E-06
KCNJ12	0.687438204	6.14462E-06
GSTT1	0.928557223	6.23449E-06
INPP5E	1.064720941	6.24659E-06
SLC26A7	0.575546281	6.48577E-06
SLC43A1	1.100278942	6.6132E-06
ACSM3	0.869554106	6.78913E-06
GSTZ1	0.890026411	6.82167E-06
COX6C	0.955599888	7.06889E-06
MAN1C1	0.850882429	7.09143E-06
ALDH9A1	0.968395726	7.1042E-06
TRPM4	0.914296338	7.10762E-06
HLCS	1.066900598	7.18416E-06
SLC27A5	1.187503134	7.24291E-06
CLCN4	1.203725625	7.2436E-06
FDX1	0.948992369	7.24766E-06
ABCB1	0.817263193	7.42754E-06
KCTD13	1.079991237	7.4802E-06
SLC15A4	1.050030788	7.491E-06
TPK1	0.875803887	7.56375E-06
NUDT7	0.920268232	7.64262E-06
HKDC1	1.158453089	7.72169E-06
ETFB	0.952562836	7.79561E-06

ABCA5	0.864807824	7.87102E-06
B4GALT7	1.040158924	7.93599E-06
NUDT6	0.861434313	7.94755E-06
GSTCD	1.081121965	7.95979E-06
ENPP4	0.954822032	8.01133E-06
ALG11	1.222652923	8.07306E-06
ENPP1	0.843698708	8.25784E-06
PPAPDC3	0.778183074	8.36426E-06
MGAT1	0.965732014	8.59057E-06
ATP13A1	1.046720196	8.67728E-06
AQP1	0.900417528	8.69066E-06
DPM1	1.07874769	8.86114E-06
PMVK	0.94853394	8.88911E-06
PMM1	0.928197813	8.89307E-06
ECHDC3	0.794373978	8.92439E-06
SLC1A4	1.064001057	9.01471E-06
PTGIS	0.783889698	9.06873E-06
CDA	0.844649267	9.1253E-06
CEL	1.806935172	9.38053E-06
SLC25A19	1.065316735	9.75159E-06
ECHDC2	0.87959293	9.86809E-06
SLC35B3	0.943409937	9.89032E-06
SLC29A1	1.099850967	1.00443E-05
DECR1	0.944828276	1.02133E-05
PC	0.876994976	1.03947E-05
CHST7	0.848808727	1.05975E-05
DAK	1.053758019	1.07372E-05
FAR1	0.962171662	1.07968E-05
FAHD1	0.950507651	1.1085E-05
SLC25A3	0.972388326	1.12042E-05
SLC16A14	0.839233787	1.12053E-05
NAT8B	0.54711615	1.12524E-05
COX5B	0.939714305	1.12883E-05
SLC7A2	0.804511757	1.13858E-05
HSD17B10	1.061632723	1.13899E-05
B3GALNT2	1.048053943	1.14957E-05
COX6A1	0.952554756	1.15113E-05
PFKFB2	0.912646708	1.18033E-05
ALG8	1.055563286	1.18713E-05
NUDT21	1.032964612	1.23878E-05
MGAT5	1.114020644	1.24435E-05
SLC26A4	0.711259663	1.28477E-05
SLC35D2	0.942538322	1.314E-05
LST-3TM12	19.77305031	1.31948E-05

ALDH1L1	0.70229113	1.33022E-05
B3GNT6	0.650076252	1.33546E-05
BLVRB	0.936600002	1.34738E-05
COQ10B	0.954758192	1.36756E-05
TPMT	0.942354585	1.3846E-05
ITPK1	0.966437228	1.38851E-05
UCK1	0.959240613	1.3955E-05
TREH	0.589635675	1.40123E-05
GAPDH	1.029343144	1.42845E-05
DERA	0.941145461	1.44969E-05
THTPA	0.932878448	1.48732E-05
FLAD1	1.047314569	1.48861E-05
MICAL2	0.944417383	1.49786E-05
AKR1B1	0.892182739	1.51934E-05
KCTD1	0.865152138	1.52498E-05
DGKA	0.901725996	1.54837E-05
NDST4	0.220771167	1.55274E-05
KCNH4	1.881084887	1.59653E-05
CMAS	0.953657174	1.62056E-05
SLC9A5	1.344984786	1.62138E-05
PIP5K1C	0.948690843	1.65157E-05
ACOT9	1.077438572	1.66159E-05
CHRNA1	0.65186104	1.67515E-05
FUT8	1.095790753	1.75165E-05
STARD13	0.918351439	1.764E-05
LDHAL6B	0.530165758	1.77557E-05
SLC8A1	0.84895903	1.80418E-05
IVD	0.965362136	1.82886E-05
CHSY1	1.055204134	1.85758E-05
B3GNT1	0.930797865	1.85867E-05
TRPM5	0.674557223	1.86282E-05
TRPC1	0.779441602	1.86595E-05
CRAT	0.90298898	1.88127E-05
SLC25A29	1.102823364	1.97934E-05
SLC34A3	2.101691261	1.98413E-05
NDUFA4L2	1.207453128	1.99251E-05
PYROXD1	0.951786435	2.01143E-05
GSR	0.947019639	2.01478E-05
HDC	0.743941919	2.02069E-05
DCI	0.949935533	2.03722E-05
CYP8B1	0.502247784	2.04968E-05
PIK3CD	0.870329219	2.10728E-05
C9orf103	0.878679651	2.12481E-05
ACYP1	1.104111517	2.18287E-05

KCNN1	0.590015765	2.18653E-05
GNE	0.899322618	2.21067E-05
CYCS	0.958302033	2.22195E-05
GLS	1.051562285	2.23561E-05
AS3MT	0.761558823	2.27691E-05
SLC25A16	0.940581444	2.36433E-05
COX7A2	0.948740127	2.38253E-05
DHRS4L1	0.780961754	2.38259E-05
AQP9	1.583300842	2.39591E-05
OTC	0.624169339	2.40915E-05
NUDT12	0.859806001	2.43545E-05
PDE6B	0.675436445	2.47063E-05
PIGM	1.043324321	2.47801E-05
NDUFA2	0.940916602	2.50621E-05
SULF1	1.161728536	2.58196E-05
GSTM3	0.841763289	2.58895E-05
CYB5R3	0.970017866	2.61729E-05
ADSSL1	0.788646947	2.64603E-05
GAMT	0.78571893	2.69372E-05
FRRS1	0.84764751	2.74112E-05
HPSE	0.865994082	2.80028E-05
OAS3	1.10121882	2.82961E-05
PCYT1B	0.57472782	2.83729E-05
PCK2	0.948909331	2.84532E-05
PEMT	1.072205531	2.85153E-05
UCK2	1.048819506	2.8617E-05
DAGLB	1.042630139	2.93314E-05
NUDT11	0.721398424	2.93889E-05
SLC35A1	0.923230229	2.96123E-05
KCNV1	0.58914655	2.97956E-05
NQO1	1.095096119	2.99812E-05
PPCS	0.952398487	3.02308E-05
VDAC1	1.043661935	3.03169E-05
GATM	0.85451209	3.16218E-05
ABHD2	1.099556434	3.22099E-05
AGXT	0.629535052	3.36288E-05
PIK3R1	0.951442306	3.36496E-05
CERK	0.909337568	3.3907E-05
ADC	0.867426015	3.40398E-05
HK2	0.924499785	3.63645E-05
NDUFS8	0.967399678	3.66837E-05
AK3	0.960481652	3.70249E-05
KCND3	0.750346069	3.73231E-05
ACHE	0.858224429	3.86272E-05

EXT1	0.957608403	3.94947E-05
PLA2G2A	0.806927371	3.99987E-05
GLO1	1.05412884	4.20451E-05
SLC5A1	1.098224103	4.36925E-05
SLC2A12	1.1435891	4.3833E-05
SLC47A2	0.487319381	4.41996E-05
GLYATL1	1.776374994	4.44315E-05
SUOX	0.960038679	4.46593E-05
SLC46A1	0.895482977	4.48876E-05
TRPC7	0.160475197	4.49658E-05
ECHDC1	0.929974943	4.58649E-05
IDI2	1.081744739	4.62502E-05
ITPA	1.054234114	4.75552E-05
LDHA	1.037176322	4.78146E-05
CYP2U1	0.90479696	4.82872E-05
ACOT4	0.827197392	4.87731E-05
FN3K	0.92572159	4.88697E-05
SLC16A2	0.877215114	4.89232E-05
SULT1A3	0.93333681	4.91968E-05
SLC25A1	0.967491694	4.94485E-05
GABRB3	0.633086949	4.94882E-05
GCSH	1.368878334	5.0243E-05
ATP8B2	0.896777123	5.06735E-05
PRPS2	1.054879228	5.08483E-05
NUDT14	1.079711353	5.18653E-05
SFXN5	0.915523675	5.29187E-05
UPP1	0.921450919	5.30496E-05
PDXP	0.949001658	5.44932E-05
PCTP	0.948776932	5.4851E-05
NUDT3	1.046476454	5.50215E-05
SLC4A8	1.254365112	5.52913E-05
STARD5	0.903881172	5.64055E-05
CNDP2	0.966888191	5.68493E-05
LASS4	0.800801933	5.73114E-05
CYB5B	1.049967673	5.75015E-05
CYP4F3	1.207532912	5.78713E-05
GAL3ST2	0.824954217	5.81083E-05
HSD17B12	1.032214149	5.8397E-05
NOS3	1.14212913	5.84665E-05
GLOD4	0.958988882	5.94863E-05
SLC5A10	0.753434428	5.98021E-05
G6PC	0.130582505	6.06182E-05
ATP5C1	0.965285745	6.11779E-05
SLC37A3	1.034697948	6.36986E-05

LIAS	0.934783385	6.40914E-05
DGKB	0.510980163	6.42728E-05
TSTA3	1.082968511	6.63767E-05
MGST1	0.938257416	6.66902E-05
GSTA4	0.930454599	6.7812E-05
ADCY6	0.940080969	6.84928E-05
HAGH	0.964243311	6.88743E-05
ACACA	1.070097237	6.9055E-05
NAT15	0.96731388	6.98286E-05
AKR7A3	0.914580405	7.50308E-05
SLC6A17	0.67857669	7.5867E-05
ATP6V1E1	0.970971967	7.63819E-05
ADPGK	1.044796648	7.93928E-05
STARD8	0.880340215	7.99333E-05
CACNA1B	0.509161721	8.00693E-05
ST6GALNAC1	0.865515416	8.22453E-05
KCNN4	1.1197853	8.24554E-05
DBT	0.944241586	8.438E-05
NDUFA13	0.957718521	8.44672E-05
MAN2B1	0.960258932	8.50364E-05
COX6B1	0.965343988	8.72495E-05
ABO	0.884061227	8.81017E-05
SLC6A8	0.907499682	9.04299E-05
ABCA1	0.913926137	9.09373E-05
PIGA	1.06142599	9.1595E-05
NDUFB7	0.952389018	9.33329E-05
FMO3	1.391716265	9.65705E-05
CYP27C1	0.72643543	9.69161E-05
PGM3	1.060613842	9.84895E-05
ASAH1	0.96121899	9.95813E-05
SLC25A39	1.035795604	0.0001024
CACNA1H	0.852092506	0.000102562
UGT2B7	0.731576267	0.000104796
KCNJ9	0.437285335	0.000104814
PTGES	1.139127702	0.000104822
C9orf95	0.946978915	0.00010953
CYP2C18	0.752916515	0.000110073
SLC22A15	1.167000517	0.000111645
CLIC1	1.033692535	0.000117822
NOX5	0.497875088	0.000117931
SV2A	0.833540211	0.000119783
PFKFB4	1.108705626	0.000122644
NAGK	0.962915114	0.000124424
PLA2G3	3.172985444	0.000124582

CTNS	0.964283461	0.000126127
CYP2J2	0.928232594	0.000126282
SAT2	0.929215883	0.000126709
UGCG	0.938981894	0.000127461
SLC10A3	1.045107346	0.000128766
CHRNA6	3.298045304	0.000129223
ATP13A5	0.128863524	0.000134592
UROD	0.950810884	0.000136064
CBR4	0.944400409	0.000141529
GMPPB	0.952020366	0.000142435
ITPKB	0.926753665	0.000142959
SLC25A44	0.967852081	0.00014307
PANK4	0.9668051	0.000144522
KCNJ16	0.525320946	0.000145171
NFS1	1.059563789	0.00014818
SLC37A2	0.815423684	0.000149842
SLC32A1	0.06220937	0.00015027
APRT	1.039798068	0.000151297
PPAP2C	1.077999065	0.000151309
GLCE	1.048047799	0.000151729
PCCB	0.969594986	0.000155665
CYP19A1	1.722512846	0.000156798
NDUFB2	0.968179527	0.000157035
SLC39A1	1.023438332	0.000159563
SLC35B4	1.055868157	0.000160643
SLC16A13	1.092295417	0.000165936
SLC8A2	0.540760445	0.000171161
ACSS3	0.732338204	0.000171321
SFXN4	1.038057957	0.00017248
NPC1L1	1.734271727	0.000173176
PNPLA7	0.840817535	0.000174
AGPAT1	0.975012053	0.000175276
INPP4A	0.957841952	0.000175302
ADAD2	0.542151234	0.000175802
TBXAS1	1.09828679	0.000176308
GAL3ST1	0.839914929	0.000179673
PLA2G2C	0.054279727	0.000180887
PDE6G	0.716653488	0.000184514
TAP1	1.052776772	0.000184666
SLC22A4	0.820294349	0.000186433
PI4KA	0.963022883	0.000191384
PLD4	0.744038119	0.000193744
GSTA2	0.440635474	0.00019582
SLC35A2	1.045656092	0.000197857

AGK	1.058788277	0.000203007
P2RX7	0.807326954	0.00020467
PIK3R5	0.868636051	0.000204764
TM7SF2	1.067963273	0.000207284
PDE11A	0.773523157	0.000207321
DDO	0.779834061	0.000212286
NUDT4	1.052842985	0.000213727
ATP6V0D2	0.719074645	0.000214871
UGT1A5	0.112366917	0.000215487
CHI3L2	0.67119379	0.000216432
PI4KB	0.97661841	0.000218248
CHRNG	4.090720025	0.000219864
STARD10	0.945636747	0.000221144
PAOX	0.8915551	0.000223946
AGPAT6	1.043547766	0.000224412
SLC16A7	0.747713756	0.000225276
MAN2A1	0.945841831	0.00022919
COASY	1.030044517	0.000229938
KCNJ15	1.480475865	0.000230969
DHRS7B	0.944937515	0.000233222
SLC29A4	0.857951435	0.000241018
TDO2	1.28125091	0.000241571
SDSL	0.915692932	0.000244938
AQP2	6.466862763	0.000245448
PTGES2	1.032801098	0.000248632
PLA2G4E	2.125288745	0.000252187
EBP	1.048924792	0.000266377
ENTPD6	1.048801614	0.000269637
SLC24A2	3.119657445	0.000270968
PMM2	1.041415413	0.000274733
TTYH2	0.889698098	0.000277459
SLC45A1	0.822373254	0.000280974
KCNK12	0.603139331	0.000282337
CYB561	1.035936408	0.00028668
SLC30A7	1.040025313	0.000287228
FOLR1	1.621689016	0.000288305
ALOXE3	2.869275539	0.000289084
CACNG5	0.169629165	0.000290257
GAL3ST3	0.169071783	0.00029283
ABCC3	0.923069762	0.000293691
SLC6A12	0.771860242	0.000295011
UAP1L1	1.094653269	0.000297133
ST3GAL6	0.820445647	0.00029737
SCN5A	0.716075348	0.00029824

SHMT1	0.963984745	0.000298345
GPD2	0.966570102	0.000301948
WWOX	1.085168091	0.000305272
SLC30A9	0.96973267	0.000310795
RRM1	1.037706167	0.00031274
SLC25A41	0.645582187	0.000319102
NADK	0.967909281	0.000322904
PTGDS	0.845627444	0.000323833
SLC38A5	1.157992042	0.000330864
GSTM1	0.855735526	0.000331662
CLCN1	2.052935759	0.000336522
ATP1A3	0.746119102	0.000338839
SULT1C4	0.766851385	0.000344547
STS	0.919068147	0.000351874
SGMS2	0.949652845	0.000362966
RDH12	1.665550966	0.000371733
GALNT7	0.94972113	0.000374802
COQ4	0.962178006	0.000378276
NEU1	1.059621879	0.000382888
COX6B2	0.773849009	0.000398992
NPR3	0.607099111	0.000399618
CHST6	0.841647563	0.000402259
ARG2	1.060348479	0.000422277
PDE8B	0.824470818	0.000427247
CYB5D2	0.930941591	0.000437534
PDSS2	0.968023182	0.000438063
ENOX1	0.764650702	0.000438811
SLC26A9	5.462207122	0.00044116
IP6K1	0.97776467	0.000443822
ABHD11	1.042047277	0.000446166
UQCRB	0.967000527	0.00044951
SLC23A3	0.75691144	0.000452999
SFXN1	0.953302807	0.000454523
PAPSS1	1.035963539	0.00047029
ST8SIA4	0.895892657	0.000471212
ADK	1.06665024	0.000472432
B3GNT5	0.942941489	0.000472956
KCNV2	2.193142679	0.000476096
BCAT2	0.9543534	0.000480564
SLC25A36	1.033515766	0.0004825
SDHC	0.971436406	0.000487129
CRYL1	0.936153395	0.000490118
CTBS	0.954455422	0.000490469
GABRA4	0.658234584	0.000492204

FAR2	0.95744916	0.000492559
GALC	0.913444156	0.000495599
CYP46A1	0.703773405	0.000509947
KCNK6	0.935368339	0.000525716
DLAT	0.972283446	0.000529555
ST3GAL5	0.918468946	0.00053287
MSRB2	0.957978332	0.000533635
SULT1C2	1.313867033	0.000535013
NQO2	1.066102538	0.000540704
TK1	1.066730975	0.000545375
KCNQ4	0.722951334	0.000545967
MFSD1	0.965437402	0.000547672
HSD17B8	0.930710205	0.000560173
LYPLA1	1.056077396	0.000561575
RFK	0.946808399	0.000565923
DOLK	1.033115017	0.000566556
AHCYL1	0.976488007	0.000568574
ENO3	1.158393899	0.000569762
NDUFB5	0.959253693	0.000570198
ATP11C	1.055071188	0.000573706
UGT1A7	0.078925784	0.000575973
ACSM2B	0.17797697	0.000590623
SLC30A6	1.032105325	0.000595075
HS3ST1	1.078473259	0.000617083
HS3ST5	0.526715583	0.000619266
POFUT1	1.059323192	0.000619641
AQP12B	0.733648364	0.000624789
SLC16A4	1.1456567	0.000635356
SLC24A6	0.952941862	0.000637469
KCTD21	0.959904328	0.000639838
EDEM3	0.962153743	0.000644348
KCNAB2	1.094138565	0.000655717
PLA2G4F	0.921228745	0.000663928
PNLIPRP1	0.267982209	0.00066393
SLC38A2	1.028649009	0.000671
NDUFB4	0.968443875	0.000671227
SLC25A46	0.956176699	0.00067253
POR	1.038569906	0.000675897
CHPT1	0.960982423	0.000695449
NPC2	0.957413911	0.00074248
CA11	0.848113163	0.000746136
SLC27A4	0.955543439	0.0007509
SLC5A12	0.620705836	0.000773728
MGAM	0.67484868	0.000786393

NPR2	0.905126903	0.000788694
ILVBL	0.979092596	0.000821565
MCAT	1.031247325	0.000821822
GANAB	1.022338272	0.000822146
MCCC2	1.033110839	0.000824748
HMBS	1.040518265	0.000830778
MAT2B	0.968346779	0.000832467
GLUD2	0.974991303	0.000866079
PGD	1.035061714	0.00087056
PGAM2	0.867523846	0.00087112
LASS6	1.044195204	0.000874833
HAAO	0.882280359	0.00089012
SLC10A5	0.808322559	0.000899713
CYP2W1	1.364118762	0.000902247
PPA2	0.961571185	0.000921924
ABCC6	0.886512523	0.000942717
GALNT2	1.027194798	0.000961321
COQ5	0.96329166	0.000967817
GPX1	1.043680366	0.000979267
CBR1	0.926220888	0.000985208
MTHFSD	1.03137694	0.000989786
CANT1	0.960784962	0.000996632
CRYM	0.881762161	0.000999012
CYP39A1	1.128362825	0.001003436
NDUFV3	0.965458258	0.001008857
MGAT2	0.96854981	0.001018262
ALOX12	0.763451748	0.00102245
COX7B	0.953405852	0.001043523
CATSPER1	1.922895304	0.001054646
NAT8L	0.743083757	0.001057197
SLC28A3	1.533824331	0.001059557
ALG14	0.954328527	0.001065634
CYP27A1	0.907000727	0.001073706
HS6ST2	1.382183195	0.001080608
INMT	0.885953178	0.001081885
GRIK4	0.537907217	0.001083486
GAA	0.954128858	0.001087001
NUDT18	0.943304663	0.0010895
SLC25A27	1.142576699	0.001092794
GABRA1	0.247077957	0.001103385
ABHD12	1.048242582	0.001138876
CHID1	1.02447313	0.001142247
POMT1	0.969479405	0.001144492
CYP4F8	2.690765208	0.001162189

GANC	0.968096932	0.001168495
CACNG6	0.490293467	0.00117795
CLIC6	0.825795683	0.001188655
B4GALNT1	0.853776501	0.001189744
GFOD1	0.904192678	0.001190478
ATP6V1B1	1.355844368	0.00119936
MAT1A	1.529114022	0.001208546
GLRA2	0.639429149	0.001211738
CACNA2D2	0.816240827	0.001229161
SGPP2	0.899657062	0.001238392
UQCRQ	0.960610226	0.001252793
GUCY1A3	0.877352038	0.001282023
SEPHS2	0.958726878	0.001292742
LPIN3	1.067503499	0.001297281
ATP13A2	1.038181705	0.00129775
MGAT4B	0.964273189	0.001299927
ORAI2	1.056454554	0.001308537
THEM4	1.049305663	0.001330585
SCN1A	0.299552503	0.001334388
AK1	0.942983191	0.001339778
GLDC	0.691521988	0.001377881
ATP7B	1.081858508	0.001382846
SLC25A30	0.955184041	0.001395425
ENTPD4	0.956605682	0.001403544
GNPDA2	0.951319614	0.001417321
PIP4K2A	0.969307869	0.001419935
PLA2G12A	0.973652732	0.001450894
SLC7A3	0.466998047	0.001454717
TRPA1	0.852796826	0.001455659
B3GAT3	1.024835022	0.001468667
NDUFAF2	1.060328932	0.001477575
DHFRL1	0.959904107	0.001520079
SLC1A2	0.744715954	0.001527952
HSDL2	0.971662127	0.001533595
PIGX	1.035914142	0.00153784
B4GALNT3	0.915112127	0.001539743
KCNJ6	0.453540824	0.00154877
RDH11	1.038635233	0.001561783
CACNG2	0	0.001590295
GALNT4	1.059680085	0.001593504
SRD5A3	1.054985655	0.001603148
P2RX6	0.664281594	0.001606032
HEPH	0.946746026	0.001608355
KHK	1.081841707	0.001621567

CRLS1	1.045436331	0.001668489
PIGG	0.967048328	0.00175762
NNT	0.933049914	0.00176515
SLC20A1	0.922818719	0.001768692
INPP5J	0.910614057	0.001782224
CLIC4	0.944223069	0.001787073
CLIC3	1.221460481	0.001787357
MAN2A2	1.031126918	0.001796452
ST6GALNAC2	0.85415812	0.001808975
SLC3A1	0.822929602	0.001814257
ST3GAL1	1.09021291	0.001821727
ABCB8	1.03403942	0.001837536
ABCG4	0.752032709	0.001858547
CHST2	0.918048366	0.00187482
PIGV	0.968616992	0.001883555
ABHD1	1.455126645	0.001919287
ABCB4	0.852465769	0.001929817
GGH	1.079013737	0.001955053
LIPI	0.30610023	0.001974766
ST3GAL4	0.891639524	0.001983094
KCNC2	0.311869519	0.00200649
PLA2G2D	0.741246463	0.002020308
BEST3	3.987472439	0.002025951
PRPS1L1	3.228935232	0.002037636
ATP6V1G1	1.031570661	0.002038394
PLA2G4A	0.856965338	0.002095753
LASS5	1.028333479	0.002136069
PIGB	0.966340485	0.002159042
ATP6V0A4	9.448652815	0.002177291
CHRNA2	0.212527215	0.002228027
ASL	0.962743061	0.00222996
MFSD5	0.97731372	0.00223399
COX15	0.978547078	0.002238246
CHST10	0.867238133	0.002241261
ELOVL5	1.074578814	0.002252436
MCCC1	0.972106301	0.002310712
SLC39A8	0.942203081	0.002360548
TKTL1	0.587180619	0.002377164
CSGALNACT1	1.099967049	0.002453266
GRID2	0.249920791	0.002463604
ACO1	0.96744852	0.002503541
SLC39A13	1.037054807	0.002582738
ASNSD1	1.038086121	0.002591106
NDST1	0.960516333	0.002638398

ST6GALNAC4	0.951922216	0.002642323
ARSE	1.063965528	0.002673025
B3GALT2	0.800383255	0.002753181
NT5M	0.82492194	0.002762805
GAL3ST4	0.924975697	0.002794804
UQCRH	0.968195231	0.002809583
ORAI1	1.028012617	0.002836194
GCLM	1.048615259	0.002855765
SLC35B1	1.033555165	0.002868353
PGM2	1.027312178	0.002878814
PDE10A	1.175835829	0.00288206
TPCN2	0.971870157	0.002885288
KCNMB3	1.088071141	0.002901574
ISCA1	0.970158902	0.002902272
CYP7A1	0.461777767	0.002981173
SLC30A1	0.961651957	0.003000574
SLC27A2	0.943051233	0.00302095
FUT7	1.21868619	0.003030226
ACOXL	0.697762699	0.003031077
OAS2	1.09193244	0.00305608
CYP20A1	0.974602461	0.003063084
CSGALNACT2	1.046117009	0.003110683
PLD6	1.099148911	0.003123034
NDUFB9	0.966262493	0.003152489
CYP1B1	0.829113279	0.003155945
CYP4F22	0.684634192	0.003159839
SLC40A1	0.956372028	0.003174548
OASL	0.889912956	0.003186681
GLUD1	0.98367479	0.003223633
SLC14A2	0.520293163	0.003280969
CYP4X1	1.267385774	0.003281633
SCD5	0.893315649	0.003283127
LPIN2	0.967270419	0.003342465
NDUFA12	0.970051442	0.003353885
DPYSL3	0.922483364	0.003374847
MCEE	0.934634056	0.003413231
ENPP3	0.838032931	0.003417084
RPE	1.031277852	0.00343519
NUDT16L1	0.963980713	0.003467816
IPPK	1.031259323	0.003481108
CTH	0.939638246	0.003535772
LBR	1.032936209	0.003591392
PNLIPRP3	0.102531032	0.003598244
TYRP1	0.767465289	0.003613724

CYP4F11	1.159661832	0.003639602
PCYOX1L	1.051113079	0.003644454
ADH4	0.704140889	0.003665902
ALDH1A2	0.698282036	0.003670803
CHST1	1.152641472	0.003740031
FADS2	1.106181351	0.003751126
ATP2A1	1.286885613	0.003754288
ACSF3	0.967052811	0.003765103
CHRNB3	#DIV/0!	0.003772657
RDH14	0.974587292	0.003790064
DDOST	1.017303	0.003830796
ASAH2B	1.385332455	0.0038548
XYLB	1.052009132	0.004024066
GYS1	0.978690858	0.004027112
B3GNT4	1.315996854	0.004027496
CHRNA5	1.099235502	0.004048837
ST6GAL2	0.786514419	0.004085045
GK5	0.969119194	0.004096112
BEST1	0.888549688	0.00412559
AK7	0.887400849	0.00416394
KCNE1	0.628582137	0.004171028
EDEM2	1.038570298	0.004186292
NUDT19	1.047274952	0.004203689
NIPA1	1.028343069	0.004312601
MECR	1.028143344	0.004318578
KCNJ5	0.78456564	0.004320228
SLC6A3	1.963018517	0.004425979
CYBB	0.932180732	0.004542462
DHDH	1.430048316	0.004607424
SLC18A1	0.739770789	0.004636005
ISCA2	0.954430615	0.004646584
CYP51A1	1.043436186	0.004656193
DDHD1	1.031129875	0.004704656
MFSD9	0.961535267	0.004728758
SLC22A20	1.35604363	0.004787773
MIOX	4.130944312	0.004792062
GLT25D2	0.774533245	0.004800799
GLB1L3	0.329369728	0.004838027
NDUFAF1	0.962407052	0.004840746
G6PC2	0.585579759	0.004855814
CHST14	1.042635422	0.004859949
TPH2	0.160888193	0.004883341
CHRNA7	0.652508482	0.004898331
DHFR	1.04767102	0.004939265

CYB5R1	0.968346015	0.005098038
ACSBG2	1.882668088	0.00511503
SLC24A1	1.036459092	0.005264128
HSD3B7	1.050108936	0.005286391
CYB5R4	0.965583758	0.005337972
OXCT1	0.91743199	0.00543394
GGT1	0.924834247	0.005457232
DCT	0.451286053	0.005487985
CYP26A1	2.235490096	0.005512987
UGT2B10	0.607207949	0.005543988
SLC4A1AP	1.016392318	0.005581451
MTR	1.036123096	0.005600335
CYP24A1	2.280029709	0.005660897
NDUFA8	0.973558268	0.005668556
FADS3	1.078845562	0.005672377
TRPM7	0.974041077	0.005735958
BCKDHB	0.965854791	0.005792114
GALT	0.966885549	0.005831032
SOAT1	0.939643233	0.005954162
MICAL3	0.96398744	0.006024133
ARG1	0.55273238	0.006036614
PDHX	0.977022584	0.006123496
SULT1C3	2.712708665	0.006156837
PIGQ	1.023798471	0.006223254
DHRS7	0.962486891	0.006310128
KCNE3	1.071390018	0.006346175
PI4K2B	1.030879082	0.006399156
AQP5	2.39234575	0.006480632
SV2C	0.60107073	0.006603359
SDR42E1	0.896410743	0.006622213
SLC38A3	1.747735783	0.006629485
HACL1	1.033198589	0.006674255
ALG5	1.042968958	0.006747854
LPIN1	1.0516262	0.006768184
FA2H	0.955619235	0.00678155
ACSS1	1.054870366	0.006831814
SLC27A1	1.055199412	0.006877662
ACCN5	0.200192207	0.006884196
SC4MOL	1.050081622	0.006979263
KCTD15	0.935031087	0.007018653
CHKA	0.960360618	0.007077838
IMPAD1	1.023379079	0.007153631
ABHD12B	0.63907424	0.007223499
KCTD20	1.02401407	0.007224448

PTGR2	0.960272198	0.007256495
CACNG4	1.437670647	0.007297271
ABHD4	0.960496135	0.007323564
SLC33A1	1.023264968	0.007417379
MOCS2	0.962781719	0.007536553
SLC9A4	0.54619567	0.007551437
STARD7	1.011198086	0.007647893
ALDH3A1	0.888759077	0.007688011
SLC35F5	0.967093336	0.00769531
SLC25A37	1.041900419	0.007702006
SLC9A10	1.618939515	0.007736794
SLC19A3	0.870880804	0.007857105
C9orf98	1.215612498	0.007922051
FPGT	0.970189968	0.007935068
GPX4	1.031680346	0.007960053
CHDH	1.056686628	0.008229911
SLC16A5	0.934528504	0.008364075
HSDL1	1.031414131	0.008374519
B4GALT2	1.0277897	0.008413181
CYP3A43	3.887018023	0.008429448
B3GALTL	1.034498809	0.008444024
PLCH1	1.064094658	0.008470772
DIO1	0.589674892	0.008572537
SLC27A3	0.963180861	0.008614576
ME1	1.078725222	0.008639902
ANKH	1.037981809	0.008640926
DPM3	0.950852169	0.008665073
SRD5A2	2.317853653	0.008724273
B3GAT1	0.839382953	0.008734238
CEPT1	1.02148281	0.008745091
B4GALNT4	1.295465015	0.008762629
ATP5J2	1.033648529	0.008864076
FUT4	1.040065166	0.008869976
NUDT13	0.942890805	0.008932193
SLC35C1	0.973630415	0.009008308
ALDH1B1	1.032966566	0.009071339
MGST2	0.966755718	0.009106623
THNSL2	0.87908988	0.009253478
MCART6	0.873545541	0.009388437
CYP17A1	3.634360877	0.009390876
PGK2	#DIV/0!	0.009393524
PIP5K1A	1.018160625	0.009407376
XYLT1	0.955005682	0.009420768
KCNA10	3.604213255	0.009725144

ABCA2	1.044368036	0.009741579
ACCN4	2.092087155	0.009865623
ABAT	0.921711502	0.009875927
IDH1	0.971942588	0.009930525
ACOT6	0.536921017	0.010027359
KCNK5	0.939626971	0.010053546
SLCO1B1	8.569639911	0.010115954
ACAD9	1.01608463	0.010311893
FDXR	1.046756151	0.010370306
DGAT1	0.968119837	0.01039381
AGA	0.96858817	0.010403108
FADS1	1.106838269	0.010416193
NADSYN1	0.96769579	0.010418966
UGT2B11	0.337074796	0.0104306
PPOX	1.035229051	0.010528404
KCNC4	1.054265139	0.010610066
A4GALT	0.92269821	0.010624505
CA5A	#DIV/0!	0.010636183
ARSF	0.41176747	0.010776055
ST8SIA5	0.618552203	0.010824857
KCNH5	0.136641417	0.010946814
IPMK	0.955550286	0.010972222
ATP6V1A	0.983816221	0.010977867
SLC34A1	2.546930091	0.011089087
SLC7A8	1.052639225	0.011165372
PKD2	0.945486292	0.011188691
SLC24A3	0.930648542	0.011247402
MAGT1	1.027140939	0.011365571
NDUFA3	0.969353371	0.011467292
PRPSAP2	0.963368521	0.01159327
KCNJ2	0.923273737	0.011620608
ARSH	1.949811414	0.011628186
PLA2G16	1.093219887	0.01166233
MANEAL	1.066320555	0.011710075
SLC6A18	0.52768175	0.011718328
PPCDC	0.960648019	0.011733447
EPX	0.631353047	0.011734055
ADSL	1.03220114	0.012037535
SLC12A7	1.026777565	0.012233473
ADSS	1.01642132	0.012272528
TPCN1	0.974949425	0.012321336
CLCC1	1.018609808	0.012354559
MFSD3	1.048258232	0.012378489
NT5C	0.972450276	0.012405167

ABCF1	1.02154984	0.012491351
ATP10D	0.960480246	0.012776672
ATP10B	0.953891713	0.012924323
CKM	0.633467248	0.012931101
GSS	1.029124175	0.013032284
KCND2	0.760424269	0.013101888
HSD17B14	0.885651739	0.013355418
MOGAT3	0.90189632	0.013413385
UROC1	2.788055424	0.013486647
OXA1L	0.981065573	0.013543765
DGKH	1.081658166	0.013566179
SLCO1A2	2.897868236	0.013630911
NME7	1.04245074	0.013725959
CUBN	0.884168222	0.013837059
MOCOS	1.06123683	0.013885806
GNPTG	0.981220802	0.013890662
LTA4H	1.020641038	0.013959984
RENBP	0.918593932	0.013963188
MPST	0.975836878	0.014074692
HNMT	0.963626024	0.014333519
SLC22A2	0.552092606	0.014601914
COQ6	0.967531394	0.01467801
GUCY1B3	0.911258123	0.014708156
HIBADH	1.024267845	0.014751372
MFSD8	0.976215407	0.014973031
FOLH1	1.157551826	0.015009891
LIPJ	0.454261281	0.01527662
AGMAT	1.054471407	0.015386014
AGPS	0.982145704	0.015394296
GALNT9	0.660466752	0.015429893
ACE	0.961631798	0.015523905
TRPV6	0.640826683	0.015585649
PFKM	1.042918364	0.015604898
GGTLC2	0.901131688	0.015975646
PGM2L1	1.044201269	0.016019415
GPHN	1.032452895	0.016127549
NPC1	1.028019792	0.016538261
ATP5SL	0.979642251	0.016607828
CYB5RL	1.042704616	0.016682868
LYZL4	17.33241557	0.016716379
AMPD3	1.047988214	0.016822806
AGPAT3	0.974542251	0.01685592
PLD3	0.977975858	0.01691636
SLC25A21	0.666478879	0.017145967

ALDH1A3	1.062092743	0.017164882
SLC12A6	0.9697773	0.017171314
ATP5S	0.957805566	0.017231203
CS	0.985779926	0.017308087
CYC1	0.972783242	0.017370641
PLA2G15	1.030257068	0.017456222
SLC25A10	1.031712814	0.017572256
PNPLA2	0.968689629	0.017775403
L2HGDH	1.031112541	0.017871688
SLC2A3	1.063368609	0.017952542
KCNJ13	0.643814502	0.018229398
NMNAT2	0.840856661	0.018247104
UGT1A4	0.287382166	0.018293168
CYP2A6	0.461194624	0.018468253
NT5C3L	1.056966273	0.018580451
SLC6A14	1.338169874	0.018591594
PIK3R6	0.881468911	0.018691414
UCP3	0.879739209	0.018832779
AQP6	1.463982622	0.018868104
NDUFA9	0.977228132	0.018936378
CYP4A11	2.702325754	0.019353563
GABRP	1.294689088	0.01937719
MDH1B	0.806588672	0.019545973
ASRGL1	0.922601156	0.019594032
GMPR	1.095964828	0.01964231
NDUFS6	0.982593899	0.019869618
GTDC1	0.973011636	0.019922116
MOSC1	0.955294968	0.020015786
PHGDH	1.084610456	0.020106024
ABCB6	1.052493181	0.020269366
AGPAT4	0.925182683	0.020348258
POFUT2	1.026648522	0.020585673
SLC25A5	0.979897186	0.02064706
ATP6V0A1	0.973780188	0.020681479
GNMT	1.557146955	0.020910231
CLC	0.750931442	0.020976588
HSD17B13	0.82408633	0.021018944
CYP4Z1	4.094372816	0.021022649
NDUFC2	0.978453424	0.021168326
CACNG8	5.623453318	0.02120076
COX6A2	0.252647959	0.021428432
FUT2	0.962126455	0.021554668
ALG2	1.020065235	0.021642721
GGPS1	1.017886609	0.021767891

OLAH	2.594686784	0.022123738
ALOX5AP	0.936297643	0.022225866
PCYT1A	0.981118977	0.022261462
PFKL	0.978773349	0.022302956
HS3ST4	1.938631935	0.022480822
DGKE	1.0351888	0.023007821
SLC4A7	1.033729949	0.023037407
BCMO1	0.8903957	0.023163758
B4GALT4	0.965945722	0.023202615
ATP1A1	0.973394211	0.0232208
CHRND	#DIV/0!	0.023424081
PLA1A	1.117273879	0.023439486
GLT8D2	0.935946049	0.023502415
TRPV1	0.942589664	0.023626837
ABCF3	1.016484888	0.02435779
ALDOC	1.066348355	0.024830816
PLA2G4C	0.917057256	0.025073338
ENPP5	1.062884961	0.025109838
NT5E	0.938509275	0.025163417
ABHD14A	0.95667888	0.02532219
GMPR2	0.985035724	0.025323991
KCNQ1	1.052008824	0.025328694
ALG9	1.016258263	0.025357124
CACNA1S	0.512922123	0.025446954
KYNU	1.118072798	0.025574805
GRIN3B	1.960304646	0.025587617
HMOX2	1.018910415	0.025655831
AOC2	1.208041414	0.025950419
SLC16A6	1.157769242	0.02607888
B4GALT6	1.045340517	0.026119982
FUT11	1.025765357	0.026699416
SLC39A4	1.050809954	0.026710078
LDHAL6A	0.712646166	0.026921123
NDUFAB1	0.977775931	0.027089607
ATP6V1H	1.014634674	0.027167349
SYNJ2	1.027957526	0.027534275
SARDH	0.891721907	0.02783276
ABCB7	1.02264021	0.028063162
SLC25A2	1.764611622	0.028125866
ACOX2	0.925818895	0.028244817
SLCO2B1	0.956989101	0.02849884
SLC6A11	0.38466773	0.028592411
IDH3B	0.977671139	0.02860322
COX11	0.974401935	0.028719474

SMPD2	1.030627523	0.029051162
TAP2	1.034526041	0.029169862
NMNAT3	1.045990973	0.029255712
SLC45A3	0.955700178	0.029474818
SLC5A2	1.77517979	0.029613687
KCNC3	1.10993129	0.029980187
GLYAT	0.435591646	0.030043926
NUDT15	1.024002647	0.030077578
PIGK	0.981591018	0.030179873
ST8SIA2	0.832792891	0.030665611
RDH13	1.022001581	0.030973453
NAT14	1.071365437	0.030981879
FUT3	0.952147117	0.03108028
FDX1L	1.032567438	0.031621772
ACAD10	0.969989282	0.031830176
SLC35E1	1.011250887	0.031923424
KCNE1L	1.417237017	0.032040088
OAT	0.972929807	0.032390335
HS3ST3A1	1.111097045	0.032656871
SLC35E3	0.975066296	0.033219149
ABCG5	1.394585218	0.033347241
PLCB4	1.067162385	0.033411335
AQP12A	0.686879127	0.03358744
ATP12A	0.607405204	0.034011943
CYP2C9	0.690151163	0.034138285
GLRB	0.830697102	0.03437066
DGUOK	1.0225559	0.034648545
PIGO	1.022560307	0.034788357
CYP2C8	0.740150243	0.035144909
ADA	1.073200638	0.035230039
ATP8B3	1.145646931	0.035612714
DDAH2	0.967923335	0.036530246
HMGCR	0.971090121	0.037414473
SLC13A5	1.926098494	0.037587817
RPE65	0.695058889	0.037902012
GLUL	0.981566183	0.037970782
NT5DC1	1.015016391	0.038137932
KCNK7	0.873390308	0.038185497
C6orf192	0.9676064	0.038242301
ARSB	0.971850006	0.038673723
SLC39A2	0.753249303	0.038709328
CLCN3	0.975793117	0.038761414
ELOVL1	0.986793766	0.039528742
EXTL3	1.033591407	0.039916156

ABCA3	0.903016404	0.039966208
ATP1B1	0.96764137	0.040289215
AGPAT5	1.025494987	0.040446926
ABCC9	0.876770304	0.040945527
SLC6A7	0.878230497	0.041854341
ALDH8A1	0.844417069	0.042066525
INPP5B	0.975856833	0.042326935
PGS1	1.023843076	0.042374256
SLC39A7	0.981607751	0.042495697
CA6	11.37327148	0.042581797
OCA2	1.456018577	0.042638762
ABHD14B	0.97938709	0.043098455
SLC25A26	1.020609379	0.043268006
GCLC	1.017056304	0.043268215
SLC22A12	6.022315436	0.043283272
ITPR3	1.03484379	0.043739748
LIPK	#DIV/0!	0.043874549
DGKQ	0.963366342	0.044178887
KMO	0.87505505	0.044406466
SLC2A8	1.038458853	0.044450043
SLC4A9	0.612481043	0.044522704
KCNK1	0.943721001	0.045113855
GCAT	1.041498598	0.045337012
AADAT	1.085339016	0.045546435
LYG1	1.223307544	0.045661622
ACCN1	0.803764687	0.045743368
ACAD11	1.033848086	0.046249254
SPHK2	0.97249307	0.046465893
GLT8D1	1.015766654	0.046649896
ABCA7	1.055404703	0.046803378
SLC39A3	1.016336504	0.046828991
SCNN1A	0.965734854	0.047336368
UPB1	0.768376496	0.047496979
ALDOB	1.159338225	0.048063273
PDE12	1.0141872	0.048861458
TPI1	1.012401392	0.049022057
FUT6	0.943912314	0.049706101
TRPC4	0.859720939	0.050891641
LYZL2	#DIV/0!	0.051339327
ATP5L2	0.698553192	0.051402444
NDUFB6	0.97663618	0.051452306
CHIA	0	0.051543543
NAGS	0.910373876	0.051894128
CYP2C19	0.6971867	0.052001628

SLC6A5	0.153576817	0.052184299
HAS1	0.758591621	0.052222314
MAT2A	1.018113582	0.052559991
GLB1	1.016342258	0.052658305
INPP5F	0.976916577	0.052951461
KCND1	0.934784418	0.053539264
DHRS3	0.977482311	0.053956339
SLC39A11	1.03161126	0.054064895
GALNT8	0.881650574	0.054691394
BHMT	0.630996071	0.055276488
ACYP2	0.958402177	0.055303213
HPDL	1.09407526	0.055307044
MAN1B1	1.01564533	0.055922528
SLC26A1	1.069125876	0.056151888
PIGT	1.01672198	0.05626562
ABCG1	0.965228929	0.057225122
NDUFS5	0.982726458	0.057630695
GLT1D1	0.85849493	0.058097028
SLC2A7	0.464007372	0.058182936
SLC24A5	0.749634317	0.058774539
PNPLA1	1.326581093	0.059361643
IDS	0.980346431	0.059523756
PYGL	0.956720601	0.059674921
PIGY	0.980663565	0.059825293
SLC37A1	0.963251867	0.060274935
SLC12A3	0.686269667	0.060481768
ATP9A	1.036805926	0.060617289
PLA2G4D	1.265654968	0.061780684
SUCLA2	0.981302426	0.062504882
SLC16A11	0.85764345	0.062548501
NAT8	0.655230145	0.063780741
LYPLAL1	1.033404192	0.063970752
SLC16A1	0.96297974	0.064978885
ATP1A4	0.626053203	0.065364979
MANEA	1.026033442	0.06601661
NAGPA	0.978507754	0.066048764
CKMT2	1.275630175	0.066245319
DPYSL2	1.033115475	0.06687466
SLC35F4	0.537770349	0.067397641
GLRX	0.967262753	0.067521064
UGT2B4	3.049718435	0.067834004
NNMT	1.053898689	0.068289679
GSTK1	0.985226149	0.068325344
ACOT8	0.971527718	0.068608089

BPGM	0.976441275	0.069127739
CYB5R2	1.061887498	0.070156301
HK1	0.981536945	0.071446155
P2RX5	0.931486969	0.071872323
PIP4K2B	1.015032883	0.072197702
FUCA2	1.012319742	0.072349086
LYZ	1.056516857	0.073258737
PLCH2	1.112430996	0.073309795
ATP11B	0.985609417	0.074074564
SLC1A6	0.274054887	0.07415954
FAAH	0.971666354	0.074256679
DPYS	0.592473531	0.074887539
SLC38A1	1.017985224	0.074931513
ALG6	1.020371948	0.074973767
SLC22A13	1.464808594	0.075076427
DDC	0.940424635	0.075605619
MMACHC	1.026174905	0.075718967
HS3ST3B1	0.90775764	0.075739115
HEXA	0.98058855	0.076187072
SLC23A2	0.969390902	0.076261208
SLC17A3	0.452128832	0.076314486
COX8C	0.407994587	0.076338844
SLC44A3	0.970715612	0.076473982
HCN3	1.047815217	0.076694572
PKD2L1	0.762988186	0.077181094
ATP4A	0.30264801	0.077495869
KCNH2	0.92600919	0.078627421
SLC45A4	1.02664413	0.079128
ETNK2	0.900167451	0.079137
MCART1	1.019324537	0.07921716
CDADC1	1.01874237	0.079798796
DHRSX	1.022541078	0.07984098
SULF2	1.033432716	0.081470228
GCNT1	1.031847414	0.081500306
GALNT5	1.047253947	0.081823636
SLC36A3	0.264397906	0.083372502
CYP4A22	#DIV/0!	0.083448462
GUCY1A2	1.234861055	0.084530891
CLCN6	0.96749185	0.085013073
ENTPD2	1.045482041	0.085169911
POMT2	1.021826525	0.085593381
NT5DC3	0.936863619	0.085603086
NAALADL2	0.93472931	0.085714489
HSD17B4	0.983447856	0.085922996

TRPV5	0.446567203	0.087775055
PLCB1	1.069236322	0.087946747
KCTD16	1.357916388	0.088762887
ITPKC	1.015881432	0.089504662
FTCD	0.608543223	0.090891125
GRXCR2	0.335110288	0.091176035
MOGAT1	2.636704292	0.092427123
CACNA1I	0.786408739	0.092998756
ACAT2	0.970572329	0.093114141
PDHA2	3.285887656	0.093803902
CACNA2D4	0.941613173	0.094770159
AASDH	1.018777285	0.095315685
ENTPD7	1.024522304	0.095729366
AADACL4	0.452646579	0.096162408
MANBAL	1.019282153	0.096560821
CERKL	0.943937189	0.096996977
CACNA1C	0.917386202	0.097600369
GYG1	0.965095663	0.098078268
MMAB	1.016826716	0.098110535
CBS	0.903055305	0.098226549
CYP2E1	1.169587878	0.09937974
SCN10A	0.349104525	0.099893543
TMEM104	1.017633397	0.100139294
B3GAT2	0.884132543	0.101233849
CYP2B6	1.107027146	0.103445503
B4GALT1	0.979603245	0.103844452
CACNA1G	0.832557491	0.104107075
PGAM4	0.976133542	0.104231267
KCNQ3	1.269520549	0.104963326
GALNT3	1.02383962	0.105125313
BLVRA	0.953363272	0.106614909
SLC1A3	1.098700231	0.106936346
PLA2G1B	0.636617196	0.107013141
SOD1	0.984485426	0.107278072
SOD3	0.960548396	0.107719043
ETNK1	0.97094508	0.109479254
GPX8	1.045939971	0.110216276
MANBA	1.017627464	0.110374116
PIP5KL1	1.215930431	0.110570056
CABC1	1.016407213	0.110609821
PLB1	0.930707984	0.111633017
LGSN	0.700673362	0.112056285
SLC15A3	0.969176248	0.112711909
GNS	1.010264535	0.113977776

KCTD5	0.984660006	0.114155508
PIK3CB	0.987497514	0.114221325
MGAT3	0.935144222	0.114929949
GUCY2D	1.848469095	0.114946083
CACNG1	2.755557936	0.11557832
GSTO2	1.0527748	0.115900408
ADI1	0.984891151	0.115957666
ALG10B	1.028257711	0.11647529
LCAT	0.947090318	0.116747601
KCNK13	0.899301978	0.117005037
ATP6AP2	1.013673942	0.11727595
SLC12A5	0.841066368	0.117551053
FUT10	1.030539749	0.117704375
PAM	0.977945589	0.117999911
HYAL4	0.655647797	0.118165437
COQ3	0.975934104	0.118258311
INPPL1	1.011705762	0.118361298
CCBL2	0.988396042	0.118438614
GALNT14	1.196550036	0.118967537
SLC25A6	1.013909127	0.119810482
ATP5G2	0.988034272	0.119819726
TRPM8	1.474386881	0.12055424
GMDS	1.023456485	0.120628736
DEGS1	1.021363336	0.121604077
IDH3G	1.013995988	0.122378567
ADCY7	0.971582011	0.122513501
STARD3	1.014394209	0.122574485
TRPV2	0.964650295	0.122849653
SAT1	1.019247851	0.12317998
HYAL1	0.918871026	0.123432948
CES7	0.43719067	0.123746148
OGFOD2	1.012436788	0.126096789
SLC30A2	1.234761804	0.126887733
EXT2	1.009456046	0.127923713
KCNH6	0.812164041	0.127944647
SLC25A38	0.986890785	0.129798953
GABRG3	0.295283417	0.130418528
GBGT1	0.952369271	0.132804871
ACY1	0.981191694	0.132971934
GFPT2	0.922381447	0.133743884
CYP11B1	0.176199918	0.133985472
COQ2	0.982406434	0.134720544
HEXDC	1.027339144	0.134950958
KCTD19	1.352108162	0.135107622

PHOSPHO1	0.911560519	0.135273174
AASS	0.9546255	0.13620917
SLC30A5	1.010081338	0.136288971
PDE6C	0.540380464	0.136322489
KCNA7	3.084602133	0.137147041
SLC37A4	0.98437852	0.137970585
ITPR2	1.032045543	0.13971413
BPHL	1.020075689	0.139848723
IDH2	1.021879337	0.139893476
B3GNT9	1.026376129	0.140407844
MVK	1.016337371	0.140582222
GUCY2C	0.962493677	0.14098849
CNGA4	0.847836752	0.141507717
NME3	0.978684607	0.142433162
PIGF	1.018119617	0.142531158
AQP3	1.071453228	0.144464216
VDAC3	0.984197922	0.144466047
KCNQ2	0.697544821	0.144631948
MPO	1.201806658	0.14480299
MBOAT7	0.985361051	0.146083964
PIP4K2C	0.988967215	0.146293227
GGT7	0.95443631	0.14766928
DHRS12	0.976244666	0.148111657
PTS	1.021326681	0.14865571
MCART2	1.088299915	0.149536867
SLC25A28	0.988910927	0.149744748
FDFT1	1.016040251	0.15070581
AGXT2	0.50869046	0.151353441
ALDOA	0.99115415	0.152487517
DCXR	0.980294167	0.153073832
PRDX2	1.012305378	0.153653816
GOT2	1.009412581	0.153901672
SLC30A3	1.313513822	0.154610765
SLN	1.389224533	0.15464356
PCBD2	1.012280472	0.155142608
CYP1A2	2.378688921	0.155826537
NOX3	0.215867309	0.155917071
DDHD2	0.984056823	0.156041087
PIK3C2A	1.010616898	0.156144854
CATSPER2	1.056215205	0.157576517
SLC28A1	0.678676036	0.158164831
NDUFB11	1.013351652	0.160818237
SLC7A13	0	0.160919774
HAO1	#DIV/0!	0.16128825

DBH	0.862317186	0.161613057
CA8	0.896184641	0.161774704
LALBA	#DIV/0!	0.162625963
PLCB2	0.961504208	0.163584209
LIPM	0	0.163761736
ARSD	0.979789793	0.165884521
PNPLA5	0	0.166176857
MAN2C1	0.985778898	0.166817852
GGTLC1	0.850535678	0.167284891
COX17	0.985872126	0.167545795
ALDH7A1	0.975231575	0.168825321
PCCA	0.965986944	0.16946138
PANK1	0.97713425	0.17025781
CYB5D1	0.979733838	0.172844696
SLC7A7	1.036594541	0.173380571
CHRNA9	1.737967319	0.173547574
AMY2A	0.701103131	0.173900685
SCN2A	0.846316285	0.174029208
PIK3R4	1.013171615	0.176020579
SLC6A4	1.250172065	0.176170818
ENPP7	1.579795871	0.176206303
CFTR	0.946572274	0.176828016
MICAL1	1.020650783	0.178208104
ABCC4	0.971609462	0.179444609
ASAH2	0.881394403	0.18039677
NUDT2	0.973048836	0.180719599
GRIN1	0.91076847	0.181451055
KCNN2	0.876555831	0.181555203
LPPR4	1.074064946	0.182261758
SPR	0.986445288	0.183782211
PGAM1	0.991482819	0.184279388
CSAD	1.036563526	0.187096096
AMDHD1	0.869888422	0.187748748
SLCO3A1	1.040645282	0.190910503
B4GALT5	1.012692334	0.19263231
PPAPDC2	0.983539318	0.194029005
SLC2A10	0.969032903	0.196645547
SLC4A3	0.902395848	0.196654463
SLC14A1	0.900149493	0.198156289
CPT1B	1.039826581	0.198448329
FDPS	1.016411391	0.198703757
UGT1A6	1.072991704	0.199666139
NOX1	1.033837927	0.199853201
PRODH2	#DIV/0!	0.199931329

FBP2	1.343902617	0.202811081
AMD1	1.010810712	0.203212237
EDEM1	0.987195846	0.204578017
SPTLC2	0.985252777	0.205313167
HCCS	0.985927488	0.206772968
SLC7A9	1.30617847	0.207044993
ABCA12	1.403094597	0.209690815
STARD3NL	1.015610959	0.210200709
B3GNT3	0.98489023	0.212060831
MTRR	1.008510281	0.212062793
HSD3B1	0.81462683	0.212926482
SLC39A9	0.992667327	0.213626624
CHIT1	1.183810331	0.213930225
SLC16A3	1.016598116	0.216607726
ADH7	0.095592225	0.218339573
HSD11B1L	0.964359138	0.219934048
ABHD13	1.012522601	0.219989178
CA5B	1.026339466	0.220121314
ACE2	0.941669352	0.220959704
HTR3D	0.228496232	0.223873037
VKORC1	1.013024576	0.224496602
KCNG2	0.843969348	0.225257214
GSTO1	1.019224908	0.225274716
NEU2	0.43820482	0.225733864
GLRX2	1.015174319	0.227229442
INPP1	0.981194018	0.228016216
SLC2A2	1.710988501	0.228631374
HAS2	0.96373153	0.229409365
PANK2	1.011584158	0.232181678
LIPE	1.035686924	0.232262248
ABCC11	1.250635557	0.234100493
ACCN3	1.137471357	0.235020072
ACSL3	1.009764805	0.235812583
NDUFA7	1.013546156	0.237080896
SDS	1.07239105	0.238341989
PGLS	1.010685132	0.240480295
ACOT7	1.011640753	0.240835041
AMACR	0.977762232	0.2408431
ATP7A	1.015907012	0.242997374
SLC25A43	1.011783164	0.243184086
SGMS1	0.980354242	0.243921416
SDR39U1	1.012934061	0.244979449
HEXB	1.008271203	0.24531977
PIGC	1.01216016	0.246141387

ACMSD	#DIV/0!	0.247754762
ORAI3	1.0141216	0.248838583
GUK1	1.013316311	0.250666558
FUK	1.012556034	0.250674311
CYP7B1	0.9388294	0.251149223
TYMP	1.025221109	0.252432883
OXCT2	0.929359346	0.252722248
ELOVL7	1.024365008	0.252902022
ATP2A2	1.010006652	0.252905864
ACN9	1.025096425	0.253954502
TMCO3	1.015649799	0.255081791
SLC35F2	0.984029214	0.255221643
ACOT12	0.438524278	0.256580039
SLC26A8	0.895662526	0.257499296
NDUFA4	1.013714777	0.258316864
COX7A2L	0.984377252	0.258614907
ATP6V0E1	0.990419371	0.259463065
LIPF	2.361580121	0.259600578
ACBD3	1.007837845	0.264348137
TRPM1	2.168368476	0.265815947
PLCXD2	1.017872008	0.266040344
SLC25A33	1.017496915	0.267050125
GYS2	0.773850364	0.267730325
CYP2D6	0.925874213	0.26875098
ISYNA1	1.03344939	0.27197742
ABCB9	1.023946696	0.272245102
DAGLA	1.020513772	0.272488226
CACNB4	0.930384373	0.273067604
CYP2F1	1.400107478	0.27493055
CYBA	0.989048054	0.27607828
IDO2	0.871280905	0.27925281
SGPL1	0.990173418	0.279711436
SLC22A7	2.075241224	0.279748826
KCNJ10	0.951997852	0.281697742
B3GNT2	0.990407634	0.281791279
SLC2A14	1.064577578	0.284388289
LNPEP	0.985324096	0.285195917
LDHB	1.024058005	0.285531137
PGAP1	0.977794246	0.287281044
XYLT2	1.011373227	0.288023145
KCTD6	1.019695143	0.289036737
PDE4A	1.014057434	0.28928946
PKLR	0.87729364	0.291548202
ACSM4	0.387529064	0.29380025

SPTLC1	1.004855688	0.294889133
SLC10A7	0.986993318	0.295453122
GSTT2	0.942075953	0.296337143
CYP26C1	1.647195453	0.296680827
PLA2G2F	1.286367103	0.296977753
PIK3CA	0.988386317	0.297249347
CHST12	0.987922997	0.297347161
SLC13A4	0.904500065	0.297403129
ABCA13	1.112900356	0.298528235
LIPA	0.983441212	0.29881288
GGT5	1.029332725	0.2992451
D2HGDH	1.019101807	0.299988047
KCNH3	0.90919183	0.302564605
HCN2	0.910380568	0.302803286
ASS1	0.980885024	0.303502772
PPAPDC1B	1.015947692	0.304803537
ASNA1	1.009386348	0.30729606
GALK2	1.010415639	0.308142048
FBP1	0.97824492	0.308571227
UCP2	0.975925358	0.308651085
DHTKD1	0.990532902	0.308808943
SLC7A10	0.741213575	0.312590372
HK3	1.058961651	0.313104707
UCP1	2.757604765	0.313459126
SLC26A10	0.886877228	0.313469552
PI4K2A	1.008189838	0.314017354
GSTA3	0.144295856	0.314402764
HSD11B1	0.938889964	0.314494053
CHRNB1	0.971076725	0.314749006
ALDH18A1	1.011705254	0.316593817
CRYZ	1.024410563	0.319911837
ATP2C2	0.983901175	0.321178552
SLCO6A1	#DIV/0!	0.325052733
SLC17A2	#DIV/0!	0.325052733
ADAD1	#DIV/0!	0.325052733
PNLIP	0	0.325052733
SLC22A24	0	0.325052733
KCNK18	0	0.325052733
ATP6V1G3	0	0.325052733
CYP2A13	#DIV/0!	0.325052733
GOT1L1	0	0.325052733
GPX5	0	0.325052733
GPX6	#DIV/0!	0.325052733
LYZL1	#DIV/0!	0.325052733

CYP11B2	#DIV/0!	0.325052733
PKD1	0.984112087	0.325070131
AK2	0.992262706	0.325094347
PLA2G12B	0.892841431	0.325159965
AGPAT2	0.98810913	0.325649269
KCNJ3	0.905238551	0.326123615
TXNRD2	1.007563567	0.326655991
PFKP	1.01267833	0.329630342
PDHA1	0.98952126	0.329642471
SLC6A13	1.427164522	0.331307608
DHRS13	1.018286975	0.332856091
HS6ST1	0.987323521	0.333632853
GLYATL2	0.827601647	0.333771292
NALCN	0.905972228	0.337945689
CNP	1.006421495	0.338849691
A4GNT	1.385020165	0.339525977
CYP2R1	0.98781027	0.340064697
SLC43A2	0.988401029	0.345310261
ST6GALNAC5	1.055563926	0.345523904
PDXK	1.0113976	0.345636439
RDH8	1.492120986	0.346276072
PRDX5	0.985689338	0.346342618
PNPLA6	0.989872628	0.346478481
BCAT1	0.96614235	0.347138882
ACBD5	1.010548148	0.347153854
GABRQ	1.272057744	0.347502984
BBOX1	0.72510538	0.349237671
PRDX3	0.990381452	0.349432949
GABRB1	1.229080696	0.350151027
SLC22A14	1.329195922	0.35545265
IDI1	0.986511347	0.356267284
DPYSL4	0.93047711	0.357394036
LSS	0.986265443	0.358427878
SLC25A40	1.009243868	0.358713219
LYZL6	4.038618717	0.358746668
CNDP1	1.346392622	0.358982885
IP6K2	0.991300935	0.362955307
NAPRT1	0.987885931	0.363298316
ALOX12B	0.896222317	0.363595075
NPL	0.973846026	0.364913033
IDO1	1.050118346	0.367001136
ADCY1	0.960616103	0.36813734
ELOVL3	1.14861008	0.368875996
DUOX1	1.059228844	0.369355402

MVD	1.013448635	0.370107034
SCCPDH	0.990168153	0.370402771
LIPN	1.300433239	0.3738147
RYR2	0.93442596	0.373905923
ADCY10	0.84302518	0.378425313
GLB1L2	1.012180683	0.380844168
SLC25A45	0.980798455	0.381439948
TRPM3	0.807250264	0.386886104
ENO2	1.026714105	0.387008853
HAS3	1.045829525	0.388699788
CACNA1E	1.115368169	0.390051365
GRHPR	0.992257687	0.392754464
KCNU1	1.931852456	0.392975777
OAS1	0.985249149	0.394830388
KCNK4	1.206357985	0.396184611
CPT1C	0.948573565	0.396584754
FAH	0.988125823	0.401204251
HMGCS1	1.014109307	0.402553663
ATP6V0E2	0.98347801	0.403763137
C14orf68	1.106931438	0.407807699
ALDH5A1	1.012718033	0.410155938
GABRR1	1.206072701	0.410898534
ALDH3B1	1.013958913	0.412009043
PLCB3	1.011706785	0.416976637
PIPOX	1.07310895	0.423897946
SC5DL	1.010931371	0.426802498
NOS2	0.959935829	0.427107518
PRG2	1.09499057	0.427778558
AMY2B	0.969502986	0.427907924
SLC46A2	0.926061148	0.430561051
ATP4B	0.762885957	0.431104462
SLC16A8	1.133085742	0.432734565
HCN1	1.163413079	0.434836218
CNGB3	1.296089611	0.436669511
SFXN2	1.014635877	0.438448613
FOLR3	0.862817188	0.440130631
CYP3A5	0.982661295	0.442149544
GALE	0.989668543	0.443581665
GALNT10	0.989979013	0.443861072
AK3L1	0.976212529	0.443989883
GABRR2	1.114476683	0.445072356
ABHD10	1.009216521	0.450902683
FN3KRP	0.994207392	0.452395828
AQP10	1.302145791	0.452596713

SLC7A4	0.953675016	0.452906239
UGT8	0.990095756	0.453132082
SLC5A5	0.8218144	0.45385664
NME4	1.015043281	0.45509616
NT5C1A	0.74109085	0.4575493
CATSPER3	1.041790983	0.457613638
DGKD	0.989682037	0.458680726
UGT2B28	2.181076208	0.458977036
DIO3	1.054511462	0.467344926
GCDH	1.00611743	0.468380868
GRIN2C	0.938659674	0.469959625
KCTD11	0.992659291	0.472624466
SLC16A10	1.041957622	0.473145413
UGT1A3	1.225741324	0.474451614
SLC25A31	1.651788609	0.474675399
LIPT1	0.988964652	0.474720944
AGXT2L1	0.679291204	0.475215698
TK2	0.99281714	0.477302969
PON2	1.00861827	0.483063484
ATP5E	1.006794294	0.484672224
SLC9A8	1.0107987	0.485211129
GBE1	0.993166463	0.486398481
DHCR24	0.990468358	0.491065905
CDIPT	0.995249191	0.491545517
STARD6	0.517515989	0.493561819
SDR16C5	1.05146688	0.493653198
NT5C3	0.993351405	0.496990917
ALG13	1.007912908	0.4977878
TRPC3	0.938795114	0.5001215
CHST13	0.955169697	0.501465271
KCTD7	0.987694766	0.501589267
HSD17B1	0.981205937	0.501977429
GLB1L	1.014010678	0.502981703
GALNT11	1.011525866	0.505633255
KCTD17	0.989119722	0.506251014
ASMTL	0.991390937	0.50918688
OXSM	0.992020829	0.509290941
CACNB3	0.993533835	0.509859264
SEPX1	0.991411342	0.511044682
SULT1E1	1.118997046	0.512278186
PRODH	1.053427093	0.518618776
PLCXD1	0.989401516	0.520482773
HAO2	1.26498174	0.521693573
SOAT2	1.163520188	0.522042152

TYMS	1.013882727	0.522073289
ATP6V1B2	0.993743594	0.523396087
ATP1B4	0.444565133	0.523978913
MTHFS	1.009734982	0.52629949
CPS1	1.073045578	0.531209619
FMO1	1.083553265	0.531734337
GPD1	0.963130286	0.532823772
DGKI	1.071608896	0.536106185
GAD2	2.052805439	0.536391759
SGSH	0.99409529	0.537196507
DUT	0.993170835	0.539611019
ABCD1	0.990739617	0.541945076
SLC2A6	0.981617045	0.545559269
GCH1	0.991497457	0.547070687
UPRT	1.007045997	0.548506039
SLC38A6	1.014515965	0.550404968
SATL1	1.011097824	0.551277762
CA3	0.954083356	0.552442419
ARSJ	0.977183269	0.552574179
ADCY4	0.982918064	0.552584311
CHRNE	1.039450698	0.55706932
NME6	1.005445001	0.560246132
SLC2A11	1.011202558	0.562212243
GDPD1	0.974756252	0.564104957
KCNE4	1.022890212	0.564271628
SLC43A3	1.019222803	0.56688441
SOD2	1.005131049	0.568416902
AMDHD2	1.009262148	0.569102386
MTTP	1.103954248	0.569254009
LCT	1.14721981	0.570606378
PYGB	0.990489801	0.571993054
SLC26A11	0.990446703	0.573850794
CATSPERB	1.047818667	0.574094813
NUDT8	0.988707899	0.577271589
KCNJ1	0.852130599	0.580009877
GABRA3	0.873943359	0.580199969
ACER1	0.808929285	0.581289735
KCNJ4	0.911067328	0.581415552
ABCB10	1.004450767	0.583143767
SCN8A	1.032537322	0.586437584
AKR1D1	0.777684616	0.587268224
TYR	1.444353584	0.587493157
COX16	0.994563753	0.58938034
SLC36A4	1.017858186	0.591425589

IDUA	1.01474519	0.595202975
NDUFA11	0.993200096	0.596093355
PLA2G7	1.02314063	0.60083687
LPL	1.031107363	0.602840523
COX4I2	0.960873456	0.60353147
PRPSAP1	0.996138531	0.604023469
PIK3R3	1.00833748	0.605224208
CYP3A7	1.048884792	0.608734836
COX18	1.00675216	0.609674785
HSD17B3	1.100043657	0.609848544
ST6GAL1	0.986746939	0.610971834
MMADHC	0.9962878	0.614997686
MCOLN3	0.963484511	0.615255513
AKR1A1	0.995684136	0.615583341
KDSR	0.995894565	0.618686638
ARSI	1.028749082	0.619834818
GMPPA	1.004467054	0.622840035
H6PD	1.005683983	0.623929114
PIGH	1.005446698	0.624297223
SLC26A6	0.991904825	0.62448841
GABRA5	0.800255029	0.624507299
CYP4F2	0.961185967	0.628120455
LYPLA2	0.995913874	0.62956696
GUSB	0.995991822	0.630798279
UROS	1.005487183	0.632585116
CACNA1F	1.056508376	0.633853221
NDST2	0.995980265	0.640054977
SLC25A25	1.006931223	0.640085682
B3GALNT1	0.980525578	0.64166931
SLC5A9	0.965948281	0.641982537
GALNS	0.993484005	0.642724019
CBR3	0.98434093	0.643609558
SLC22A16	0.915310773	0.643776339
GDA	0.986836612	0.644577903
SLC5A4	1.0884846	0.645266895
SLC2A9	1.014350857	0.649256845
GUCY2F	0.483745984	0.649460598
NT5C2	0.996487159	0.650475395
PLD2	1.005190228	0.652675418
ABCC5	0.993203383	0.656610097
GFPT1	0.995787865	0.657634714
DDAH1	0.996413032	0.661352846
GLRA1	1.242481429	0.66145958
IAH1	0.994606398	0.661484316

GALNTL6	0.918669837	0.663041153
DGKK	0.782121422	0.663077014
TKTL2	0.902224882	0.663573524
ASMT	0.908215852	0.666093579
SLC9A6	0.995973666	0.670350602
FHIT	1.015366601	0.674311956
CHST3	0.987339297	0.67629325
DGKG	1.029021748	0.681065333
CHRNA10	1.020859217	0.686661957
AADACL3	0.683660966	0.686735758
MMEL1	1.030824001	0.688570629
PFKFB1	0.93684253	0.689948088
MIP	0.947472342	0.694754035
PON3	0.970835307	0.696671538
CYP2A7	0.857999737	0.69699756
ENTPD1	1.004987503	0.69721705
GADL1	1.135491516	0.69802889
GCNT7	1.101078387	0.698356188
QPRT	0.985294433	0.706074714
NDOR1	0.99631059	0.713846956
SLC22A9	0.866013779	0.714608918
KCNK16	0.761547062	0.714746157
FAHD2A	0.996679986	0.716674556
ATP6V0B	0.995563342	0.719509712
MBOAT2	0.992512725	0.720430435
ADCY8	0.837843801	0.72282529
PIGP	0.995300114	0.723311812
DGAT2L6	1.221826481	0.725303286
KCNJ8	0.981506636	0.727285681
ME3	0.9939074	0.729949264
BSND	0.851400043	0.73005944
UGT3A1	1.306727245	0.733434562
PNPLA8	0.996963518	0.733826367
SLC34A2	1.119220009	0.73508295
RRM2B	0.996185551	0.740177272
ALDH16A1	1.003388256	0.745103691
GDPD4	1.083568486	0.745736769
PNPLA4	0.99447225	0.746813654
PNMT	0.958931035	0.748597516
FUT5	1.058398698	0.749695323
TRPC5	0.880394095	0.75093038
ABCG8	0.946753872	0.754181335
ARSG	1.009915723	0.754349745
TXNRD1	1.00236489	0.754611806

CHST11	1.011122832	0.755710514
GABRA6	1.543800974	0.75579953
MTHFD2L	1.005575423	0.760867787
PDE6D	0.997334296	0.764970006
ALOX15B	0.975319451	0.765450692
PDE4B	0.992027015	0.768840507
ELOVL2	1.035711665	0.771474365
SLC12A1	0.906635675	0.772067401
AKR7L	0.991892365	0.772425894
UGT2A1	1.220894429	0.772845086
SLC9A11	0.834421772	0.772971216
INPP4B	0.993778019	0.775829484
APEH	0.998066943	0.776944687
ACSL1	1.005556258	0.779483125
LASS3	1.114939553	0.783190076
COMTD1	0.994605257	0.784158976
PLA2G6	1.004552085	0.787093411
ACER2	0.990526978	0.787417436
MINPP1	1.003471342	0.788920754
NAGLU	0.997768003	0.789264496
PON1	1.09014083	0.789598313
CDS2	1.002805314	0.794403977
CYP1A1	0.936028	0.80149617
KCNAB3	1.020537407	0.802350149
SCNN1D	0.98390433	0.803978789
SDR9C7	0.809240269	0.80548427
AANAT	1.072849206	0.805742873
KCNH7	0.951169368	0.809978407
SULT2A1	0.945206059	0.81141538
NDST3	0.976960085	0.812553262
SLC5A3	0.997069462	0.813040246
ALOX15	0.976145892	0.813622604
DAD1	1.002069903	0.81989753
COQ10A	1.005674121	0.820563838
GALNTL4	1.007062026	0.825307126
SLC45A2	0.97419161	0.831550452
KCNMB4	0.98843885	0.831846333
KCNK17	1.016271739	0.833981197
ARSK	0.996339826	0.836004791
HAL	1.019888038	0.839599836
GK	0.995946276	0.844838107
GLRA3	0.930860534	0.846773066
MAN1A2	1.001486671	0.851930949
CA13	1.006102119	0.852298785

SLC39A14	0.998086803	0.852321044
PIK3C2B	0.997888759	0.855303516
ESD	1.001522026	0.857408521
ACY3	1.005186613	0.863085324
STARD4	1.003995532	0.864293036
HS3ST2	1.016686131	0.869079073
ZACN	0.943473706	0.869867558
ALOX5	1.004738854	0.870814658
FOXRED1	0.998467355	0.872798779
KCTD3	0.998652276	0.873749909
PHOSPHO2	0.997179301	0.878208159
DCK	0.99794729	0.885882446
SLC35E2	0.996140291	0.89148619
TMLHE	1.002038999	0.892661469
ACSL5	1.001907229	0.893779065
GNPAT	1.000992724	0.897280292
COX7B2	1.202013845	0.899606339
ATP6V0A2	1.001077566	0.900424987
ATP6V0C	0.999112252	0.900530434
TCIRG1	1.001377343	0.900871348
CACNG3	0.88830241	0.902488722
HGSNAT	0.998647003	0.903090471
KCNT1	1.027153116	0.904410823
AMY1A	0.975925638	0.905175831
GPX7	0.996390244	0.905742787
GLYCTK	0.997696115	0.912213274
SLC25A13	0.998426341	0.915269993
PLCZ1	1.115327901	0.917205313
DEGS2	0.997349772	0.91890674
P2RX3	1.048138826	0.918997014
KCNK15	1.013049706	0.919206243
ABCA4	0.985720909	0.921695643
PDE6H	0.908863692	0.922437994
PTER	1.000814246	0.923881308
AOAH	1.003625941	0.923979932
SLC12A4	1.001596317	0.92629631
BCKDHA	1.000817464	0.929201477
UPP2	0.969439525	0.931157356
LCTL	1.012849061	0.932430653
SLC1A7	0.992989787	0.936231281
DSE	0.998636753	0.938552244
GYG2	1.003123508	0.93875587
SLC36A2	0.953422005	0.944688334
AACS	1.000638381	0.945608036

ATP10A	1.002485126	0.947110898
GABRR3	0.921241586	0.947207014
FAHD2B	0.997667288	0.947324465
SLC22A8	0.910070835	0.948222709
LPPR2	1.001590125	0.949020344
KCNS1	0.987770951	0.949043463
PTGS2	1.002984693	0.954146872
SLC31A1	1.000496358	0.956590556
PDSS1	1.000735062	0.957964702
SLC10A6	1.006101112	0.959311475
SLC22A1	0.993677482	0.962778958
CH25H	0.99813017	0.969068817
ALDH1L2	1.001517844	0.971179757
DECR2	0.999600945	0.972108647
PCYT2	1.00048696	0.973052694
NT5C1B	1.003626495	0.977237481
NUDT17	0.998634938	0.978786778
THEM5	1.005485315	0.981684975
DUOX2	0.998279754	0.982365311
QDPR	1.000248705	0.982751095
IYD	1.000551863	0.984137574
SLC44A5	0.998343709	0.985100021
LDHC	1.001461528	0.988295327
GPAM	1.000203199	0.990993322
EXTL2	0.999843178	0.993172831
SLC26A5	0.999153618	0.997913934

Paired analysis in TCGA CRC RNA-Seq		
Metabolism-related genes	Tumor/Normal	P Value
LYZL2	#DIV/0!	0.0175925
KLK8	502.6674486	0.0001055
CEL	127.2136394	0.0093752
SLCO1B3	91.42823437	0.0097002
STRA6	85.32553346	0.0001744
AKR1C4	53.35858317	0.0043614
CA9	41.49325031	0.0008199
GAD1	36.06381947	4.788E-08
GRIN2D	35.46182944	3.236E-15
PAH	32.27221839	5.616E-07
SLC35D3	31.44324866	0.0019867
EPHX4	23.231693	2.193E-11
LYZL4	22.88669558	0.00049
SLC13A3	22.7568002	1.835E-05
SLC22A11	22.12489096	6.517E-06
ACSL6	20.77255632	3.351E-07
CHST4	18.99328606	0.0016471
NPC1L1	18.6449143	0.0030951
LPO	18.08940513	3.195E-05
CYP2W1	17.41583048	0.002022
SLC22A12	16.96321413	0.0033231
ALDH3B2	16.94087273	0.0066123
SLC4A11	16.30755834	5.994E-08
PLA2G3	15.96135236	5.353E-05
UCP1	14.92740168	0.0002822
GRIN2B	14.22891293	0.0112854
HAGHL	13.23293534	8.982E-10
BAAT	12.87818462	0.0052732
CACNG8	12.77430697	2.359E-07
SLCO4A1	12.63239972	8.283E-15
HS6ST2	12.51467371	2.632E-08
PRPS1L1	12.17343425	0.0332009
PNPLA3	11.27161761	3.688E-05
HS3ST4	11.19780759	0.0259279
MAT1A	11.05808618	0.0036175
CHI3L1	10.76170558	0.0001203
ATP6V0A4	10.73827411	0.0093675
FOLR1	10.7118074	0.0054173
AQP2	10.00741068	0.0035415
DPEP1	9.821684108	0.0013696

NOX4	9.774864129	0.0005023
SULT2B1	9.767756993	1.787E-10
PGK2	9.200051849	0.0009401
CYP4X1	9.158426642	0.0019656
SLC7A5	8.84919257	1.023E-12
CACNG4	8.644880171	0.003747
SLC6A6	8.517573791	4.467E-11
GABRD	7.984998317	1.942E-09
CKMT2	7.975122204	0.000308
GLYATL1	7.871429972	0.0001442
TH	7.850992836	2.808E-06
SLC11A1	7.845918888	0.000159
RDH16	7.719138372	5.049E-06
FUT1	7.677777391	9.489E-13
ALOXE3	7.613662317	0.0009423
PSAT1	7.50286634	3.423E-11
GABRE	7.314308358	1.367E-06
CYP26A1	7.202443275	0.0067641
GDPD5	6.988157982	2.142E-08
CHRNA6	6.806640478	0.0016098
KCNA10	6.773210877	0.0012492
AQP9	6.758494875	0.0067485
RHBG	6.732469193	3.958E-08
GABRP	6.676152993	0.0013612
NDUFA4L2	6.670195926	0.0045709
CYP4Z1	6.535656693	0.0015368
B4GALNT4	6.459135076	0.0035592
ABCC2	6.411672939	0.0076735
KCNH8	6.385266518	7.022E-08
SLC7A11	6.346086039	2.46E-07
GLS2	6.253312855	1.11E-09
CYP19A1	5.979938496	0.0418381
KCNK9	5.897973921	2.188E-05
SLC38A3	5.70369866	0.0168102
SMOX	5.588813612	1.523E-14
ATP11A	5.572484349	3.498E-12
SLC28A3	5.491922629	1.237E-05
SULT1C2	5.461771839	0.0006345
NT5DC4	5.451740832	5.871E-06
RHCG	5.44119071	9.995E-05
MTHFD1L	5.417726639	1.176E-19
CYP4F8	5.236283608	0.0012557
SPHK1	5.117313473	2.77E-05
KCNJ15	5.110822031	0.0034174

KCNH4	5.060985554	0.0093486
EPHX3	5.044516961	0.0149088
SLC17A9	4.952478348	1.435E-10
DIO2	4.846126469	7.562E-07
ATP6V1C2	4.828701174	2.581E-12
KCNT1	4.816420546	0.0230399
UROC1	4.734003796	0.0002068
AWAT2	4.702145643	0.0482148
ACBD7	4.643432728	8.15E-08
SLC6A20	4.560055691	0.0001653
SLC38A5	4.476647966	2.729E-10
SCD	4.439541208	1.73E-12
KCNK16	4.393110551	0.0306748
SLC14A1	4.316334927	0.046908
SULF1	4.309670948	4.44E-06
KCTD16	4.259621194	0.0005824
SLCO5A1	4.256436523	0.0042353
CYP27B1	4.229935573	9.82E-14
ARSH	4.192176343	1.597E-05
GABRR1	4.185576558	0.0192779
SLC39A10	4.182730403	9.358E-11
SLC5A6	4.175294041	2.762E-11
TRPV4	4.169286988	2.742E-06
SLC22A3	4.109342734	3.134E-09
OCA2	4.085022369	0.0130031
KCNJ11	4.059148645	0.0006459
SLC27A5	4.049958136	5.266E-06
CNGB3	4.013344151	0.0229189
GALNT6	4.002081005	1.492E-10
PDE10A	3.997164357	0.0088202
CHRNB3	3.955608014	0.0275041
GADL1	3.942024059	0.0406042
CHPF	3.938596776	2.148E-14
ALDH4A1	3.913382446	3.355E-10
CNDP1	3.890621447	0.0305139
SLC6A14	3.881867144	0.0028388
KCTD19	3.877444096	0.0002026
PYCR1	3.826676376	9.735E-14
CHRNG	3.803628253	0.0016574
KCNJ14	3.754312203	2.672E-11
AHCY	3.728247124	5.892E-11
SLC6A3	3.714123288	1.042E-05
DGAT2	3.707174435	1.551E-11
HCN1	3.703728925	0.0001663

SLC24A2	3.700651917	0.0025517
TRPM2	3.648770479	2.406E-10
SLC2A1	3.599693577	1.523E-08
SLC25A2	3.562610216	0.0066492
TTYH3	3.556155513	3.238E-14
SRD5A2	3.539362927	0.0004093
NT5DC2	3.537002828	7.822E-09
OLAH	3.528001614	0.0097946
CYP2F1	3.525286716	7.236E-06
SLC16A8	3.498553335	0.032518
SLC12A2	3.449781792	1.936E-05
FADS2	3.446033685	0.0001447
SLC2A12	3.412402427	9.032E-05
TDO2	3.378325918	1.959E-05
ENGASE	3.373681042	2.296E-07
GPT2	3.368069219	2.749E-14
PLA2G4E	3.347175143	1.787E-05
CLCN4	3.318936777	5.944E-08
PPAT	3.219215637	9.52E-13
SLC34A1	3.214092312	0.0006312
SLC4A8	3.212319479	1.215E-05
TRPM8	3.202189827	0.0069206
PLCB4	3.185935081	0.0010565
CA5A	3.178163809	0.0015676
AQP3	3.177671256	0.0387851
IMPDH1	3.175064668	1.273E-14
AQP6	3.146103329	5.327E-05
KCNN4	3.140913728	8.839E-08
NME1	3.138041718	1.343E-14
COMT	3.13656122	0.0002117
GALK1	3.135640238	2.018E-08
AKR1E2	3.12217152	1.201E-07
SRM	3.109748137	5.302E-11
KCNK15	3.104814653	0.0041322
B3GNTL1	3.098534566	1.716E-16
PLA2G4D	3.089615528	0.0008353
CAD	3.078883583	8.117E-14
PHGDH	3.076814365	2.731E-06
CLIC3	3.072761468	3.66E-05
UCKL1	3.059229585	7.671E-08
SLC5A2	3.05289285	5.328E-06
SLC9A7	3.047677927	5.166E-06
CHSY3	3.036969448	2.583E-07
GRIN3B	3.028865821	1.174E-05

ATP6V1B1	3.026568527	0.0095094
FASN	3.017549363	4.896E-07
SORD	3.007313632	3.965E-11
SCLY	2.965925714	2.777E-12
CACNA1D	2.955732961	9.74E-10
SHMT2	2.944687408	1.118E-14
HS3ST3A1	2.891530695	0.0019945
CYP4F3	2.855496203	1.366E-06
SDR16C5	2.82246357	0.0007496
GMPR	2.813741609	1.322E-05
RRM2	2.808355206	4.04E-11
ASNS	2.804284595	1.356E-11
MTHFD2	2.792407155	9.977E-13
UGT1A6	2.757365953	0.0002385
MOGAT1	2.752064673	0.0264211
PSPH	2.7403054	1.444E-13
SLC7A6	2.726475617	2.4E-10
FADS1	2.719403756	5.841E-06
TSTA3	2.716147848	3.506E-09
SLC30A2	2.699216173	0.0049679
DHCR7	2.698469968	1.057E-10
SQLE	2.692207949	1.181E-07
SLC1A7	2.690309914	0.0029284
AANAT	2.683163495	5.143E-06
SLC35E4	2.678964983	4.211E-08
NANP	2.640744794	5.272E-11
LYZ	2.624963116	0.0145508
CYP39A1	2.613911535	1.204E-05
CLCN1	2.609753837	0.0011299
SLC6A1	2.59745997	6.914E-06
CYP2S1	2.596625777	8.085E-08
SLC22A15	2.591876409	2.445E-05
SLC1A5	2.591435173	3.511E-10
PTGES	2.57639402	2.797E-05
TXNRD3	2.560404361	7.641E-12
HYAL3	2.556293422	1.562E-10
NOS3	2.550210266	2.121E-09
UGGT2	2.544055641	1.397E-08
SLC25A27	2.519363468	2.782E-05
SLC9A5	2.519172785	1.274E-06
PRDX4	2.516256275	2.201E-10
ABHD1	2.515085255	0.0001817
SLC25A29	2.507051526	5.328E-10
GNMT	2.506953571	4.005E-06

HPDL	2.48087067	2.828E-07
ADCY3	2.475651199	1.876E-11
PLA2G4B	2.471793261	3.922E-05
SLC12A8	2.471215031	1.041E-10
CA8	2.462362175	0.0417399
SLN	2.457325867	0.0406151
ABCB6	2.45205568	1.826E-07
LYG1	2.451807303	0.0034945
LIPG	2.44976216	6.624E-10
SLC25A22	2.446506712	6.533E-11
MGAT5	2.445721381	9.414E-08
GSTP1	2.437620638	2.801E-09
ISYNA1	2.430844711	0.0011114
PLCG1	2.416434472	6.824E-08
INPP5D	2.4105293	2.352E-10
PAICS	2.407777157	1.197E-12
SLC7A1	2.40722898	1.784E-11
MCOLN3	2.398114481	0.0202281
KCTD14	2.393677921	3.032E-11
SLC11A2	2.393420864	9.566E-11
SLC43A1	2.392762721	4.037E-09
RDH12	2.384974968	0.0001036
SLC22A2	2.382681257	0.0045577
ATP2A1	2.377873075	1.167E-06
PNPLA1	2.367194307	0.0044088
ABCC1	2.357794188	7.336E-12
TGDS	2.353973188	1.278E-11
ODC1	2.350438464	6.447E-10
CHST1	2.348671641	0.002574
GLA	2.345020002	2.807E-10
MOCS3	2.34324137	1.737E-09
NAT9	2.333667834	5.182E-15
TRPV5	2.32144449	0.0431632
AMPD2	2.316138052	1.825E-18
LPCAT1	2.314704119	2.623E-07
AADAT	2.310623307	5.52E-07
GABRB1	2.309081409	0.0130141
SLC3A2	2.300406737	2.566E-15
FMO3	2.293680996	2.797E-06
PLCH2	2.280303064	0.0006103
FADS3	2.278372406	5.679E-07
FUT7	2.276874576	3.846E-05
SLC29A1	2.271616354	2.074E-09
TKT	2.27101136	9.492E-15

NSDHL	2.268511586	3.035E-10
NUDT1	2.261460963	6.569E-13
KCNJ3	2.250392378	0.0487016
SFXN3	2.247026243	6.547E-14
SLC22A14	2.237384733	0.0257972
MANEAL	2.236015551	5.883E-07
FPGS	2.227107412	3.523E-12
HYAL2	2.223205483	1.784E-07
DIO3	2.219649695	0.0051563
ADA	2.211516121	1.2E-06
B3GNT4	2.205060216	0.0002218
PPA1	2.202872224	3.515E-14
NAT10	2.199385741	2.647E-13
ATP6V1E2	2.197744963	3.351E-13
NQO1	2.187435674	3.298E-06
MTAP	2.186788713	4.52E-09
SLC25A32	2.185116877	8.549E-11
CLCN5	2.183708089	2.418E-06
QPRT	2.173159241	3.447E-05
ARSE	2.164970816	8.863E-05
SLC25A15	2.159474923	2.256E-12
FOXRED2	2.137391602	1.864E-09
TBXAS1	2.13723988	3.212E-05
PRPS1	2.135805396	9.354E-12
ATIC	2.132894426	1.509E-15
ALG3	2.130204291	5.175E-13
SLC2A8	2.121960375	0.0001025
GPX2	2.120571756	2.053E-08
KYNU	2.117703649	0.0040846
TM7SF2	2.115533777	8.099E-05
ACBD6	2.111277456	3.127E-11
EBPL	2.108272802	7.85E-16
SLC25A6	2.106396103	0.005432
ATP7B	2.101601192	1.048E-06
OAS3	2.101219547	1.117E-05
HSD17B10	2.100700113	7.462E-08
PGAM5	2.098008602	1.475E-14
HSD17B7	2.096412961	2.63E-09
KDM1A	2.094769671	0.0026225
ENOPH1	2.093643713	5.282E-16
CSGALNACT1	2.085704871	0.0102347
CATSPER1	2.083022194	0.0004251
GGH	2.076016951	1.074E-05
ATP6V1F	2.075973047	3.896E-12

SLC19A1	2.065621931	1.206E-08
ME1	2.06001292	8.457E-05
AOC2	2.05909596	0.000367
FUT8	2.056152991	1.163E-07
FDXR	2.05267482	3.39E-05
SOD3	2.050508604	0.014336
CLCN7	2.049635565	4.448E-08
ST6GALNAC5	2.049347015	0.0478804
ABCA13	2.047937329	0.0129028
SLC22A13	2.045918169	0.0047678
SLC39A4	2.045897646	0.0002843
DTYMK	2.045463798	1.251E-12
ACSL4	2.044477468	1.676E-07
OAS2	2.044282184	0.0019678
ST3GAL2	2.041070734	1.562E-14
NMRAL1	2.027242092	8.399E-09
PEMT	2.0211477	5.888E-08
SRXN1	2.020903999	9.875E-07
ABCE1	2.012955073	1.256E-07
PLA2G16	2.008563034	3.089E-05
TK1	2.008014866	4.043E-08
NQO2	2.005248232	4.301E-10
CPOX	2.004804913	2.018E-13
SLC19A2	2.003382827	3.972E-11
GNPDA1	2.003315489	7.479E-14
PIP5KL1	1.996942445	0.0050994
GART	1.995952363	6.719E-12
B3GALT6	1.992880012	3.034E-10
AFMID	1.992178322	9.427E-12
ELOVL5	1.988195875	5.903E-08
ENOX2	1.985128651	6.667E-08
SLC16A4	1.98375477	0.0009606
ACSBG2	1.981963115	0.0042531
RPIA	1.979089434	1.738E-14
SLC4A2	1.974763869	7.885E-12
KCTD13	1.974705488	2.728E-10
ST3GAL1	1.9741619	0.000539
ENO3	1.973400816	2.171E-06
KCNQ1	1.973262517	6.941E-06
CHRNA5	1.970670015	4.068E-07
MPO	1.968532037	0.0072941
IDUA	1.953045992	3.122E-05
MFSD3	1.946884214	4.975E-08
NAA20	1.946332399	1.431E-08

NUDT14	1.942769411	4.306E-07
GUK1	1.942668678	1.389E-05
DGKH	1.938525827	0.0313956
PGK1	1.938470758	2.717E-09
DPM2	1.93060694	8.731E-15
NEU3	1.927464925	5.387E-13
LPCAT2	1.927267507	2.991E-06
LDHB	1.92128551	2.54E-05
KCNS1	1.916357171	0.012999
LIPM	1.914939329	1.162E-05
SLC25A14	1.906919438	3.902E-11
HKDC1	1.901938436	3.019E-05
UAP1L1	1.899836842	2.91E-06
PFAS	1.887159029	2.222E-10
ARSI	1.886213745	0.001558
GMPS	1.883957529	5.01E-12
SLC2A3	1.879901685	0.0098836
NIT2	1.879167774	4.01E-13
DGKZ	1.872058732	3.35E-07
PTDSS2	1.870340462	2.788E-06
LPIN3	1.863059494	0.0003112
GPX4	1.858102361	2.396E-05
ENO1	1.8572014	1.508E-10
NUDT8	1.856828526	0.0015448
POFUT1	1.856001371	2.831E-05
PIGU	1.855421238	9.595E-08
SLC43A3	1.853481274	0.0055634
RDH10	1.850277201	3.265E-06
DEGS2	1.850039438	0.011111
ACSS1	1.845070091	0.0021626
HS3ST1	1.84494562	5.074E-07
CTPS2	1.844901555	7.752E-08
IMPDH2	1.843843502	2.391E-13
SRD5A3	1.835480879	3.624E-05
KCNE3	1.834449041	1.562E-05
G6PD	1.832078435	3.042E-09
GAPDH	1.830734062	3.883E-08
HK3	1.82736789	0.002742
PTGES2	1.826143705	2.173E-06
FAAH2	1.826107558	2.051E-07
KCNAB2	1.824531451	4.18E-07
PTDSS1	1.81667998	4.411E-12
HPRT1	1.815167316	2.288E-07
ABCC10	1.814141531	3.158E-10

ENTPD6	1.808501835	3.909E-06
SLC39A6	1.807207989	6.367E-10
B4GALT7	1.807139695	1.669E-07
SLCO3A1	1.801567313	0.000987
CYB5R2	1.800912377	3.634E-05
D2HGDH	1.799251574	0.0083821
DPM1	1.797959566	2.994E-06
SLC12A9	1.797620521	4.429E-11
DCTD	1.796650384	1.229E-12
PLD6	1.7965906	2.918E-05
NNMT	1.79613195	0.0125347
ACYP1	1.795397384	1.515E-08
GSTCD	1.793980525	9.564E-07
PLA1A	1.792393273	0.0002294
B3GALNT2	1.792119359	9.04E-08
NAT14	1.791968001	0.0002775
FXN	1.791051125	2.946E-09
ALDOC	1.786812432	0.003686
MFSD10	1.784853236	1.491E-10
SLC1A3	1.779565023	0.0040762
SLC38A7	1.778555362	1.659E-12
SLC1A4	1.778534931	2.235E-09
NMNAT3	1.777518681	4.265E-06
FLAD1	1.771111688	1.706E-09
PPOX	1.759486313	1.304E-08
PDE7A	1.758264882	2.982E-06
SLC35C2	1.756597391	3.483E-10
ACLY	1.754375198	1.532E-13
ATP8B3	1.753045285	0.0052379
KCNMB3	1.752748111	0.0124033
GLRX3	1.752122061	1.576E-10
INPP5E	1.750302584	2.551E-10
DHODH	1.748955944	3.236E-14
ADK	1.746036697	3.502E-08
SLC16A10	1.741386738	0.0064884
NOX1	1.739690128	0.0003884
IDH3G	1.738715436	0.0014997
ALDH1A3	1.736475125	0.0001932
GYG2	1.735599241	0.0011532
GCAT	1.734939489	3.265E-05
NUDT5	1.734824934	1.226E-11
G6PC3	1.734642163	1.452E-11
HMBS	1.731417157	2.345E-08
THNSL1	1.729897096	4.249E-07

ENTPD2	1.728526722	0.0011413
COX19	1.722693538	2.45E-11
HCN3	1.717042105	0.0004555
HLCS	1.713888176	8.802E-10
SLC29A3	1.713052697	8.727E-10
ACACA	1.708772723	6.16E-07
MTHFD1	1.70724189	1.733E-09
AMDHD2	1.706117564	0.0002519
SLC29A2	1.705653748	5.935E-08
WWOX	1.705073898	3.115E-07
ACOT9	1.704093611	9.078E-08
SPNS1	1.699762939	7.308E-08
SLC25A39	1.695419379	1.688E-09
CRLS1	1.691570829	1.583E-07
ABCA2	1.686876483	0.000192
PCBD1	1.683525824	3.352E-12
CSAD	1.683217783	2.607E-05
PFKFB3	1.682075107	1.315E-06
SMS	1.68008641	6.436E-07
SULF2	1.677546532	1.778E-05
EBP	1.675577693	1.04E-06
GSTO2	1.672759558	1.814E-05
GPI	1.672712294	6.083E-11
CHID1	1.672484862	2.698E-07
PNPO	1.671745842	2.104E-11
ITPR2	1.669755012	0.0066497
PRPS2	1.668838837	6.879E-07
GNPNAT1	1.665878381	8.672E-07
PIK3R2	1.665662843	2.508E-10
PLCB1	1.66564503	0.0140354
POMGNT1	1.65770844	9.151E-10
SLC22A1	1.65543484	0.0084298
GPX8	1.652474564	0.0003825
PYCR2	1.651545468	1.599E-11
ABCF2	1.651304736	3.809E-12
CYB5B	1.649858556	2.027E-08
MOGS	1.648227097	8.018E-15
SLC41A1	1.640666527	8.422E-07
SRD5A1	1.638841106	1.783E-07
ORAI1	1.637873714	0.000101
SLC27A1	1.637405732	1.351E-05
SLC39A3	1.635394777	1.66E-05
NEU1	1.633580337	1.752E-05
IDH2	1.632767788	0.0001761

SMPD4	1.632513848	1.535E-08
PIGL	1.628721605	1.834E-07
MVD	1.627569105	0.0138453
ALG10	1.623878789	0.0016513
OGFOD1	1.623703698	1.103E-12
COMTD1	1.622924812	0.0022312
ITPA	1.622876816	2.158E-09
LCTL	1.622584274	0.0072522
SULT1A3	1.619976276	0.0162823
MOCOS	1.617446578	2.75E-06
PGD	1.617331374	1.366E-08
CYP51A1	1.616535586	0.00112
ALDH1B1	1.616380812	5.235E-07
ABCC11	1.614653128	0.00428
FOLH1	1.614626395	0.0038651
HSD3B7	1.614535427	1.945E-05
FLVCR1	1.612704155	0.0001789
UMPS	1.612268112	1.867E-12
GLCE	1.611693214	8.612E-06
MMEL1	1.609588782	0.0329753
GLO1	1.608071668	2.271E-06
SLC35B2	1.606861828	5.865E-11
SLC7A8	1.605750668	0.0008973
LPGAT1	1.602026766	0.0003511
NME4	1.598415136	0.0008831
AGK	1.597007537	7.668E-08
ATP11C	1.596898322	0.0241738
NFS1	1.595154639	1.421E-06
DHRS13	1.593812166	0.0004138
CPT1B	1.590914625	0.0014799
GCSH	1.590467995	1.15E-05
DPYSL2	1.590315273	0.007855
ADSL	1.58922883	2.565E-07
CATSPER2	1.585907202	0.0258124
SLC25A19	1.581818713	1.86E-09
AASDHPPT	1.57936923	2.426E-07
SLC16A13	1.576895195	6.69E-06
ADO	1.576838281	6.694E-12
ALG1	1.57666023	2.528E-11
PIGQ	1.573417538	7.828E-06
ATP13A2	1.572408	3.296E-06
PFKFB4	1.572176147	0.0170663
CHDH	1.57056834	1.431E-05
APRT	1.569441862	3.733E-08

SLC7A4	1.569172067	0.0320166
SCNN1D	1.567235079	0.0176377
SLC39A13	1.562720505	3.832E-08
LPIN1	1.560039519	4.547E-05
HS2ST1	1.559175611	9.185E-06
SLC41A3	1.55775483	9.084E-12
SLC26A1	1.557275318	0.0005922
SCN8A	1.556813837	0.0147318
SLC25A10	1.555278248	6.346E-06
TAP1	1.555274976	0.0010415
MIP	1.551935917	0.0087486
ATP13A1	1.551749556	2.479E-08
ELOVL3	1.550111982	0.0403238
SLC25A17	1.545367495	1.679E-06
TYMS	1.541958749	0.0048466
TALDO1	1.541432471	3.931E-08
SLC15A4	1.538262513	2.392E-10
PFKM	1.532642436	4.839E-06
CHSY1	1.530318461	2.11E-05
AGMAT	1.52719744	3.5E-05
PCYOX1L	1.52487759	1.772E-05
ALG5	1.524207308	1.5E-05
ABHD11	1.52120718	5.873E-08
PGM2L1	1.520851367	0.0345786
GSS	1.519058472	1.126E-06
PGM3	1.517852476	1.196E-06
ENPP5	1.517821599	0.0006579
KCNC3	1.517152677	0.0039774
SEPHS1	1.516996978	1.452E-12
MMAB	1.515826636	3.809E-06
NUDT19	1.515231207	1.267E-07
KCNG2	1.514034589	0.0413251
SDS	1.510523269	0.0361237
KCNC4	1.508562406	0.0007758
PRDX2	1.50746095	1.758E-05
LYPLA1	1.505320883	0.0005062
THEM4	1.504468794	8.932E-07
PIGW	1.502935668	2.205E-05
ALG8	1.502916173	3.748E-09
B4GALT2	1.502174337	1.981E-07
SLC35B4	1.501353026	2.031E-06
ATP13A3	1.501137815	0.0007717
ATP9A	1.495495447	0.0016819
ABHD12	1.491416995	6.819E-05

VKORC1L1	1.484789788	1.572E-10
ABCA7	1.484605296	0.0003795
NME2	1.483113504	1.047E-05
FUT10	1.483083596	0.0009537
ADPGK	1.48108026	3.103E-08
PISD	1.480017828	2.1E-11
AMPD3	1.479868514	0.0002343
EDEM2	1.478739165	4.044E-06
DPAGT1	1.476845604	1.475E-10
FUT4	1.476743104	8.79E-05
PGS1	1.474677955	6.848E-07
DGKE	1.473648229	0.0002604
LDHA	1.471346438	2.343E-05
CHST14	1.471255533	2.653E-06
ABCB8	1.46813779	7.277E-08
GALNT4	1.467977835	0.0295528
FUT11	1.466590901	1.064E-05
ACOT7	1.463697289	9.331E-05
POR	1.462898297	2.766E-05
TCIRG1	1.46227052	0.0009474
ARSJ	1.45318523	0.0345402
GLS	1.452522704	0.0103366
MCAT	1.452429238	2.253E-07
GPX7	1.4498076	0.01706
CSGALNACT2	1.447807107	0.004197
FUK	1.447670062	5.104E-05
DHRSX	1.444590126	3.17E-06
CYB5RL	1.442817076	2.468E-05
PTGES3	1.441441387	5.132E-06
PIGA	1.43925554	0.0029415
ATP6V1C1	1.43803811	7.712E-06
UGGT1	1.433682277	2.052E-05
PAPSS1	1.432711659	5.417E-09
NDOR1	1.43114538	5.347E-06
CACNA1F	1.430176212	0.0133018
CLIC1	1.429761986	9.818E-08
MDH2	1.429501292	3.09E-10
DDOST	1.429165024	3.919E-08
NUDT21	1.428746389	1.534E-05
PMM2	1.427715592	1.036E-05
NUDT4	1.427714885	0.0034421
C1GALT1	1.426612591	7.343E-05
SLC30A7	1.425134151	0.0111137
SLC35A2	1.424324091	1.867E-07

DOLK	1.422845844	1.288E-07
POFUT2	1.420125772	3.413E-08
CHRNA10	1.41724825	0.0036985
SMPD2	1.416948105	3.709E-07
ASAH2B	1.416109726	0.0005162
SLC24A1	1.416034368	4.705E-06
RDH11	1.412957785	3.975E-06
ORAI2	1.411857701	9.098E-05
SLC10A3	1.409950864	4.119E-06
AMDHD1	1.409087523	0.0297169
B3GNT9	1.40872159	2.135E-05
B3GAT3	1.407000053	9.38E-06
NIPA1	1.406952102	1.511E-05
TECR	1.406254688	6.395E-09
SLC25A36	1.405955926	0.0071484
NME3	1.405180626	0.0077777
ALDH3B1	1.405173185	0.0031442
RRM1	1.404912636	9.786E-05
ATP2C1	1.402454694	2.469E-07
KCNE4	1.400871195	0.0243375
GXYLT1	1.400467172	0.0006489
NDUFAF2	1.399220075	6.086E-05
PRDX1	1.398969698	2.568E-05
HSD17B12	1.398321301	1.758E-05
MTR	1.39605603	0.0010426
ATP6AP1	1.393610235	5.106E-08
DECR2	1.390800824	0.0048603
NAA50	1.390768987	4.996E-05
SFXN2	1.389693702	0.0001756
ASNA1	1.388933502	5.166E-05
CATSPERB	1.387340545	0.0413617
B4GALT3	1.387121197	1.154E-08
PIGM	1.387025265	9.855E-07
MAN2A2	1.385242875	1.216E-06
NME7	1.384599263	0.0005687
LBR	1.382434	0.0004078
GPHN	1.381417783	8.023E-06
GALNT2	1.380691471	2.519E-08
CYBA	1.38029266	0.0046525
DPM3	1.379583993	0.0396362
CA5B	1.379494623	0.0136368
TMCO3	1.377386993	0.0002664
CRYZ	1.37514644	0.004567
EXTL3	1.374905804	0.0005608

LCAT	1.374695521	0.0160197
UXS1	1.372880463	8.018E-12
TYMP	1.372540649	0.0252857
HSD11B1L	1.371098828	0.0069358
VDAC1	1.370518851	1.647E-05
MECR	1.367545996	9.826E-08
CATSPER3	1.366901224	0.0017936
DCXR	1.366831298	0.0024544
CYB561	1.366700665	7.772E-06
NUDT3	1.36549678	0.0003499
PLCH1	1.365067248	0.0091324
GLYCTK	1.363822805	0.0120597
MANBAL	1.363616432	1.987E-05
HMOX2	1.362553486	2.174E-05
ACAD9	1.361271402	3.925E-07
RPE	1.357410195	0.0003185
MAN1B1	1.356836375	1.008E-05
SLC39A1	1.354811765	2.61E-09
SFXN4	1.353710799	9.576E-06
PDXK	1.352985867	0.000153
SLC36A4	1.352878026	0.0444036
ASNSD1	1.351744078	5.612E-05
SLC38A2	1.350667336	0.0012488
KCTD20	1.350244562	2.589E-05
HSDL1	1.350152433	8.192E-05
PCYT2	1.349673225	0.0006576
LYPLAL1	1.349459322	0.0058163
PTS	1.34676622	0.0019097
CYC1	1.346395626	0.0243944
DHFR	1.346059036	0.0052837
FDPS	1.343179175	0.0018951
NDUFS8	1.342900915	0.0204896
MMACHC	1.340388184	0.0001865
PLA2G15	1.339727254	1.471E-05
FOXRED1	1.338254105	4.069E-05
AGPAT5	1.337133334	0.0016952
FAAH	1.336169774	0.0127773
KCNAB3	1.335564974	0.0394099
XYLB	1.334769452	0.00047
TPI1	1.334306929	1.415E-05
GRHPR	1.331718634	0.0005673
ABHD14A	1.331134458	0.0203676
PCBD2	1.330970627	0.0009812
GMDS	1.32998687	0.0022401

MCCC2	1.326617391	0.0001315
ACOX2	1.326271093	0.0327946
GLB1L	1.326241444	0.0012954
SAT1	1.326177224	0.0163694
CHPF2	1.325294683	1.164E-06
MAT2A	1.323468459	0.0001183
B4GALT6	1.323449438	0.0140593
ALG2	1.323052638	3.709E-05
GLB1L2	1.322345735	0.0068154
PFKP	1.321454632	0.0020178
PON2	1.320762169	0.0111097
ADCY4	1.320141917	0.0165821
GLB1	1.317062753	1.014E-05
SLC25A37	1.315982166	0.0034965
PI4K2B	1.314567754	0.0049253
SLC12A4	1.313938932	0.0110975
SLC37A3	1.313590576	9.731E-05
MAGT1	1.312752845	0.0024615
HACL1	1.312308768	0.000152
L2HGDH	1.311771815	0.0002553
PIGT	1.31104011	8.026E-05
SLC25A26	1.310422205	5.66E-07
MAN2C1	1.310368313	0.0048485
KDM1B	1.309293479	0.0047426
GALNT11	1.309134564	0.0001475
IPPK	1.307794676	2.068E-05
COASY	1.304755728	5.859E-07
ALDH16A1	1.303954446	0.0036425
SLC12A7	1.303710898	0.0001047
HIBADH	1.303646187	3.389E-05
ABCB9	1.303461694	0.0030863
PIGX	1.302957099	0.000502
PRDX5	1.302376579	0.0384481
XYLT2	1.300831636	4.751E-05
SLC35B1	1.299627113	3.77E-05
DGUOK	1.29812144	0.0004838
STARD3	1.29580348	0.0003214
ANKH	1.294329791	0.0067354
MVK	1.293830077	0.0004277
SLC2A6	1.290650851	0.0455855
ITPR3	1.288596639	0.0106344
LCLAT1	1.288584122	0.0035378
POMT2	1.288212379	1.601E-05
ST6GAL1	1.28769494	0.0290364

NDUFS5	1.287489612	0.0109289
ATP6V0B	1.285947014	0.0255529
SLC30A6	1.285090602	0.0003026
ATP6V1G1	1.283303003	0.0004193
COQ10A	1.281777472	0.0042634
QDPR	1.28172572	5.184E-05
GLT8D1	1.281301368	4.2E-06
GSTO1	1.279434455	0.0362431
IDH3B	1.278714457	0.0114337
NDUFS6	1.276734322	0.0250247
APEH	1.276064482	0.0007678
GCDH	1.273544029	0.0001162
NDUFB11	1.272636624	0.0005989
UCK2	1.272327067	0.0005022
KCTD6	1.271841926	0.0027851
GALK2	1.270894752	0.0016862
ABHD10	1.270796918	0.0011926
FDFT1	1.270027035	0.0046322
DAGLB	1.268782344	1.262E-05
TXNRD2	1.268258979	0.00029
MICAL1	1.267655411	0.0085877
MANBA	1.267220556	0.0009114
ABCB7	1.266329385	0.0003918
UROS	1.265253135	1.746E-05
COX18	1.26374982	0.0026369
INPPL1	1.26122588	0.0001317
ACY1	1.252979415	0.0343142
GANAB	1.252107372	5.954E-05
GUSB	1.250367143	0.0093601
AACS	1.250025134	0.0011068
GOT2	1.248342182	0.0001629
GCNT1	1.245938565	0.0315091
SLC16A3	1.245624809	0.0120137
MTHFSD	1.24431429	5.303E-06
ORAI3	1.244199979	0.0008656
DAGLA	1.243649341	0.0372925
PIGC	1.241460367	0.0001929
ALG11	1.24091013	0.013615
GCLM	1.23976069	0.0289327
FAHD2B	1.238682123	0.0305157
ME3	1.238644029	0.0070024
SLC2A11	1.237926451	0.001512
IAH1	1.237477005	0.0017799
PGM2	1.23620431	0.0006196

SYNJ2	1.233921127	0.0045814
IMPAD1	1.232329548	0.0188159
TMEM104	1.230778123	0.0012848
ABHD13	1.230449989	0.0323514
AKR1A1	1.227449324	0.0087753
SDR39U1	1.227369919	0.0082074
KCTD17	1.226568041	0.0208218
GMPPA	1.224262213	0.0001566
ILVBL	1.224214553	0.0414074
SLC39A11	1.224127648	0.0169674
GALNT3	1.223727012	0.0258275
SLC33A1	1.217640428	0.0036814
GLRX2	1.216881645	0.0021096
LTA4H	1.215286565	0.0020667
PIGO	1.215094241	0.0009698
THNSL2	1.214902158	0.0482499
OGFOD2	1.21346533	0.0029219
AASDH	1.21335716	0.017424
NUDT15	1.211060788	0.0050132
PIP5K1A	1.210422135	0.0003364
NDUFA7	1.209929255	0.0067573
ABCF3	1.208549245	2.715E-05
ABCF1	1.206766261	9.321E-05
CDADC1	1.206765465	0.0010944
NDUFA11	1.204305913	0.0489459
ALDH5A1	1.200863178	0.0227233
PANK2	1.200455662	0.0042159
PDSS1	1.200307235	0.0056861
ATP6V1H	1.199739898	0.0001611
MANEA	1.199022209	0.0454449
DEGS1	1.194616826	0.0178972
ALDOA	1.192081662	0.0242374
PDE12	1.190942967	0.0049726
NAGLU	1.190321578	0.0029425
NDUFA4	1.190159761	0.0334705
SGSH	1.188616656	0.0342009
PRPSAP1	1.185523159	0.0006754
NPC1	1.184838166	0.0067939
GGPS1	1.18449169	0.0038298
FAH	1.181687119	0.0150581
NME6	1.179901279	9.015E-05
HEXB	1.179167664	0.0016997
GCLC	1.17798602	0.0061042
STARD3NL	1.177373558	0.0215721

SLC45A4	1.17698608	0.0230156
ATP6AP2	1.176957151	0.013968
PLD2	1.174558702	0.0142441
SLC30A5	1.172519099	0.0011106
VKORC1	1.172410538	0.0239922
EXT2	1.171612213	0.0017735
CEPT1	1.170781018	0.0463182
ADSS	1.169913079	0.0078176
CHST12	1.169018452	0.0259349
ASMTL	1.165158837	0.0473332
RDH13	1.162879963	0.0242324
FHIT	1.162543954	0.0308228
NAA40	1.162203667	0.0210646
PIGF	1.161769054	0.0143782
BPHL	1.158615799	0.0255103
PIP4K2B	1.156916037	0.0021181
STARD7	1.153395795	0.0006992
CLCC1	1.150345668	0.0214228
FAHD2A	1.145671721	0.0113275
GNPAT	1.144807278	0.0228335
CNP	1.140108588	0.0049407
FUCA2	1.132584013	0.0316788
PI4K2A	1.116129077	0.0135486
MTRR	1.115064461	0.0218207
SLC35E1	1.11467414	0.0023498
FN3KRP	1.11278919	0.0245586
SLC25A28	1.111760564	0.0260411
SLC4A1AP	1.1043142	0.0399828
GFOD2	0.902330973	0.0349535
MGAT1	0.899774398	0.0263234
CS	0.897467561	0.0181363
SLC25A3	0.89634141	0.013635
GLUD1	0.895817242	0.0331329
NADK	0.892344685	0.0207043
KCTD11	0.891391885	0.0393966
SUOX	0.890129888	0.0336318
AHCYL1	0.887822177	0.010194
SLC39A7	0.887136516	0.0456772
CYB5R3	0.887085391	0.0246302
ATP6V0A1	0.887071045	0.0273862
AGPAT1	0.886111453	0.0029748
NDUFB4	0.883589344	0.0383862
PIGV	0.879869208	0.0185668
AKR7A2	0.877867087	0.0360079

IVD	0.87776798	0.0043081
BCKDHB	0.875471983	0.0387151
PMVK	0.873306793	0.028297
PDSS2	0.872869869	0.0032434
NDUFA10	0.869810491	0.0011947
PIK3CB	0.867945042	0.0421763
COX17	0.867527295	0.0210502
COQ5	0.866046621	0.018934
ATP6V1E1	0.86542439	0.0023486
NDUFC2	0.864344686	0.0329779
FAHD1	0.863215448	0.0179405
IDH1	0.859712383	0.018533
SLC25A44	0.858500554	0.0006584
MFSD5	0.857759239	0.0051036
INPP4A	0.856803824	0.0275331
ITPK1	0.856054354	0.0051529
COQ7	0.856001583	0.0002135
PIP4K2C	0.855639932	0.0117351
ALG14	0.855452848	0.0067942
DLAT	0.853246114	0.0055668
COX16	0.853012404	0.0125162
SDHC	0.85147096	0.0017349
AGPAT3	0.847924016	0.0038958
SLC25A16	0.847669881	0.0123878
CMPK1	0.847318601	0.0293276
KCTD21	0.846111383	0.0030742
MGAT4B	0.84527562	0.012188
PDHB	0.84453074	0.0009529
FH	0.843768692	0.0012311
NDUFB2	0.843311321	0.0004688
B4GALT4	0.840413773	0.0356625
ISCA2	0.839340373	0.0199258
CANT1	0.839274091	0.0237265
ASAH1	0.836715486	0.0022584
KCTD5	0.836068547	0.004299
FAR1	0.833850908	0.0211146
PCYT1A	0.833774323	0.0002752
MAT2B	0.833634999	0.0045446
FA2H	0.833108637	0.0217478
NDUFB9	0.831869037	0.0129696
COQ6	0.83124032	0.0002177
ALDH9A1	0.830528618	1.183E-05
GSTA4	0.830468709	0.0362999
PPIP5K2	0.829658729	0.0283834

CYB5R4	0.827851682	0.0071871
PCTP	0.826848208	0.0033584
HNMT	0.82483339	0.0463518
FUT2	0.824205455	0.0113011
DHRS7	0.823263601	0.0321446
NPC2	0.823148554	0.0095798
ABCD4	0.822927327	0.0019559
GAA	0.821664473	0.018966
PANK1	0.820518413	0.0429946
PIK3R1	0.820389914	0.0237787
NUDT22	0.818723797	0.0018406
NDUFA2	0.817078347	0.0263122
ACAD10	0.816863908	0.0027635
KCTD10	0.815059541	4.857E-05
HYI	0.814136675	0.001868
GLRX5	0.81354209	1.734E-05
NDUFA6	0.810936128	0.0172002
COX6A1	0.810714067	0.0067598
KCTD18	0.810084406	2.861E-06
ISCA1	0.807817501	0.0009012
GLOD4	0.807540766	0.0001385
UQCRB	0.806985436	0.0004933
SLC25A11	0.806933813	0.0018697
FIG4	0.806211409	3.857E-07
PTGR2	0.806052595	0.0008399
ENTPD4	0.804937105	0.0065444
PI4KA	0.804330636	2.778E-05
ST3GAL5	0.803648669	0.012421
MT-ND3	0.803618896	0.0272686
SLC12A6	0.803613834	0.0049947
COQ9	0.80111299	0.0001573
CHPT1	0.800414714	0.0004251
NDUFS3	0.79843463	0.0004856
ATP6V0C	0.795814639	0.0014002
EDEM3	0.794851314	0.0036249
PCYOX1	0.793616169	0.0003032
MGAT2	0.792816494	0.0016027
ENPP4	0.792699605	0.0009515
MAN2B2	0.792079682	2.275E-05
NDUFB5	0.789977902	0.0047999
CTBS	0.789773298	0.0017445
LIAS	0.789428361	0.0003055
MFSD9	0.788933432	0.0001382
ABHD4	0.786984342	0.0079969

DHRS1	0.786349076	0.0074504
IDS	0.785967001	0.0299352
SLC35A5	0.785477291	0.0001114
GAL3ST4	0.784182846	0.0078995
KCNK6	0.783403678	0.0024209
DLD	0.781076767	7.407E-07
THTPA	0.780549025	4.068E-05
SLC30A1	0.780104419	0.0042316
COX7A2	0.779737957	0.005219
GSR	0.779692147	0.0004506
PIK3C3	0.778728601	0.0002455
ACOX3	0.778240936	4.922E-07
NAGA	0.778153949	1.231E-06
STARD5	0.777626857	0.0015876
UGCG	0.77730012	0.0021767
VDAC2	0.776699283	2.069E-06
ECHDC2	0.775912163	0.0023942
ACAD8	0.775864933	6.459E-10
SOAT1	0.772580627	0.0047994
FDX1	0.772421615	9.496E-07
HMGCR	0.772039796	0.0124508
SLC35A4	0.771386333	3.188E-07
COQ10B	0.770669989	4.427E-05
MFSD1	0.767966992	6.964E-05
SLCO2B1	0.767937724	0.0032162
LIPA	0.766625731	0.0351347
CHKA	0.766380938	0.0002848
CNDP2	0.766085191	0.0009808
SLC39A8	0.76445667	0.0104678
NDUFS2	0.764359285	1.035E-07
UEVLD	0.762464999	4.722E-05
CERKL	0.762241825	0.0130315
PTEN	0.761339419	0.0030178
MAN2B1	0.758858699	1.391E-05
SDR42E1	0.758742619	0.0031666
DHRS4	0.758599685	0.0004939
AK3	0.75706333	3.879E-06
COX6C	0.756593553	9.982E-07
NDUFB8	0.754881371	2.445E-05
PPCS	0.754352276	0.0001155
PIK3R6	0.753396019	0.0157156
HEPH	0.752285724	0.000501
NDUFS4	0.752159164	0.0002252
UQCRC2	0.750857838	1.332E-07

COX10	0.750183761	1.172E-06
ECHS1	0.749194015	2.827E-07
OGDH	0.749164715	6.497E-07
MICAL3	0.748665708	0.0019349
SDSL	0.748600488	0.0004725
ALDH2	0.748273925	3.134E-07
FUT3	0.747426577	0.0026773
GFOD1	0.745935055	0.0009418
DBT	0.745410512	1.075E-05
PMM1	0.74422858	0.0012616
NAPEPLD	0.743685444	0.000517
ACER3	0.743356044	0.0002031
MT-CO3	0.743273303	0.0053013
PIGN	0.740392269	4.092E-05
KCNJ2	0.739869225	0.0061878
NPR2	0.736184238	0.0071141
COX8A	0.734521109	5.778E-06
SLC22A18	0.733840787	0.0003933
CYB5A	0.733376585	0.0027046
GALNT1	0.733216557	1.089E-05
GPD2	0.733151111	8.262E-05
SFXN1	0.732948836	0.0001097
NDUFA1	0.730846177	9.989E-06
PIP5K1C	0.730447924	6.718E-05
STARD13	0.729745059	0.0042481
NUDT9	0.729347773	9.777E-11
B4GALT1	0.72923188	0.0009455
ATP1A1	0.727448763	0.000188
INPP5K	0.727387363	9.155E-07
FAR2	0.726756019	2.48E-05
DDO	0.726423871	0.0312687
UQCR10	0.725545013	8.434E-05
SLC27A4	0.72501602	1.834E-05
MMAA	0.723842621	2.037E-05
GBA2	0.723812093	1.587E-06
MDH1	0.723794903	8.041E-08
ABCG1	0.723722724	0.00177
SLC16A14	0.723578837	0.0128187
NDUFC1	0.723314739	2.5E-06
PDE3B	0.723035778	0.0080229
NNT	0.720102572	3.436E-06
CRYZL1	0.718871805	7.623E-06
NDUFS1	0.718400197	8.434E-10
LPIN2	0.718139137	2.684E-05

PAOX	0.717533529	2.233E-05
ACBD4	0.717509967	4.595E-05
MAN2A1	0.716945943	7.67E-05
KCTD2	0.716890608	1.201E-12
GSTZ1	0.715964788	0.0002566
DHRS4L2	0.715290836	6.532E-05
CMAS	0.71471641	2.076E-06
ATP10B	0.71414369	0.0004082
SEPHS2	0.712289324	4.956E-05
IPMK	0.709888562	0.0001757
TTYH2	0.709727469	0.0017093
SFXN5	0.707518037	3.044E-05
ALAS1	0.705072505	4.379E-07
SLC40A1	0.704188161	0.0049231
SRR	0.703019034	2.558E-08
SLC44A2	0.701149	1.676E-08
SCNN1A	0.70035449	0.0022092
B4GALNT1	0.69999741	0.0111312
PNPLA7	0.697677126	0.0021627
NIT1	0.697382036	5.114E-09
TRPA1	0.694248728	0.0177798
EXT1	0.691764042	4.068E-09
AK1	0.691320718	0.0001208
CYP2U1	0.690825794	0.000928
ETFA	0.690121179	3.679E-07
XYLT1	0.689898139	6.878E-05
ACOT1	0.689876843	4.959E-05
ABCG4	0.689503326	0.0027628
SDHB	0.688979403	3.097E-08
SLC35B3	0.68818507	3.238E-08
SLC25A12	0.687170234	4.731E-07
ADH5	0.686841996	3.983E-06
ECH1	0.686486543	8.355E-06
NDST1	0.686169859	8.177E-07
ABHD8	0.685100738	0.0002588
MT-ND1	0.685052925	0.0004412
NDUFV2	0.68462501	8.712E-05
PYROXD1	0.684548987	1.067E-08
ACOT2	0.682723289	2.665E-07
AGXT	0.677837294	0.0305449
GALNT7	0.677585555	3.404E-05
ALAD	0.676657573	2.502E-12
MGST1	0.676327187	1.43E-05
ACOT13	0.675064179	1.323E-07

SLC35D2	0.674760884	7.282E-07
UPP1	0.674480153	4.513E-05
SGPP2	0.672332843	1.36E-05
AKR1B1	0.669825369	0.0007793
NDUFA5	0.668504055	6.108E-08
ACOT11	0.668364406	8.921E-05
GAL3ST2	0.668333371	0.005679
ECHDC1	0.668208726	1.605E-08
ADAL	0.667584784	1.375E-07
DERA	0.667177601	6.165E-07
TKTL1	0.664435932	0.0125679
SMPDL3B	0.664315217	0.0003749
CYCS	0.661812307	3.231E-07
SLC27A2	0.661488497	0.0001166
ADSSL1	0.660970382	0.0004822
SDHA	0.660874133	5.534E-09
SYNJ1	0.660525558	3.579E-07
SLC25A24	0.660173901	1.02E-08
CRAT	0.659468125	0.0006406
PLA2G4F	0.658531406	0.0001727
SLC35A1	0.657837376	0.0001089
AKR7A3	0.656974635	0.0001996
MICAL2	0.656600065	3.026E-08
AUH	0.656271623	2.06E-10
NDUFB3	0.655534677	6.755E-07
ADCY6	0.654790435	1.114E-06
COX7C	0.654116422	3.029E-08
PIGS	0.653297874	2.746E-11
SLC16A1	0.652339128	0.0003072
PCK2	0.652167326	0.0007696
ABO	0.651160658	4.813E-05
LPCAT4	0.650757449	2.966E-06
GGT1	0.650414926	0.0165551
ATP1A4	0.64989362	0.026248
NUDT7	0.649759563	8.613E-10
GDE1	0.649439342	3.147E-14
KCTD1	0.648895799	0.000496
ABCC3	0.648824151	6.209E-05
TPK1	0.648429283	0.0001735
STARD8	0.647790084	5.09E-05
ATP6V0D1	0.647535175	2.852E-09
NUDT16	0.646481983	8.338E-12
ABCA1	0.646208396	0.0030934
SLC45A1	0.64561251	0.0007309

CYP4F22	0.64558349	0.0019885
SLC16A2	0.644570183	0.0003687
CBR1	0.643705734	0.0113148
INPP5J	0.64356089	0.0068519
BDH1	0.64165753	1.213E-07
TPMT	0.640321595	2.895E-07
BPNT1	0.638898817	1.202E-08
ATP8B2	0.638328614	8.429E-05
FRRS1	0.637608274	4.624E-05
SLC12A3	0.63726796	0.0169857
ATP8B4	0.635606008	0.003212
SLC44A1	0.634699066	2.98E-10
UAP1	0.634376624	1.491E-10
MPI	0.633970701	1.935E-10
ACADVL	0.63355781	5.392E-12
MFNG	0.63351998	9.303E-05
COX5A	0.632579099	1.319E-08
ALDH3A2	0.631635311	1.936E-13
PFKFB2	0.631068256	3.67E-06
AGL	0.631015362	3.583E-08
PIK3R5	0.630669422	0.0012733
CHST15	0.630245049	0.0020014
ARSF	0.630067318	0.0285456
DOLPP1	0.629780933	1.279E-10
GNE	0.629196694	0.0008297
MTHFR	0.627920383	1.014E-09
GRID1	0.626511021	3.381E-05
SLC20A2	0.625216299	8.056E-07
CERK	0.625192805	3.359E-08
TRPC4	0.623388061	0.0333394
GSTM1	0.62177223	0.0015322
ATP6V1D	0.621490285	2.808E-13
RFK	0.621475112	0.0001852
IMPA2	0.620718671	1.405E-08
SLC17A5	0.618665926	3.03E-08
ST8SIA4	0.618567434	0.0001099
NDUFB1	0.617884081	2.917E-06
DECR1	0.617613895	1.874E-08
SLC25A35	0.616342444	2.421E-09
KCNK5	0.615750164	1.163E-05
SUCLG1	0.61524962	1.616E-09
SLC25A18	0.614977091	1.613E-06
MBOAT4	0.614484024	0.0025533
SLC35A3	0.613778799	1.979E-08

P2RX7	0.613639727	0.0015208
CDS1	0.613358578	2.552E-09
HIBCH	0.61273169	1.822E-11
GNPTAB	0.611108283	3.987E-09
CYP21A2	0.60965095	0.0318134
AMT	0.609008087	7.92E-06
GOT1	0.608966395	4.637E-12
CYP4V2	0.608753951	2.051E-07
SCN1B	0.608066846	0.0004254
GBA	0.607300355	2.101E-11
FECH	0.607006791	9.968E-10
SLC6A17	0.605561414	0.0210759
ACOT4	0.6050101	0.0007194
IDH3A	0.604778552	1.843E-11
SGPP1	0.604153903	2.728E-07
NOX5	0.60297952	0.0058083
MGST3	0.601917896	3.425E-13
ETNK1	0.601456423	0.0024344
CYP2J2	0.598740904	0.007005
MFSD11	0.598547252	9.728E-13
INPP5A	0.598496885	1.277E-10
HPSE	0.597847424	1.492E-05
CYP27C1	0.597843901	0.0030146
DPYSL3	0.595159133	0.0073876
ACO2	0.594607658	2.539E-14
CA11	0.593712881	0.0006946
MT-CO1	0.593681494	1.061E-06
TYRP1	0.59300607	0.0093999
KCNH1	0.592498339	0.0003429
EPHX1	0.592289934	1.458E-09
BTD	0.591067338	3.977E-12
NANS	0.590472442	1.087E-09
KCNK12	0.590355752	0.0078031
ZADH2	0.590181023	7.579E-15
CAT	0.590126998	9.772E-13
CLYBL	0.588687166	7.738E-12
NMNAT1	0.588555012	1.111E-11
KCNN1	0.584704091	6.453E-05
B3GNT5	0.584554204	6.577E-07
SPTLC3	0.584365106	0.0003203
ALDH6A1	0.583539367	1.692E-09
ARSA	0.582891262	8.332E-11
SLC1A2	0.582212348	0.0480866
ABHD6	0.582050151	1.465E-08

ATP1B3	0.581821152	4.694E-09
MBOAT1	0.581200946	8.837E-09
NME5	0.581198088	0.0047023
ADAD2	0.580804654	0.0027868
ST3GAL3	0.580221595	2.95E-05
AS3MT	0.579106733	0.0037376
GSTM3	0.578413108	0.0019564
ACSM3	0.577973146	0.0001636
PLD4	0.577448194	0.0001496
GCK	0.577242158	3.327E-05
ACSBG1	0.577087585	0.0051364
ME2	0.576792547	1.037E-11
ST3GAL4	0.576378628	0.0041032
SLC25A4	0.576312887	4.589E-05
SLC26A4	0.575260182	0.0007537
ACAA1	0.574439758	1.909E-12
GSTM4	0.573927674	7.535E-10
MSRA	0.572912074	4.87E-09
ACSS2	0.57240571	3.619E-09
DDT	0.572143255	1.118E-09
HMGCL	0.57193497	3.196E-14
TRPM4	0.571847162	1.506E-07
PLD1	0.571417969	3.369E-08
SCN5A	0.571398162	0.0003383
GLRB	0.571395897	0.0303014
ITPKB	0.569996052	5.564E-05
UQCRFS1	0.569794365	8.433E-14
AKR1D1	0.566178465	0.0395254
SLC31A2	0.565810739	1.167E-07
CYP46A1	0.563579046	0.0002915
LTC4S	0.563092319	0.0331968
DGKA	0.563044288	5.559E-08
SLC15A2	0.562421511	3.11E-06
ACHE	0.562231063	1.059E-05
ALDH1A1	0.55952283	0.0014397
PANK3	0.559111999	1.81E-06
NUDT12	0.559015893	2.066E-07
KCNA2	0.558909156	0.0016705
COX6B2	0.557864927	0.0038864
ACADSB	0.557864362	4.808E-13
PKD2L1	0.557086083	0.0025604
B3GNT6	0.556212639	0.0498254
DPEP2	0.55447744	0.0004855
SLC6A8	0.553998744	1.581E-05

MT-ND5	0.552356314	1.941E-06
PECR	0.551958024	9.269E-11
SV2A	0.551621915	4.191E-05
SLC6A12	0.551430058	0.0002778
ACSM4	0.551074946	0.0018086
CLC	0.548778619	0.0425558
HADHA	0.548659592	3.409E-17
ST8SIA5	0.548092252	0.0002641
AQP1	0.548046142	4.104E-05
PDE6B	0.546492737	0.0009012
GPLD1	0.546470992	0.0002249
NT5C1A	0.545698538	0.0319408
CLIC6	0.545594033	0.0031105
KCNJ5	0.544667076	0.0047682
SLC2A7	0.544368308	0.0256092
BST1	0.544133881	0.0004709
HADH	0.542521003	1.423E-14
PIK3CD	0.542356997	1.404E-05
ATP2B1	0.542298244	2.395E-09
KCNK2	0.540153736	0.024675
HADHB	0.537124711	2.103E-19
TRPC1	0.534851079	0.0037923
HPD	0.532149092	0.0026297
TRPC6	0.531900374	8.198E-05
B4GALNT3	0.530237029	1.778E-07
GABRA2	0.528151184	9.955E-05
CACNA1C	0.528048426	0.0306066
PTGDS	0.525637037	0.00168
MAN1C1	0.525470204	1.493E-07
IMPA1	0.524673863	6.439E-12
AQP11	0.523954408	0.0042
SLC30A4	0.523513554	0.0002057
ST6GALNAC1	0.523226734	0.0005269
PIP5K1B	0.520044285	7.504E-11
NAALAD2	0.519628477	0.0105753
ACSM1	0.518920191	4.047E-06
KCNQ4	0.518209931	0.0011325
GALM	0.518046501	2.902E-15
PC	0.517571904	1.593E-08
STARD9	0.517284405	0.0002065
PTGR1	0.517064274	0.0086589
PDE4D	0.516460258	7.767E-05
SLC35F3	0.515899434	0.0002561
P2RX4	0.51447993	7.101E-10

ABCA5	0.51405152	2.83E-06
ABHD5	0.513799683	3.002E-11
ELOVL6	0.512032731	1.153E-07
KCNS3	0.509710618	1.062E-06
ABCD3	0.508795117	1.485E-16
SLC25A42	0.50872726	4.499E-08
PDE4C	0.507136157	7.604E-09
LPCAT3	0.50545938	1.329E-10
HVCN1	0.502950104	9.497E-06
KCND3	0.501348264	6.492E-05
TTYH1	0.500852707	0.0012533
SLC18A2	0.500172604	1.535E-06
BDH2	0.498582428	1.16E-08
SLC24A4	0.49820872	1.21E-05
PDE8A	0.497579284	9.01E-13
HDC	0.494833722	1.245E-05
DHDDS	0.492857696	5.996E-18
DMGDH	0.492696677	2.35E-06
CES1	0.486926735	0.0058783
SCP2	0.483952087	2.356E-17
RBKS	0.481661402	9.325E-10
GABRB2	0.479833131	0.0007824
SLC47A2	0.478828984	1.498E-05
KCTD12	0.478592734	2.705E-07
PLPPR5	0.47756961	0.0276075
ABCA10	0.475974914	0.0003998
SDHD	0.47576685	8.334E-15
HGD	0.475105406	2.576E-08
SLC26A7	0.473698049	0.002037
RYR1	0.472703507	1.258E-06
ITPKA	0.470711717	1.385E-08
CDA	0.470376695	2.337E-07
SLC6A16	0.470134255	8.564E-05
CLCA2	0.469077105	0.0273016
LIPH	0.468691628	7.722E-11
ADCY9	0.466944018	1.652E-12
SLC4A9	0.466534675	0.0013123
SLC6A18	0.466309679	0.0027062
MAN1A1	0.463851081	6.609E-12
SLC35D1	0.463741002	2.187E-13
PHYH	0.463605857	1.904E-13
DSEL	0.461125119	0.0001376
KCTD9	0.460350587	3.508E-15
CKMT1B	0.460045318	7.286E-12

HK2	0.459800974	1.008E-07
SLC39A5	0.458729338	1.588E-08
A1CF	0.457931337	2.411E-10
ST6GALNAC3	0.457707534	6.343E-12
ATP8A1	0.457028538	4.937E-10
PDE1B	0.456510758	1.795E-07
GALNT12	0.456221388	1.164E-11
SMPD3	0.456210488	8.124E-11
EHHADH	0.455828192	6.466E-12
CROT	0.454784979	3.935E-12
PTGIS	0.452608522	0.000168
PLCL1	0.452224651	4.265E-05
GRID2	0.450117541	0.0230426
ACADM	0.448159138	9.96E-17
AKR1C3	0.44798459	9.354E-08
CYP4F12	0.44787583	9.166E-08
MGAT4A	0.447009773	1.53E-12
SLC2A5	0.446235681	0.0361229
AK5	0.445727209	0.0054993
TF	0.443905267	0.006071
PCYT1B	0.443458135	0.003074
TPO	0.441862338	0.0021205
CYP26B1	0.44182009	3.112E-07
CACNB2	0.440434298	1.566E-06
G6PC2	0.44020684	4.481E-05
MT-ND6	0.440152316	5.711E-08
CPT1A	0.439027296	2.441E-15
MGLL	0.438289836	2.387E-12
CLIC2	0.437589387	1.167E-08
SLC22A23	0.437124491	3.465E-10
SLC47A1	0.435420053	1.821E-07
GLOD5	0.433284834	1.893E-12
CACNA2D3	0.432658952	6.059E-06
PLA2G10	0.431906663	1.385E-10
HSD17B11	0.431345739	1.577E-13
ITPR1	0.430443195	4.856E-07
AKR1C1	0.430069724	2.552E-05
COX7A1	0.429061314	0.0002184
CHI3L2	0.427681777	0.0043849
ATP2A3	0.425979022	5.36E-12
ACAT1	0.42545754	1.674E-22
SLC9A1	0.42203531	1.207E-11
CLCN2	0.421158079	1.485E-10
CACNB1	0.421117366	2.752E-09

PDE1C	0.41986172	5.602E-09
SLC6A9	0.419742778	5.222E-09
CYP2C8	0.419640062	0.0482008
SLC46A1	0.418861734	1.657E-05
SLC8A1	0.417848172	0.0007023
ENOX1	0.417333487	0.0001105
OASL	0.417083694	0.0001114
ATP13A5	0.416531505	0.0004042
KCNAB1	0.413431741	0.0052172
GSTM2	0.412274359	4.514E-11
ADCY2	0.412210203	0.0049389
CACNA1H	0.410303394	0.0020344
NAT1	0.410269963	4.91E-11
GALNT13	0.409199823	0.0018299
KCNT2	0.409014717	7.105E-05
TST	0.407898205	1.697E-14
HMOX1	0.407400257	7.935E-07
ENPP2	0.405965408	1.815E-12
PDE1A	0.403372349	1.115E-05
CHRNB4	0.40132276	0.0001972
SUCLG2	0.401269806	4.45E-19
SLC17A3	0.400806979	0.0039467
UST	0.399337637	6.797E-06
PHYHD1	0.399190738	7.728E-08
PGM1	0.396028436	6.311E-21
CPT2	0.395152609	1.475E-20
GPD1L	0.394861975	4.386E-20
CKB	0.39166954	3.387E-06
SLC6A11	0.390384564	7.072E-05
B3GALT4	0.388563839	8.976E-19
SLC7A3	0.386593116	1.035E-05
ACOX1	0.38629822	6.221E-11
SIAE	0.384293758	5.904E-16
CNGA1	0.383463083	4.79E-08
SLC20A1	0.381829398	1.125E-05
ST8SIA1	0.381498518	1.328E-05
NPR1	0.381117758	3.117E-08
SLC22A4	0.381075327	0.0011969
EPHX2	0.378927773	3.413E-12
KCNC2	0.378847626	0.0036715
CACNA1A	0.378379339	9.508E-07
ENPP1	0.377588902	1.415E-08
KCNK10	0.376948426	4.287E-12
SLC25A20	0.376910422	1.513E-19

DPYD	0.376874182	1.661E-12
B3GNT8	0.375648086	9.473E-13
AQP12B	0.37432649	5.091E-06
NUDT11	0.372759155	6.205E-05
GLTP	0.3694543	1.882E-27
AQP12A	0.369130546	1.336E-05
KLB	0.368599013	3.785E-06
ATP8B1	0.367781223	9.637E-19
PRDX6	0.367026417	3.067E-18
SLC3A1	0.365969062	0.0001223
HSD17B6	0.365607197	2.136E-05
IP6K3	0.365171628	9.81E-06
SLC41A2	0.364655273	2.389E-14
PIK3CG	0.363401131	9.251E-09
ACSF2	0.362948078	9.86E-12
SLC44A4	0.3625262	6.859E-17
ADH6	0.360740517	2.435E-12
GABRG3	0.360009526	0.000681
KCNJ16	0.359462382	0.0178314
SLC46A3	0.359403947	6.897E-11
CYP8B1	0.358634742	3.935E-06
AOX1	0.35835653	2.223E-07
PLCD4	0.357053341	9.683E-05
PLA2G2D	0.356750738	0.000775
CMBL	0.354678873	8.696E-16
SLC5A8	0.354438302	0.0043351
ABCB1	0.352591171	1.415E-06
PLCD3	0.351337286	2.731E-14
SLC2A13	0.351332229	1.152E-16
KL	0.350876984	2.273E-06
TCN2	0.350717479	1.009E-09
KCNB2	0.348094142	6.142E-10
CYP11A1	0.346433343	2.018E-07
CHRNA1	0.345357301	1.144E-07
SV2B	0.344912668	1.888E-07
SLC6A2	0.344582224	4.566E-05
SLC25A23	0.342702128	1.125E-21
ELOVL4	0.342505477	1.274E-07
SLC23A3	0.340995116	0.0015146
MOCS1	0.340609166	4.234E-12
HCN4	0.339784712	1.028E-06
GRIK2	0.338885698	7.897E-06
CLCNKA	0.338184875	0.0007315
ACACB	0.335972085	4.296E-14

PLCG2	0.335897118	1.761E-08
SLC22A17	0.335704785	2.122E-09
EXTL1	0.333047199	9.48E-08
PDE5A	0.330623608	4.138E-07
SLC22A5	0.326280004	8.85E-13
SLC1A1	0.32612675	2.354E-07
GRIA2	0.324956264	0.0007263
ADH1A	0.322512492	0.0004138
ATP8A2	0.322153264	4.527E-09
ENTPD8	0.321741925	2.94E-08
CACNG6	0.321196125	1.648E-05
ALAS2	0.319977089	0.003054
FMO4	0.319252496	1.831E-14
ACAA2	0.317762941	4.114E-17
SCN4A	0.317019465	0.0003598
AQP7	0.315690966	1.882E-08
FUCA1	0.314723479	3.163E-19
FMO2	0.313033966	0.0003889
CDO1	0.312228436	2E-06
CHRNA3	0.311064206	5.414E-07
CACNA2D1	0.30974929	0.0001272
SMPD1	0.309702269	2.574E-18
MCOLN2	0.30949569	5.418E-09
SLC36A1	0.308380347	1.081E-12
KCTD4	0.30654623	0.0089619
HS6ST3	0.3062833	0.0003054
DPEP3	0.304122232	2.379E-06
NAT2	0.304029589	8.268E-17
AKR1C2	0.30301279	5.444E-13
PIGZ	0.302890984	9.897E-12
PAPSS2	0.30232028	7.387E-16
RETSAT	0.302291689	3.321E-17
SDR9C7	0.301851612	0.0035019
CKM	0.301505457	0.0034218
CYBRD1	0.301076839	0.0001786
GLYAT	0.29891341	0.019335
ABCD2	0.298499361	3.095E-10
ACSM2B	0.297653003	0.0009686
NEU4	0.29746085	3.65E-12
KCNC1	0.296154425	1.494E-08
ST8SIA6	0.294827938	2.247E-09
NUDT10	0.294425568	8.04E-07
DPYSL5	0.293491924	0.000597
GLB1L3	0.293184821	0.0134666

SCN4B	0.292606961	6.434E-16
GRIN3A	0.290559171	3.137E-06
MAOA	0.290225341	5.045E-17
FOLR2	0.28960708	5.379E-08
FLVCR2	0.28914992	2.389E-10
PDE3A	0.289067768	9.149E-13
P2RX1	0.288869311	5.225E-15
GCNT4	0.287910373	0.0006123
UGDH	0.28762852	1.508E-18
PDE7B	0.287321753	3.46E-15
SLCO2A1	0.28562266	3.628E-13
GGT6	0.285373222	4.682E-18
SLC37A2	0.283246585	0.0307702
ETFDH	0.280433244	3.808E-27
KCNA3	0.28003343	1.995E-10
GPX3	0.279849328	3.159E-09
ABCA6	0.27896324	3.586E-07
UGT3A2	0.277450607	1.407E-07
KCNMB2	0.276129403	0.0001352
KCNG3	0.274280531	2.834E-10
CLIC5	0.273446518	1.546E-11
ENTPD3	0.272035215	9.016E-09
RDH5	0.269958465	1.499E-23
KCNG1	0.269543256	3.421E-09
ABHD3	0.268720133	4.341E-16
SLC8A3	0.266970082	1.114E-12
RYR3	0.265039499	0.0002098
SLC9A9	0.264115225	1.33E-16
SLC28A2	0.263349174	0.0127353
PLCD1	0.263017759	2.155E-15
GRIA3	0.262848922	1.307E-11
CES2	0.261091235	1.366E-16
ACADL	0.260457819	5.542E-06
NAAA	0.259170992	9.694E-17
ATP2B2	0.256099545	4.458E-09
BHMT2	0.255928272	9.689E-08
KCNN3	0.254280026	4.624E-06
FMO5	0.254245079	5.722E-16
ATP13A4	0.253770354	7.002E-10
GDPD3	0.253296804	2.21E-13
PLCE1	0.252913399	2.372E-19
SLC4A1	0.252147958	7.333E-05
CHRNA4	0.249513311	6.304E-05
ACSM2A	0.249378355	1.084E-06

PLCL2	0.248438733	4.339E-17
SCN3A	0.246650728	9.342E-15
SLC35F1	0.246230194	2.755E-07
SULT1A1	0.244991734	6.554E-16
ACADS	0.244547734	1.171E-19
CA12	0.242293193	6.376E-18
SLC13A2	0.241731255	3.993E-06
GRIA1	0.241405413	0.0006238
ATP6V0D2	0.241341896	2.12E-09
CHST5	0.241080052	9.014E-10
CES3	0.240924561	1.458E-16
SCN3B	0.239526661	2.948E-12
MAOB	0.238773789	0.0013056
GABRA1	0.238238936	9.032E-05
KCNS2	0.234288504	2E-05
SLC9A2	0.234179369	3.11E-21
DHRS11	0.232546624	1.409E-18
UGP2	0.232506204	3.375E-21
CA14	0.232117577	7.674E-05
SLC30A8	0.230228233	2.137E-09
SLC22A18AS	0.229906567	8.329E-19
TRPC7	0.229424055	4.116E-14
CYP2C18	0.228331924	0.0005817
GCNT3	0.228047482	4.259E-15
SLC38A4	0.227195973	1.666E-07
ATP2B4	0.227195714	0.0001011
HSD11B2	0.226018718	1.344E-18
ASPG	0.224765839	8.853E-06
MSRB3	0.223748571	2.321E-05
SLC23A1	0.221553837	0.0309108
CHST8	0.221425895	4.184E-09
UGT1A9	0.216027735	2.531E-07
ATP1B2	0.207951712	8.094E-13
PLA2G5	0.205781054	6.8E-06
LIPI	0.201654448	0.0012207
SLC32A1	0.199510611	0.0004981
ATP6V1G2	0.196635145	8.038E-10
PDE2A	0.196587595	4.435E-09
HMGCS2	0.196010362	8.24E-12
KCNQ5	0.191962559	3.601E-10
PDE9A	0.189209435	2.578E-12
UGT2B15	0.189189493	3.316E-09
SULT1B1	0.187751283	7.612E-18
AOC3	0.187542543	4.409E-05

GLRA4	0.186592177	1.868E-11
XDH	0.185823497	1.585E-09
KCNA5	0.183981808	7.425E-06
CYP4B1	0.180156367	4.92E-05
ENTPD5	0.178038773	1.139E-19
ST6GALNAC6	0.177524527	1.848E-12
SMPDL3A	0.177183962	1.771E-19
LIPC	0.176632468	2.96E-11
PTGS1	0.175397625	2.01E-06
SLC10A4	0.171486884	6.919E-10
ADHFE1	0.169062114	6.126E-16
GRIA4	0.167316157	2.424E-06
GRIN2A	0.167273233	2.392E-09
SLC16A9	0.167230403	6.68E-10
ABCA9	0.167019897	1.545E-07
GAL3ST3	0.166469044	0.0001233
SLC16A12	0.166124151	2.58E-09
AHCYL2	0.165736772	1.578E-18
MOGAT2	0.16328871	9.777E-16
SLC17A1	0.162914707	4.123E-10
KCNMA1	0.161928823	2.389E-06
CP	0.161311244	1.187E-15
SLC39A12	0.161267905	1.194E-09
ACSM5	0.160826664	1.082E-09
GPT	0.160474774	1.288E-19
GSTM5	0.159582177	9.647E-16
SLC2A4	0.156318944	4.094E-05
SLC8A2	0.156127273	0.0011594
CA10	0.150556287	3.587E-16
SLC5A11	0.148495043	0.031012
ADCY5	0.148447401	2.043E-11
SLC18A3	0.148023035	1.402E-05
GRIK5	0.147261138	3.402E-06
HPGD	0.142985008	1.689E-18
HTR3A	0.140898415	2.711E-09
TRPV3	0.140736998	4.051E-09
B3GNT7	0.140503749	1.412E-08
LDHD	0.1388464	4.811E-19
SLC17A7	0.138270782	1.484E-17
KCTD8	0.138176391	0.0002961
B3GALT5	0.138012927	2.411E-12
HMGCLL1	0.137813149	3.132E-16
KCNK3	0.137164221	4.639E-06
SCN9A	0.134583506	4.275E-17

PLD5	0.131547597	5.794E-07
KCNMB1	0.128760563	5.541E-05
UGT1A1	0.128478565	0.0003022
HTR3C	0.126837542	5.148E-10
PLA2G2C	0.125887552	3.592E-05
CACNG7	0.125296803	1.042E-10
PCK1	0.124025443	6.467E-10
KCNE2	0.122772115	3.049E-14
NAALADL1	0.122724331	0.0180159
SLC27A6	0.120763468	7.761E-15
PGM5	0.118409991	2.715E-06
UGT2A3	0.118275752	3.883E-17
HSD17B2	0.118092247	3.189E-13
ABCB5	0.116855311	1.235E-06
GDPD2	0.116212739	5.251E-13
HPSE2	0.113728328	4.612E-18
KCNA4	0.111389016	3.259E-07
TPH1	0.111183368	6.488E-06
ENPP6	0.110405995	5.115E-21
NOS1	0.109227139	0.0006691
KCNA1	0.109084942	0.0014867
KCNH5	0.108112012	0.0002013
HTR3E	0.107529763	4.288E-10
ATP2B3	0.107462681	5.263E-07
GABRG1	0.106930722	1.292E-07
BEST2	0.106901031	8.625E-13
B3GALT1	0.106804038	1.021E-19
PIK3C2G	0.105394671	0.0001116
AADACL4	0.103130963	1.797E-05
SLC17A4	0.103113873	1.731E-13
AQP4	0.101281293	1.187E-06
SCN11A	0.100860559	2.334E-11
SCN2B	0.100397987	4.57E-08
AKR1B10	0.094886154	3.081E-17
PLCXD3	0.092955798	1.023E-10
DHRS9	0.092251107	5.86E-17
ASPA	0.091744127	3.34E-11
AMPD1	0.088199089	1.007E-12
AADACL2	0.087982484	9.654E-11
GCNT2	0.087484661	4.582E-22
SLCO4C1	0.08717563	2.007E-09
UGT2B17	0.086525022	5.488E-12
B4GALNT2	0.086123422	1.355E-09
CLCNKB	0.085972614	0.0028549

UGT1A10	0.085291327	1.109E-16
ADH1C	0.084771938	5.825E-17
SLC4A10	0.084588696	2.192E-08
CACNG5	0.08453849	2.249E-06
MGAT4C	0.081977077	7.897E-10
GRIK3	0.081663905	2.659E-07
PDE6A	0.080735286	1.724E-16
LRAT	0.079990138	0.0374246
PYGM	0.078205913	0.0001095
SLC6A15	0.077460631	8.565E-12
SLC9A3	0.075360078	1.218E-06
ST8SIA3	0.072269823	5.706E-09
SULT1A2	0.071421977	1.767E-13
GABRG2	0.070864911	5.222E-09
ABCA8	0.070675849	4.584E-20
SLC25A34	0.069444307	2.151E-16
ADH1B	0.067336924	4.19E-14
CNGB1	0.065237211	2.147E-08
KCNB1	0.065034849	7.968E-06
SLC7A14	0.064911487	1.951E-08
RHAG	0.064862349	1.91E-05
TAT	0.060247935	8.943E-10
SCNN1B	0.059861734	3.436E-13
TRPM6	0.057054385	1.897E-12
SLC26A2	0.05594286	1.084E-14
HTR3B	0.055398712	9.05E-08
SLC5A7	0.055159033	1.235E-06
CHST9	0.054923661	1.145E-08
P2RX2	0.054550912	2.404E-10
UGT1A8	0.053458621	1.324E-14
BCHE	0.051483617	1.224E-06
CA2	0.050522417	1.16E-20
HS3ST6	0.049643102	1.735E-05
FUT9	0.046254879	4.184E-05
SLC6A19	0.042710944	0.0274349
SCN7A	0.042262725	9.924E-06
ATP1A2	0.042219881	3.017E-05
CHAT	0.041754627	9.238E-07
SLC4A4	0.041598844	6.949E-21
ABCG2	0.040680026	7.759E-08
SLC26A3	0.039242114	6.561E-15
CA4	0.035833527	2.236E-15
SCNN1G	0.034563162	0.0001499
SLC30A10	0.033828806	2.079E-08

ABCB11	0.029934918	5.9E-06
SLC17A8	0.02797693	0.000151
PNLIPRP3	0.027188818	7.552E-05
DHRS7C	0.02598302	9.427E-10
CLCA4	0.025495825	1.359E-10
HSD3B2	0.024761449	0.0015446
DAO	0.019526476	4.435E-10
BEST4	0.018116525	1.72E-15
CA7	0.014691335	3.651E-18
CA1	0.012121678	6.845E-14
AQP8	0.008144078	2.933E-08
SLC7A13	0	0.0256936

Analysis of 2752 metabolism-related genes in GSE25070			
Probe	Gene.symbol	Normal/Tumor	P Value
ILMN_1713462	AQP8	44.06933465	5.04E-16
ILMN_1652431	CA1	36.11077179	3.4E-14
ILMN_1679176	CLCA4	31.62965358	1.62E-13
ILMN_1763749	GUCA2A	26.96468733	2.68E-15
ILMN_1735578	GUCA2B	24.92477766	1.7E-22
ILMN_1695157	CA4	18.54855352	1.17E-12
ILMN_1731529	MS4A12	17.95581161	1.05E-11
ILMN_1760087	SLC26A3	16.19500131	6.2E-10
ILMN_2165993	ITLN1	12.03242996	0.000000446
ILMN_1797219	CLCA1	12.01079199	5.02E-08
ILMN_1752214	UGT2B17	11.79132182	6.71E-08
ILMN_1764309	ADH1A	11.75159429	6.21E-14
ILMN_1740717	ADH1C	11.40738923	1.37E-10
ILMN_1669410	CHGA	11.40222057	3.59E-11
ILMN_1771467	TMIGD1	10.79131453	3.63E-18
ILMN_2199439	CA2	10.29644602	5.01E-11
ILMN_1679194	UGT2B7	10.29196399	3.44E-09
ILMN_1813957	CEACAM7	10.21033531	1.92E-09
ILMN_1672148	AKR1B10	10.14816038	3.47E-11
ILMN_1815203	HMGCS2	9.80951664	1.59E-09
ILMN_1755897	UGT2B7	9.460214595	7.26E-09
ILMN_1695631	CHP2	8.915584543	5.28E-14
ILMN_1806386	NXPE4	8.454903391	7.96E-09
ILMN_1733998	DHRS9	8.305791464	8.62E-10
ILMN_1657435	MT1M	8.128850188	3.35E-12
ILMN_2072568	CLDN8	8.009377396	3.13E-08
ILMN_1777190	CFD	7.91584158	1.26E-17
ILMN_1666536	VSIG2	7.850961661	4.15E-10

ILMN_1808713	HSD17B2	7.781234779	6.59E-14
ILMN_1724396	GCG	7.409109523	2.55E-11
ILMN_2373670	UGT2B17	7.312147487	2.99E-09
ILMN_1703075	PYY	7.206259976	3.11E-13
ILMN_1679391	MAMDC2	7.045446016	3.16E-14
ILMN_2337263	PKIB	6.760776056	4.07E-16
ILMN_2149164	SFRP1	6.684432257	5.23E-16
ILMN_1794638	VIP	6.640591992	2.5E-10
ILMN_2384181	DHRS9	6.41986233	1.37E-10
ILMN_1787526	C2orf88	6.316288637	2.3E-14
ILMN_1750312	SLC51B	6.30474268	6.78E-14
ILMN_1751062	SCARA5	6.298370953	6.91E-24
ILMN_1659984	MEP1A	6.275831248	0.000000188
ILMN_1813350	HSD11B2	6.082479868	5.26E-12
ILMN_1715401	MT1G	6.080624707	0.000000041
ILMN_1718984	FCGBP	6.043116606	4.56E-10
ILMN_1791447	CXCL12	5.943106318	2.26E-15
ILMN_1755796	BEST2	5.7550754	1.18E-12
ILMN_1766264	PI16	5.715153668	4.65E-18
ILMN_1812824	SST	5.609475416	7.41E-12
ILMN_1802192	C10orf99	5.565058642	0.000000395
ILMN_2124802	MT1H	5.547481841	9.01E-09
ILMN_1682176	CLEC3B	5.526861462	1.47E-18
ILMN_1660086	MYH11	5.397986393	0.000000139
ILMN_2181064	GBA3	5.392029539	4.89E-09
ILMN_2302757	FCGBP	5.391606212	0.00000258
ILMN_1740917	SCNN1B	5.373861093	2.57E-12
ILMN_2093343	PLAC8	5.359289499	8.91E-08
ILMN_2166457	HPGD	5.351307937	1.21E-16
ILMN_1796685	BEST4	5.322810481	9.44E-18
ILMN_2379920	NXPE4	5.277226077	3.1E-09
ILMN_2103107	ADAMDEC1	5.194065546	7.48E-12
ILMN_2278984	NXPE4	5.16195552	5.27E-09
ILMN_1746888	PCOLCE2	5.114988655	6.9E-18
ILMN_1680652	SELENBP1	5.07487019	1.99E-11
ILMN_2078592	ADTRP	5.069590053	1.06E-10
ILMN_2180885	CWH43	4.982366295	3.83E-14
ILMN_2174437	CIDEC	4.952098428	2.07E-10
ILMN_1814296	TRPM6	4.850600457	6.78E-09
ILMN_1712082	GCNT3	4.849598194	0.000000432
ILMN_1740233	UGT1A10	4.847320986	3.45E-13
ILMN_2137789	KLF4	4.841207026	9.11E-14
ILMN_1725338	CLDN23	4.82757894	2.42E-11
ILMN_1709486	SRPX	4.822471091	2.67E-11

ILMN_2306540	PDE9A	4.684414636	7.2E-12
ILMN_1756701	DHRS11	4.678042634	1.35E-12
ILMN_1732410	SLC16A9	4.611893379	1.41E-11
ILMN_2357542	VIP	4.583618017	3.34E-13
ILMN_1804593	HEPACAM2	4.548829531	2.16E-08
ILMN_1721283	HSPB6	4.542436038	0.000000246
ILMN_1663640	MAOA	4.524493886	6.05E-12
ILMN_1776905	LOC340843	4.503636723	3.56E-11
ILMN_1709590	PGM5	4.484417128	0.00000383
ILMN_1790859	PLAC9	4.463958701	1.8E-16
ILMN_1781388	PGM5	4.448333489	0.00000351
ILMN_1795257	GPT	4.443060604	1.64E-11
ILMN_2173611	MT1E	4.440915098	1.01E-08
ILMN_1813561	SCIN	4.434475021	1.16E-11
ILMN_1761789	CA7	4.360657709	3.89E-18
ILMN_2120695	TSPAN7	4.352055546	3.71E-18
ILMN_1729170	GPA33	4.346901938	0.000000304
ILMN_1811598	ADH1B	4.346492243	6.44E-23
ILMN_1805842	FHL1	4.337183972	7.08E-09
ILMN_1684982	PDK4	4.232558563	3.72E-12
ILMN_1723678	PRPH	4.228059297	5.86E-14
ILMN_1708107	DPT	4.206647642	1.02E-09
ILMN_1705107	SDCBP2	4.187645884	1.65E-10
ILMN_1670385	TUBAL3	4.175002785	1.53E-08
ILMN_1656395	MYOT	4.164427915	9.13E-15
ILMN_1712786	AHCYL2	4.161942836	2.81E-11
ILMN_1676822	C2orf40	4.151868556	2.72E-10
ILMN_2210934	NR3C2	4.116589664	3.72E-16
ILMN_2278335	AKR1B15	4.058839409	1.34E-12
ILMN_2366041	ITM2C	4.032823818	5.31E-11
ILMN_1744293	MUCDHL	3.979544586	1.8E-09
ILMN_1769219	TPSG1	3.969338971	7.2E-09
ILMN_1805410	C15orf48	3.964343875	0.000000371
ILMN_1680424	CTSG	3.949838621	2.82E-09
ILMN_1694840	MATN2	3.915569865	1.41E-13
ILMN_1703205	EDN3	3.877030219	1.29E-11
ILMN_1720998	CA12	3.866947721	0.00000017
ILMN_1704861	MYO1A	3.852641273	1.05E-08
ILMN_2358886	EDN3	3.817563065	3.21E-11
ILMN_2063168	MALL	3.798939483	4.2E-10
ILMN_1700310	SDCBP2	3.797434841	1.03E-10
ILMN_1762561	PLA2G10	3.789220251	0.00000332
ILMN_1813295	LMO3	3.776965197	0.00000476
ILMN_1719250	SLC44A4	3.75132394	0.000000136

ILMN_1726666	GPX3	3.748229109	2.17E-11
ILMN_1743445	FAM107A	3.744748372	2.76E-14
ILMN_1665884	REP15	3.723517453	7.69E-09
ILMN_2219683	NEU4	3.718304524	2.5E-09
ILMN_2176592	BCHE	3.712926046	8.41E-08
ILMN_1730977	SLC44A4	3.696380005	9.74E-08
ILMN_1733415	MFAP5	3.686816775	2.04E-09
ILMN_1732398	SCGB2A1	3.660482963	0.00000043
ILMN_1680453	ITM2C	3.650344641	2.11E-10
ILMN_1733042	BCAS1	3.625520522	1.1E-09
ILMN_1687848	C7	3.588113981	5.5E-11
ILMN_1789648	SCGN	3.561313078	9.82E-20
ILMN_1696675	CES2	3.560057591	4.52E-09
ILMN_2407346	LDHD	3.492451159	1.62E-12
ILMN_1814221	NPTX1	3.490606937	0.00000404
ILMN_1715612	OTOP2	3.429897754	8.8E-15
ILMN_1654696	C15orf48	3.385853261	2.87E-08
ILMN_2327860	MAL	3.35403176	1.27E-12
ILMN_1720461	SLC17A4	3.328293245	2.87E-11
ILMN_2382942	CA12	3.302853661	2.13E-08
ILMN_2388263	HEPACAM2	3.298527608	5.29E-08
ILMN_1709634	CMBL	3.285449257	6.71E-08
ILMN_2399016	MMP28	3.281173359	1.07E-11
ILMN_1788942	GGT6	3.278986632	7.91E-08
ILMN_2232463	ARL14	3.234668394	8.95E-10
ILMN_1735438	GPM6B	3.220922929	1.34E-11
ILMN_2322996	EYA2	3.211340405	0.000000081
ILMN_1728009	TMEM171	3.192661367	2.36E-09
ILMN_2065022	ARHGAP44	3.183180442	3.22E-10
ILMN_2412336	AKR1C2	3.180990603	0.000000605
ILMN_2049184	DNASE1L3	3.158084383	0.000000109
ILMN_1809456	CNTFR	3.157037506	3.38E-13
ILMN_1680344	MYOM1	3.151829492	0.000000639
ILMN_1704665	GPM6B	3.14270122	3.77E-11
ILMN_1740479	FABP2	3.128966169	7.56E-10
ILMN_2210753	LYVE1	3.115541513	2.63E-11
ILMN_1691048	SLC22A18AS	3.100818665	4.99E-09
ILMN_1661309	XKR4	3.09786398	3.29E-15
ILMN_2045849	SI	3.096452146	0.000000881
ILMN_1808249	MS4A8	3.092600459	0.00000402
ILMN_2387865	KCNIP4	3.090417345	1.22E-12
ILMN_2410929	PAPSS2	3.089082304	1.04E-09
ILMN_1729563	UGDH	3.083283526	4.47E-13
ILMN_1664330	CEACAM1	3.081956801	3.23E-08

ILMN_1740949	LRRC19	3.080970374	2.25E-08
ILMN_1748840	CALB2	3.078579042	0.00000173
ILMN_2376822	SDCBP2	3.067735885	1.14E-09
ILMN_1794875	GPAT3	3.055461749	5.42E-13
ILMN_1672264	NXPE2	3.047609188	0.000000099
ILMN_2136089	MTE	3.041179919	4.6E-09
ILMN_1790098	OGN	3.035103259	1.85E-12
ILMN_1726928	TCEA3	3.013098466	0.00000012
ILMN_1670672	TMEM37	3.010921448	3.33E-11
ILMN_2199389	VIPR1	3.006649217	9.12E-10
ILMN_1678816	GREM2	3.005481355	1.91E-19
ILMN_1673639	ABI3BP	3.004368319	1.9E-14
ILMN_2048414	UGT2B15	2.995585746	0.00000132
ILMN_1791123	TMPRSS2	2.967471759	0.00000176
ILMN_2297765	KCNMA1	2.964809152	0.00000459
ILMN_2339835	PTGS1	2.948471318	0.000000759
ILMN_1689111	CXCL12	2.946665566	9.53E-12
ILMN_1747546	TSPAN1	2.933702097	0.00000157
ILMN_2305407	ZBTB16	2.925522127	0.00000161
ILMN_1677108	CAPN13	2.92307906	0.000000235
ILMN_1696657	LRRN2	2.899835435	2.89E-09
ILMN_1709399	SLC25A34	2.89484088	2.67E-13
ILMN_1770663	KRT24	2.892108946	1.2E-14
ILMN_2197381	PCK1	2.890019145	4.55E-09
ILMN_1722869	PDZD3	2.884484098	2.09E-09
ILMN_1702487	SGK1	2.883281107	2.79E-08
ILMN_2387105	OGN	2.872650702	8.6E-13
ILMN_1696434	LAMA1	2.851339783	1.54E-08
ILMN_1796094	CD36	2.846701811	2.51E-10
ILMN_1669362	IGFBP6	2.84012847	0.000000108
ILMN_1795104	ACADS	2.833509914	1.19E-13
ILMN_1801043	GSN	2.831825151	1.19E-08
ILMN_1773006	FABP4	2.824850505	2.11E-09
ILMN_1701441	LPAR1	2.812800028	5.16E-15
ILMN_1657373	P3H2	2.809084378	0.000000181
ILMN_1809384	CYP4F12	2.805369397	0.000000481
ILMN_2089752	FAM150B	2.803106289	4.7E-14
ILMN_1665372	FXYD3	2.802723396	0.000000442
ILMN_1738849	SLC9A2	2.799044843	2.34E-10
ILMN_1808258	HTR4	2.796494669	1.27E-13
ILMN_2126038	STMN2	2.790941955	5.57E-12
ILMN_1761946	PROM2	2.784511826	0.000000265
ILMN_1772131	IL1R2	2.779464527	0.000000802
ILMN_2370241	TEX11	2.775088094	6.48E-11

ILMN_1745849	ENTPD5	2.764316967	4.73E-14
ILMN_2109197	EPB41L3	2.762123296	3.46E-11
ILMN_1774901	GDPD3	2.760129276	6.92E-10
ILMN_2171481	NXPE1	2.757021848	6.65E-08
ILMN_1769394	PLCD1	2.74910216	4.73E-14
ILMN_1691156	MT1A	2.723295736	8.16E-08
ILMN_2161330	SPDEF	2.721796019	0.000000127
ILMN_1674817	C1orf115	2.717515775	1.93E-10
ILMN_2189675	HRCT1	2.713640901	9.81E-10
ILMN_1736184	GSTM3	2.713270755	0.000000014
ILMN_1811303	NR5A2	2.707145989	3.96E-14
ILMN_1776516	ITPKA	2.702218435	0.000000329
ILMN_2366654	NR5A2	2.700643914	3.46E-12
ILMN_2281502	DHRS9	2.697524887	5.55E-11
ILMN_1768016	TNFRSF17	2.693491271	0.000000198
ILMN_2083567	PHLPP2	2.688005053	3E-13
ILMN_2391512	NAAA	2.687593582	2.61E-13
ILMN_2234697	BEX1	2.682668927	0.000000194
ILMN_2376723	CDKN2B	2.682621176	2.78E-11
ILMN_1705116	SLC22A23	2.667382961	4.75E-11
ILMN_2219867	KRT20	2.657198748	5.16E-10
ILMN_1741465	PTPRH	2.639367386	8.65E-10
ILMN_2120210	RCAN2	2.63688174	1.97E-11
ILMN_1680081	B3GNT6	2.633304781	4.29E-08
ILMN_1778964	CLIC5	2.631250131	0.00000344
ILMN_2230016	HIGD1A	2.629734024	2.56E-11
ILMN_1784447	PLCE1	2.627492216	1.19E-12
ILMN_2390310	MGC14376	2.624985493	5.81E-12
ILMN_2416019	ENTPD8	2.621686284	6.93E-09
ILMN_1666503	DENND2A	2.61341448	1.21E-08
ILMN_1691736	ST6GALNAC6	2.606746137	0.000000673
ILMN_2201580	GSTM2	2.598383957	0.00000159
ILMN_1722718	BMP2	2.595265759	1.45E-10
ILMN_1717163	F13A1	2.582545917	0.00000302
ILMN_2059689	TMEM54	2.5806351	0.000000375
ILMN_1766914	MFAP4	2.577926283	0.00000249
ILMN_2061537	UGT1A8	2.57428858	2.61E-12
ILMN_1702633	RETSAT	2.572066066	6.61E-14
ILMN_1656560	PARM1	2.571815146	0.000000687
ILMN_1773395	RDH5	2.567676572	1.5E-12
ILMN_1752728	FUCA1	2.564942421	3.88E-13
ILMN_1661799	LOC643940	2.564426851	2.75E-13
ILMN_1683063	PDE9A	2.559517943	9.45E-14
ILMN_1750062	PPARGC1A	2.558022741	0.000000123

ILMN_2112474	PAQR5	2.542336648	9.99E-13
ILMN_2400219	SRI	2.528822611	5.47E-12
ILMN_1737089	CAPN5	2.528657498	0.000000419
ILMN_1698259	TMEM100	2.527417552	1.28E-14
ILMN_1801491	SLC26A2	2.525875569	0.000000133
ILMN_1801616	EMP1	2.518303703	0.000000018
ILMN_1715991	SDPR	2.510369571	0.000000377
ILMN_1759312	AMPD1	2.49998904	1.89E-11
ILMN_1737076	HHLA2	2.489898806	2.64E-12
ILMN_1739161	PLPP1	2.488416039	1.04E-13
ILMN_1811313	SLIT3	2.486248034	1.35E-08
ILMN_1670903	NAT2	2.482509933	0.00000012
ILMN_1717636	RGMA	2.482022029	0.00000114
ILMN_1791580	FXYD3	2.476818358	0.00000023
ILMN_1777411	ATP1A2	2.473333563	3.17E-08
ILMN_1762531	FGF9	2.471842995	4.65E-08
ILMN_1733627	NEDD4L	2.466576854	3.68E-15
ILMN_1656285	METTL7A	2.454883005	1.17E-09
ILMN_2343278	PLPP1	2.44308744	2.9E-11
ILMN_1810836	PDE5A	2.442267472	0.00000119
ILMN_2361603	NDRG2	2.434309441	8.5E-13
ILMN_1775170	MT1X	2.421367803	0.00000278
ILMN_2362681	CES2	2.417111139	9.98E-08
ILMN_1775814	GHR	2.407184146	5.89E-09
ILMN_2062714	PTGDR	2.404401958	7.37E-09
ILMN_1666109	MB	2.39668803	1.01E-10
ILMN_1728570	TCF21	2.391464259	2.46E-12
ILMN_1678478	CHST5	2.390451956	2.36E-08
ILMN_1802053	ZNF91	2.389095659	5.82E-08
ILMN_2143314	SPIB	2.385340432	1.98E-12
ILMN_2224907	SMIM14	2.382227507	1.9E-10
ILMN_2391861	GSTM1	2.379454933	0.000000058
ILMN_1687306	LGALS2	2.373419042	0.000000432
ILMN_1657708	MGLL	2.366975889	1.04E-11
ILMN_1784863	CD36	2.366466829	4.99E-08
ILMN_1668605	NAAA	2.365795087	4.61E-12
ILMN_1677038	CPED1	2.362975613	0.00000183
ILMN_1802397	GNA11	2.3590148	2.09E-11
ILMN_2389151	UGP2	2.354848995	4.71E-15
ILMN_1740706	PMP2	2.341572001	3.61E-13
ILMN_1724805	MUCDHL	2.338291317	2.79E-10
ILMN_1660446	BTNL3	2.337860732	0.000000465
ILMN_1788267	PPP1R14D	2.334677941	0.000000222
ILMN_1712613	PNLIPRP2	2.328679609	0.00000115

ILMN_2339294	LILRB5	2.32811635	1.43E-08
ILMN_2153916	HSPA2	2.318115419	0.000000938
ILMN_2171023	LGI1	2.314817667	2.32E-20
ILMN_1763852	ACACB	2.30476018	2.55E-09
ILMN_1796089	NKX2-3	2.302685928	2.87E-11
ILMN_2167758	CILP	2.301753053	0.00000228
ILMN_1733690	AKAP7	2.298800407	3.04E-11
ILMN_1799015	PXMP2	2.295449241	5.05E-08
ILMN_1671891	PID1	2.293883066	5.93E-10
ILMN_1660114	MMRN1	2.292939898	5.45E-11
ILMN_1784967	EPB41L4B	2.292775184	1.81E-09
ILMN_2336609	SYTL2	2.289616367	0.00000325
ILMN_1766712	TCF21	2.289040613	7.43E-11
ILMN_1778523	KLF9	2.287131267	0.000000256
ILMN_1789641	ABCG2	2.285750792	3.08E-12
ILMN_2212999	KIF5C	2.28395746	2.05E-12
ILMN_2381257	DSC2	2.275544753	0.000000112
ILMN_1689046	RBM47	2.272793074	4.05E-08
ILMN_1703572	PCDH20	2.271576733	0.000000645
ILMN_2112638	SVEP1	2.269597741	9.78E-08
ILMN_1704369	LIMA1	2.2685968	8.24E-11
ILMN_1730645	TMEFF2	2.267269095	2.78E-16
ILMN_1703178	SCG2	2.266070758	1.33E-08
ILMN_2304404	PBLD	2.264635857	9.65E-09
ILMN_1766405	GOLM1	2.260079043	0.00000127
ILMN_2405602	OSBPL1A	2.256930683	8.55E-10
ILMN_1697925	APOBR	2.255592936	1.98E-09
ILMN_1810716	GFRA3	2.252514729	6.34E-10
ILMN_1808157	RUNDC3B	2.251726243	4.56E-17
ILMN_2389155	UGP2	2.250914894	2.48E-12
ILMN_1681634	PXMP2	2.247444641	1.21E-08
ILMN_1676296	PLPP1	2.24256792	1.5E-14
ILMN_1699357	SLC22A5	2.241497795	4.9E-09
ILMN_1680390	GCNT2	2.239828171	6.41E-12
ILMN_2384056	GPER1	2.235950035	4.61E-08
ILMN_1802780	CD163L1	2.229621501	0.000000116
ILMN_1811632	FMO5	2.22693514	0.00000138
ILMN_1737025	PLCL2	2.226309872	3.86E-11
ILMN_1758034	ETFDH	2.226034419	9.69E-14
ILMN_1801767	ABHD3	2.225027828	7.05E-08
ILMN_2226324	MPC1	2.200728861	1.33E-11
ILMN_1727150	DHRS9	2.198375011	4.69E-14
ILMN_1717381	HOXD1	2.19688311	5.75E-09
ILMN_1678579	CPT2	2.196554811	2.94E-11

ILMN_1759075	TNFRSF13B	2.195701916	4.25E-13
ILMN_1715463	SOSTDC1	2.192021274	0.000000138
ILMN_1720513	SETBP1	2.189053919	6.42E-09
ILMN_1667199	SQRDL	2.185669266	7.75E-08
ILMN_1795838	C4orf19	2.174852636	8.65E-08
ILMN_2273911	ACSL5	2.173538213	0.0000047
ILMN_1756502	RAXLX	2.170896522	1.54E-10
ILMN_1775235	AFF3	2.168501017	1.67E-14
ILMN_1778650	VILL	2.168123369	0.000000169
ILMN_1803256	STOX2	2.164690443	9.62E-12
ILMN_1784287	TGFBR3	2.15653313	0.000000753
ILMN_2400372	SULT1A2	2.149010095	1.39E-08
ILMN_1698685	MATN2	2.148717219	8.07E-16
ILMN_1795298	GPR30	2.143182179	5.42E-08
ILMN_1693941	IGSF9	2.139304612	0.000000137
ILMN_1694106	GPD1L	2.138701947	7.09E-11
ILMN_1804798	BEXL1	2.136915662	0.00000148
ILMN_1714567	AHNAK	2.126913159	8.16E-10
ILMN_1712632	XDH	2.12338558	0.000000693
ILMN_1731062	NPY	2.12034617	0.00000143
ILMN_1686906	TP53INP2	2.117979947	3.95E-12
ILMN_1679719	FLJ32063	2.11561962	0.000000834
ILMN_1705231	SLCO2A1	2.113215695	1.33E-08
ILMN_1759330	KIF1A	2.113134373	0.00000057
ILMN_2195703	PPARGC1B	2.109637059	2.22E-12
ILMN_1765076	APPL2	2.106120308	7.08E-08
ILMN_1722798	PLCD3	2.104867194	9.44E-12
ILMN_1764380	GLTP	2.101628733	7.45E-18
ILMN_1712430	ATP5G1	2.097721669	0.000000815
ILMN_1804737	RAVER2	2.089239565	1.87E-12
ILMN_1806533	PDE7B	2.088767478	1.22E-09
ILMN_1812461	WISP2	2.088140042	9.43E-13
ILMN_2184556	SLC4A4	2.087151232	6.88E-12
ILMN_1653429	SLC35A3	2.084371234	0.000000327
ILMN_1764607	UGT1A7	2.080676508	5.28E-12
ILMN_1730660	LGALS3	2.079064102	9.17E-08
ILMN_1663092	CITED2	2.07495306	1.8E-11
ILMN_1677292	C5orf30	2.070893426	5.58E-09
ILMN_1748983	RTN4	2.07015593	7.03E-11
ILMN_2373763	CASP7	2.068948938	0.000000305
ILMN_1769433	IQGAP2	2.066076166	0.000000693
ILMN_1665219	LTBP4	2.065731589	0.000000066
ILMN_2310909	ATP2A3	2.059127723	5.73E-11
ILMN_1664922	FLNB	2.056786834	0.000000204

ILMN_1783672	SLC30A10	2.055250194	2.34E-09
ILMN_2396272	PDCD4	2.055241661	1.29E-08
ILMN_1709237	EPHX2	2.053599443	5.25E-08
ILMN_2320330	MAL	2.052205579	9.68E-12
ILMN_2215824	ANKRD20A1	2.050793187	1.09E-08
ILMN_1658356	PAMR1	2.047833683	0.000000302
ILMN_2225144	EIF4E3	2.045989796	1.27E-10
ILMN_1786396	ZZEF1	2.045019415	2.09E-12
ILMN_1652379	SUCLG2	2.041008782	0.000000051
ILMN_1800425	SLC9A1	2.03947101	6.97E-10
ILMN_1763638	BCAR3	2.038182631	5.7E-09
ILMN_2414135	PLP1	2.034127471	5.78E-14
ILMN_2351638	BEX4	2.033243497	0.000000218
ILMN_1763127	ACKR2	2.027798682	3.85E-16
ILMN_2341067	NLGN4X	2.010562188	3.17E-09
ILMN_1703648	TMEM82	2.009457103	3.77E-09
ILMN_2285568	NAAA	2.00534931	3.14E-12
ILMN_2402341	MAPK3	2.005242741	7.51E-10
ILMN_1717046	MOB3B	2.004602045	1.13E-09
ILMN_1660413	SGK2	1.999483617	3.17E-10
ILMN_1804419	LRMP	1.995049068	0.00000251
ILMN_2384536	ECI2	1.992141522	1.12E-11
ILMN_1731783	ATP1A1	1.983362509	0.000000265
ILMN_1798081	PTPRF	1.982707034	1.9E-09
ILMN_2246956	BCL2	1.982175084	5.58E-13
ILMN_2404135	RIOK3	1.982137246	2.62E-09
ILMN_1795930	PTGER4	1.98110598	0.000000072
ILMN_1728107	GNG7	1.97868492	7.14E-15
ILMN_1689552	FAM63A	1.977936856	0.00000154
ILMN_1734229	SPPL2A	1.977829345	5.08E-11
ILMN_2077952	GALNT16	1.976786454	0.000000231
ILMN_1701551	ABCA6	1.97590524	8.74E-15
ILMN_2323801	MOCS1	1.97249788	1.53E-08
ILMN_1692824	ASPA	1.97116211	3.6E-14
ILMN_1702858	ADHFE1	1.970459053	2.63E-09
ILMN_1679949	SLC25A23	1.966683211	1.28E-10
ILMN_1761131	ECI2	1.966468259	0.00000019
ILMN_1718173	CDHR2	1.965059995	0.000000514
ILMN_1652631	GLIPR2	1.964834121	0.000000537
ILMN_1742390	FRMD1	1.963112675	6.71E-09
ILMN_2065690	GRAMD3	1.961531522	1.52E-12
ILMN_1717599	CNTN3	1.959488519	6.6E-10
ILMN_1673566	ADAMTS1	1.958423032	0.00000406
ILMN_1691076	TEX11	1.958122714	8.5E-11

ILMN_1668345	OAF	1.956120683	0.000000275
ILMN_2188521	NECTIN3	1.953741423	5.49E-08
ILMN_1762606	AQP11	1.951631751	0.00000147
ILMN_1724700	RIOK3	1.950158629	1.64E-09
ILMN_1740170	CHCHD10	1.94978711	0.000000372
ILMN_1776363	ANK2	1.949477224	3.95E-08
ILMN_2108735	EEF1A2	1.948275272	6.02E-08
ILMN_1742025	OLFM1	1.947680614	0.000000859
ILMN_1655720	CNNM4	1.94345342	4.27E-09
ILMN_1787518	GSN	1.939495489	0.000000212
ILMN_1800008	ACAT1	1.936427087	8.22E-10
ILMN_2383874	PRELID2	1.934594792	8.47E-09
ILMN_1693009	FGL2	1.930424887	0.000000148
ILMN_2100357	SMPDL3A	1.929706505	6.9E-10
ILMN_2229379	KIT	1.929017926	0.000000158
ILMN_1682919	PAFAH2	1.925033484	6.08E-12
ILMN_1695706	H3F3B	1.924649235	2.01E-09
ILMN_1793267	ETHE1	1.923819612	1.36E-08
ILMN_2384237	STAP2	1.923759685	0.000000152
ILMN_2110751	CHRNA5	1.920792053	0.00000289
ILMN_1741265	CRYBA2	1.917139715	2.53E-08
ILMN_2283772	HEPACAM2	1.917135994	4.09E-13
ILMN_1660199	ACAA2	1.912549119	0.00000206
ILMN_1800659	PGM1	1.908569073	1.01E-08
ILMN_2405642	DHDDS	1.908523367	1.99E-13
ILMN_1779416	SCUBE2	1.907390584	6.23E-08
ILMN_1719906	HADH	1.903193382	3.08E-08
ILMN_1686109	CCL23	1.894719207	0.00000337
ILMN_2408683	PPAP2B	1.891669195	2.19E-08
ILMN_1735157	GALNT12	1.891175276	4.58E-11
ILMN_1672350	JAM2	1.889378727	2.55E-08
ILMN_1703326	PTGDR2	1.887574308	1.43E-09
ILMN_1789166	SHD	1.886206814	0.000000382
ILMN_1664912	IL11RA	1.885469966	0.000000623
ILMN_1775566	ATP1A1	1.878042329	0.00000377
ILMN_1739521	NLGN1	1.877832965	0.000000422
ILMN_1767842	SLC17A8	1.876977232	0.00000214
ILMN_1750158	ACOX1	1.875699543	0.000000288
ILMN_1744614	PLEKHG6	1.875552673	0.00000322
ILMN_1803180	PRDX6	1.872331682	1.93E-08
ILMN_1686562	KIF13B	1.870163599	5.39E-08
ILMN_1712718	BPNT1	1.868669978	0.000000486
ILMN_1790106	PLP1	1.866624556	3.64E-11
ILMN_1757387	UCHL1	1.865910981	0.000000241

ILMN_1750790	GSTM5	1.861499334	3.36E-14
ILMN_1668351	TAF6	1.860265939	0.000000242
ILMN_1795183	RNASE1	1.857368574	0.00000481
ILMN_2329773	RAB27A	1.857275701	2.11E-09
ILMN_1654861	ACO2	1.855699913	3.92E-11
ILMN_1730416	CYCS	1.855108168	1.28E-14
ILMN_1797735	C1orf21	1.854504103	2.57E-08
ILMN_1678095	SMPDL3B	1.8484059	0.00000237
ILMN_1743232	MICALCL	1.846832608	1.32E-08
ILMN_1677885	FMO4	1.844550439	1.23E-09
ILMN_1807493	ACVRL1	1.839461792	3.54E-11
ILMN_1723134	LOC650695	1.834774116	0.000000202
ILMN_1801307	TNFSF10	1.830734168	0.00000185
ILMN_1759117	XK	1.828495074	0.000000087
ILMN_1744968	KCNAB1	1.82723752	2.46E-08
ILMN_1765620	VSTM2A	1.824382011	1.91E-15
ILMN_1674934	FOXF2	1.823749509	6.39E-11
ILMN_1798971	FZD5	1.818055007	1.05E-10
ILMN_1675117	HSD17B11	1.816815576	0.00000139
ILMN_1704500	STAP2	1.815741089	0.00000141
ILMN_1755383	LRRC1	1.814660131	0.000000161
ILMN_1685441	ASAP3	1.814616409	7.57E-08
ILMN_1774261	DOK4	1.813493381	0.000000374
ILMN_1758672	FAM107B	1.808206092	0.00000223
ILMN_1671482	GALM	1.80675042	7.08E-11
ILMN_1656300	GFRA2	1.804562536	4.56E-11
ILMN_1668134	GSTM1	1.803237784	0.00000178
ILMN_1657515	RPS6KA5	1.802667615	1.29E-08
ILMN_1696316	CPT1A	1.792617418	2.1E-09
ILMN_2412192	CFH	1.792119561	9.42E-08
ILMN_1788394	TTLL6	1.791755223	0.00000016
ILMN_2059886	TTC38	1.791684595	0.000000173
ILMN_1667260	MAPK3	1.786463453	3.7E-09
ILMN_1690209	C1orf186	1.78645616	8.89E-10
ILMN_1701827	CADM3	1.786162378	8.22E-14
ILMN_1729482	KLK15	1.7839798	0.000000366
ILMN_1671191	UQCRC1	1.782631729	4.32E-09
ILMN_1745994	GAS7	1.780272517	0.000000657
ILMN_1794074	MXI1	1.779176811	1.43E-11
ILMN_2240221	SYTL2	1.775381735	0.000000301
ILMN_1720501	ABCA9	1.772867344	4.83E-17
ILMN_1772627	NSG1	1.772720072	0.00000163
ILMN_1693233	KIAA0513	1.769928425	0.000000046
ILMN_1813517	DISP2	1.769880936	0.000000102

ILMN_2165975	CES3	1.769599766	0.000000045
ILMN_1741389	B3GNT8	1.76841688	7.56E-08
ILMN_1680251	SORCS1	1.768382019	6.74E-11
ILMN_1763228	MEF2D	1.766893287	1.09E-10
ILMN_1671076	ADGRB3	1.76408954	9.12E-10
ILMN_2322806	CAST	1.762693844	0.000000563
ILMN_1791593	DENND5B	1.761256706	3.63E-08
ILMN_1747192	RNF125	1.758672976	7.84E-08
ILMN_1788886	TOX	1.757523183	2.99E-09
ILMN_1701386	STRADB	1.757249543	0.000000013
ILMN_1698533	IDH3A	1.755730145	0.000000437
ILMN_1714445	SLC6A9	1.754611635	0.000000177
ILMN_1791302	EFHC2	1.754338484	1.4E-11
ILMN_1778242	CALM1	1.753319164	4.24E-08
ILMN_2347097	KLK15	1.750435388	0.000000463
ILMN_1759910	SERPINA5	1.749517869	0.000000464
ILMN_1704353	IGSF3	1.749113235	0.000000205
ILMN_2254962	ACSS2	1.74390975	5.5E-10
ILMN_2205896	MEIS3P1	1.742334168	0.000000454
ILMN_1742444	UGT2B10	1.740723198	3.57E-09
ILMN_1813846	P2RX4	1.739458404	0.000000151
ILMN_1674609	CLTB	1.735416053	6.28E-12
ILMN_1670263	CNST	1.731935557	4.15E-10
ILMN_1758529	P2RX1	1.731845859	0.00000305
ILMN_2131861	SOCS2	1.731136049	0.00000374
ILMN_1764030	CCL23	1.731067571	2.87E-08
ILMN_1812616	MYO1C	1.730254648	4.12E-09
ILMN_1656927	SEMA5A	1.728768428	0.00000155
ILMN_1810684	TINAG	1.725428642	0.00000198
ILMN_2297710	PLEKHB2	1.721530236	0.000000108
ILMN_2374234	PRKACB	1.718529253	0.000000703
ILMN_2097858	CIPC	1.718104348	7.63E-10
ILMN_2374244	DYRK2	1.71487191	2.31E-08
ILMN_1773073	PHYH	1.71396272	0.000000306
ILMN_1813361	ANGPTL7	1.711175877	0.00000258
ILMN_2352245	RASSF6	1.71030094	0.00000143
ILMN_1773063	OSBPL1A	1.710087968	0.000000047
ILMN_1763091	ELMSAN1	1.709534929	4.99E-12
ILMN_1800713	B3GALT5	1.704454231	0.00000334
ILMN_1674151	Gcom1	1.702910036	1.18E-09
ILMN_1735502	FAM181B	1.702247802	1.7E-12
ILMN_1805225	LPCAT3	1.700863907	0.000000306
ILMN_1710523	ATP8B1	1.696788726	1.99E-08
ILMN_2157510	BLOC1S1	1.696329488	0.00000183

ILMN_1693891	MOGAT2	1.694666855	1.78E-08
ILMN_1778681	EBF1	1.694595038	6.97E-08
ILMN_1740523	KTN1	1.693824485	0.000000314
ILMN_1795826	ATP6V0D1	1.692582892	0.000000055
ILMN_1686985	MTM1	1.691582394	1.46E-09
ILMN_2204876	FLVCR2	1.688616738	0.0000014
ILMN_1688071	NAT1	1.687295642	0.00000408
ILMN_1789463	FXYD1	1.68646782	6.91E-11
ILMN_1779616	SUCLG1	1.686359565	1.84E-09
ILMN_2400947	CELF2	1.68622385	1.62E-08
ILMN_1792972	ZNF439	1.685790876	0.000000232
ILMN_1706635	ELANE	1.68454666	2.64E-16
ILMN_1666924	PINK1	1.683411467	9.26E-09
ILMN_1664608	INPP5A	1.682291172	0.000000782
ILMN_2165354	DCLK1	1.68215491	8.29E-08
ILMN_1727805	SYNGR1	1.681634812	2.85E-08
ILMN_1758315	SLC9A9	1.680503398	0.00000152
ILMN_2310814	MAPT	1.679525279	0.00000465
ILMN_1783131	SAMD13	1.67699076	0.00000121
ILMN_2048607	ANKRD9	1.676785737	6.53E-08
ILMN_2179717	FAM189A2	1.67569667	0.000000632
ILMN_1789535	DHDDS	1.674903248	6.55E-13
ILMN_1774071	CD177	1.67236846	0.000000535
ILMN_2364864	MB	1.671437709	7.84E-08
ILMN_2193864	PHOX2B	1.670201424	7.33E-12
ILMN_1770682	TEP1	1.669690692	0.000000776
ILMN_1773814	MUSTN1	1.669374768	1.56E-09
ILMN_1688702	PJA2	1.669076419	0.000000359
ILMN_1742544	MEF2C	1.662314411	0.000000525
ILMN_2195319	PDE6A	1.661859723	1.05E-09
ILMN_1771622	CALY	1.661371142	1.66E-08
ILMN_1711928	ACSF2	1.66136292	0.00000428
ILMN_1738921	ACAA1	1.661218715	0.000000239
ILMN_1703330	FEM1C	1.660691184	1.69E-09
ILMN_1672486	TCF7L2	1.659838458	0.000000435
ILMN_2358919	TP53I3	1.659600285	0.00000459
ILMN_2319952	VDR	1.657784283	0.00000206
ILMN_1682232	MIER1	1.657337152	9.54E-10
ILMN_1774330	WSCD1	1.657297462	9.03E-12
ILMN_1795561	CAMK1D	1.657169565	5.45E-08
ILMN_1782429	TMEM56	1.656761427	0.00000438
ILMN_2366714	UQCR10	1.656589432	8.08E-11
ILMN_2229940	PRADC1	1.655518933	8.18E-11
ILMN_2076429	GALNTL6	1.655326219	0.000000155

ILMN_1768705	SYT4	1.654623124	0.000000346
ILMN_1752669	ALPI	1.65109224	0.00000324
ILMN_1660552	BMP5	1.650457135	3.22E-12
ILMN_1718907	TSHZ1	1.650415562	1.49E-10
ILMN_1666004	WASL	1.64749723	1.39E-08
ILMN_2406106	CACNB2	1.644759117	0.00000319
ILMN_1715173	RPS6KA1	1.642451421	0.00000203
ILMN_1662886	KLHL34	1.640361161	2.58E-10
ILMN_1719599	SYTL4	1.636775698	0.00000446
ILMN_1781691	TRAK2	1.636165006	1.37E-10
ILMN_1676383	ENPP6	1.632863021	1.48E-12
ILMN_1663975	CNNM2	1.630117095	3.6E-12
ILMN_1688242	C6	1.629972055	1.03E-08
ILMN_1679580	KCNIP4	1.629653976	2.07E-08
ILMN_1803094	PDGFD	1.628733902	1.84E-08
ILMN_2053679	ACADM	1.628686668	5.67E-08
ILMN_1701170	BARX2	1.626439716	5.31E-08
ILMN_2405018	PPP1CB	1.626202108	0.000000124
ILMN_2055760	LNPK	1.624107874	4.08E-11
ILMN_1813763	SLC20A2	1.622046783	9.92E-08
ILMN_1757338	PLSCR4	1.618550355	0.000000842
ILMN_1719351	TCF21	1.61494858	8.56E-15
ILMN_1664236	ADAM28	1.614440084	0.000000169
ILMN_2284181	UGP2	1.612879168	1.73E-09
ILMN_1685156	ADCY6	1.612845473	3.34E-08
ILMN_1718629	NRIP1	1.612191365	3.81E-08
ILMN_1728698	GDE1	1.607563627	0.000000291
ILMN_1754753	IL6R	1.607266331	5.51E-11
ILMN_1700042	TLN2	1.606749719	0.000000297
ILMN_2256953	CASZ1	1.606416051	2.11E-10
ILMN_1690682	B3GALT4	1.60421577	4.21E-10
ILMN_1807925	GNG2	1.603557737	1.07E-09
ILMN_2287157	DST	1.602875376	0.00000271
ILMN_1713759	UBE2J1	1.602686779	0.000000344
ILMN_1675857	COL4A6	1.601657818	9.08E-09
ILMN_1697529	RNF10	1.600791542	2.22E-09
ILMN_1737406	KLF6	1.598784033	0.00000194
ILMN_1767111	ANO10	1.595010808	0.000000138
ILMN_2100815	TMEM9B	1.594234561	2.77E-08
ILMN_1801090	KRT222	1.592088507	3.42E-14
ILMN_1651385	MFN2	1.590762017	4.99E-09
ILMN_1654287	ADCY9	1.589989788	1.14E-12
ILMN_2160428	IL1RAPL1	1.588485059	3.54E-10
ILMN_1652806	ATP5J	1.588145211	0.00000219

ILMN_2166524	CCNYL1	1.586569247	0.000000182
ILMN_2353143	FBLIM1	1.584785955	1E-10
ILMN_1782761	ARHGAP20	1.584763019	1.04E-12
ILMN_2319913	DGKA	1.584623991	0.00000119
ILMN_1656826	SH3RF1	1.582694845	0.000000251
ILMN_1764769	VWA5A	1.581196186	6.76E-09
ILMN_1686988	ST5	1.580137956	0.00000103
ILMN_1706511	TEF	1.579320615	1.47E-08
ILMN_1653115	ECH1	1.576744324	0.00000121
ILMN_1809259	HRASLS2	1.574961148	0.00000406
ILMN_1710619	C17orf73	1.574572907	0.00000221
ILMN_1795063	ZADH2	1.573997021	8.98E-08
ILMN_1682996	VWA5A	1.573773608	5.2E-10
ILMN_1721106	C14orf159	1.572132234	0.000000159
ILMN_2263466	ACADVL	1.570572694	0.000000609
ILMN_1763516	SPINK2	1.569360039	6.27E-11
ILMN_1709094	LIFR	1.569343015	1.11E-11
ILMN_1662038	LARGE1	1.56844712	0.00000312
ILMN_1687315	RXRA	1.568358769	9.6E-09
ILMN_1747197	SLC41A2	1.567158132	0.00000106
ILMN_2415303	CLEC10A	1.565122544	0.0000021
ILMN_1733648	SNAP91	1.563329701	1.05E-09
ILMN_2203876	CCDC68	1.561546045	4.04E-08
ILMN_1746408	MIDN	1.55936605	0.00000305
ILMN_1688633	CLCN2	1.559173245	1.78E-08
ILMN_2205963	C10orf54	1.557864774	0.00000147
ILMN_1758673	SLC44A1	1.557441139	0.00000311
ILMN_1729450	C16orf62	1.555173661	2.22E-10
ILMN_1673529	PHYKPL	1.555126263	0.000000661
ILMN_1667429	SLC25A20	1.554962879	2.02E-09
ILMN_1664292	ZNF415	1.553438676	0.00000102
ILMN_1749846	OMD	1.553115681	0.000000109
ILMN_2363634	ADHFE1	1.549266024	0.000000393
ILMN_2091375	KRCC1	1.548439902	0.000000129
ILMN_1762972	CHD9	1.547638271	3.96E-09
ILMN_1803772	POLD4	1.547378196	0.00000291
ILMN_1782079	ZFP3	1.547280489	0.00000227
ILMN_1753016	MRPL35	1.545657554	8.47E-09
ILMN_1661875	ANK3	1.54461041	1.12E-13
ILMN_2342835	P2RY14	1.544414067	8.13E-10
ILMN_2113490	NTN4	1.544110972	0.00000136
ILMN_1755643	MGAT4A	1.543175004	0.00000092
ILMN_1740269	WNT2B	1.542925251	0.00000155
ILMN_1721989	ATP5F1	1.541353849	3.34E-09

ILMN_2048636	ME2	1.539411265	0.00000462
ILMN_1672191	ATP5F1	1.539078609	0.0000031
ILMN_1811636	IFT57	1.537940234	8.26E-10
ILMN_2234956	LEPR	1.536397573	0.00000469
ILMN_1679115	TPH1	1.535813921	4.91E-09
ILMN_2352009	ACADVL	1.535430818	0.00000248
ILMN_1741684	SMPD1	1.534658409	1.41E-10
ILMN_1665775	43892	1.533125309	2.08E-08
ILMN_1779410	VAPA	1.532177319	5.08E-08
ILMN_2402581	MYZAP	1.531572276	1.1E-09
ILMN_1791508	MMP28	1.53082113	4.2E-10
ILMN_1718646	MMP15	1.529146934	7.62E-09
ILMN_1670535	NDRG2	1.528924716	3.67E-11
ILMN_1707310	MGLL	1.528770114	1.06E-10
ILMN_2319910	DGKA	1.526796834	0.00000133
ILMN_1671489	PC	1.526576269	0.00000133
ILMN_1668247	LTC4S	1.526561043	3.78E-08
ILMN_1811426	LOC653626	1.526435987	0.00000289
ILMN_1656902	HECTD3	1.525904635	3.52E-08
ILMN_1695590	ADRB2	1.52584199	0.000000947
ILMN_1674560	GBA2	1.525107683	0.00000136
ILMN_1753502	IGSF11	1.524950697	5.92E-09
ILMN_1697793	SYNJ2BP	1.524513272	0.00000108
ILMN_1801851	ALPPL2	1.523308473	0.00000064
ILMN_1761945	FGFBP2	1.522682657	0.00000181
ILMN_1702396	PACSIN2	1.52167405	1.43E-08
ILMN_2228180	MSRA	1.521451168	0.00000349
ILMN_2376859	PDGFD	1.521355161	0.000000894
ILMN_2124471	SLC36A1	1.519839428	0.000000326
ILMN_1757370	SMPD1	1.519693856	5.45E-10
ILMN_1654541	ATP6V1G2	1.519108315	1.09E-08
ILMN_1673704	INA	1.518496991	1.49E-08
ILMN_1749848	SLC35F1	1.516825055	0.000000528
ILMN_1700652	CHAD	1.516644438	0.000000651
ILMN_1692413	NMNAT1	1.511734	7.36E-09
ILMN_1653750	SOX10	1.511677857	0.000000098
ILMN_2311674	ADCY6	1.511414239	3.83E-09
ILMN_1801105	PRKCD	1.510470167	0.00000217
ILMN_1726549	GDPD2	1.510037733	6.86E-09
ILMN_1659371	ADRA2A	1.509947376	0.00000191
ILMN_1806721	MS4A2	1.507768744	0.00000385
ILMN_1654692	RALGPS2	1.506069033	0.000000116
ILMN_1812777	MRPL35	1.504855633	6.28E-08
ILMN_2408430	LARGE1	1.503444686	4.25E-08

ILMN_2299450	P2RX2	1.501380937	1.18E-10
ILMN_2371825	AGL	1.500320579	0.00000393
ILMN_2193214	MPND	1.500254264	5.58E-09
ILMN_1726368	ZNF135	1.499755363	0.00000172
ILMN_2410406	AKAP7	1.499699061	5.38E-08
ILMN_2197846	HADHB	1.498332684	0.000000271
ILMN_2288232	PHKB	1.498183429	3.04E-09
ILMN_1722286	ABCA5	1.496272664	6.46E-08
ILMN_1764609	PWWP2B	1.4951608	0.00000143
ILMN_1752668	DAAM2	1.493916289	2.38E-11
ILMN_1732127	RBKS	1.490712195	0.00000261
ILMN_1755504	CALCOCO2	1.489389415	1.35E-10
ILMN_1728083	EIF4EBP2	1.489191524	0.000000103
ILMN_2406299	SEMA3B	1.488601371	0.00000383
ILMN_2347068	MKNK2	1.488174455	0.00000123
ILMN_1656840	VPS13D	1.487262094	2.24E-09
ILMN_1684210	NIPAL3	1.48612837	0.000000356
ILMN_1686805	CRK	1.485623931	0.00000313
ILMN_1673950	STBD1	1.485029613	2.16E-08
ILMN_2052331	MYOC	1.484609381	0.000000312
ILMN_1784333	SECISBP2L	1.481922643	8.84E-10
ILMN_2369603	CPEB1	1.481604186	0.00000176
ILMN_1756898	COQ9	1.48110542	0.000000215
ILMN_1772667	P2RY1	1.480906586	5.67E-11
ILMN_1701940	MIGA2	1.480225823	7.57E-08
ILMN_1764494	ATP5A1	1.47996331	0.00000173
ILMN_1773228	DLST	1.479438126	1.94E-08
ILMN_1781996	NUDT16	1.479279853	7.2E-09
ILMN_1682099	TNFAIP8L3	1.477945909	0.000000473
ILMN_1659312	PMP22	1.476734644	4.19E-10
ILMN_2340347	PC	1.473481891	2.45E-08
ILMN_1658800	BRPF3	1.471198965	0.00000129
ILMN_1662935	C1QTNF7	1.470848567	1.65E-09
ILMN_1738652	BAD	1.470406277	0.000000173
ILMN_1779670	A1CF	1.469856099	0.00000392
ILMN_1770466	ATP5G3	1.469003568	0.00000255
ILMN_1713491	VAMP2	1.468266957	0.000000743
ILMN_1676998	SCN2B	1.466909086	2.79E-08
ILMN_2412922	ELMSAN1	1.465497981	0.000000136
ILMN_1665492	SORCS1	1.465252471	1.11E-08
ILMN_1654060	MKNK2	1.465056375	1.24E-09
ILMN_1736888	SAR1B	1.46311995	0.00000151
ILMN_1749001	ABCA5	1.463114403	0.000000472
ILMN_1773901	STX12	1.462596779	6.27E-08

ILMN_1742788	PKD1L2	1.459556369	4.23E-12
ILMN_2385566	PHYH	1.458993241	5.68E-08
ILMN_1661833	ANKRD12	1.458937874	2.99E-08
ILMN_1731397	STMN4	1.458087966	1.96E-11
ILMN_2129015	AFF1	1.454436812	1.05E-08
ILMN_2298988	ENTPD8	1.453894535	6.56E-09
ILMN_2361920	SGK2	1.451954435	0.00000255
ILMN_1776515	MPPE1	1.449718207	4.92E-08
ILMN_1720024	IL11RA	1.44864902	0.0000008
ILMN_1657087	ZNF385B	1.44815399	0.000000426
ILMN_1660306	ITGA8	1.4480818	0.00000232
ILMN_1773278	OR8D4	1.446009249	6.08E-13
ILMN_1810729	UBL3	1.444967997	0.000000628
ILMN_1652128	LMBRD1	1.443791995	0.00000399
ILMN_1706056	ENTPD8	1.442940099	0.000000111
ILMN_1780236	PMM1	1.442680458	0.000000621
ILMN_1800642	RELN	1.441919916	0.000000294
ILMN_1796180	CRY2	1.439374046	0.000000972
ILMN_1699728	BTD	1.438974474	1.52E-09
ILMN_1691291	PIGS	1.438484614	1.09E-10
ILMN_2150586	ZNF536	1.435821644	1.63E-14
ILMN_2122420	HMGCL	1.435743211	0.0000003
ILMN_2345292	FXYD1	1.435256809	0.000000793
ILMN_1765232	RNLS	1.43170904	0.00000283
ILMN_1696806	CTNND1	1.4313375	0.000000322
ILMN_1765858	CAB39	1.429426183	0.0000033
ILMN_1664679	CADM2	1.428864618	0.00000219
ILMN_2221784	LRRC75A	1.428689048	1.75E-08
ILMN_1745988	NUDT12	1.427213295	4.36E-09
ILMN_1792110	C10orf76	1.426007402	7.66E-08
ILMN_1792800	LCN6	1.425758734	0.000000046
ILMN_2333219	ACHE	1.425728701	0.000000327
ILMN_1728803	HMP19	1.425254009	6.89E-10
ILMN_1786718	NDUFV1	1.423884791	0.00000221
ILMN_2380494	ANXA11	1.422377315	4.21E-10
ILMN_1684357	C14orf139	1.421660546	3.65E-09
ILMN_1807095	MRPS36	1.419982244	0.000000924
ILMN_1696394	IL6R	1.419603405	9.46E-09
ILMN_1692539	SH3BP1	1.419420768	0.00000173
ILMN_2295987	NBPF1	1.417420589	6.78E-08
ILMN_1728467	GHITM	1.416032708	0.00000199
ILMN_1813581	CNR1	1.414226227	4.68E-09
ILMN_1808537	LRRC66	1.411504834	0.000000275
ILMN_1663099	UBE2B	1.411292561	9.27E-10

ILMN_2390609	ANK3	1.41090987	0.000000283
ILMN_1653940	USP2	1.410825043	9.6E-09
ILMN_1674034	H2AFY	1.410596534	0.00000106
ILMN_2232066	ABCB11	1.410247002	0.00000364
ILMN_1715674	ITPK1	1.409945531	0.00000173
ILMN_2064694	STIM1	1.409435708	4.19E-09
ILMN_1726575	CHODL	1.408912318	5.65E-09
ILMN_1723909	LOC402176	1.408782946	3.4E-09
ILMN_2410421	NBPF1	1.407747569	9.46E-08
ILMN_1810441	TRIM3	1.405799903	5.61E-08
ILMN_1751460	FEV	1.404783369	5.55E-08
ILMN_1715662	CCDC80	1.404714811	0.000000333
ILMN_1716563	PRKCB	1.404147877	0.00000214
ILMN_1664631	ADAM28	1.40213356	0.0000025
ILMN_1793915	MXI1	1.401783366	0.000000498
ILMN_1737588	C21orf33	1.395782598	1.94E-08
ILMN_2329165	MYO1C	1.394351264	0.00000173
ILMN_2065254	ELAVL4	1.394284037	2.13E-09
ILMN_1773906	NCOA4	1.391890279	0.00000369
ILMN_1655983	CDC14A	1.390619264	0.00000371
ILMN_1712751	HADHA	1.389454932	4.96E-08
ILMN_1654370	TESK2	1.389381989	0.00000395
ILMN_1711807	ADGRL3	1.387953351	4.69E-13
ILMN_2366710	UQCR10	1.387688263	0.00000002
ILMN_2071826	RNF152	1.386793827	2.91E-11
ILMN_1738494	AQP7	1.384305717	0.00000178
ILMN_1713301	DGCR2	1.384229974	0.00000223
ILMN_1658040	CRABP1	1.383929719	0.00000368
ILMN_2384158	CNGA3	1.38304828	0.00000235
ILMN_1795419	CNPPD1	1.382313474	0.000000138
ILMN_1665132	CD36	1.379768051	8.19E-10
ILMN_1734695	MAP4	1.378842908	0.0000019
ILMN_1798841	PLCXD3	1.377123934	6.3E-11
ILMN_2352077	KL	1.377013258	0.000000492
ILMN_2189406	ARPIN	1.37690095	0.000000208
ILMN_2325168	ARRB1	1.365023132	6.18E-09
ILMN_2313782	ATG4A	1.364541006	0.0000023
ILMN_1783149	CDH23	1.35892589	0.000000542
ILMN_1680239	NUDT9	1.358101127	0.000000333
ILMN_1716057	CAPN2	1.358012943	0.00000379
ILMN_1790885	KCNK10	1.357637848	0.00000213
ILMN_1706859	SMDT1	1.357288277	1.96E-08
ILMN_1718896	CNTN2	1.357007628	5.24E-11
ILMN_2234310	CPTP	1.356911106	0.00000219

ILMN_1800855	PPTC7	1.356407785	0.000000829
ILMN_2408946	PDE8A	1.353480329	0.000000256
ILMN_1767129	ABCC8	1.353414425	2.09E-11
ILMN_2195236	PGRMC2	1.353376891	0.000000903
ILMN_1711810	PNKD	1.350346676	0.00000473
ILMN_1701483	SYP	1.35034033	3.85E-08
ILMN_2383516	WDR7	1.349720308	0.000000409
ILMN_1731596	AP3S2	1.348486529	0.0000034
ILMN_1685170	ANXA11	1.347829745	0.00000225
ILMN_1797310	ATP6V1D	1.346034241	9.77E-08
ILMN_1811178	SCAPER	1.345748335	0.00000296
ILMN_1659649	SGCG	1.340487578	3.75E-11
ILMN_1707553	GAREM1	1.338989001	0.00000198
ILMN_1744676	RNF112	1.338114481	3.68E-10
ILMN_2375156	PHF7	1.337957278	0.000000149
ILMN_1730620	ARRB1	1.33764784	6.45E-08
ILMN_1722771	LDHD	1.336323453	0.000000206
ILMN_1750969	FAM120AOS	1.334338064	1.45E-11
ILMN_1705848	FAM104B	1.33377104	0.000000462
ILMN_1771862	TXNDC11	1.328794328	0.00000226
ILMN_1659857	SNAP29	1.328666492	2.27E-08
ILMN_1666986	GCNT2	1.325577895	0.00000298
ILMN_2383455	SUOX	1.325499991	0.00000326
ILMN_1731609	CHMP6	1.321922144	1.02E-08
ILMN_2413572	MARK2	1.321654936	0.00000341
ILMN_2130409	PEX26	1.319674829	1.6E-09
ILMN_1671898	LMX1A	1.317835942	0.00000369
ILMN_1778425	DPP10	1.317323576	8.75E-10
ILMN_2169692	SCN9A	1.305959186	1.26E-09
ILMN_2197946	SCG3	1.304536961	0.00000395
ILMN_1675992	DST	1.304352257	0.00000112
ILMN_1778132	C2CD2L	1.303109447	0.000000393
ILMN_1681008	CGRRF1	1.302982005	0.000000484
ILMN_2352917	LTBP4	1.297022314	4.4E-09
ILMN_1775508	CYLD	1.296135322	0.00000319
ILMN_1761262	MPI	1.291995755	0.00000261
ILMN_1670308	GPM6B	1.291301063	0.00000214
ILMN_1704380	INCA1	1.290824271	0.00000386
ILMN_2084836	CORO2B	1.290068436	2.01E-10
ILMN_1800332	NGB	1.289416339	0.00000364
ILMN_1747577	ALAD	1.288931237	0.00000289
ILMN_1771148	CNTN4	1.288046913	0.00000149
ILMN_1783500	HOXD12	1.287820491	0.0000036
ILMN_1807689	PKNOX2	1.287741422	1.02E-09

ILMN_1741032	HERC3	1.28744897	0.00000465
ILMN_1705397	PDK2	1.287031282	0.000000747
ILMN_1653161	SNCG	1.284347795	0.00000214
ILMN_1673152	NRXN1	1.279333989	0.000000218
ILMN_1689976	EDIL3	1.277498564	0.000000995
ILMN_2334359	GFRA1	1.276460461	0.000000379
ILMN_1726496	SEL1L	1.276247195	0.00000447
ILMN_1716803	RALYL	1.275693751	1.46E-10
ILMN_1721769	FBLN2	1.275502848	1.99E-09
ILMN_2371152	KLC4	1.274383754	0.00000315
ILMN_1693345	CPEB1	1.274132867	0.000000688
ILMN_2121979	UGT2A3	1.272631459	2.07E-08
ILMN_1771852	ZNF575	1.268844136	0.000000858
ILMN_1667578	DUSP26	1.268409423	4.58E-10
ILMN_2115350	TNXA	1.268298755	4.07E-10
ILMN_1779370	ARHGEF9	1.267899979	1.12E-10
ILMN_1713835	ENHO	1.267065917	1.62E-08
ILMN_1712023	SLC8A3	1.263984919	1.52E-08
ILMN_2399622	AP1G1	1.26368199	0.00000167
ILMN_2398847	ARHGAP17	1.263662641	0.00000012
ILMN_1791270	CDH10	1.262466897	9.32E-09
ILMN_1809013	MYL6	1.260812008	0.0000019
ILMN_1752884	AADACL2	1.259077065	2.66E-09
ILMN_1707513	PGPEP1	1.2549235	0.000000789
ILMN_2291455	FAM102A	1.254558609	0.00000137
ILMN_1738773	HRC	1.253580019	0.000000672
ILMN_1655521	DMRTA1	1.252497966	2.65E-10
ILMN_1711328	EPHA10	1.252455296	0.00000176
ILMN_1687751	BAALC	1.24445247	3.61E-10
ILMN_1654246	SIRT6	1.243510069	0.00000168
ILMN_1690329	FAM118B	1.242134234	0.00000229
ILMN_2157544	GBF1	1.241444942	0.00000033
ILMN_1798624	MOCS1	1.241279608	0.000000728
ILMN_1705627	USP2	1.241231065	7.95E-08
ILMN_1674984	LPAR1	1.240904354	1.99E-08
ILMN_1806147	GNG3	1.240322853	0.000000235
ILMN_1731688	KLB	1.234878584	9.61E-09
ILMN_1682459	TUBB4A	1.23308687	0.000000241
ILMN_2287543	SHISA3	1.23308646	0.000000332
ILMN_2341382	ANKHD1	1.232331153	0.00000351
ILMN_2290204	ARHGAP28	1.227366167	0.0000031
ILMN_1797892	SCN11A	1.226054976	4.63E-09
ILMN_1668446	ABCA9	1.223934218	0.000000104
ILMN_1711504	NRXN1	1.223913815	0.000000305

ILMN_1800270	GABRG2	1.218296915	0.00000086
ILMN_2279484	C2orf88	1.216324253	0.000000164
ILMN_1700770	PSKH1	1.211518899	0.000000884
ILMN_1778277	HTR3E	1.210029366	0.00000289
ILMN_1786080	HTR4	1.209906666	3.22E-08
ILMN_1682100	IGSF10	1.208761227	0.000000432
ILMN_2317493	SEMA6D	1.207112225	0.00000014
ILMN_1740024	NAALAD2	1.205860802	0.00000175
ILMN_1812302	RMDN2	1.204200716	0.00000301
ILMN_1788400	KIAA2022	1.20410423	0.000000391
ILMN_1806407	SEMA6D	1.203013715	0.000000249
ILMN_1799099	TEKT3	1.202445545	1.28E-08
ILMN_1665435	GPER1	1.200377864	1.44E-08
ILMN_2287941	CYLD	1.197889689	1.63E-09
ILMN_1724183	CDKL1	1.197285689	3.56E-08
ILMN_1680828	KRT27	1.197173193	0.000000411
ILMN_1685467	ZNF397	1.196232045	0.000000365
ILMN_1700547	CHST9	1.191919283	0.00000439
ILMN_1784626	CA7	1.188328263	0.000000126
ILMN_1685056	FLRT1	1.183998563	0.000000479
ILMN_1763267	ZFYVE28	1.178569527	0.000000439
ILMN_1814770	CPNE4	1.174509319	1.95E-10
ILMN_1729165	TCEAL6	1.171915541	0.00000124
ILMN_1692433	RECQL5	1.170813544	0.0000026
ILMN_2129886	ABCA8	1.168398896	0.000000134
ILMN_1673360	BDH2	1.167158045	0.00000179
ILMN_1815033	HPCA	1.166407855	5.83E-08
ILMN_2215989	NEFM	1.165976143	0.000000616
ILMN_1771223	PADI2	1.15455203	0.00000429
ILMN_2111255	VIT	1.152948818	0.000000718
ILMN_2293529	AKAP7	1.142735426	0.000000653
ILMN_1775373	CMTM5	1.138371454	0.000000958
ILMN_1692623	LOC653950	1.137972205	0.000000274
ILMN_2298464	MOBP	1.13769637	0.00000459
ILMN_1753005	RELN	1.137301338	0.000000223
ILMN_1763612	TMEM132D	1.135728992	0.00000467
ILMN_1679694	MMP27	1.127498356	0.000000658
ILMN_1809730	RXRG	1.121638846	9.07E-09
ILMN_2166696	ANGPTL5	1.121611168	0.000000967
ILMN_1770629	SLC17A7	1.111421836	0.00000117
ILMN_2339192	HMGCLL1	1.105224562	5.86E-09
ILMN_1708615	TNXB	1.104388783	0.0000034
ILMN_2372199	C6orf1	0.918585732	0.00000417
ILMN_1779742	GABRD	0.911676662	0.00000388

ILMN_1751395	FLJ12949	0.89215115	0.00000268
ILMN_2364414	PCDHA6	0.891116648	0.00000394
ILMN_1721029	RHEBL1	0.885122665	0.00000204
ILMN_1730729	TMEM206	0.884984363	0.00000107
ILMN_2148012	EPR1	0.884300298	0.00000349
ILMN_1672393	CUL7	0.876542519	0.00000116
ILMN_1806779	ZNF155	0.874646915	0.00000392
ILMN_1656942	PRICKLE3	0.873752413	0.00000269
ILMN_2048633	LRRC8E	0.873485997	0.00000351
ILMN_1740776	OR5B12	0.873328454	0.000000108
ILMN_1676833	CYB5RL	0.871199276	0.00000116
ILMN_1802146	ZNF74	0.866515523	1.38E-08
ILMN_2273103	ELK4	0.864794486	0.00000296
ILMN_1733874	SH2D2A	0.859334594	3.6E-10
ILMN_1653163	ZSCAN2	0.855186284	0.000000881
ILMN_2322597	LYPD5	0.853944628	0.0000022
ILMN_1776119	ABCC10	0.853690536	0.00000429
ILMN_2073184	S1PR5	0.852559838	0.000000125
ILMN_2173891	FAAP24	0.852054448	0.00000155
ILMN_1732336	RFC2	0.85049638	0.00000162
ILMN_1712894	FUT1	0.848752021	0.000000119
ILMN_1742003	RHPN1	0.847223188	0.00000169
ILMN_2386040	MYO19	0.845679499	0.00000293
ILMN_1677794	BRCA2	0.844345046	1.55E-08
ILMN_2392940	CEP250	0.840702833	0.00000441
ILMN_1807379	WHSC1L1	0.840452564	0.000000485
ILMN_2121555	FBXO5	0.834211711	2.57E-08
ILMN_1708900	TMEM161A	0.833876045	0.0000043
ILMN_1718830	ICE2	0.830155992	0.0000024
ILMN_1683747	CD276	0.827040474	0.00000352
ILMN_1689932	DPH7	0.825819838	0.000000959
ILMN_1689720	PPP2R3B	0.825243932	0.00000018
ILMN_1750102	EME1	0.825211287	0.00000355
ILMN_1774806	SLC9A7	0.82517563	0.000000036
ILMN_1682198	CSTF1	0.824783753	0.00000447
ILMN_1808325	TM4SF19	0.824698461	7.71E-08
ILMN_2127624	UBE3D	0.823617466	0.00000258
ILMN_1783075	MMS22L	0.821484853	3.78E-08
ILMN_1763467	LY6G5C	0.821138623	0.00000299
ILMN_1698252	FANCB	0.817637483	0.000000173
ILMN_1666882	DACH1	0.817623853	0.000000445
ILMN_1687419	DHX8	0.817305994	0.000000215
ILMN_1718668	ZBTB26	0.817020017	0.00000341
ILMN_1685109	POLR3D	0.816072742	0.000000431

ILMN_1712608	BFSP1	0.815813412	0.000000381
ILMN_1774722	CGREF1	0.813786916	0.00000356
ILMN_2115974	GSDMA	0.813729602	0.000000134
ILMN_1665622	WDR5B	0.812748616	0.00000229
ILMN_2366795	KIF27	0.811681699	3.92E-08
ILMN_1668984	SPNS3	0.811443601	0.00000228
ILMN_1684591	ZSCAN32	0.811108715	0.00000264
ILMN_1726698	PSME1	0.810834393	0.00000128
ILMN_2347044	SLC25A14	0.808874329	0.00000196
ILMN_1775183	CBX8	0.807664436	1.83E-11
ILMN_1735792	RHBDF2	0.807131234	5.13E-08
ILMN_1697975	PHF14	0.806638913	0.00000194
ILMN_1779040	INO80B	0.806306809	0.00000014
ILMN_1721732	GSDMC	0.805559283	5.5E-10
ILMN_1812915	TNFRSF10B	0.803656734	0.00000157
ILMN_2084073	UCN	0.802750147	0.00000244
ILMN_1663171	MATN3	0.802362503	0.000000289
ILMN_1712257	PPP2R3B	0.802318401	0.00000118
ILMN_1660541	PCNXL2	0.801357115	0.00000198
ILMN_1813701	RASAL2	0.800081704	0.00000313
ILMN_1732921	ITGB8	0.798767312	0.000000231
ILMN_1813277	SUPT3H	0.798429488	0.00000202
ILMN_2346649	ZNF473	0.796914333	0.00000156
ILMN_2302654	LRP8	0.796807946	5.72E-11
ILMN_1652487	RIOK1	0.796372286	0.00000448
ILMN_1777156	GTPBP3	0.795663658	0.00000266
ILMN_2376833	ZNF200	0.795176436	0.00000266
ILMN_1778168	ELMO2	0.794699135	0.00000341
ILMN_1780172	FNDC11	0.791619036	5.77E-08
ILMN_2343273	TMEM255B	0.791531895	0.00000305
ILMN_1716370	TNS4	0.791379122	0.00000374
ILMN_1723117	IPO9	0.791247351	0.000000027
ILMN_2415011	DCTD	0.789769052	0.00000292
ILMN_1752111	SMARCAL1	0.789449852	0.000000444
ILMN_1692620	ZNF263	0.786765685	0.00000297
ILMN_1731193	BCL2L12	0.786586265	0.00000138
ILMN_2357062	IL1RAP	0.78603049	7.28E-08
ILMN_2251375	ZFP64	0.785645299	0.000000162
ILMN_1687275	AJUBA	0.78562662	1.6E-10
ILMN_1691487	TRAF2	0.785589428	0.00000027
ILMN_1677113	RNF8	0.784847188	0.0000023
ILMN_1812967	CNPY3	0.783762808	0.00000114
ILMN_1747870	CD3EAP	0.783070872	2.65E-08
ILMN_1651544	HDAC8	0.782546941	0.000000816

ILMN_2283398	DUSP4	0.782084567	0.00000151
ILMN_1799011	CEP250	0.78082973	0.000000074
ILMN_2370685	C6orf1	0.780316943	0.00000221
ILMN_1749071	CNIH3	0.779804913	0.000000615
ILMN_1798819	DTNB	0.779710744	7.29E-09
ILMN_1771957	MAN1B1	0.778934261	0.00000307
ILMN_2370907	RAD51D	0.778247256	0.00000152
ILMN_1771805	ELK4	0.778014377	9.59E-08
ILMN_1684726	C2orf27A	0.777226874	2.64E-08
ILMN_1712505	KDELC1	0.777220118	0.000000243
ILMN_2260392	ACTR3B	0.777168466	0.000000138
ILMN_1760506	LOC158160	0.776202363	6.68E-08
ILMN_2317658	SLC3A2	0.775917048	0.000000666
ILMN_2313946	CPNE7	0.775279896	0.00000098
ILMN_1676864	DDX31	0.775221748	0.000000331
ILMN_2405078	OSBPL8	0.774580308	0.00000107
ILMN_2351916	EXO1	0.774036364	0.000000199
ILMN_1802951	NUF2	0.773745088	3.78E-08
ILMN_1760332	PIGL	0.77275409	0.00000278
ILMN_1796813	EED	0.772635922	0.00000409
ILMN_1785914	CDC6	0.770985488	0.0000017
ILMN_1703013	NOC4L	0.770972101	0.0000024
ILMN_1787370	NDUFAF7	0.770741602	0.000000437
ILMN_1702211	TGIF1	0.769200806	0.000000679
ILMN_1656335	RIT1	0.768915608	0.000000387
ILMN_1673798	PPOX	0.768488294	0.0000042
ILMN_1655482	TRIM27	0.768435902	7.64E-08
ILMN_2302075	MSI2	0.767856859	0.000000319
ILMN_1667883	THOC5	0.766958994	0.00000358
ILMN_1804929	OXTR	0.765881842	0.000000264
ILMN_2259223	TMTC4	0.765842893	0.00000313
ILMN_1810834	MSH5	0.765466624	0.000000444
ILMN_1751743	XRCC1	0.765080098	0.000000383
ILMN_1807609	SPTBN5	0.765034196	0.000000378
ILMN_1720442	NCBP2	0.764632517	0.00000351
ILMN_2191436	POLA1	0.763083137	0.0000017
ILMN_2323491	NUP62	0.76293769	0.000000499
ILMN_1664682	DNA2L	0.762187192	0.00000126
ILMN_2163819	KIF21B	0.761614299	0.0000024
ILMN_1736176	PLK1	0.761473392	0.00000264
ILMN_1691180	OTX1	0.761327381	0.000000202
ILMN_1730631	WDCP	0.761310647	0.00000227
ILMN_1693210	NSMCE2	0.761212987	0.0000032
ILMN_1653896	ATRIP	0.761159756	0.000000056

ILMN_1693430	NME1-NME2	0.761013736	0.000000367
ILMN_1781680	DAP3	0.760941478	0.0000028
ILMN_1670723	MSL3	0.760691411	4.58E-09
ILMN_1708486	CNN2	0.760668823	0.000000787
ILMN_1676745	ZNF142	0.760183892	0.00000016
ILMN_2048388	ZFP69B	0.760161219	0.00000229
ILMN_1767324	EIF4EBP1	0.759988698	0.000000108
ILMN_1809662	LOC440145	0.759495794	5.08E-09
ILMN_1673450	DDN	0.759143338	0.00000413
ILMN_1678745	NUDCD1	0.758485797	0.000000982
ILMN_1761147	GABPB1	0.757909024	0.00000102
ILMN_2307656	AGTRAP	0.757899663	0.00000157
ILMN_1675695	APRIN	0.757859208	0.000000886
ILMN_2373779	COPS8	0.757813791	0.00000414
ILMN_1778152	FIGNL1	0.757673776	0.000000876
ILMN_1733164	FBXO11	0.75747174	0.000000228
ILMN_1702817	B3GNTL1	0.75679247	5.1E-11
ILMN_2076463	SLC15A4	0.7560988	0.00000074
ILMN_1811102	LRSAM1	0.755403739	0.00000327
ILMN_2157020	SNORD48	0.755375507	0.000000176
ILMN_2124951	RBMX	0.755169687	0.00000443
ILMN_1688853	ZBTB9	0.755001204	0.000000243
ILMN_1669674	CNPY3	0.753825064	0.00000275
ILMN_1722156	RWDD2A	0.753780307	0.000000294
ILMN_1692394	KIAA1024	0.753699735	8.95E-09
ILMN_1664177	ATXN7L2	0.75362253	9.59E-11
ILMN_1728517	FNTB	0.752898735	0.000000827
ILMN_1793712	SCAMP3	0.752871692	0.00000122
ILMN_2112460	MAD2L1	0.752403544	0.00000197
ILMN_1789508	GTF3C3	0.752362079	0.000000985
ILMN_1763540	PSME4	0.751884341	0.000000722
ILMN_1739236	ZNF668	0.751183417	4.07E-08
ILMN_1686610	APBA3	0.751096755	0.000000234
ILMN_2269136	AGAP3	0.750566336	0.000000261
ILMN_2328433	NOP2	0.750469554	0.00000143
ILMN_2375651	SCNM1	0.750046128	0.00000102
ILMN_1774890	LAS1L	0.750029398	0.00000253
ILMN_2384513	C2CD2	0.750025151	0.00000156
ILMN_1784948	SPOCD1	0.749604116	0.000000241
ILMN_1651405	BRD9	0.749041522	0.00000204
ILMN_1776925	PRSS22	0.749015584	0.000000136
ILMN_2372413	BID	0.748955979	0.000000458
ILMN_1677887	PMS2	0.748925948	1.66E-08
ILMN_1804490	PRKRIP1	0.748794306	0.000000423

ILMN_1776073	CCT4	0.748245971	0.00000423
ILMN_1722059	SAFB	0.74777662	0.00000159
ILMN_1783843	IIP45	0.747208903	0.000000457
ILMN_1786469	FBXO22	0.747208556	0.00000158
ILMN_1697736	EXOSC2	0.747183018	0.000000906
ILMN_1671288	ODF2	0.746946009	0.00000166
ILMN_2199284	ANAPC7	0.746808793	0.000000737
ILMN_1715994	HGS	0.746697388	0.000000376
ILMN_1761479	ZC3HC1	0.746504877	0.00000222
ILMN_2298261	RBM3	0.74619603	0.00000224
ILMN_2408400	NSUN5	0.745701284	0.00000342
ILMN_1757230	ZNF250	0.745212619	0.000000131
ILMN_2353431	ANKRD16	0.745175729	2.45E-09
ILMN_1701131	C2orf49	0.744945537	0.00000326
ILMN_1781999	ABCF2	0.744676125	0.000000037
ILMN_2401436	PKMYT1	0.743655041	0.00000107
ILMN_2396648	EXOSC1	0.743615553	4.05E-08
ILMN_1695317	RCBTB1	0.743484737	0.000000084
ILMN_1812478	ZNHIT3	0.743380273	0.000000407
ILMN_1730999	TPR	0.742517792	0.00000391
ILMN_1779584	UTP18	0.742393637	0.000000411
ILMN_1680091	POP7	0.742112864	0.00000312
ILMN_1738819	EFTUD2	0.742087824	0.00000431
ILMN_1773935	TMEM165	0.741783375	0.00000206
ILMN_1687359	MRPS23	0.741399075	0.00000106
ILMN_1696556	URB2	0.740097747	0.000000131
ILMN_2143566	SLC39A6	0.739969699	2.15E-08
ILMN_1735798	OSBPL3	0.739861781	6.21E-13
ILMN_1743049	PWP1	0.739204969	0.000000988
ILMN_1789775	WDR74	0.739155326	0.000000148
ILMN_1761442	WDR24	0.739137728	0.00000127
ILMN_1654289	ELK1	0.738902892	0.000000592
ILMN_1750088	VRK2	0.73886551	3.03E-08
ILMN_1726512	ZSCAN2	0.738850437	0.000000884
ILMN_1730698	ODF2	0.738248267	0.000000258
ILMN_1802458	AGTRAP	0.738139009	3.72E-08
ILMN_1784540	KBTBD2	0.738119674	0.00000297
ILMN_1800654	MCM7	0.73770316	0.000000155
ILMN_1689972	RRP9	0.737361824	0.000000769
ILMN_1807662	IGF2R	0.736995232	0.000000421
ILMN_1759175	DUSP10	0.736734746	0.000000818
ILMN_1660232	PEX5	0.736713911	0.00000116
ILMN_2396947	PSMC3IP	0.736699971	2.52E-09
ILMN_1785926	ZNF621	0.736491653	0.000000155

ILMN_1701403	HIP1	0.73569044	0.000000641
ILMN_2344283	FMO3	0.734813914	0.00000342
ILMN_2085441	FOXD4L1	0.734407751	0.000000108
ILMN_2070210	RNF219	0.733510575	0.00000121
ILMN_2380999	RECQL	0.73341858	0.00000192
ILMN_1660368	TRRAP	0.732460609	0.000000372
ILMN_1767362	ADAMTS6	0.732160897	1.22E-09
ILMN_1733045	RAB36	0.732074384	0.00000289
ILMN_2324994	IKBIP	0.731450033	1.42E-08
ILMN_1698365	NHLRC3	0.730758166	9.91E-08
ILMN_1739441	GANAB	0.730360041	6.07E-08
ILMN_1667043	EIF4A3	0.730107158	0.00000041
ILMN_1815723	NUP35	0.729878915	0.00000214
ILMN_1698038	FAM188B	0.729567673	0.00000141
ILMN_1711311	PODXL	0.728793572	2.57E-08
ILMN_2062370	NOL8	0.728737517	0.00000388
ILMN_2360184	INVS	0.728724591	0.000000169
ILMN_1795678	POLR3C	0.728220086	4.63E-09
ILMN_1756360	RPL35A	0.728153218	0.00000422
ILMN_1733103	LOC285908	0.727710115	0.00000288
ILMN_1757347	KIAA0930	0.727575017	4.36E-10
ILMN_1790062	OTULIN	0.72705948	0.00000371
ILMN_2234873	NME2	0.726915982	2.82E-09
ILMN_1736234	CHTOP	0.726794159	0.000000142
ILMN_1684271	ACBD6	0.726482082	8.24E-09
ILMN_2215631	OTUD6B	0.726223173	0.00000316
ILMN_1669635	NUP85	0.725532417	0.00000143
ILMN_1697544	SLC25A29	0.725188303	1.94E-08
ILMN_2224103	PAPSS1	0.725054115	0.00000211
ILMN_2149053	RIF1	0.725007714	0.00000472
ILMN_1746720	TTC39C	0.724865183	0.00000027
ILMN_1787949	RPS15A	0.724759423	0.00000273
ILMN_1703743	AATF	0.724715025	0.0000039
ILMN_2323633	TPD52L2	0.724513672	6.97E-08
ILMN_1811957	CAMSAP1	0.724417252	0.000000983
ILMN_1802404	ABCC1	0.723810825	0.000000568
ILMN_1692698	VASH2	0.723202325	0.00000385
ILMN_1784785	COPS7B	0.722426255	2.49E-09
ILMN_1691798	ZNF26	0.722382085	0.00000235
ILMN_2389114	FIGNL1	0.722213248	0.000000598
ILMN_1775348	KCNH8	0.721323599	4.88E-08
ILMN_2075818	ZNF598	0.720907253	0.0000023
ILMN_1652540	RELL2	0.720482373	0.000000562
ILMN_2043615	OXLD1	0.720440245	0.00000134

ILMN_1806867	PPM1G	0.720323631	0.00000006
ILMN_1764043	TTL	0.720240589	4.87E-08
ILMN_1709085	GSG2	0.720092627	0.000000429
ILMN_1776858	DUS4L	0.719950009	0.000000305
ILMN_1768480	VGLL4	0.719923312	0.000000238
ILMN_1656807	RPL27	0.719355893	0.00000285
ILMN_2350801	SLC25A29	0.717835695	2.07E-08
ILMN_2219512	NPHP4	0.717630519	0.000000397
ILMN_2047112	DPCD	0.717592313	0.00000175
ILMN_2413041	TEAD4	0.717275342	0.000000904
ILMN_1780292	MSH5	0.716737314	0.000000847
ILMN_1812503	PHKA1	0.71614646	0.000000171
ILMN_2412564	NCBP2	0.716013195	4.97E-08
ILMN_1678490	RILPL2	0.715279535	0.00000303
ILMN_2086064	SNRPC	0.715028925	0.00000418
ILMN_1713867	PMFBP1	0.714570356	0.00000105
ILMN_2317463	INTS1	0.714521788	0.00000206
ILMN_1761044	GNB1L	0.714510506	1.09E-08
ILMN_1742779	CENPL	0.714141533	0.00000143
ILMN_1814859	DDX47	0.714075567	0.000000768
ILMN_1706645	MB21D1	0.71384335	0.00000124
ILMN_1751793	PCNXL2	0.7138319	2.1E-09
ILMN_1707534	NKRF	0.713593436	0.00000119
ILMN_2351611	UBQLN1	0.713394916	0.00000419
ILMN_1767816	APH1B	0.712849695	0.00000111
ILMN_2319344	APEX1	0.712673774	0.000000452
ILMN_1695491	WDYHV1	0.712081028	5.01E-08
ILMN_1741755	TRIM29	0.712017986	0.000000218
ILMN_1705093	ORC5	0.711945351	9.45E-09
ILMN_1681628	ZNF277	0.710464309	0.00000164
ILMN_1706342	ZNF746	0.710233015	1.02E-10
ILMN_1739397	GLMN	0.709733535	1.44E-10
ILMN_1683660	EIF3H	0.709529218	0.000000563
ILMN_2373689	DIAPH3	0.708857171	6.27E-08
ILMN_2401822	FTSJ1	0.708232896	8.96E-09
ILMN_2275533	DIAPH3	0.707638884	4.12E-08
ILMN_1745593	STMN1	0.707537309	0.00000333
ILMN_2334693	NARF	0.707029688	3.51E-09
ILMN_1782897	CAPRIN1	0.706951873	2.73E-08
ILMN_1813374	MFSD12	0.706435486	3.39E-09
ILMN_1794260	KDM2B	0.706325717	0.00000141
ILMN_2334204	ERCC6L	0.706205401	0.0000042
ILMN_1765409	STAM	0.705629514	0.000000801
ILMN_1732577	TMEM216	0.705499145	0.00000227

ILMN_1667050	PRPS1	0.705161429	0.00000432
ILMN_1814106	CYSRT1	0.704653662	0.000000732
ILMN_1737005	SMG9	0.704431535	6.95E-09
ILMN_2395236	CHEK2	0.704197412	8.99E-08
ILMN_1712517	ZNF696	0.704037183	1.27E-09
ILMN_1693145	BUB3	0.703862915	0.000000832
ILMN_2151541	DNAJC10	0.703799513	0.000000931
ILMN_1782403	PRR11	0.703472299	0.00000231
ILMN_1733562	TFB1M	0.702748444	5.69E-08
ILMN_1727740	SYNCRIP	0.702622947	0.000000307
ILMN_1665205	ZNF260	0.702293832	0.00000117
ILMN_1801109	NAA16	0.702136333	2.67E-10
ILMN_1741475	PPP1R35	0.700836229	0.00000303
ILMN_1669390	PPP1R13L	0.700722876	0.00000422
ILMN_2268068	MAPKAP1	0.700565443	0.000000383
ILMN_1701402	IKBIP	0.700070665	4.78E-08
ILMN_1814156	PSMB7	0.699950435	0.000000203
ILMN_1736814	CLNS1A	0.699890931	0.00000148
ILMN_1728435	C12orf45	0.699750923	0.00000154
ILMN_1778238	UTP6	0.699285313	0.000000233
ILMN_1709611	PSMA1	0.699201837	4.76E-08
ILMN_1803593	WNT3	0.69819528	0.000000168
ILMN_2338116	TRAF5	0.698032242	0.00000117
ILMN_1768784	PSMC2	0.698000885	3.78E-10
ILMN_2060770	RAI1	0.697663784	0.00000256
ILMN_1706813	SLC6A14	0.697609547	0.00000115
ILMN_2074860	RN7SK	0.697296304	0.00000274
ILMN_2135175	SNORD36A	0.69693967	3.51E-08
ILMN_2300396	COMMD5	0.696828999	0.000000146
ILMN_1653042	HSD3B7	0.696819219	0.00000385
ILMN_2320250	NOL6	0.696175959	0.000000277
ILMN_1721977	NAA10	0.696174357	0.0000015
ILMN_1741398	TTI1	0.695622614	5.82E-09
ILMN_1719870	FAM72D	0.695367367	0.000000443
ILMN_1655117	WDR19	0.69526834	0.00000443
ILMN_1652017	PPEF1	0.694884377	0.00000036
ILMN_1785198	POLE3	0.694871965	0.000000197
ILMN_1721605	SMYD2	0.694419743	4.08E-08
ILMN_1749410	PKP4	0.694281791	0.0000019
ILMN_2400940	L3MBTL1	0.694190492	0.000000792
ILMN_1655663	P3H4	0.694028893	0.00000111
ILMN_1737396	PSMD14	0.69379377	0.00000128
ILMN_1683441	NCAPD3	0.693426915	0.00000152
ILMN_2121816	GPR137B	0.693194753	0.00000157

ILMN_1724407	TACC3	0.692971757	0.00000293
ILMN_1746137	ST7	0.691293866	0.00000013
ILMN_1669502	E2F3	0.691286401	9.88E-08
ILMN_1723846	METTL21B	0.691095227	0.000000344
ILMN_1775573	DPY19L4	0.691008073	0.00000131
ILMN_1662364	AARS	0.690943151	0.00000329
ILMN_1788377	COL27A1	0.690661663	0.0000017
ILMN_1809292	IMMP2L	0.690607166	0.00000376
ILMN_1658695	NUF2	0.690551095	8.52E-08
ILMN_1705876	NAP1L1	0.690312135	0.00000287
ILMN_1725130	FAM50A	0.690268302	0.000000505
ILMN_1682197	NFXL1	0.690099145	0.000000123
ILMN_1779381	SEC61A2	0.689761208	9.96E-09
ILMN_1671374	C19orf53	0.689754706	0.000000615
ILMN_1716736	CD80	0.68957405	0.00000142
ILMN_1689110	NOB1	0.689475609	1.17E-08
ILMN_1683562	SNRPG	0.689090995	0.00000039
ILMN_1708858	CSNK1E	0.688719992	0.000000595
ILMN_1812940	TRMT1	0.688699355	1.23E-09
ILMN_1708160	KPNA2	0.688597358	0.0000016
ILMN_1695226	DIAPH3	0.688111824	1.57E-10
ILMN_1785161	CHCHD6	0.687208659	2.93E-09
ILMN_1682694	VMA21	0.686777676	0.0000012
ILMN_2192683	DHX37	0.686683798	0.00000146
ILMN_1747506	DHX34	0.685822385	1.6E-10
ILMN_1673962	NUP205	0.685655948	7.62E-09
ILMN_1790757	ADSL	0.685584629	0.000000109
ILMN_1658027	RAD54L	0.685541909	0.000000482
ILMN_2123402	CNPY2	0.685242724	0.000000209
ILMN_1719498	EPHX4	0.684670264	2.34E-08
ILMN_1730491	FMNL2	0.684314722	6.61E-08
ILMN_1682494	RSRC1	0.684033473	0.000000016
ILMN_1787511	THUMPD2	0.684026299	2.6E-12
ILMN_1701243	C10orf2	0.683672244	1.09E-08
ILMN_1799198	OTUB2	0.683668486	0.000000843
ILMN_1680782	PATL1	0.683398587	0.000000671
ILMN_1694731	CLCN7	0.683196335	0.00000185
ILMN_1696713	POLA2	0.683080394	0.00000177
ILMN_2152502	MGC72080	0.683013117	3.05E-10
ILMN_1777745	FAM133B	0.682776756	4.89E-09
ILMN_1654671	LOC653705	0.682686241	0.000000045
ILMN_1769925	C2CD4A	0.682538263	0.00000269
ILMN_1700811	UBFD1	0.682460329	9.58E-12
ILMN_1707484	GEMIN6	0.682335347	7.46E-09

ILMN_2072598	LOC401357	0.682322317	0.00000039
ILMN_1674662	TICRR	0.681634811	0.00000387
ILMN_1764522	LMBR1	0.681555104	0.000000456
ILMN_2081335	COA1	0.681530185	4.64E-09
ILMN_1676625	SS18L1	0.681307942	0.00000174
ILMN_2353202	PTK7	0.68111618	0.000000224
ILMN_1709484	BLM	0.680646599	0.00000348
ILMN_1810992	CAD	0.680377251	1.94E-08
ILMN_1772713	BMS1	0.679627022	5.09E-08
ILMN_1717313	NFKBIE	0.67948826	0.00000147
ILMN_1805481	TRMT11	0.678837148	9.81E-08
ILMN_2413644	TM4SF19	0.678649082	4.91E-09
ILMN_1789112	TMEM145	0.678638832	0.000000253
ILMN_1803124	BIRC5	0.67790744	0.000000359
ILMN_1704305	NIP7	0.677521132	0.00000349
ILMN_1669696	ZNF792	0.676621971	5.02E-09
ILMN_1747303	DDX39A	0.676568245	0.000000299
ILMN_2393994	CSPP1	0.676218931	0.000000653
ILMN_2170515	NTMT1	0.676005388	6.21E-10
ILMN_2082244	FOXK1	0.675954756	5.17E-11
ILMN_1686082	C14orf79	0.675838836	2.12E-10
ILMN_2055330	KIF26B	0.675316986	2.29E-08
ILMN_1750256	ALS2	0.67506021	1.31E-09
ILMN_1662954	CCT7	0.674935068	0.00000342
ILMN_1719611	CCT6A	0.674714209	0.000000609
ILMN_1733956	IARS	0.674017797	0.00000281
ILMN_2055477	EXOSC7	0.673474212	4.91E-08
ILMN_2054725	SUGT1	0.673424587	0.000000985
ILMN_1801869	WDR75	0.673334606	0.00000013
ILMN_2152429	C12orf76	0.672257448	7.02E-09
ILMN_1811877	ANTXR1	0.672101468	1.32E-08
ILMN_1810235	ATP6V1E2	0.672014301	4.81E-09
ILMN_1691809	PSMA1	0.671972785	8.57E-08
ILMN_2091217	FAM150A	0.67151768	9.18E-08
ILMN_1808779	CSTF3	0.670461818	0.000000852
ILMN_1676197	LRP11	0.67045172	0.00000283
ILMN_1661776	CENPJ	0.670447481	0.000000064
ILMN_1746846	TTLL4	0.670447119	1.91E-08
ILMN_1726025	ASXL1	0.670067584	0.00000179
ILMN_2193315	EFCAB11	0.669431888	4.33E-08
ILMN_2141807	KNSTRN	0.668523778	0.00000135
ILMN_2115218	ANKRD10	0.668074612	0.000000576
ILMN_1673991	ATIC	0.667844633	0.00000273
ILMN_2232430	NMD3	0.667356631	0.000000125

ILMN_1652594	ACTR5	0.66735164	1.11E-08
ILMN_2415911	ENOX2	0.667327721	6.38E-08
ILMN_1813840	KDM1A	0.667078847	0.000000432
ILMN_1655229	SLC7A11	0.666647079	5.68E-09
ILMN_2087528	CPSF3	0.666520849	1.36E-11
ILMN_1768293	NUP155	0.666382339	0.000000279
ILMN_1804498	BRAT1	0.666278451	0.00000441
ILMN_1690484	KIAA0895	0.666148265	0.000000683
ILMN_1660426	CPSF4	0.665879901	8.25E-09
ILMN_1677439	GLS2	0.665143209	0.00000101
ILMN_1792672	POLR2D	0.664714919	3.19E-08
ILMN_1789349	UBQLN4	0.664202717	1.67E-09
ILMN_2103591	MORC2	0.664059413	2.56E-10
ILMN_2329569	C9orf116	0.664009805	1.15E-08
ILMN_1659156	ANKRD16	0.663956661	4.32E-09
ILMN_2052598	ARMC10	0.663722584	2.04E-08
ILMN_1695731	TUBG1	0.663677854	0.00000178
ILMN_1797154	AZGP1	0.663624296	0.00000387
ILMN_2265654	UBE2C	0.663487233	0.00000302
ILMN_2331010	TNFRSF10B	0.662431747	7.07E-12
ILMN_1781987	CDK5	0.662248121	2.59E-10
ILMN_2135798	NR2C2AP	0.661934853	9.05E-09
ILMN_1806106	GNL3	0.661541409	0.00000235
ILMN_1764794	PSMB2	0.66128796	3.54E-08
ILMN_2187727	NOC3L	0.660968815	0.00000102
ILMN_2217601	ANXA9	0.660911209	3.16E-09
ILMN_1795918	AGAP3	0.660672189	0.000000268
ILMN_2371700	HAUS7	0.660307972	0.000000129
ILMN_1790625	CBX3	0.660207334	0.00000315
ILMN_2389013	ADRM1	0.659947703	0.00000153
ILMN_1748894	GTPBP3	0.659878917	0.00000369
ILMN_2176063	FCGR1A	0.659874636	0.00000163
ILMN_1715905	DSN1	0.659323482	0.000000453
ILMN_1776088	NAT9	0.658987772	1.28E-09
ILMN_2383305	GPATCH4	0.658837469	0.000000786
ILMN_1803624	TMEM147	0.658776707	0.000000547
ILMN_1689353	APLN	0.658665518	4.43E-08
ILMN_2380850	SDCCAG3	0.658376242	1.18E-08
ILMN_1709483	MRE11A	0.658341039	3.7E-10
ILMN_2231020	TMEM185B	0.658275975	6.86E-09
ILMN_2328972	DNMT3B	0.658193156	1.74E-08
ILMN_1716480	ACD	0.65810319	0.000000162
ILMN_1664167	RPF2	0.657798298	0.00000226
ILMN_1769013	ASGR1	0.657716154	0.000000211

ILMN_1740010	PCNX1	0.657321036	0.00000148
ILMN_1711878	ENOPH1	0.656762223	4.14E-08
ILMN_2352934	ASPH	0.656360894	0.00000301
ILMN_1685661	RRP15	0.656275359	0.00000453
ILMN_2228710	PDCD5	0.656006048	0.000000167
ILMN_1662318	CCDC59	0.655913134	0.000000292
ILMN_1754489	FBXL20	0.655831117	0.000000152
ILMN_2105308	DPM1	0.655623858	0.00000215
ILMN_2392472	CENPA	0.654785115	3.08E-08
ILMN_1669273	PPT1	0.654569987	0.00000272
ILMN_1804812	ANAPC1	0.654518216	0.000000489
ILMN_2117987	TFDP1	0.654389684	0.00000413
ILMN_1786189	NIFK	0.653720942	4.14E-09
ILMN_1736597	TKT	0.653570082	9.61E-08
ILMN_2054392	PPIL1	0.653153459	0.000000158
ILMN_2404906	SGO1	0.652998766	0.000000448
ILMN_1688152	IL27RA	0.652976882	0.00000195
ILMN_1722127	RAD54B	0.652263317	1.94E-08
ILMN_2128128	SHFM1	0.652049719	0.000000415
ILMN_1707858	H2AFZ	0.651928445	0.00000272
ILMN_2044617	MTERF3	0.651697565	2.77E-08
ILMN_1690252	ALKBH2	0.651640682	0.000000208
ILMN_1803317	SPOUT1	0.650403614	0.000000296
ILMN_1676893	ADCY3	0.649903684	0.000000814
ILMN_1759495	XPO5	0.649257928	2.75E-08
ILMN_1700448	SIM2	0.649186957	3.91E-10
ILMN_1693334	P4HA1	0.649023301	0.00000422
ILMN_1652677	FAM89A	0.648637847	9.89E-08
ILMN_1690259	RAE1	0.648560821	5.64E-09
ILMN_2325394	MSH5	0.648533723	1.22E-09
ILMN_1752394	CCNB1IP1	0.647799109	1.52E-09
ILMN_1764096	KYAT1	0.646975524	1.98E-11
ILMN_2050617	CCT6P1	0.646450765	3.28E-09
ILMN_1664931	GTF2E2	0.646025143	6.47E-08
ILMN_2132161	KIF18A	0.645359349	0.000000451
ILMN_1796210	PPRC1	0.64511945	0.000000105
ILMN_1749662	GPX1	0.644253837	0.00000382
ILMN_1776653	SCML1	0.64393391	0.00000332
ILMN_1771039	GTSE1	0.643528135	0.00000211
ILMN_1746206	CEP131	0.643525967	0.000000169
ILMN_1738725	LIF	0.643396088	0.00000248
ILMN_1697440	PRPF4	0.643100738	6.36E-10
ILMN_1703092	RECQL4	0.643046589	0.000000435
ILMN_2109156	RANBP1	0.642741312	0.000000884

ILMN_1669703	TNK2	0.642671731	0.00000167
ILMN_2147114	PSPH	0.642581304	1.42E-10
ILMN_1740487	CMTM7	0.642331721	0.000000444
ILMN_1656540	RUVBL1	0.642073829	4.85E-11
ILMN_1726388	ACBD7	0.641754664	0.00000222
ILMN_1691731	PARP14	0.641288166	0.00000137
ILMN_2413899	MCM10	0.641281134	3.18E-08
ILMN_1687533	SEMA4D	0.641116996	0.00000194
ILMN_1677693	HCAR3	0.640995098	0.000000177
ILMN_1651987	CCDC167	0.640954628	0.000000134
ILMN_1748241	CENPP	0.640771732	2.63E-12
ILMN_1673138	ZBTB33	0.640649643	0.000000176
ILMN_1751330	RBCK1	0.639995834	3.89E-08
ILMN_1660222	MTBP	0.639960817	4.96E-09
ILMN_2347234	PRMT1	0.639959224	0.00000106
ILMN_1762594	NOD2	0.639874509	4.73E-08
ILMN_2344956	ACP1	0.639289175	0.00000124
ILMN_1813836	DARS	0.638958674	1.39E-09
ILMN_1692473	PRMT1	0.638575063	0.00000056
ILMN_1687922	RP9	0.637864768	8.26E-09
ILMN_1748770	CKAP5	0.637393579	9.52E-08
ILMN_1711314	NUDT5	0.636698654	0.000000991
ILMN_2286334	U2SURP	0.636372333	0.000000625
ILMN_2379130	IRAK1	0.635677209	1.29E-08
ILMN_1724497	ABI2	0.635663493	5.61E-11
ILMN_1721138	GRPEL2	0.635526624	0.00000374
ILMN_2396948	PSMC3IP	0.635134353	0.000000162
ILMN_1747943	ZNF695	0.635062158	2.16E-09
ILMN_1797425	DDX55	0.634887426	3.66E-10
ILMN_1706610	PLEKHA9	0.634341516	1.89E-08
ILMN_2066020	TCEB1	0.634048572	0.0000017
ILMN_1753885	YTHDF1	0.633780879	0.000000927
ILMN_2414027	CKLF	0.633591871	0.00000169
ILMN_2415235	CSNK1E	0.633314458	3.05E-08
ILMN_1651506	TGS1	0.632975761	2.45E-11
ILMN_1697614	SNU13	0.632922438	8.45E-08
ILMN_1810474	UBE2I	0.632679761	9.47E-08
ILMN_1741954	SMYD3	0.632609734	5.58E-09
ILMN_1690706	SNRPB2	0.632510121	0.000000188
ILMN_1710428	CDK1	0.63227265	1.16E-08
ILMN_1723709	C9orf116	0.631992547	2.81E-09
ILMN_2383871	ZNF74	0.631901642	8.4E-09
ILMN_1763487	CTLA4	0.631340134	0.00000028
ILMN_1711383	STK4	0.630957095	9.48E-08

ILMN_1677261	LZTS1	0.630651634	3.4E-09
ILMN_1652913	EZH2	0.630455173	0.000000125
ILMN_1755335	C19orf45	0.630371174	0.00000436
ILMN_1656368	ALDH4A1	0.629980466	0.00000335
ILMN_1687958	SLC25A22	0.629932076	0.00000156
ILMN_1788387	UGGT2	0.629779883	4.67E-11
ILMN_1708105	EZH2	0.629719466	0.00000118
ILMN_1762142	CDCA7	0.629704346	0.000000767
ILMN_1771026	GARS	0.629406137	2.12E-08
ILMN_1686748	TMEM9	0.629377819	0.00000114
ILMN_1774836	PLOD3	0.62900132	0.000000373
ILMN_1766637	GLA	0.628771194	4.95E-08
ILMN_1653529	TEX10	0.628655818	3.09E-11
ILMN_1805827	PPA1	0.628567354	0.000000284
ILMN_2200503	NIT2	0.62850095	5.83E-13
ILMN_2347349	CCNB1IP1	0.628374939	1.72E-10
ILMN_2364971	PPP2R5C	0.628171894	0.00000286
ILMN_1665538	SKP2	0.627724899	0.00000224
ILMN_1739429	HS6ST2	0.627674811	0.000000665
ILMN_1736816	SKA3	0.627304862	1.72E-08
ILMN_1809866	WDR74	0.627295583	2.8E-09
ILMN_2221564	LYAR	0.627234617	0.000000104
ILMN_1741422	FUT8	0.627165485	0.000000402
ILMN_2293374	TOP1MT	0.626686274	0.00000219
ILMN_1803110	SF3B3	0.62666045	1.06E-08
ILMN_1799516	DNAJC9	0.626524491	0.00000152
ILMN_1785345	GPR84	0.626273257	0.00000235
ILMN_1734194	EXOSC3	0.626152246	4.05E-11
ILMN_1781721	DDX31	0.625701093	3.33E-11
ILMN_1689097	NAA20	0.625568754	4.73E-09
ILMN_2318638	TGIF1	0.625201645	0.000000469
ILMN_1756162	EXOSC8	0.625095595	0.000000433
ILMN_1687107	RFWD3	0.624199047	4.14E-08
ILMN_1685567	TGDS	0.624005465	2.06E-09
ILMN_2074477	GPR4	0.623550413	0.000000678
ILMN_2261627	CTLA4	0.62345768	1.76E-09
ILMN_1756705	CHTF18	0.622930756	0.00000121
ILMN_1713875	NME1	0.622644313	6.86E-10
ILMN_1700762	PBX4	0.622350059	3.08E-08
ILMN_2112256	TNFRSF4	0.62207781	0.00000351
ILMN_1711023	CDK5RAP1	0.622065042	0.000000174
ILMN_2411559	PUS1	0.622042251	3.53E-09
ILMN_1781479	SUV39H1	0.621633587	0.000000272
ILMN_1800197	MRPL36	0.621306934	0.000000254

ILMN_1681737	TMSB15A	0.620787253	0.00000371
ILMN_1682792	BYSL	0.620694441	7.64E-09
ILMN_1677484	SNAPC4	0.620223141	3.2E-09
ILMN_1665559	CDK2	0.620032176	8.19E-09
ILMN_1738093	RNFT2	0.619777433	0.00000439
ILMN_1675677	TMPRSS3	0.619702236	7.98E-09
ILMN_1796235	UTP4	0.619461124	8.61E-10
ILMN_1767658	RRS1	0.619287916	0.00000407
ILMN_2112811	RPL36A	0.61906575	0.00000268
ILMN_1796490	GRINA	0.618887059	1.74E-08
ILMN_1674874	MFSD10	0.618230476	5.66E-09
ILMN_1775268	HECW2	0.617800807	1.63E-08
ILMN_1653319	MC1R	0.616198451	0.00000258
ILMN_2184640	NOLC1	0.616092095	0.000000956
ILMN_1683740	TOP1MT	0.615669092	1.68E-08
ILMN_2052790	NONO	0.614926429	0.000000408
ILMN_1722502	CCT6A	0.614785251	4.93E-09
ILMN_1811472	KIF23	0.614208844	0.00000375
ILMN_1676899	YEATS2	0.612983799	4.91E-13
ILMN_1793033	RBM28	0.612865067	9.75E-12
ILMN_1745420	PHF19	0.612792612	2.88E-13
ILMN_2343624	METTL13	0.612636318	5.13E-08
ILMN_2045729	WDR12	0.612077025	0.00000002
ILMN_1727055	PARPBP	0.611653294	0.00000143
ILMN_1789492	ZDHHC8	0.611451045	6.61E-09
ILMN_1668822	BATF	0.611446535	0.000000147
ILMN_1695962	SLC12A9	0.611258717	2.82E-09
ILMN_1751789	HNMT	0.610941768	0.000000239
ILMN_1799667	KIF4A	0.610805492	0.000000738
ILMN_2288784	CCDC34	0.610643297	0.000000304
ILMN_1758728	FANCG	0.610352553	1.93E-10
ILMN_1674231	CHAF1B	0.609664184	1.53E-08
ILMN_2214910	EPHB4	0.60949666	2.91E-10
ILMN_1804448	MSI2	0.609474122	9.45E-08
ILMN_1760718	ZMIZ2	0.608794462	2.01E-10
ILMN_1703906	HJURP	0.608635838	0.00000348
ILMN_1740160	PLCG1	0.608461165	0.00000148
ILMN_2188374	XPOT	0.608238802	0.00000146
ILMN_1713088	MSI2	0.607689513	2.36E-09
ILMN_1798952	KDELR3	0.607409615	8.07E-09
ILMN_2354140	NAA20	0.607256347	0.000000178
ILMN_2379469	EIF3B	0.606655637	1.29E-10
ILMN_1792506	PLA1A	0.60471316	0.00000482
ILMN_1744212	INPP5D	0.604008815	4.74E-08

ILMN_1803882	VEGFA	0.603931668	3.85E-08
ILMN_1743065	HACD3	0.60253852	0.00000295
ILMN_1708348	ADAM8	0.602361639	0.00000026
ILMN_2232157	PRELID3A	0.60227472	5.13E-08
ILMN_2374633	ZWILCH	0.60177893	0.00000236
ILMN_1695357	SPDL1	0.600811957	3.31E-08
ILMN_2391765	C6orf48	0.60066625	0.000000527
ILMN_1789384	QSOX2	0.600408701	2.32E-11
ILMN_2414399	NME1	0.60031157	7.08E-09
ILMN_2347592	NMB	0.599040073	0.00000102
ILMN_1712389	CKLF	0.597870806	0.000000188
ILMN_2298860	HAGHL	0.597611303	3.84E-10
ILMN_2209515	SNORD56	0.597300535	8.71E-08
ILMN_1719205	FBL	0.596760264	2.71E-08
ILMN_1662390	ASPHD1	0.596195024	2.37E-09
ILMN_2225709	PTCD1	0.595459924	0.000000003
ILMN_1679476	GART	0.595416188	0.000000589
ILMN_2369785	SNRPD2	0.59539826	3.13E-10
ILMN_1673586	SLC6A6	0.595247813	3.21E-13
ILMN_1803997	SDCCAG3	0.595040294	4.27E-15
ILMN_1655642	FANCI	0.595006354	0.00000112
ILMN_1755620	IER5L	0.59447862	2.53E-11
ILMN_1656452	C16orf59	0.594467671	0.00000106
ILMN_1774823	RPL34	0.594123043	0.000000207
ILMN_1708743	NT5DC2	0.593993706	0.000000469
ILMN_1769517	PRKDC	0.593878364	0.00000386
ILMN_1803036	TARBP1	0.59383816	2.06E-08
ILMN_1788024	PCID2	0.593481926	5.06E-10
ILMN_1796423	CLIC3	0.593121741	0.00000114
ILMN_1753183	CDCA4	0.59275412	1.2E-12
ILMN_2279961	LAMP2	0.592601043	0.00000159
ILMN_1791569	PLXNA1	0.592573625	0.000000247
ILMN_1731353	CHPF	0.592545934	1.89E-08
ILMN_2202423	HELLS	0.591672456	5.87E-08
ILMN_1756676	PHF19	0.591659029	1.22E-13
ILMN_1659725	EXOSC5	0.591491848	5.63E-08
ILMN_1703108	UBE2L6	0.591437596	0.00000188
ILMN_1771903	NUP37	0.591312377	7.86E-09
ILMN_2369580	NPRL3	0.591021251	1.71E-09
ILMN_1694177	PCNA	0.590136923	0.00000113
ILMN_1726456	SLC3A2	0.589561962	8.82E-09
ILMN_2403247	CMTM7	0.589381732	0.000000113
ILMN_1810901	RNASEH2A	0.589319737	6.04E-09
ILMN_1708936	EXOSC3	0.589082745	4.7E-09

ILMN_1797933	MRPL17	0.589051542	1.89E-08
ILMN_1717714	CDKN2A	0.588730959	0.000000129
ILMN_1712998	CORIN	0.588594028	0.000000179
ILMN_1693538	STK36	0.588093312	0.00000124
ILMN_1684045	CDCA4	0.586590081	4.66E-09
ILMN_1714296	DNAH2	0.586324375	0.000000762
ILMN_1708151	LAGE3	0.585985784	6.09E-10
ILMN_1705594	NAT10	0.585576393	1.78E-09
ILMN_1808999	ARHGEF19	0.584808027	9.29E-08
ILMN_1724489	RFC4	0.584777533	6.13E-08
ILMN_2289093	RNF213	0.584355952	0.000000071
ILMN_1702171	LPCAT1	0.584176606	2.84E-10
ILMN_1795026	FAM189B	0.583490535	0.000000165
ILMN_2405628	TOP1MT	0.583258224	0.000000257
ILMN_1810423	RPP40	0.582606245	1.78E-08
ILMN_1752953	BCL2L12	0.582534512	8.78E-09
ILMN_2399523	JAG2	0.582067604	1.52E-08
ILMN_1807833	HM13	0.581707073	0.000000501
ILMN_1800958	TMEM237	0.581631226	2.06E-09
ILMN_1676946	AP3M2	0.581258108	4.37E-08
ILMN_2124352	DCUN1D5	0.581244047	7.11E-10
ILMN_1672496	DNAJA1	0.581123745	2.27E-08
ILMN_1760201	DNMT1	0.581039114	1.23E-09
ILMN_1738681	NUP62	0.580738035	1.31E-12
ILMN_2077550	RACGAP1	0.580490761	0.00000162
ILMN_2222074	PTPN12	0.579769221	7.53E-10
ILMN_1767894	POLB	0.579219645	2.72E-12
ILMN_2215545	CMSS1	0.578396084	3.1E-10
ILMN_1706149	PDCD2L	0.578073911	3.38E-12
ILMN_1767422	POLR1D	0.577993659	8.6E-10
ILMN_1716674	VWA2	0.577220819	0.00000246
ILMN_1676555	TTC26	0.57701648	1.15E-14
ILMN_1664294	P3H1	0.576569729	4.45E-10
ILMN_2285375	SORD	0.576013776	7.48E-08
ILMN_1804851	MRPS17	0.57589557	4.15E-09
ILMN_2399363	CLEC4A	0.575339521	0.00000262
ILMN_2067370	SNRPF	0.574944678	0.000000356
ILMN_1712545	S100A3	0.573751915	8.68E-08
ILMN_1690523	LRRC20	0.573727327	6.62E-08
ILMN_1808391	DUSP4	0.573675201	0.000000119
ILMN_2324056	GNL3	0.573403331	0.000000774
ILMN_1720124	RCC2	0.573391344	2.14E-09
ILMN_1790518	JADE3	0.572008571	1.91E-09
ILMN_2226415	MZT1	0.57140682	1.66E-09

ILMN_1677509	PRR7	0.570881102	2.06E-10
ILMN_1661577	MAFA	0.570836365	0.00000022
ILMN_2099783	ATP6V1F	0.570452802	2.29E-09
ILMN_1808071	KIF14	0.570384724	0.000000141
ILMN_1738712	GPR180	0.569811119	7.85E-14
ILMN_1763842	PTRH1	0.569496267	3.36E-10
ILMN_1699570	TPD52L2	0.568811718	3.13E-10
ILMN_1730084	COMT	0.567408619	0.000000289
ILMN_1727540	C1orf112	0.5670239	1.11E-09
ILMN_2328378	OSBPL3	0.566536597	2.75E-09
ILMN_1784706	GABRE	0.566442728	0.00000245
ILMN_2406084	ITGA11	0.566219684	7.17E-08
ILMN_1656129	SLC39A10	0.565477047	1.24E-08
ILMN_2353633	EMR2	0.564731649	1.93E-08
ILMN_2348367	FGFRL1	0.564685343	2.59E-11
ILMN_1656628	WDR4	0.563854615	2.78E-08
ILMN_1685916	KIF2C	0.563810527	0.00000008
ILMN_1809951	ZNF200	0.563593009	1.49E-09
ILMN_1665792	ITGA2	0.563437945	0.0000011
ILMN_2150654	ZSWIM4	0.563420472	5.68E-12
ILMN_2181060	CKAP2	0.563004231	6.43E-08
ILMN_1727194	CALU	0.562732269	0.00000143
ILMN_1654324	HEYL	0.562576276	0.00000384
ILMN_2110252	NPM3	0.562361354	4.15E-08
ILMN_2392803	COL11A1	0.562111295	0.000000055
ILMN_2061310	ZNF280C	0.561425629	1.45E-09
ILMN_2170949	SNX10	0.561383793	0.000000116
ILMN_1742577	GTPBP4	0.560801126	1.31E-10
ILMN_1664630	CHEK1	0.560444408	0.00000343
ILMN_1684391	PLOD1	0.560424386	6.87E-09
ILMN_1803686	ADA	0.560188033	0.00000063
ILMN_1683598	ACSL4	0.559795972	0.0000032
ILMN_2155172	BRIX1	0.559418388	6.72E-10
ILMN_2330243	NUDT1	0.55928736	0.000000054
ILMN_1757627	ZMYND19	0.557752825	4.52E-12
ILMN_2373728	ASB9	0.557510148	5.95E-08
ILMN_1729288	C1QTNF6	0.557334544	6.48E-11
ILMN_1658926	NOTCH3	0.55702569	0.00000109
ILMN_1671843	PSRC1	0.556775139	4.07E-08
ILMN_1735093	TIMELESS	0.556307014	0.00000012
ILMN_1763907	CENPW	0.555324411	0.000000244
ILMN_1800611	CEP72	0.555156249	1.12E-08
ILMN_1779648	HIST3H2A	0.554964537	1.26E-08
ILMN_1662198	RANGAP1	0.55445661	3.49E-11

ILMN_1731720	PDRG1	0.553997254	9.55E-10
ILMN_2224143	MCM3	0.553928702	0.000000178
ILMN_1800317	WNT5A	0.553382781	0.00000192
ILMN_2391231	SORD	0.553085786	7.97E-12
ILMN_1678493	CHN1	0.55224182	0.000000962
ILMN_1744963	ERO1A	0.551590957	6.05E-08
ILMN_1807277	IFI30	0.550815236	0.0000011
ILMN_1748427	ZNF239	0.550752834	0.00000211
ILMN_1721868	LOC643995	0.550585236	2.79E-09
ILMN_2222234	PRDX4	0.550474401	3.34E-08
ILMN_1669376	DRAM1	0.549832431	0.00000302
ILMN_1675927	NEBL	0.549728923	2.01E-10
ILMN_2101034	CAPN12	0.54742806	0.00000157
ILMN_1781155	LYN	0.547210379	1.15E-08
ILMN_1666305	CDKN3	0.546439871	0.00000479
ILMN_1760160	STX1A	0.546180215	0.000000029
ILMN_2131293	ALG1L	0.545190731	0.00000176
ILMN_2396982	BCL2L12	0.545052638	2.38E-10
ILMN_1793201	HAGHL	0.542928251	6.81E-10
ILMN_1798654	MCM6	0.542652392	0.000000378
ILMN_2254635	ITGAX	0.541897956	0.000000876
ILMN_1766408	CBFB	0.541888926	3.43E-09
ILMN_1803194	GALK1	0.541630633	3.4E-09
ILMN_1670134	FADS1	0.540393954	0.00000023
ILMN_1703433	PLSCR3	0.54032685	9.46E-11
ILMN_1676631	CCNO	0.538538776	1.47E-08
ILMN_1689001	CDK4	0.538219975	8.46E-11
ILMN_1715603	IL23A	0.538137038	2.49E-09
ILMN_1773119	CCNF	0.537505216	0.00000123
ILMN_1743402	SIX4	0.537372676	7.55E-08
ILMN_1776490	C17orf53	0.536835994	5.67E-08
ILMN_1661861	CSF2	0.536751075	0.0000032
ILMN_1674302	PPAT	0.536584256	9.21E-10
ILMN_2285817	FAM89A	0.536241705	8.77E-08
ILMN_1745034	SLC11A2	0.535881115	0.000000915
ILMN_2357134	SPHK1	0.534794925	0.000000512
ILMN_1790100	DDIAS	0.534454364	1.25E-08
ILMN_1708101	LMNB2	0.534266432	0.00000107
ILMN_1737514	KYNU	0.533906364	0.000000559
ILMN_1705750	TGM2	0.533805805	0.000000903
ILMN_1774661	SNRPB	0.533792781	0.000000661
ILMN_1709039	RPL13	0.533487303	5.41E-13
ILMN_1746686	POLR1C	0.533277602	3.57E-09
ILMN_2370365	RFC4	0.533025301	0.000000167

ILMN_1724145	CBX4	0.531669931	6.04E-12
ILMN_2261600	FCGR1B	0.531399354	0.00000116
ILMN_1654319	HAPLN3	0.530742995	2.62E-10
ILMN_1709044	TGIF2	0.530341669	8.11E-11
ILMN_1660973	GAD1	0.529712761	0.000000559
ILMN_1715583	BOP1	0.529269091	1.19E-10
ILMN_1784709	GNPDA1	0.529204146	1.71E-10
ILMN_1781514	PCDH17	0.5281303	5.52E-09
ILMN_1775224	NOS3	0.527433499	9.43E-08
ILMN_2289849	FCGR2A	0.526408832	0.00000228
ILMN_1717490	RPL6	0.526045819	2.71E-09
ILMN_1663195	MCM7	0.525113882	6.19E-08
ILMN_1770290	CNN2	0.525098187	1.87E-10
ILMN_1787762	HEATR1	0.524960825	6.42E-12
ILMN_1671257	DKC1	0.524760175	1.72E-08
ILMN_1720526	CENPN	0.524583236	0.000000196
ILMN_1760280	NXT1	0.523768365	1.29E-09
ILMN_1771051	RPL29	0.522943624	0.00000396
ILMN_1751776	CKAP2L	0.52262694	0.000000806
ILMN_2098446	PMAIP1	0.522259817	9.93E-09
ILMN_1656196	E2F6	0.521713301	1.71E-12
ILMN_1702310	LGR5	0.521681815	0.000000232
ILMN_1664362	SGIP1	0.521421696	0.000000029
ILMN_1740418	CYP27B1	0.521127596	1.47E-11
ILMN_1723158	NOP2	0.520773391	9.19E-12
ILMN_1739645	ANLN	0.520233984	8.45E-10
ILMN_1807491	LAIR2	0.519792542	3.41E-08
ILMN_1657862	AHCY	0.519613039	0.00000466
ILMN_1806951	CSTF3	0.517975983	4.35E-09
ILMN_2317923	TMEM132A	0.517850074	6.56E-15
ILMN_1727288	EVPL	0.517527428	0.000000538
ILMN_1658289	WDR54	0.516887189	4.83E-09
ILMN_1727589	SULT2B1	0.516834915	0.00000131
ILMN_1782247	KAT2A	0.515746164	2.45E-09
ILMN_1784860	RFC3	0.515703263	9.29E-09
ILMN_2413158	PODXL	0.515516504	8.45E-11
ILMN_1651343	ITGA11	0.515185466	2.85E-08
ILMN_1710740	C2	0.514011699	7.79E-10
ILMN_1751028	SERPINH1	0.513793791	0.000000989
ILMN_1796417	ASNS	0.51315117	3.2E-09
ILMN_1758457	TBC1D16	0.511994927	6.08E-12
ILMN_1670841	CPNE1	0.511392845	1.98E-08
ILMN_1801257	CENPA	0.511365572	0.00000206
ILMN_1773963	GNA15	0.51121327	0.000000746

ILMN_1791545	KRT23	0.510880233	0.00000145
ILMN_1740493	TRAF5	0.510395767	1.42E-09
ILMN_1712628	GTF2IRD1	0.51035385	8.38E-11
ILMN_1676515	IMPDH1	0.510000848	1.5E-10
ILMN_2128770	CDR2L	0.508804438	0.00000152
ILMN_2285404	DMKN	0.508734511	0.00000171
ILMN_1699265	TNFRSF10B	0.508137038	1.27E-10
ILMN_1680591	RTKN	0.507998559	2.07E-08
ILMN_1762713	MCEMP1	0.507652042	0.000000452
ILMN_2323933	LAIR2	0.507132905	1.03E-08
ILMN_1757631	DBNDD1	0.505581616	0.00000011
ILMN_1728478	CXCL16	0.505430451	2.46E-08
ILMN_1779353	PUS7	0.504444656	4.04E-10
ILMN_1662658	PUS1	0.504414827	4.22E-10
ILMN_2383484	C19orf48	0.504235004	4.22E-09
ILMN_1751079	TAP1	0.503986285	0.0000018
ILMN_1669550	MAD2L2	0.503870417	1.03E-09
ILMN_1692731	TTYH3	0.503603003	0.000000139
ILMN_1758895	CTSK	0.502249849	0.00000149
ILMN_2048700	ATAD2	0.501497398	0.000000032
ILMN_1808824	NEBL	0.501494547	0.000000121
ILMN_1661264	SHMT2	0.500815816	0.000000015
ILMN_1695658	KIF20A	0.499832481	0.00000349
ILMN_2070072	RPS7	0.499780161	0.000000376
ILMN_2413898	MCM10	0.499631496	3.59E-08
ILMN_1769520	UBE2L6	0.499606253	0.00000177
ILMN_1737157	GRAMD1A	0.498539527	2.59E-09
ILMN_1674706	MTHFD2	0.497706533	1.27E-11
ILMN_1741133	NME1	0.497581896	7.5E-09
ILMN_1663954	NELFCD	0.495976531	5.48E-10
ILMN_2202948	BUB1	0.495701311	0.000000263
ILMN_1795865	FGFRL1	0.495112175	7.12E-09
ILMN_1658143	RFC3	0.495098815	1.33E-08
ILMN_1777325	STAT1	0.494922711	8.28E-09
ILMN_2399893	RPS24	0.49467292	6.56E-08
ILMN_1815745	SOX4	0.493403033	1.99E-09
ILMN_1746465	FJX1	0.492976028	6.77E-12
ILMN_1673721	EXO1	0.492673101	9.55E-08
ILMN_1659990	HILPDA	0.492565176	0.000000012
ILMN_1728298	SBK1	0.491734796	0.00000369
ILMN_1694011	WNT2	0.490531483	3.05E-13
ILMN_2375879	VEGFA	0.490302482	1.01E-08
ILMN_1801504	RUNX1	0.489333327	4.17E-10
ILMN_1693471	FLJ33790	0.488607048	1.68E-10

ILMN_1662470	C10orf35	0.488421519	3.67E-10
ILMN_1688480	CCND1	0.488262451	1.47E-08
ILMN_1786041	ASB9	0.487645473	0.000000202
ILMN_1752622	PROX1	0.487329344	0.00000207
ILMN_1747016	CEP55	0.486787039	3.37E-08
ILMN_2051373	NEK2	0.48630076	0.000000341
ILMN_1784871	FASN	0.485879136	0.00000073
ILMN_2186061	PFKFB3	0.485492546	0.00000225
ILMN_2170814	LAMP3	0.484468114	7.78E-08
ILMN_1711470	UBE2T	0.484007876	3.57E-08
ILMN_2390853	CTSH	0.482065955	0.000000126
ILMN_1665797	CSE1L	0.481895632	9.29E-12
ILMN_1706238	CSE1L	0.480443238	1.71E-11
ILMN_2041046	CKS1B	0.480289479	7.62E-09
ILMN_1787280	AUNIP	0.479949607	2.48E-09
ILMN_1736242	PLEKHG4	0.479019134	0.000000198
ILMN_2044832	NOP56	0.478323103	1.29E-09
ILMN_1807074	MIF	0.478015555	2.7E-11
ILMN_1781454	RAE1	0.477708304	9.55E-10
ILMN_1796949	TPX2	0.4758709	2.73E-08
ILMN_1780060	FCN3	0.475557545	3.07E-09
ILMN_1800573	RPS21	0.475026235	2.94E-09
ILMN_2206722	FER1L4	0.474029967	0.00000288
ILMN_2292646	GAD1	0.472104526	0.00000212
ILMN_2089875	TNFSF4	0.471854403	0.000000296
ILMN_1745964	IRAK2	0.471299224	9.48E-08
ILMN_2349459	BIRC5	0.470847939	0.00000161
ILMN_1712888	HSPH1	0.470060064	7.07E-08
ILMN_1701643	GDPD5	0.469844811	0.000000785
ILMN_1813530	AGT	0.468577258	0.000000486
ILMN_1747911	CDK1	0.468529201	0.00000108
ILMN_1758939	RIPK2	0.467922031	5.93E-14
ILMN_1693014	CEBPB	0.46758669	0.000000115
ILMN_2151114	VSNL1	0.465084863	2.94E-08
ILMN_1808707	FSCN1	0.463188436	0.000000722
ILMN_1759184	C19orf48	0.462686968	6.6E-10
ILMN_1788107	IL11	0.460697026	0.00000155
ILMN_1687768	NCOA7	0.459829083	4.99E-08
ILMN_1751228	TMEM74B	0.459529068	0.00000294
ILMN_1764729	JAG2	0.45889568	2.13E-09
ILMN_2405521	MTHFD2	0.458507545	0.00000003
ILMN_1695404	LY6E	0.4542194	0.00000182
ILMN_1670130	ARID3A	0.454051447	0.0000038
ILMN_2307025	CPNE1	0.452166702	1.31E-08

ILMN_1713499	WISP1	0.452030734	1.91E-12
ILMN_1683450	CDCA5	0.45112963	0.0000031
ILMN_1690105	STAT1	0.449838286	0.000000501
ILMN_1734276	PMEPA1	0.448204342	0.00000297
ILMN_1691717	RHBDF2	0.446492526	4.31E-15
ILMN_2212909	MELK	0.445345151	0.00000271
ILMN_1673352	IFITM2	0.44478918	0.000000107
ILMN_1805750	IFITM3	0.44416557	0.000000968
ILMN_1657766	TRIM29	0.44400998	2.52E-08
ILMN_2373062	RHBDF2	0.442652189	4.22E-15
ILMN_1680955	AURKA	0.442276885	0.00000197
ILMN_1721128	TOMM34	0.442200607	6.98E-10
ILMN_1653028	COL4A1	0.439681477	0.00000101
ILMN_1691364	STAT1	0.439388973	0.000000278
ILMN_1774207	ANGPT2	0.437199456	5.52E-10
ILMN_2161577	CXCL6	0.436071494	0.00000259
ILMN_2406501	SOD2	0.435843984	4.44E-09
ILMN_1796629	EDNRA	0.433723506	2.67E-11
ILMN_1746517	KYNU	0.433154401	0.000000145
ILMN_1737650	DIO2	0.43234289	1.14E-10
ILMN_1668194	LMTK3	0.428961433	7.1E-10
ILMN_1660793	PAQR4	0.427229499	1.35E-09
ILMN_1686097	TOP2A	0.424871872	0.00000456
ILMN_1748093	PAFAH1B3	0.423206476	3.54E-10
ILMN_1765557	OLFML2B	0.422860325	2.97E-08
ILMN_1772521	MTHFD1L	0.42228556	5.37E-14
ILMN_1735156	SLC4A11	0.422175283	1.52E-11
ILMN_1739496	PRRX1	0.421474002	0.000000887
ILMN_1790136	MRGBP	0.421296014	2.91E-14
ILMN_1705301	TEAD4	0.421266188	1.46E-13
ILMN_2057479	EGFL6	0.420897933	9.05E-11
ILMN_2415583	HS6ST2	0.419901841	0.000000158
ILMN_2114568	GBP5	0.419554938	0.000000351
ILMN_1698934	CMTM7	0.417746238	7.65E-09
ILMN_1704286	FXYD5	0.417314635	1.03E-14
ILMN_1787509	HELZ2	0.41476865	1.12E-10
ILMN_2347798	IFI6	0.411944296	0.00000202
ILMN_1733756	COL12A1	0.411486778	0.000000605
ILMN_2367258	SMOX	0.409389325	1.28E-12
ILMN_1661599	DDIT4	0.408289372	3.82E-09
ILMN_2374352	DBNDD1	0.40672669	8.84E-11
ILMN_1679267	TGM2	0.405949539	1.17E-09
ILMN_2072296	CKS2	0.403503348	9.65E-09
ILMN_1791726	TUBB3	0.401780383	4.61E-08

ILMN_1774287	CFB	0.399295589	0.000000142
ILMN_1687384	IFI6	0.398398674	9.11E-08
ILMN_1787981	MFAP2	0.398333649	7.85E-11
ILMN_1779147	ENC1	0.397753555	3.29E-09
ILMN_1741942	STX16	0.397091251	8.47E-10
ILMN_2213136	LEF1	0.3954157	7.38E-11
ILMN_1750373	ANOS1	0.394732934	4.6E-12
ILMN_2336781	SOD2	0.394280802	6.7E-09
ILMN_2309848	FXYD5	0.392367333	6.08E-14
ILMN_1750101	S100A11	0.391207963	5.8E-15
ILMN_1676213	SRPX2	0.389215802	0.00000023
ILMN_1715068	AQP9	0.38523398	0.00000151
ILMN_2162972	LYZ	0.38505857	0.000000155
ILMN_1689329	SCD	0.3846693	0.00000074
ILMN_1672611	CDH11	0.38297325	9.94E-10
ILMN_1775380	SMOX	0.381636753	7.75E-16
ILMN_2143795	MGC4677	0.380933045	4.59E-10
ILMN_2387471	FLJ22184	0.378806857	1.83E-08
ILMN_1793410	SNTB1	0.373063469	4.12E-11
ILMN_1796589	TRIP13	0.371604537	2.04E-10
ILMN_2399919	MLXIPL	0.367244238	0.000000544
ILMN_1741165	SLC11A1	0.367004005	7.09E-08
ILMN_1691290	CELSR3	0.364144871	1.27E-12
ILMN_1735996	NOX4	0.360583971	8.77E-12
ILMN_2402392	COL8A1	0.356311324	1.26E-08
ILMN_1760412	SHISA2	0.355676352	7.69E-10
ILMN_1687319	SLCO1B3	0.353495075	0.000000191
ILMN_1741847	MMP10	0.351028251	0.000000169
ILMN_1811468	IRX3	0.350605468	0.00000152
ILMN_1801246	IFITM1	0.349054767	1.79E-08
ILMN_1677765	LRP8	0.344160228	6.18E-16
ILMN_1682636	CXCL2	0.341674835	4.11E-08
ILMN_1792455	TMEM158	0.340066378	2.18E-08
ILMN_1787897	CXCL1	0.335516686	1.48E-08
ILMN_2388547	EPSTI1	0.334058522	2.1E-10
ILMN_1777397	MSX1	0.330690453	2.06E-11
ILMN_1758164	STC1	0.329058946	1.25E-09
ILMN_1729117	COL5A2	0.328567901	8.03E-09
ILMN_2338323	CDC25B	0.325645226	1.37E-12
ILMN_1727200	SLCO4A1	0.323004579	3.71E-11
ILMN_1780546	OSM	0.322291742	1.51E-08
ILMN_2341661	ETV4	0.319603391	2.34E-14
ILMN_1692938	PSAT1	0.314784363	7.1E-10
ILMN_1741768	TMPRSS3	0.310440619	4.44E-13

ILMN_1670490	PDPN	0.305682924	5.49E-13
ILMN_1666845	KRT17	0.305340814	2.69E-09
ILMN_1691884	STC2	0.296495226	2.32E-11
ILMN_1678842	THBS2	0.29605755	0.00000154
ILMN_1695687	SALL4	0.29584221	2.04E-16
ILMN_1714730	UBE2C	0.294154443	1.19E-08
ILMN_1753830	ETV4	0.287824668	1.33E-14
ILMN_1796316	MMP9	0.287248412	0.000000269
ILMN_2301083	UBE2C	0.286767253	6.37E-09
ILMN_1711566	TIMP1	0.284210939	1.14E-12
ILMN_1774685	IL24	0.281091977	5.19E-08
ILMN_1731206	NKD2	0.280403739	8.84E-10
ILMN_1702973	EVA1A	0.278454629	2.15E-15
ILMN_1685433	COL8A1	0.274621195	1.49E-09
ILMN_1770678	CBX2	0.272591753	5.32E-13
ILMN_1751161	COL7A1	0.264718179	2.72E-08
ILMN_1663866	TGFBI	0.264521892	2.5E-15
ILMN_1656057	PLAU	0.261086122	1.43E-11
ILMN_2232854	FAP	0.260168761	7.03E-12
ILMN_1745356	CXCL9	0.257574679	2.01E-08
ILMN_1661825	TNFRSF6B	0.256553464	3.23E-09
ILMN_1726448	MMP1	0.251064292	1.63E-09
ILMN_2212878	ESM1	0.247393198	1.66E-18
ILMN_2049766	NFE2L3	0.246496861	1.29E-14
ILMN_1721354	KRT6B	0.24574158	0.000000144
ILMN_2188862	GDF15	0.245382825	0.000000282
ILMN_1787815	TRIB3	0.244126733	8.31E-14
ILMN_1677636	COMP	0.240820867	9.94E-09
ILMN_2171384	CXCL5	0.240561509	0.00000244
ILMN_1720373	SLC7A5	0.23911762	6.2E-15
ILMN_1768469	TCN1	0.238594371	0.000000202
ILMN_2117508	CTHRC1	0.23718902	7.85E-13
ILMN_1723412	ASCL2	0.232029945	0.000000734
ILMN_1780255	KLK6	0.23102642	4.79E-08
ILMN_1795190	CLDN2	0.230002199	0.000000942
ILMN_2073758	MMP12	0.229419008	0.0000002
ILMN_1723035	OLR1	0.22467962	6.41E-12
ILMN_2374449	SPP1	0.224258992	0.000000202
ILMN_2109489	GZMB	0.220430052	2.21E-11
ILMN_1672776	COL10A1	0.217901368	5.07E-10
ILMN_2206746	BGN	0.21712236	1.87E-12
ILMN_2104356	COL1A2	0.21631466	3.89E-12
ILMN_1791759	CXCL10	0.211556364	1.1E-09
ILMN_1702363	SULF1	0.211393957	3.4E-11

ILMN_1705814	KRT80	0.204121311	1.02E-14
ILMN_1651354	SPP1	0.198096523	8.68E-08
ILMN_1687978	PHLDA1	0.18278105	1.46E-12
ILMN_1750181	TESC	0.180949977	3.94E-11
ILMN_1739001	TACSTD2	0.18041417	7.79E-09
ILMN_1704294	CDH3	0.1748741	3.8E-17
ILMN_1701308	COL1A1	0.169305678	7.82E-11
ILMN_1678841	UBD	0.157290959	3.23E-11
ILMN_1789507	COL11A1	0.141168489	6.49E-16
ILMN_2331231	TNFRSF6B	0.140479639	5.13E-11
ILMN_1784459	MMP3	0.13419751	3.8E-11
ILMN_1655915	MMP11	0.129756834	4.12E-12
ILMN_1753449	CST1	0.129749045	2.32E-11
ILMN_1724686	CLDN1	0.093109275	1.13E-18
ILMN_2184373	CXCL8	0.078652005	5.61E-13
ILMN_1813704	CEMIP	0.073862301	6.25E-21
ILMN_1669046	FOXQ1	0.062779905	1.78E-17
ILMN_2192072	MMP7	0.05312172	4.6E-16

Overlapping results in TCGA, GSE25070 and CPTAC
43 differentially expressed gene
ATP1A1
ITPK1
SLC44A1
ACADVL
HADHA
SUCLG1
ECH1
HADHB
HADH
ACO2
ACSS2
SUCLG2
PRDX6
CPT1A
HSD17B11
MGLL
ACOX1
ATP8B1
ACAA2

RETSAT
UGP2
CES2
ATP2A3
MAOA
HSD11B2
SLC7A5
SCD
NME1
SULF1
CHPF
TTYH3
SHMT2
FASN
AHCY
PRDX4
ATIC
SLC3A2
PPA1
TKT
COMT
NME2
TAP1
GANAB

11 DEGs were validated in our MS

Gene Symbol	N1	N2	N3	N4	N5	T1	T2	T3	T4	T5	P value
SUCLG2	5.12384	4.149	3.94	4.65	3.699	3.326	2.992	1.887	2.1	0.59	0.00303
UGP2	4.902855	4.754	4.29	4.78	5.089	2.129	2.822	3.482	3.2	2.02	0.00773
SUCLG1	5.594701	4.497	4.91	4.79	4.525	4.48	3.844	3.929	2.4	2.97	0.01174
HSD11B2	3.749481	3.055	2.49	3.12	2.754	0.476	1.025	-1.14	-4	-0.1	0.01175
ATP8B1	0.147437	-0.55	-0.7	0.41	-0.45	-4.28	-9.97	-3.56	-4	-10	0.01256
SLC44A1	-0.49167	-1.72	-0.8	-0.5	-1.19	-1.1	-4.69	-2.15	-2	-3.3	0.01429
MAOA	5.068838	5.024	4.34	5.05	4.476	1.843	3.247	2.543	-2	0.12	0.02256
ECH1	5.377752	4.824	4.94	5.33	4.931	4.232	3.978	4.383	4.6	2.54	0.0254

SULF1	-9.96578	-9.97	-10	-10	-9.97	-1.7	-9.97	0.6	-3	-4.6	0.02566
ACOX1	0.770871	0.684	0.11	0.98	0.958	-0.04	-2.8	-1.96	0.5	-1.5	0.02731
HADH	5.4308	4.359	3.98	5.06	4.539	3.565	4.599	3.148	3.1	2.28	0.04431

Supplementary Table S2 Clinical information for patients with CRC

Patient ID	Gender	TNM_T	TNM_N	TNM_M	Stage
1	Male	T1	N0	M0	I
2	Male	T3	N0	M0	IIA
3	Male	T3	N0	M0	IIA
4	Female	T3	N0	M0	IIA
5	Male	T2	N0	M0	I
6	Male	T3	N0	M0	IIA
7	Male	T3	N0	M0	IIA
8	Female	T3	N0	M0	IIA
9	Male	T3	N0	M0	IIA
10	Male	T3	N0	M0	IIA
11	Male	T3	N0	M0	IIA
12	Female	T3	N0	M0	IIA
13	Male	T3	N0	M0	IIA
14	Male	T3	N0	M0	IIA
15	Female	T3	N0	M0	IIA
16	Male	T3	N0	M0	IIA
17	Male	T2	N0	M0	I
18	Male	T1	N0	M0	I
19	Male	T3	N0	M0	IIA
20	Female	T3	N0	M0	IIA
21	Male	T2	N0	M0	I
22	Male	T2	N0	M0	I
23	Male	T2	N0	M0	I
24	Female	T3	N0	M0	IIA
25	Male	T3	N0	M0	IIA
26	Male	T3	N0	M0	IIA
27	Male	T3	N0	M0	IIA
28	Female	T3	N0	M0	IIA
29	Male	T3	N0	M0	IIA
30	Female	T3	N0	M0	IIA
31	Male	T3	N0	M0	IIA
32	Female	T3	N0	M0	IIA
33	Male	T3	N0	M0	IIA
34	Male	T3	N0	M0	IIA
35	Male	T3	N0	M0	IIA
36	Female	T3	N0	M0	IIA
37	Male	T3	N0	M0	IIA
38	Male	T3	N0	M0	IIA
39	Female	T3	N0	M0	IIA
40	Male	T3	N0	M0	IIA
41	Female	T3	N0	M0	IIA

42	Male	T2	N0	M0	I
43	Male	T2	N0	M0	I
44	Male	T3	N0	M0	IIA
45	Female	T3	N0	M0	IIA

Supplementary Table S3 Clinical information for CRC patients in TMA

IHC score of ACOX1	Gender	Age	OS-Days	Status	TNM_T	TNM_N	TNM_M	Stage	Differentiation
0	Male	68	2916	0	T4	N0	M0	IIB	NA
3	Male	61	2135	0	T3	N0	M0	IIA	modernately
12	Male	36	3070	0	T3	N1	M0	IIIB	modernately
8	Female	74	1987	1	T4	N1	M0	IIIB	well
4	Male	50	1184	1	T3	N2	M0	IIIC	modernately
10	Female	42	1719	1	T2	N1	M0	IIIA	modernately
1	Male	25	2171	0	T3	N0	M0	IIA	NA
11	Male	66	2251	0	T3	N0	M0	IIA	modernately
3	Female	69	2524	0	T3	N1	M0	IIIB	well
11	Male	50	1522	1	T3	N1	M0	IIIB	modernately
2	Female	36	2304	0	T4	N0	M0	IIB	NA
0	Male	68	1337	1	T4	N0	M0	IIB	modernately
4	Male	44	1843	1	T3	N2	M0	IIIC	modernately
0	Male	38	2382	0	T3	N0	M0	IIA	modernately
11	Male	25	2322	0	T3	N0	M0	IIA	modernately
7	Male	47	1381	1	T4	N1	M0	IIIB	modernately
8	Male	67	734	1	T3	N0	M0	IIA	modernately
4	Female	65	1058	1	T3	N2	M0	IIIC	modernately
8	Male	76	1375	1	T3	N0	M0	IIA	modernately
0	Male	68	690	1	T3	N1	M0	IIIB	well
3	Male	46	740	1	T3	N2	M0	IIIC	modernately
3	Male	74	1343	1	T3	N1a	M0	IIIB	modernately
0	Male	32	1061	1	T3	N1	M0	IIIB	modernately
6	Male	65	1664	1	T3	N2	M0	IIIC	modernately
2	Male	37	1289	1	T3	N2	M0	IIIC	modernately
1	Male	57	1295	1	T3	N0	M0	IIA	well
4	Male	79	1048	1	T3	N0	M0	IIA	modernately
0	Male	77	568	1	T4	N1	M0	IIIB	Poorly
2	Female	58	926	1	T3	N2	M0	IIIC	NA
2	Male	66	1935	1	T3	N1	M0	IIIB	modernately
2	Male	62	1665	1	T3	N2	M0	IIIC	NA
4	Female	37	2038	0	T3	N1b	M0	IIIB	modernately
3	Male	64	615	1	T3	N2	M0	IIIC	modernately
0	Male	58	885	1	T3	N1	M0	IIIB	modernately
9	Male	76	936	1	T3	N1b	M0	IIIB	well
4	Male	43	1179	1	T3	N1	M0	IIIB	modernately
0	Male	75	441	1	T4	N0	M0	IIB	modernately
3	Male	36	2667	0	T3	N0	M0	IIA	modernately
2	Male	50	295	1	T3	N2	M0	IIIC	Poorly
1	Male	54	840	1	T3	N1	M0	IIIB	modernately
0	Female	61	3089	0	T3	N2	M0	IIIC	Poorly

3	Male	43	2233	1	T3	N2	M1	IV	well
2	Female	69	472	1	T4	N1	M0	IIIB	modernately
2	Male	56	3002	0	T3	N0	M0	IIA	well
0	Male	46	350	1	T3	N1	M0	IIIB	NA
11	Male	61	1294	1	T3	N1	M0	IIIB	modernately
7	Male	62	1008	1	T3	N0	M0	IIA	well
7	Male	60	1966	1	T3	N1	M0	IIIB	NA
6	Male	57	526	1	T3	N0	M0	IIA	well
4	Male	64	544	1	T3	N0	M0	IIA	well
4	Male	49	659	1	T3	N2	M0	IIIC	Poorly
4	Male	60	259	1	T4	N2	M0	IIIC	Poorly
4	Male	83	806	1	T3	N1b	M0	IIIB	modernately
4	Female	69	892	1	T4	N0	M0	IIB	modernately
3	Male	47	1652	1	T3	N1	M0	IIIB	well
3	Male	26	442	1	T4	N2	M0	IIIC	Poorly
3	Female	69	400	1	T4	N2	M0	IIIC	modernately
3	Female	70	516	1	T3	N2	M0	IIIC	modernately
3	Female	44	927	1	T3	N1	M0	IIIB	modernately
3	Male	60	899	1	T3	N1	M0	IIIB	modernately
2	Male	66	937	1	T4	N2	M0	IIIC	modernately
0	Male	48	974	1	T3	N2	Mx	NA	Poorly
0	Male	74	340	1	T3	N0	M0	IIA	Poorly
0	Male	66	1660	1	T3	N0	M0	IIA	modernately
12	Male	56	2270	0	T3	N1	M0	IIIB	well
12	Male	68	2075	0	T3	N1	M0	IIIB	well
12	Male	51	2245	0	T3	N1	M0	IIIB	modernately
12	Male	61	2357	0	T3	N1	M0	IIIB	modernately
12	Male	67	2612	0	T3	N0	M0	IIA	modernately
12	Male	53	2201	0	T3	N0	M0	IIA	modernately
11	Female	46	1921	0	T2	N1	M0	IIIA	well
11	Female	61	2381	0	T1	N1	M0	IIIA	well
11	Male	32	2467	0	T3	N0	M0	IIA	well
11	Male	51	2734	0	T3	N1	M0	IIIB	modernately
11	Female	57	2346	0	T3	N0	M0	IIA	modernately
10	Male	65	2312	0	T2	N1	M0	IIIA	well
10	Male	67	2208	0	T3	N0	M0	IIA	modernately
9	Male	51	2116	0	T3	N1	M0	IIIB	well
9	Male	62	2354	0	T3	N1	M0	IIIB	well
9	Male	59	1835	0	T3	N0	M0	IIA	well
9	Male	54	2345	0	T4	N0	M0	IIB	modernately
9	Female	62	1798	0	T3	N0	M0	IIA	modernately
9	Male	33	2555	0	T3	N0	M0	IIA	modernately
9	Female	44	2685	0	T3	N0	M0	IIA	modernately
8	Male	18	2801	0	T3	N1	M0	IIIB	well

8	Male	39	2268	0	T3	N0	M0	IIA	well
8	Male	49	2240	0	T3	N1	M0	IIIB	modernately
8	Male	38	2297	0	T3	N1	M0	IIIB	modernately
8	Female	62	2527	0	T3	N1	M0	IIIB	modernately
8	Male	71	2274	0	T3	N0	M0	IIA	modernately
7	Female	62	2501	0	T3	N1	M0	IIIB	well
7	Female	54	2311	0	T3	N0	M0	IIA	well
7	Male	58	1961	0	T3	N0	M0	IIA	well
7	Female	50	2285	0	T3	N0	M0	IIA	well
7	Female	59	2197	0	T3	N0	M0	IIA	well
7	Female	61	2447	0	T3	N1	M0	IIIB	NA
7	Male	61	2683	0	T3	N2	M0	IIIC	modernately
7	Female	74	2292	0	T3	N1a	M0	IIIB	modernately
7	Female	44	2365	0	T2	N1	M0	IIIA	modernately
7	Male	58	2073	0	T3	N0	M0	IIA	modernately
6	Male	40	2981	0	T3	N0	M0	IIA	well
6	Female	48	2259	0	T3	N1	M0	IIIB	Poorly
6	Male	62	2564	0	T4	N1	M0	IIIB	modernately
6	Male	60	2280	0	T3	N2a	M0	IIIB	modernately
6	Female	48	2284	0	T3	N1	M0	IIIB	modernately
6	Male	48	1576	0	T3	N0	M0	IIA	modernately
6	Male	56	2346	0	T3	N0	M0	IIA	modernately
6	Male	73	2926	0	T3	N0	M0	IIA	modernately
6	Male	42	2753	0	T3	N0	M0	IIA	modernately
6	Male	74	2187	0	T3	N0	M0	IIA	modernately
5	Female	34	2268	0	T3	N2	M0	IIIC	Poorly
5	Female	52	3489	0	T1	N1	M0	IIIA	modernately
5	Male	38	2892	0	T3	N1	M0	IIIB	modernately
5	Male	29	2500	0	T3	N0	M0	IIA	modernately
5	Female	42	1470	0	T3	N0	M0	IIA	modernately
5	Male	68	2680	0	T3	N0	M0	IIA	modernately
5	Male	53	2969	0	T3	N0	M0	IIA	modernately
4	Female	51	2283	0	T3	N1	M0	IIIB	well
4	Female	42	2241	0	T3	N0	M0	IIA	well
4	Female	47	2565	0	T3	N0	M0	IIA	well
4	Male	55	3555	0	T3	N0	M0	IIA	well
4	Male	59	2785	0	T3	N0	M0	IIA	well
4	Male	42	2050	0	T4	N1	M0	IIIB	Poorly
4	Male	43	3309	0	T3	N2	M0	IIIC	Poorly
4	Male	46	3211	0	T3	N1	M0	IIIB	Poorly
4	Female	56	2686	0	T3	N1	M0	IIIB	NA
4	Female	57	2219	0	T3	N0	M0	IIA	NA
4	Male	67	2611	0	T3	N1	M0	IIIB	modernately
4	Male	51	2185	0	T3	N1	M0	IIIB	modernately

4	Female	68	3211	0	T3	N1c	M0	IIIB	modernately
4	Male	60	2759	0	T4	N1	M0	IIIB	modernately
4	Female	76	2297	0	T3	N1	M0	IIIB	modernately
4	Male	66	3045	0	T3	N1	M0	IIIB	modernately
4	Female	69	3055	0	T3	N1	M0	IIIB	modernately
4	Female	57	2262	0	T3	N0	M0	IIA	modernately
4	Male	52	3001	0	T3	N0	M0	IIA	modernately
4	Male	43	2134	0	T3	N0	M0	IIA	modernately
3	Male	64	2211	0	T4	N1c	M0	IIIB	well
3	Female	58	2615	0	T3	N1	M0	IIIB	well
3	Female	56	2592	0	T3	N1	M0	IIIB	well
3	Male	51	2676	0	T3	N1	M0	IIIB	well
3	Male	52	3287	0	T3	N1	M0	IIIB	well
3	Male	46	2851	0	T3	N1	M0	IIIB	well
3	Male	49	2231	0	T3	N1	M0	IIIB	well
3	Female	62	3411	0	T3	N0	M0	IIA	well
3	Female	58	2059	0	T3	N0	M0	IIA	well
3	Male	53	2950	0	T3	N0	M0	IIA	NA
3	Male	59	2396	0	T3	N2	M0	IIIC	modernately
3	Female	51	2402	0	T3	N1	M0	IIIB	modernately
3	Male	61	2678	0	T3	N2	M0	IIIC	modernately
3	Female	55	3143	0	T3	N1	M0	IIIB	modernately
3	Female	60	3000	0	T2	N1	M0	IIIA	modernately
3	Male	55	2698	0	T4	N0	M0	IIB	modernately
3	Male	65	2391	0	T4	N0	M0	IIB	modernately
3	Male	54	2405	0	T3	N0	M0	IIA	modernately
3	Male	56	2926	0	T3	N0	M0	IIA	modernately
3	Female	67	2058	0	T3	N0	M0	IIA	modernately
2	Male	55	2613	0	T3	N2	M0	IIIC	well
2	Male	35	2834	0	T3	N0	M0	IIA	well
2	Male	59	2444	0	T3	N1	M0	IIIB	Poorly
2	Female	56	2228	0	T3	N2	M0	IIIC	Poorly
2	Male	41	2770	0	T3	N2	M0	IIIC	NA
2	Female	65	3355	0	T4	N2	M0	IIIC	NA
2	Female	63	2437	0	T4	N1	M0	IIIB	NA
2	Female	50	2236	0	T3	N0	M0	IIA	NA
2	Female	37	2584	0	T3	N2	M0	IIIC	modernately
2	Female	55	2998	0	T3	N1b	M0	IIIB	modernately
2	Female	47	2313	0	T3	N1	M0	IIIB	modernately
2	Female	69	2057	0	T3	N1	M0	IIIB	modernately
2	Male	42	3491	0	T4	N0	M0	IIB	modernately
1	Female	61	3473	0	T3	N0	M0	IIA	well
1	Female	64	2936	0	T3	NX	M0	IIIB	Poorly
1	Female	78	3633	0	T3	N2	M0	IIIC	NA

1	Male	54	2913	0	T3	N1	M0	IIIB	NA
1	Female	55	2369	0	T4	N0	M0	IIB	NA
1	Female	50	2747	0	T3	N1	M0	IIIB	modernately
1	Male	45	2303	0	T3	N1a	M0	IIIB	modernately
1	Female	62	3094	0	T3	N1	M0	IIIB	modernately
0	Male	55	2547	0	T3	N1	M0	IIIB	well
0	Female	34	2505	0	T3	N2	M0	IIIC	Poorly
0	Female	37	1665	0	T2	N1	M0	IIIA	NA
0	Male	44	1890	0	T4	N0	M0	IIB	NA
0	Female	32	1494	0	T3	N0	M0	IIA	NA
0	Male	56	2742	0	T3	N0	M0	IIA	NA
0	Female	54	3287	0	T4	N1	M0	IIIB	modernately
0	Male	65	2305	0	T3	N1	M0	IIIB	modernately
0	Female	64	3093	0	T3	N1	M0	IIIB	modernately
0	Male	70	2899	0	T3	N1	M0	IIIB	modernately
0	Male	53	2763	0	T3	N1	M0	IIIB	modernately
0	Male	44	2598	0	T3	N0	M0	IIA	modernately
0	Male	45	3150	0	T3	N0	M0	IIA	modernately
0	Male	38	2867	0	T3	N0	M0	IIA	modernately

Supplementary Table S4 List of antibodies used in this study

Protein	Company	Catalog No.	Source	Dilution
ACOX1	Abcam	Ab184032	Rabbit monoclonal antibody	1:1000 (WB), 1:30 (IP) 1:250 (IHC)
ACOX1	Abclonal	A8091	Rabbit polyclonal antibody	1:1000 (WB)
DUSP14	Abnova	Pab4143	Rabbit polyclonal antibody	1:500 (WB), 1:30 (IP), 1:200 (IHC)
DUSP14	Abclonal	A10287	Rabbit polyclonal antibody	1:500 (WB)
c-Myc	Santa Cruz	Sc-40	Mouse monoclonal antibody	1:200 (WB), 1:200 (IP)
β-actin	Affinity	T0022	Mouse monoclonal antibody	1:2000 (WB)
β-actin	Zenbio	200068-8F10	Mouse monoclonal antibody	1:2000 (WB)
P-Thr	Cell Signaling Technology	9386	Mouse monoclonal antibody	1:1000 (WB)
P-Ser	Santa Cruz	Sc-81514	Mouse monoclonal antibody	1:200 (WB)
P-Tyr	Cell Signaling Technology	8954	Rabbit monoclonal antibody	1:2000 (WB)
HA	Sigma	H6908	Rabbit monoclonal antibody	1:5000 (WB)
HA	Abclonal	AE008	Mouse monoclonal antibody	1:2000 (WB)
Myc-tag	Abbkine	A02061	Rabbit polyclonal antibody	1:2000 (WB)
Flag	Proteintech	20543-1-AP	Rabbit polyclonal antibody	1:3000 (WB)
Myc-tag	Huan Bio	R1208-2	Rabbit polyclonal antibody	1:1000 (WB)
Ubiquitin	Huan Bio	ET1609-21	Rabbit monoclonal antibody	1:1000 (WB)
β-catenin	Abcam	Ab32572	Rabbit monoclonal antibody	1:1000 (WB)
β-catenin	Abclonal	A19657	Rabbit monoclonal antibody	1:1000 (WB), 1:500 (IP) 1:200 (IHC)
β-catenin	Cell Signaling Technology	8480	Rabbit monoclonal antibody	1:1000 (WB)

Protein	Company	Catalog No.	Source	Dilution
p-β-catenin S33/S37/T41	Abclonal	Ap0524	Rabbit polyclonal antibody	1:400 (WB)
p-β-catenin S552	Cell Signaling Technology	5651	Rabbit monoclonal antibody	1:1000 (WB)
p-β-catenin S45	Abclonal	Ap0580	Rabbit polyclonal antibody	1:500 (WB)
HRP-conjugated streptavidin	Sangon Biotech	D111054	/	1:200 (WB)
β-Trcp	Cell Signaling Technology	4394	Rabbit monoclonal antibody	1:1000 (WB)

Supplementary Table S5 List of primer and shRNA sequences used in this study

Primers for construction

Insert	Forward Primer (5'>3')	Reverse Primer (5'>3')
Flag-ACOX1	CGCGCGGCCGCATGAACCCGGACCTGCGCAGGGAG	CGCGTCGACGAGCTTGGACTGCAGTGACTTCA
HA-ACOX1	CGCGAATTCGGATGAACCCGGACCTGCGCAGGGAG	CGCCTCGAGTCAGAGCTTGGACTGCAGTGACTTCA
Flag-ACOX1-N	CGCGCGGCCGCATGAACCCGGACCTGCGCAGGGAG	CGCGTCGACGGCAATGGTGCACGCCTTAG
Flag- ACOX1-C	CGCGCGGCCGCATGATCCGATACAGCGCTGTGAG	CGCGTCGACGAGCTTGGACTGCAGTGACTTCA
Flag-ACOX1 S26A	CGCGCGGCCGCATGAACCCGGACCTGCGCAGGGAGCGGGATTCCGCCAGCTTCAACCCGGAGCTGCTTACACACATCCTGGACGGCGCCCCCGAG	CGCGTCGACGAGCTTGGACTGCAGTGACTTCA
Flag-ACOX1 S26D	CGCGCGGCCGCATGAACCCGGACCTGCGCAGGGAGCGGGATTCCGCCAGCTTCAACCCGGAGCTGCTTACACACATCCTGGACGGCGACCCCGAG	CGCGTCGACGAGCTTGGACTGCAGTGACTTCA
Flag-ACOX1 S126/127A	AGAGAAATGGCTGCTTGCAGCCAAAGGACTCCAGATAA	TTATCTGGAGTCCTTTGGCTGCAAGCAGCCATTTCTCT
Flag-ACOX1 K29R	CTGGACGGCAGCCCCGAGAGAACCCGGCGCCGCCGAGAGA	TCTCTCGGCGGCGCCGGGTTCTCTCGGGGCTGCCGTCCAG
Flag-ACOX1 K241R	GATAGACAATGGCTACCTCAGAATGGACAACCATCGTATT	AATACGATGGTTGTCCATTCTGAGGTAGCCATTGTCTATC
Flag-ACOX1 K255/260R	AAAACATGCTGATGAGGTATGCCCAGGTGAGGCCTGATGGCACATAC	GTATGTGCCATCAGGCCTCACCTGGGCATACCTCATCAGCATGTTTT
Flag-ACOX1 K446R	ACGGCTAGGTTCCTGATGAGAAGTTATGATCAGGTGCACTC	GAGTGCACCTGATCATAACTTCTCATCAGGAACCTAGCCGT
Flag-ACOX1 K643R	CGCGCGGCCGCATGAACCCGGACCTGCGCAGGGAG	GAGCTTGGACTGCAGTGACTTCAGGTGCTTGTAAGATTCGTGGACCTCTGCTCTGTTCAGT
Mus-ACOX1	CGCGAATTCATGAATCCCGATCTGCGCAAGG	CGCGCGGCCGCTCAAAGCTTCGACTGCAGGGG
Mus-ACOX1 S26A	CGCGAATTCATGAATCCCGATCTGCGCAAGGAGCGGGCCGCCGCCACCTTCAATCCAGAGTTAATCACGCACATCTTGGATGGTGCTCCGGAGAACAC CCGGCGCCGT	CGCGCGGCCGCTCAAAGCTTCGACTGCAGGGG

Mus-ACOX1 S26D	CGCGAATTCATGAATCCCGATC TGCGCAAGGAGCGGGCCGCCGC CACCTTCAATCCAGAGTTAATC ACGCACATCTTGGATGGTGATC CGGAGAACAC CCGGCGCCGT	CGCGCGGCCGCTCAAAGCTTCG ACTGCAGGGG
Flag-DUSP14	CGCGCGGCCGCATGAGCTCCAG AGGTCACAGCAC	CGCCTCGAGAATCCCCCAGTAA GGCATCAG
HA-DUSP14	CGCGAATTCGGATGAGCTCCAG AGGTCACAGCAC	CGCCTCGAGCTAAATCCCCCAGT AAGGCAT
DUSP14-Dead	GGCCACCTTGGTGCACTCTGCT GCAGGG GTGAGC	GCTCACCCCTGCAGCAGAGTGC ACCAAGGTGGCC
Flag-DUSP14-N	CGCGCGGCCGCATGAGCTCCAG AGGTCACAGCAC	CGCCTCGAGAAAGTACAGTCCA ATGGG
Flag-DUSP14-C	CGCGCGGCCGCATGGACACCGT GGCTGACAAGAT	CGCCTCGAGAATCCCCCAGTAA GGCATCAG
Flag-GSK3β	CGCGCGGCCGCGAAGAGAGTG ATCATGTCAG	CGCGTCGACGGTGGAGTTGGAA GCTGATGC
Flag-β-catenin	CGCGCGGCCGCATGGCTACTCA AGCTG ATTTGATG	CGCGTCGACCAGGTCAGTATCA AACCAGGC
β-catenin C466A	CACTGAGCCTGCCATCGCTGCT CTTCG TCATCTGACC	GGTCAGATGACGAAGAGCAGCG ATGGCAGGCTCAGTG
Flag-CK1	CGCGCGGCCGCATGGCGAGTAG CAGCGGCTCC	CGCGTCGACGAAACCTTTCATGT TACTCT
Flag-c-Myc	CGCGCGGCCGCATGCCCCTCAA CGTTAGC	CGCGTCGACCGCACAAGAGTTC CGTAGCT
ACOX1-TBE WT	CGCAGATCTACCATCCCATCTC AGCCTTC	CGCAAGCTTGGCTCCGAAGGTC AAGAAAC
ACOX1-TBE Mut	TTTCCCAACCCACTGACGGTG	CACCGTCAGTGGGTTGGGAAA
DUSP14-RE WT	CGCAGATCTTTGGTTGAGGACA TCGGGAG	CGCAAGCTTCTAGAGCGCTCCTC CGTGCA
DUSP14-RE Mut	TCGCTTTGCCTCTAACTCGTCTC CCTTCAC	GTGAAGGGAGACGAGTTAGAGG CAAAGCGA

qPCR primers

Amplicons	Forward Primer (5'>3')	Reverse Primer (5'>3')
GAPDH	GGAGCGAGATCCCTCCAAAAT	GGCTGTTGTCATACTTCTCATGG
ACOX1	GGAACTCACCTTCGAGGCTTG	TTCCCCTTAGTGATGAGCTGG
ACOX1-TBE	GAACTCCTGGGCTCAAGTGA	CTTTGCTTTCGCCATCACCG
DUSP14-RE	GGCATGGAGTCAGGACTCTT	GTCGGTGCGAAACGGACGTT
Gapdh	TGACCTCAACTACATGGTCTACA	CTTCCCATTCTCGGCCTTG

Acox1	GCCATTCGATACAGTGCTGTGAG	CCGAGAAAGTGGAAGGCATAGG
Dusp14	TTGCTCAGATCACCTCCTCTC	AGTACAGTCTAATGGGGGCAT
DUSP14	TCAACTGGCCCCAATTTGAGT	CATCAGGTACGCGATACACAG
Ctnnb1	ATGGAGCCGGACAGAAAAGC	TGGGAGGTGTCAACATCTTCTT
CTNNB1	AGCTTCCAGACACGCTATCAT	CGGTACAACGAGCTGTTTCTAC
SOX9	AGCGAACGCACATCAAGAC	CTGTAGGCGATCTGTTGGGG
AXIN2	TACACTCCTTATTGGGCGATCA	TTGGCTACTCGTAAAGTTTTGGT
c-Myc	GTCAAGAGGCGAACACACAAC	TTGGACGGACAGGATGTATGC
CLDN1	CCTCCTGGGAGTGATAGCAAT	GGCAACTAAAATAGCCAGACCT
MMP7	GAGTGAGCTACAGTGGGAACA	CTATGACGCGGGAGTTTAACAT
JUN	TCCAAGTGCCGAAAAAGGAAG	CGAGTTCTGAGCTTTCAAGGT

shRNA sequences

shRNA	Sequences (5'>3')
sh*ACOX1*-1#	GCAGCCAGATTAGTAGAAATT
sh*ACOX1*-2#	GCGCCTGTGTGCAACTCAAAT
Mus-sh*Acox1*-1#	CCTGAAGAAATCATGTGGTTT
Mus-sh *Acox1*-2#	GCAGCCAGATTGGTAGAAATT
sh*DUSP14*-1#	GTATCGCGTACCTGATGAAAT
sh*DUSP14*-2#	ATGCTTAGGGAAGGTTGATAA
shβ-catenin-1#	GCACAAGAATGGATCACAAGA
shβ-catenin-2#	GCTGGTATCTCAGAAAGTGCC
shCtrl	TTCTCCGAACGTGTCACGT
